# Laser photonic-reduction stamping for graphene-based micro-supercapacitors ultrafast fabrication

**DOI:** 10.1038/s41467-020-19985-2

**Published:** 2020-12-03

**Authors:** Yongjiu Yuan, Lan Jiang, Xin Li, Pei Zuo, Chenyang Xu, Mengyao Tian, Xueqiang Zhang, Sumei Wang, Bing Lu, Changxiang Shao, Bingquan Zhao, Jiatao Zhang, Liangti Qu, Tianhong Cui

**Affiliations:** 1 Laser Micro/Nano-Fabrication Laboratory, School of Mechanical Engineering, Beijing Institute of Technology, 10081 Beijing, P.R. China; 2 Beijing Institute of Technology Chongqing Innovation Center, 401120 Chongqing, P.R. China; 3 Key Laboratory of Cluster Science Ministry of Education of China, School of Chemistry and Chemical Engineering, Beijing Institute of Technology, 102488 Beijing, P.R. China; 4 Tianjin Navigation Instruments Research Institute, 300131 Tianjin, P.R. China; 5 Beijing Key Laboratory of Construction-Tailorable Advanced Functional Materials and Green Applications, School of Materials Science & Engineering, Beijing Institute of Technology, 102488 Beijing, P.R. China; 6 MOE Key Laboratory of Bioorganic Phosphorus Chemistry & Chemical Biology, Department of Chemistry, Tsinghua University, 100084 Beijing, P.R. China; 7 Department of Mechanical Engineering, University of Minnesota, Minneapolis, MN 55455 USA

**Keywords:** Batteries, Supercapacitors, Electronic properties and devices, Design, synthesis and processing

## Abstract

Micro-supercapacitors are promising miniaturized energy storage devices that have attracted considerable research interest. However, their widespread use is limited by inefficient microfabrication technologies and their low energy density. Here, a flexible, designable micro-supercapacitor can be fabricated by a single pulse laser photonic-reduction stamping. A thousand spatially shaped laser pulses can be generated in one second, and over 30,000 micro-supercapacitors are produced within 10 minutes. The micro-supercapacitor and narrow gaps were dozens of microns and 500 nm, respectively. With the unique three-dimensional structure of laser-induced graphene based electrode, a single micro-supercapacitor exhibits an ultra-high energy density (0.23 Wh cm^−3^), an ultra-small time constant (0.01 ms), outstanding specific capacitance (128 mF cm^−2^ and 426.7 F cm^−3^) and a long-term cyclability. The unique technique is desirable for a broad range of applications, which surmounts current limitations of high-throughput fabrication and low energy density of micro-supercapacitors.

## Abstract (web summary)

Microfabrication for cost-effective miniaturized energy storage devices remains a challenge. Here, the authors propose a spatially shaped femtosecond laser method, which is ultrafast, one-step, high resolution and large-scale, for use in patterning flexible high-performance micro-supercapacitors.

## Introduction

The growing demand for secure and self-sustainable energy has led to supercapacitors playing an increasingly vital role because of their ultrafast charge and discharge rates and almost infinite lifetimes. The push towards downsized and wearable electronics presents a challenge because these devices require flexible micro-supercapacitors (MSCs)^1,2^. MSCs are ultrathin and transferable, fitting into the miniaturized energy storage systems and delivering ultrahigh power density.

Although tremendous advances have been proposed to fabricate graphene-based supercapacitors, current methodologies (such as electrophoretic deposition technique^3^, photolithography^4^, printing process^5^, chemical vapor deposition^6^, and other chemical methods^7,8^) are inefficient, and often require a high-temperature processing step, masks, and collectors, or multistepped chemical synthesis, limiting their potential for widespread use. Furthermore, the manufacturing accuracy cannot meet current needs. Therefore, developing a one-step, simple, high-resolution and highly efficient method remains a technologically crucial goal in fabricating MSCs^9^. Other groups have presented laser writing methods to fabricate the graphene-based MSCs^10–12^. Some methods involve the use of Bessel beams or multiple-spot laser direct writing^13,14^ to achieve high efficiency. Although the manufacturing efficiency is higher compared with traditional methods, it is still far from meeting the requirements of large-scale rapid production and industrialization.

Furthermore, the energy density of ordinary MSCs is lower than that of microbatteries^10^. Over the past decade, graphene has attracted considerable attention because of its unique two-dimensional structure^15,16^, large theoretical specific surface area^17^, excellent conductivity^18^, and high mechanical flexibility. However, pure graphene is limited by lower capacitance compared with pseudocapacitive materials. Therefore, the addition of pseudocapacitive materials, such as manganese dioxide (MnO_2_), ferroferric oxide (Fe_3_O_4_), and stannic oxide (SnO_2_), can be introduced into graphene-based systems to improve the specific capacitance^19–21^.

Here, we propose a spatially shaped femtosecond laser (SSFL) method that is ultrafast, one-step, high-resolution and large-scale for patterning of laser-induced graphene (LIG)/MnO_2_ flexible MSCs. The SSFL technique differs from previously reported methods, which can be used to directly complete the processing of electrical devices in batches without the cooperation of any other method or the assistance of laser direct writing. The initial Gaussian beam can be made to various beam shapes by using phase modulations, similar to a variable 3D “photonic stamp”, which can pattern the MSCs with designable shapes and photoregulate the chemical reactions to synthesize LIG/MnO_2_. Over 30,000 MSCs can be produced within 10 min, and the MSCs and narrow gaps were dozens of microns and 500 nm. Benefiting from the femtosecond laser’s uniquely ultrahigh peak power (>10^13^ W cm^−2^) and ultrashort irradiation period^22,23^, the composites of LIG/MnO_2_ are synthesized through photomodulation (photochemical and photothermal reduction/oxidation)^24^. Mn^2+^ can facilitate the graphene oxide (GO) reduction process because of its high oxidation potential^25,26^, and it can use the anchor sites provided by GO to be oxidized to MnO_2_ nanoparticles in situ^27^. At the same time, a fluffy porous three-dimensional structure with ultrahigh specific surface area and toughness can be obtained. The resultant MSC has the high areal and volumetric capacities (128 mF cm^−2^ and 426.7 F cm^−3^) and ultra-small time response (0.01 ms). Furthermore, the process does not require oxidants, current collectors, demanding reaction conditions, addition of chemicals or metal foam. The SSFL technique is particularly attractive because it is suitable for numerous material systems benefiting from the universality of materials for femtosecond laser processing. The SSFL strategy has ultrahigh efficiency and consistently fabricates high-performance, high-resolution, and flexible MSCs, which is promising for application to advanced miniaturized electronics, such as microelectromechanical systems. This strategy also provides a pathway for high throughput in the industry and designable large-scale flexible energy storage devices.

## Results

### Fabrication technology for MSCs using the SSFL strategy

**Fig. 1:**
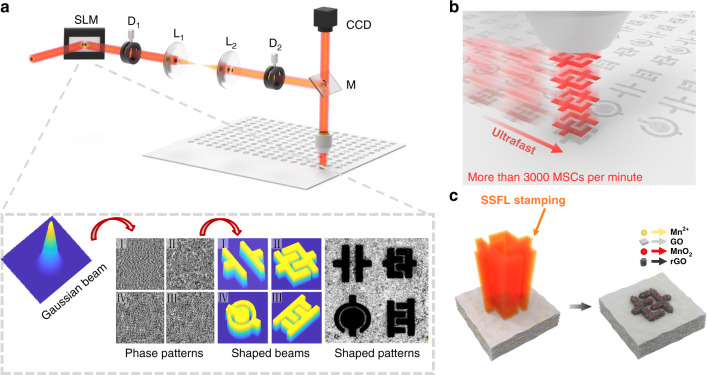
Schematics of SSFL strategy to fabricate the graphene/MnO_2_ MSCs. **a** Gaussian beams were transformed into arbitrary geometric target beams in SLM by programming phase patterns, adjusted collimation by two diaphragms (D1, D2) and transmitted by the 4f relay system including two convex lenses (L1, L2) and the dielectric mirror (M). **b** The ultrafast processing via SSFL on hybrid GO films. **c** Schematic illustration showing the fabrication process for graphene/MnO_2_ MSCs using SSFL strategy.

A complete processing system for flexible and polymorphic MSCs is illustrated in Fig. 1. We reconstructed the optical field distribution of the original Gaussian beam into an arbitrary patterned light field by using a spatial light modulator. Each single pulse laser with the designable patterned spot can directly and instantaneously complete a patterned MSC. The quality of the patterned spot throughout the process was crucial. We used the improved Gerchberg–Saxton algorithm to generate recognizable phases through the formula:

1 $$\varphi _n(x,y) = \varphi ^{(m)}(x,y) = \frac{{\varphi _n(x,y)\varphi _{n - 2}(x,y) - \varphi _{n - 1}^2(x,y)}}{{\varphi _n(x,y) - 2\varphi _{n - 1}(x,y) + \varphi _{n - 2}(x,y)}},$$

where *n* and *m* are the numbers of iterations and phase replacements. While *n* is 0, $\varphi _0(x,y)$ and $\varphi _n(x,y)$ are the initial value and iterative value, respectively. We then combined the phase values $\varphi _n(x,y)$ obtained with the amplitude of the incident light field *F*(*x*,*y*) to generate a complex amplitude *g*_*n*_(*x*,*y*):

2 $$g_n(x,y) = G_n(u,v)\exp \left( {i\psi _n(u,v)} \right) = FT\left[ {F(x,y)\exp \left( {i\varphi _n(x,y)} \right)} \right].$$

The $G_n(u,v)$was replaced in the formula by the expected optical field amplitude *G*_e_(*x*,*y*) and combined with the phase value $\psi _n(u,v)$ to obtain *g*_*n*_(*x*,*y*). According to the Fourier transform and inverse Fourier transform, phase-only holograms were computed by iterating the propagation field between the image and the spatial light modulator plane^28,29^. We optimized the results by reducing the sum-square error of the diffraction optical element for improved beam shaping. The simulation results of optical field optimization, a detailed interpretation and flowchart of the algorithm and the comparison of experimental processing results are presented in Supplementary Figs. 1 and 2.

We used the optimized algorithm to regulate the optical field region, in which the beam width and *z*-axis propagation distance represented the basic parameters of a 3D optical field. We arbitrarily altered the parameters to control the size of the optical field and the fixed depth for 3D processing within a certain range (Supplementary Figs. 3 and 4). An excellent 3D reduction was achieved in the processing of GO hybrid film because the SSFL had a uniform light field on each plane in the *z*-axis direction. Figure 1a reveals that the original Gaussian beam was transformed into a phase pattern after being reshaped and then transmitted by a 4f relay system. This approach avoided the loss of light in the transmission path^30^ and achieved excellent processing results.

The SSFL method achieved ultrafast fabrication of variously shaped MSCs (Fig. 1b) and revolutionizes the traditional processing method of direct laser writing (Supplementary Fig. 5). Traditional laser point-by-point writing of the focal spot is realized by controlling the movement of the translation stage, which considerably limits the method’s application in the ultrafast fabrication of MSCs. In our work, the spot of each laser pulse can be a designable pattern spot shaped by a spatial light modulator that directly and instantaneously completes a patterned MSC. The SSFL strategy not only retains the advantages of being mask-free, flexible and high resolution, it also achieves the ultrafast fabrication of MSCs.

We used the Ti:sapphire laser regenerative amplifier system, which can generate 1000 single pulses per second. In theory, a single MSC could be fabricated in only 1 ms. The actual processing speed observed was more than 3000/min, which is tens or hundreds of times more efficient than previously reported processes for fabricating MSCs^31–36^. The SSFL also has the advantages of high machining accuracy and near faultless processing consistency, enabling rapid and large-scale application. As illustrated in Supplementary Table 1, our SSFL has unprecedented fabrication efficiency and fabricated high-resolution and micron-scale supercapacitors (a detailed manufacturing and a processing video are included in Supplementary movie).

**Fig. 2:**
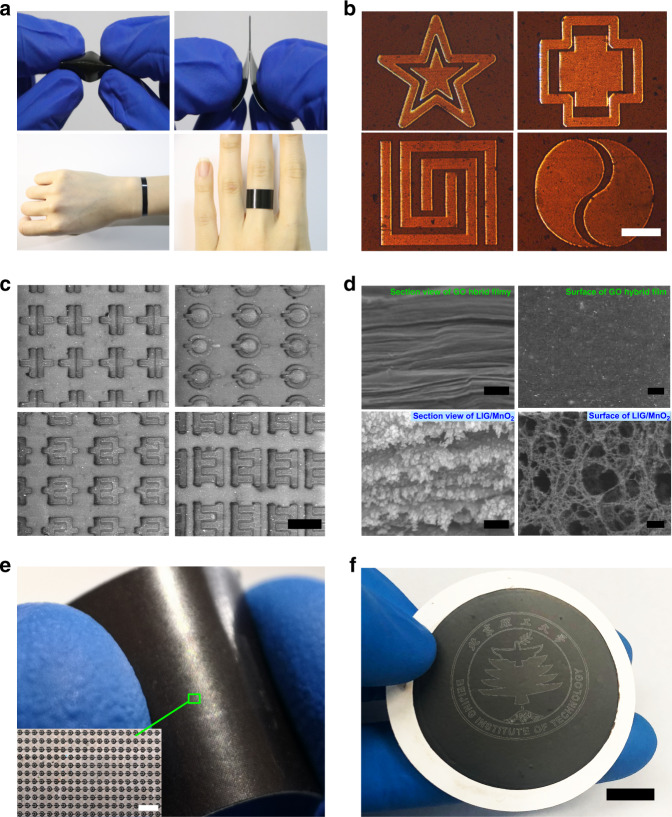
Preparation of GO composite films, and characterizations of the LIG/MnO_2_ MSCs. **a** Photographs of bent and folded GO hybrid film supported by vacuum extraction with different shapes which can be used for wearable energy storage device. **b** Optical micrographs of personalized electrode pattern processed by SSFL strategy (scale bar, 50 μm). **c** The SEM image of MSCs with different shapes (scale bar, 50 μm). **d** SEM image of cross-section and surface view of the GO hybrid film before and after shaped laser ablation (scale bar, 100 nm). **e** More than 30,000 MSCs are fabricated in the 1 cm^2^ (scale bar, 150 μm). **f** More than 100,000 MSCs form a pattern of the school badge (scale bar, 5 mm).

We then vacuum-filtered the GO/manganese acetate hybrid film (approximately a few microns thick). The graphene-based films are self-supporting and flexible, and they could be of different shapes, transferred to arbitrary substrates, which can be used as wearable energy storage devices like bracelets and have good mechanical toughness (Supplementary Fig. 6 and Fig. 2a). Figure 2b and c illustrates various shapes of MSCs, including regular and personalized shapes. These MSCs were tens to hundreds of micrometers in length. The shape of the spot could be designed, and the size of the spot could be regulated by transforming the target light field. Multiple arrays of MSCs in series and in parallel were easily obtained using SSFL (Supplementary Fig. 7). The size of the spot determined the size of the entire MSC, because the MSCs of various shapes were patterned using the SSFL in one step. The varying sizes can be controlled by designable laser spots from 15 × 15 μm^2^ to 100 × 100 μm^2^ (Supplementary Fig. 8). We could transform Gaussian light into shaped light to form the patterned light spots and use the SSFL to extrude the narrow gap of patterns that could break the diffraction limit. MSCs with different narrow gaps could be fabricated by designing different light fields, and an MSC with a narrow gap of 500 nm was fabricated using SSFL on a GO hybrid film (Supplementary Fig. 9). These findings indicate that our method achieved extremely high processing efficiency while maintaining high processing accuracy.

The 3D porous structure irradiated by SSFL could be clearly observed on the cross-sectional view and the surface of the hybrid film before and after the SSFL patterning (Fig. 2d). The original GO layers were stacked tightly. Notably, after SSFL laser ablation, the lamellar graphene fluffed up, and numerous MnO_2_ nanoparticles were attached to LIG. The porous structure resulting from the gas release and the action of 3D light field in the reduction process has the advantages of high surface area, remarkable thermal conductivity, excellent electronic conductivity, and favorable mechanical properties. The Brunauer−Emmet−Teller (BET) specific surface area of LIG/MnO_2_ is eight-times larger than the specific surface area of the GO hybrid film without SSFL ablation, with pore sizes of <5 nm (Supplementary Fig. 10). More importantly, these pores function as shortcuts for rapid ion diffusion between graphene layers and increase the speed of ion transport across the compressed film. The detailed analysis and schematic diagram are in Supplementary Fig. 11. The superiority of this special 3D porous structure can be fully demonstrated in the subsequent electrochemical performance tests.

**Fig. 3:**
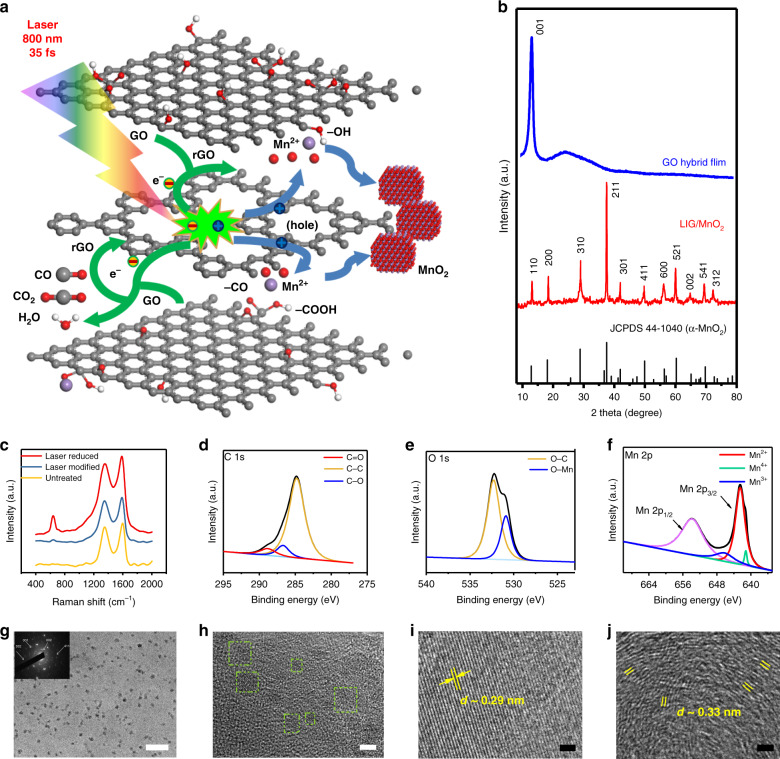
The mechanism and analytical characterization of LIG/MnO_2_ by SSFL. **a** The schematic of the resultant morphology change and phase transformation by SSFL under the proposed photochemical routes. **b** XRD patterns of GO and LIG/MnO_2_. **c** Raman spectra of GO hybrid film untreated, modified and reduced by SSFL. **d**−**f** C 1s, O 1s and Mn 2p XPS core level spectra of LIG/MnO_2_ film. **g** Large area of uniform MnO_2_ nanoparticles scattered on LIG (scale bar, 100 nm). **h** High-resolution TEM (HRTEM) image of MnO_2_ nanoparticles scattered on LIG (scale bar, 2 nm). **i**, **j** HRTEM image of selected area indicated lattice space (scale bar, 2 nm).

To demonstrate the large-scale and consistent fabrication of this technology, we integrated more than 30,000 MSCs in 1 cm^2^ (Fig. 2e) and used hundreds of thousands of MSCs to form the designable pattern (Fig. 2f). Each individual patterned MSC in Fig. 3c, f is processed by a single pulse SSFL. We can easily fabricate tens of thousands of MSCs and make them integrated into a specific pattern by controlling the movement of the translation platform. Notably, the proposed technique can fabricate numerous MSCs within an extremely short time and small area, which is particularly valuable for the practical application of energy storage in microdevices and can be widely extended to other material systems, or other graphene-based composites. We have also used SSFL pattern on Ti_3_C_2_ MXene, MoS_2_, PEDOT, and metal organic framework in one step (Supplementary Fig. 12).

### Mechanism and analytical characterization of LIG/MnO_2_ 3D composite synthesis using the SSFL

The mechanisms of laser reduction presented in this study fall into two main categories: photochemical and photothermal reduction/oxidation. In the initial stage of SSFL interaction with materials, the photochemical reduction/oxidation induces nonthermal ultrafast electron excitation, nonlinear absorption, and subsequent oxygen group removal because of the ultrashort pulse width and ultrahigh intensity of the SSFL. The instantaneous intensity of the SSFL provides sufficient kinetic energy to enable oxygen atoms to leave the graphene sheet without damaging it^24^.

Figure 3a illustrates our proposed photosynthetic route for synthesizing LIG/MnO_2_. SSFL provides numerous excited photons that form free-moving electrons and holes, which reduce GO to rGO. Under their action, GO was reduced to rGO. Mn^2+^ is absorbed on the negatively charged part of the hydrophilic oxygen functional groups of GO because of the electrostatic effect. GO-wrapped Mn^2+^ then facilitates the reduction of GO caused by the high oxidation potential and then uses the anchor sites provided by GO to be oxidized into manganese oxide nanoparticles in situ. The energy released from the deoxygenation of GO serves as an effective and in situ power source driving the oxidation of Mn^2+^ into MnO_2_. The amount of heat released during the deoxygenation of GO is several times that required to drive the endothermic oxidation of metal ions^37,38^. The equations for the reaction are as follows:

3 $$	{\mathrm{(GO) - OH + (GO) - O - (GO) + (GO) - COOH + Mn}}^{{\mathrm{2 + }}} \\ 	\hskip 2pc+ {\mathrm{e}}^{\mathrm{ - }}\mathop{\longrightarrow}\limits^{{hv}}{\mathrm{rGO + MnO}}_{\mathrm{2}}{\mathrm{ + CO}}_{\mathrm{2}} \uparrow {\mathrm{ + CO}} \uparrow$$

As the reaction continues, the photothermal reduction gradually emerges, and the cumulative effect of the pulsed laser produces heat, which results in the pyrolysis of GO, whereby oxygen-containing functional groups such as hydroxyl (−OH), carboxyl (−COOH), and oxygen bridges (C−O−C) are broken up into CO, CO_2_ and H_2_O, which can be removed^39^. When the GO is cracked, the energy and accumulated heat in the reaction provide the original energy for oxidation to the manganese ions, thus completing the formation of manganese oxide:

4 $${\mathrm{GO + Mn}}^{{\mathrm{2 + }}}\mathop{\longrightarrow}\limits^{{\Delta }}{\mathrm{rGO + MnO}}_{\mathrm{2}}{\mathrm{ + CO}}_{\mathrm{2}} \uparrow {\mathrm{ + CO}} \uparrow {\mathrm{ + H}}_{\mathrm{2}}{\mathrm{O}} \uparrow$$

Therefore, the SSFL reduction in the experiment was caused by the combined effect of photochemical and photothermal reactions. Our findings indicated that gas was released when the SSFL reduced GO composite film, which is crucial in the formation of 3D porous structure composites. The 3D porous patterned structure fabricated in one step using the SSFL employed graphene as a solid skeleton, and the uniform effect of the light field enabled manganese dioxide nanoparticles to be evenly distributed on the graphene skeleton. The pores functioned as shortcuts for rapid ion diffusion between graphene layers, which increased the speed of ion transport across the compressed film. The 3D porous scaffold was very stable, which would mitigate mechanical stress within the electrode and thus ensure the stability of long-term cycling of electrochemical energy storage systems^40,41^. Therefore, the LIG/MnO_2_ composite had improved capacitance.

During the SSFL reduction process, the effect of photothermal reduction/oxidation became increasingly apparent as the laser fluence was increased. By altering the laser fluences, LIG/manganese oxides were successfully synthesized through photomodulation of the reaction mechanisms (photochemical and photothermal reduction/oxidation). We varied the laser fluences (170−290 mJ cm^−2^) of the SSFL to investigate differences in conductivity and electrochemical characterization and identify the optimal reduction/oxidation effect. The resistance and conductivity of the material reached their minimum and maximum, respectively, when the laser fluence was 210 mJ cm^−2^ (Supplementary Figs. 13 and 14), which implies that the LIG/manganese oxides composite has large potential capacitance^42^. As expected, the measured area-specific capacitance of the LIG/MnO_2_ MSC was the highest under the laser fluence of 210 mJ cm^−2^ (Supplementary Fig. 15). We investigated whether the high-performance electrode materials were successfully synthesized under the interaction of photochemical and photothermal reduction/oxidation at this laser fluence.

The X-ray diffraction (XRD) patterns of the as-prepared GO and LIG/MnO_2_ nanocomposite synthesized under the laser fluence of 210 mJ cm^−2^ were analyzed (Fig. 3b). The most intensive peak of GO at 2*θ* = 11.2° corresponds to the (001) reflection. The diffraction peaks of LIG/manganese oxides appeared at 2*θ* = 12.73°, 18.14°, 28.82°, 37.46°, 41.95°, 49.86°, 56.32°, 60.27°, 65.11°, 69.76° and 72.73°, which correspond to the expected diffraction peaks of (110), (200), (310), (211), (301), (411), (600), (521), (002), (541) and (312) crystal planes of α-MnO_2_ standard data, respectively, following the Joint Committee on Powder Diffraction Standards card PDF file no. 44-0141. We then collected XRD patterns of different composites (LIG/MnO_2_) synthesized under different laser fluences (Supplementary Fig. 16). The different XRD patterns exhibited similar peaks, which could be all well indexed to plane of the α-MnO_2_ structure. However, when the laser fluence was considerably lower or higher than 210 mJ cm^−2^, one or two other weak peaks appeared, and the peak intensities in the XRD patterns were lower.

The Raman spectra were extremely similar when the laser fluences were between 170 and 290 mJ cm^−2^ (Supplementary Fig. 17). We compared the Raman spectra in three cases(untreated, fs-laser modified, and fs-laser reduced) on the basis of the laser fluences (Fig. 3c). Clear G bands were characteristics of sp^2^ hybridized C–C bonds at 1580 cm^−1^. D bands of residual oxygen functional groups and other defects^43^ in the graphite layer were detected at 1350 cm^−1^, and the high intensity of G peak demonstrated a high C–C bond ratio, confirming the formation of new graphite domains. The peak value at 641 cm^−1^ was caused by Mn−O vibrations perpendicular to the direction of the MnO_6_ octahedral double chains of MnO_2_, indicating the presence of MnO_2_ in the mixed electrode material^44^.

X-ray photoelectron spectroscopy (XPS) was used to assess the difference before (Supplementary Fig.18) versus after SSFL ablation under a laser fluence of 210 mJ cm^−2^ (Fig. 3d−f). The C 1s spectrum consisted of three peaks: C–C/C = C (284.8 eV), C–O (286.7 eV), and C = O (288.8 eV). After SSFL ablation, the C–O and C = O bonds of LIG/MnO_2_ decreased, indicating that certain oxygen-containing groups on the GO hybrid film were removed during the reduction. The O 1s and Mn 2p spectrum further confirmed the presence of MnO_2_. Figure 3g illustrates the peaks centered at 530.9 and 532.3 eV, which correspond to the O–Mn and O–C bonds, respectively^45^. The peaks centered at 653.8 eV could be assigned to the Mn 2p_1/2_, and Mn 2p_3/2_ peaks were observed at three peaks: 641.2, 642.5, and 646 eV, corresponding to +3, +4 and +2 Mn cations, respectively^46^ (Fig. 3f). We also obtained high-resolution Mn 2p spectra of LIG/MnO_2_ under various laser fluences (Supplementary Fig 19). Supplementary Table 2 presents the characteristic peaks and corresponding percentages of Mn^2+^, Mn^3+^, and Mn^4+^. The percentage of Mn^4+^ and Mn^3+^ are the highest (79.4%) and lowest (4%) when the laser fluence was 210 mJ cm^−2^. The percentage of manganese in different valence states could also be adjusted by altering the laser fluences. Mn 3s is more sensitive to the average oxidation state of manganese than Mn 2p was. The energy separation between the two peaks (Δ*E*) was closely related to the mean manganese oxidation state^47,48^. The Mn 3s XPS spectra (Supplementary Fig. 20) were used to assess the Δ*E* of the LIG/MnO_2_ synthesized under various laser fluences. When the laser fluence is 210 mJ cm^−2^, the Δ*E* is 4.75, which is very close to the value of MnO_2_. The different survey spectra (Supplementary Fig. 21) revealed the presence of C, O, and Mn elements, derived from the GO, LIG, and LIG doped with different proportions of MnO_2_. The C/O ratio during the reduction of GO displayed a significant increase under the promotion of SSFL and manganese ions (Supplementary Table 3). Therefore, doping with manganese ions increases the SSFL reduction of GO.

Transmission electron microscopy (TEM) images of LIG/MnO_2_.are presented in Fig. 3g, h. The MnO_2_ nanoparticles were large-scale and uniformly distributed on the graphene sheet layer. A high-resolution TEM image of the MnO_2_ nanoparticles is presented in Fig. 3i. The distance between the lattice fringes was approximately 0.29 nm, which accords with the {001} lattice planes of the α-MnO_2_ crystal^49^. A ripple-like wrinkled structure of graphene can improve the electrochemical performance of devices^50^, and the lattice space of 0.33 nm (Fig. 3j) corresponds to the distance between two neighboring (002) planes in graphitic materials^51^. The mapping and TEM energy dispersive X-ray spectroscopy are illustrated in Supplementary Fig.22.

### Electrochemical performance of the MSCs of different shapes

**Fig. 4:**
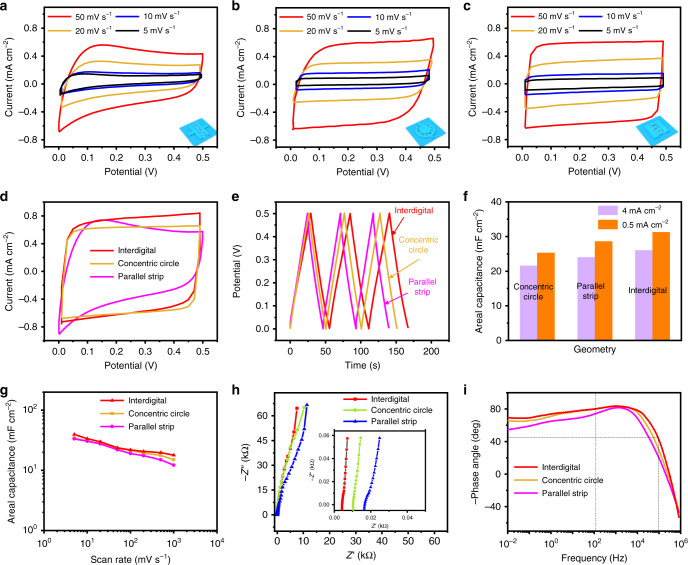
Electrochemical characterization of LIG/MnO_2_ MSCs with different geometries. **a**−**c** CV curves of parallel strip (**a**), concentric circle (**b**), and interdigital (**c**) MSCs at the scan rate from 100 to 1000 mV s^−1^ in 0.5 M Na_2_SO_4_. **d** CV curves of versatile-shaped MSCs obtained at a scan rate of 50 mV s^−1^ in 0.5 M Na_2_SO_4_. **e** The GCD profiles of versatile-shaped MSCs tested at 0.5 mA cm^−2^. **f** The area-specific capacitance of different geometries under high and low current density. **g** The capacitance of three different geometries of MSCs at diverse scan rates. **h** Nyquist plots of the versatile-shaped MSCs. **i** Bode plots of the versatile-shaped MSCs.

Versatile structural interdigital MSC devices with parallel strips and concentric circles were fabricated with specific size parameters (Supplementary Fig. 23) while a constant geometrical area was maintained for the different structures. Notably, the cyclic voltammetry (CV) curves for all three MSC shapes were rectangular at high or low scan rates (Fig. 4a−c and Supplementary Fig. 24), demonstrating the high-quality capacitive performance, shape compatibility, and suitability of MSCs processed using the SSFL method^52^. However, the CV curves of concentric circle-shaped and interdigital MSCs were more regularly rectangular than that of parallel strip-shaped MSCs. Figure 4d intuitively compared the CV curves of versatile-shaped MSCs at a scan rate of 50 mV s^−^^1^. The performance of the interdigital MSC was the most favorable. The galvanostatic charge–discharge (GCD) profiles obtained at 0.5 mA cm^−2^ are depicted in Fig. 4e. Furthermore, we calculated the area-specific capacitance according to the GCD profiles of the MSCs (Fig. 4f). The results revealed that the area-specific capacitance of the interdigital MSCs was higher than that of the other two MSCs. Figure 4g and Supplementary Fig. 25 depict the areal and volumetric capacitance of versatile-shaped MSCs at diverse scan rates: the interdigital MSCs always have a higher capacitance performance. Considering that gravimetric capacitance is a critical factor for industrial applications, we obtained the corresponding gravimetric capacitance (Supplementary Fig. 26) of MSCs with different shapes but the same mass loading at several scan rates. The interdigital MSC exhibited the optimal gravimetric capacitance; however, the parallel strip MSC exhibited excellent gravimetric capacitance, higher than the gravimetric capacitance of the concentric circle MSC. This finding indicates that the shape design of MSCs affected performance. In interdigital MSCs, electrode material areas are used more efficiently and the contact area between the electrode material and electrolytes is greater. Furthermore, interdigital MSCs are interlaced with electrode materials that can shorten the ion diffusion pathway by narrowing the width of the fingers in the MSC and increasing the length of the interface between the active-material electrode and the electrolyte. Therefore, appropriately designing the shapes of an MSC is conducive to fast ion transfer rate, rapid charge and discharge, improved double layer storage, and enhanced rate capability^53^.

Nyquist plots (Fig. 4h) reveal a slope close to 90° in a high-frequency range, indicating excellent capacitance characteristics. All the differently shaped MSCs exhibited this characteristic, suggesting that fabrication using the SSFL strategy was effective. As expected, the interdigital MSCs had the smallest equivalent series resistance (ESR) of 0.85 mΩ cm^−2^. The interdigital MSC device’s ESR was extremely low. No semicircle was detected, which indicates that there was no charge transfer resistance, and the vertical straight line at low frequencies in the Nyquist diagram also confirms that ion diffusion to the electrode and electron transfer during the redox reaction were rapid. The characteristic frequency of the interdigital MSCs at −45° is 94,439.8 Hz (Fig. 4i), corresponding to a time constant (*τ*0) of 0.0106 ms implying the efficient ion transport/electron conductance, which is much smaller than the methane–plasma GO-MSCs (0.28 ms)^54^, focused ion beam-rGO MSCs (0.033 ms)^42^, graphene/CNT MSCs (0.7 ms)^55^ and commercial aluminum electrolytic capacitor (1.1 ms). The ultrafast time constant may be attributed to several factors. First, the MSC fabricated by SSFL is tens of microns in size, and thus charge transfer and energy storage could be accomplished rapidly. Second, the smaller interspace reduced the length of the transport path of ions from one electrode to the counter electrode, resulting in reduced internal resistance and complete utilization of active materials. Third, the porous LIG/MnO_2_ had a large specific surface area and multiple paths, which facilitated ion transmission in the electrodes. The pores functioned as shortcuts for rapid ion diffusion between graphene layers, increasing the speed of ion transport across the compressed film, providing faster paths, providing more path choices for ion transfer, and enabling ions to come into contact with the electrode material quickly and fully. Therefore, the high-resolution MSCs fabricated using SSFL could charge and discharge more rapidly and exhibited an ultrasmall time response (10.6 μs). The MSCs had excellent capacitive behaviors, the phase angle at 120 Hz was measured to be 80.1°, 79.8° and 73.4°, corresponding to interdigital, concentric circle and parallel strip, respectively.

To further verify the electrochemical performance of MSCs, we performed capacitance retention tests on MSCs with different shapes. The interdigital MSC also exhibited the highest rate capability: the capacitance retention was up to 75.5% when the scan speed was increased from 50 to 1000 mV s^-1^ (Supplementary Fig. 27), which is a favorable result in a miniaturized supercapacitor.

### High electrochemical performance of interdigital MSCs

**Fig. 5:**
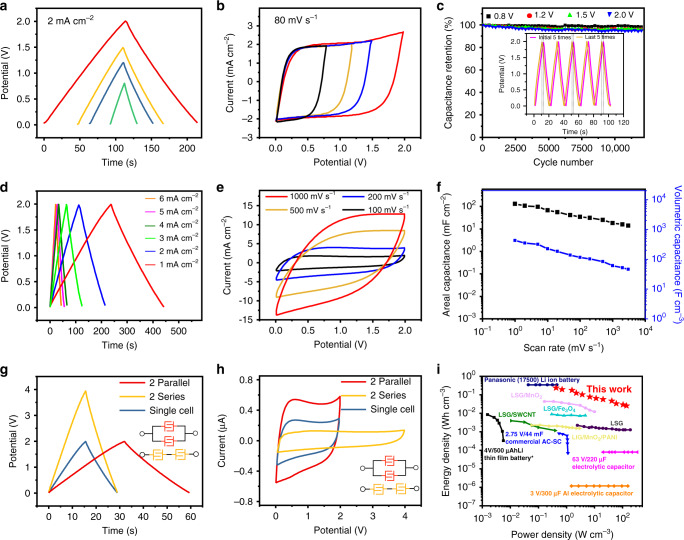
Electrochemical performances of interdigital MSCs. **a** GCD profiles of LIG/MnO_2_ MSCs in 0.5 M Na_2_SO_4_ with different operating voltages from 0.8 to 2 V at 2 mA cm^−2^. **b** CV curves of LIG/MnO_2_ MSCs in 0.5 M Na_2_SO_4_ with different operating voltages from 0.8 to 2 V at a scanning rate of 80 mV s^−1^. **c** Cycle life of the MSCs under different voltage windows. Inset: five GCD curves of interdigital MSCs before and after 12,000 cycles under the voltage windows of 2 V. **d** The GCD profiles of interdigital MSCs at current density from 1 to 6 mA cm^−2^ under 2 V voltage window. **e** CV curves of interdigital MSCs at scan rates ranging from 100 to 1000 mV s^−1^ under 2 V voltage windows in 0.5 M Na_2_SO_4_. **f** The areal capacitance and volumetric capacitance under different scan rates. **g** GCD profiles of a single device, two devices in parallel and two devices in series. **h** CV curves of a single device, two devices in parallel and two devices in series. **i** Energy and power densities of the LIG/MnO_2_ MSCs compared with other capacitors and batteries.

On the basis of our findings, we selected the optimal parameter configuration of the interdigital MSCs for further electrochemical tests. We performed a series of parameter research and optimizations for the interdigital MSCs. We investigated the interdigital gap width, device dimensions, and thickness of the MSCs (Supplementary Figs. 28−30) to explore the effects of these factors on electrochemical performance. There was another fascinating result: the interdigital MSC displayed a high operating voltage in neutral electrolyte. We obtained CV and GCD curves at 80 mV s^−1^ and 2 mA cm^−2^ in an increasing voltage window from 0.8 to 2.0 V to identify the optimal voltage window (Fig. 5a, b). The CV and GCD curves indicate that the symmetric MSCs possess a high working voltage of 2.0 V, which is much higher than that of average MSCs, using the formula of energy density, $E = (1/2)CV_0^2$, where the *E*, *C* and *V*_0_ represent the energy density, capacitance of LIG/MnO_2_ MSC and operating voltage, respectively. An increase of voltage window is of considerable value to MSCs for the high energy density. The operating voltage in aqueous symmetric MSCs is usually lower than 1.2 V because of water splitting at 1.23 V. However, water splitting processes are effectively suppressed in the neutral electrolytes^56–58^. Because the low content of hydrogen ions and hydroxide ions increases the overpotential of gas evolution, a larger operating voltage is obtained. This phenomenon frequently occurs in carbon or graphene-based materials because of the advantage of wide potential windows^59^. Furthermore, MnO_2_ are ideal additives because of their relatively stable voltage plateau^60,61^. MnO_2_ nanoparticles are attached to porous LIG, and the large specific surface area provides a large number of active sites for these hydrogen ions. This open structure and large interlayer distance allow rapid insertion of hydrogen ions, which can suppress the reaction of dihydrogen evolution. We obtained the GCD curves under multiple voltage windows. Figure 5c illustrates that over 95% of the initial capacitance was retained by the MSC after 12,000 cycles, and the first and last five GCD curves were almost consistent under the voltage windows of 2 V.

Figure 5d displayed the GCD curves of interdigital MSCs at various current densities under the voltage windows of 2.0 V. The MSCs had triangular charge−discharge curves. We further obtained the CV curves at different scan rates (1−1000 mV s^−1^) under the voltage windows of 2.0 V (Supplementary Fig. 31 and Fig. 5e). Further, the areal capacitance and volumetric capacitance of LIG/MnO_2_ MSCs were calculated to be 128 mF cm^−2^ and 426 F cm^−3^ (Fig. 5f), respectively. The thickness of the MSC has an intuitive effect on the areal capacitance. In the appropriate range, a larger MSC thickness is associated with better areal capacitance performance. When we increased the thickness of the MSC, its areal capacitance significantly improved. The results are very bright in graphene-based MSCs. Figure 5g, h showed the GCD and CV curves of a single device, two devices in parallel and two devices in series. The MSCs exhibited ideal tandem and parallel capacitive behaviors and excellent performance uniformity. In two series devices, the operating potential window can be doubled to 4 V compared with a single MSC, whereas in the two parallel devices, the discharge time is extended to twice than that of a single MSC.

A Ragone plot compared the energy density with power density of our MSCs and other energy storage device (Fig. 5i). The remarkable energy density of 0.23 Wh cm^−3^ was several orders of magnitude higher than other capacitors or batteries, and much higher than MSCs presented in previous studies (laser-scribed graphene^31^, laser-scribed graphene–MnO_2_^62^, laser-induced graphene/MnO_2_/PANI^63^, rGO/carbon nanotubes supercapacitors^64^ and LSG/Fe_3_O_4_ supercapacitors^65^). Furthermore, our MSCs exhibited excellent performance in power density, reaching 136 W cm^−3^. The electrochemical tests were carried on the MSC fabricated over 30 days and the capacitance performance is almost the same as the newly prepared MSC, which demonstrates the super stability of our MSCs (Supplementary Fig. 32). Furthermore, we obtained the CV curves of the same single interdigital MSC in various bending states (Supplementary Fig. 33). All the CV curves were almost coincident. This outstanding flexibility will expand the use of our MSCs in numerous fields, including integrated circuits, wearable microelectronics, and medical devices.

## Discussion

In conclusion, we have developed a versatile, designable, ultrafast and environmentally friendly SSFL method by merging laser-induced synthesis of 3D porous composite materials with flash MSC patterning. The method could precisely fabricate high-performance MSCs using the uniform patterned laser spot, and obtain excellent results in actual processing with an extremely high processing efficiency. This nanotechnique completely bypasses the masks and collectors and has outstanding universality for MSCs of various electrode materials or other energy storage devices. A unique three-dimensional structure of LIG/MnO_2_ can achieve super stability and huge specific surface area, and be conducive to the rapid transfer of ions. The resultant MSCs had an ultra-small narrow gap (500 nm), were minimal micro size (15 × 15 μm^2^) and had excellent electrochemical performance, such as the high areal and volumetric capacities (128 mF cm^−2^ and 426.7 F cm^−3^), ultra-small time response (0.01 ms), low equivalent series resistance (0.85 mΩ cm^−2^), and ultrahigh energy density of 0.23 Wh cm^−3^, which is approximately hundredfold higher than commercial supercapacitors. Moreover, our MSCs are extremely flexible and designable, and they can be applied to numerous applications, such as stretchable, foldable and integrated electronics. The unprecedented efficiency and high consistency of processing may enable the industrial fabrication of large-scale MSCs with high energy density, driving the widespread use of MSCs in energy storage.

## Methods

### GO hybrid films preparation

We took the graphite oxide (GO) dispersion (0.5 mg/ml,50−200 nm) purchased from XFNANO, Inc. (Nanjing, China) 10 ml and C_4_H_6_MnO_4_(Mn(CH_3_COO)_2_) powder purchased from Beijing DK Nano Technology Co., Ltd. (Beijing, China) 5 mg into the DI water 10 ml. The two solutions underwent exfoliation for 5 h, separately. Then, the two diluted mixed solutions were mixed together, treated by sonication for 2 h and stirred for 1 h. The prepared mixed solution was vacuum-filtered by fiber filter with an aperture of 600 nm. About 6 h of filtration, a layer of mixed film was produced, then it was vacuum-dried. We used acetone solution to dissolve the membrane to obtain independently supported GO hybrid films, and then transfer the GO hybrid films to any substrates.

### Beam shaping

We used a Ti:sapphire laser regenerative amplifier system, which emitted the Gaussian beam with a central wavelength of 800 nm and pulse duration of 35 fs. The spatial light modulator (Holoeye Pluto) can receive the loading phase-difference distribution and reflect the beam out. The designed electrode shape determines the intensity distribution by locating a 256 × 256 pixel area to the black 1080 × 1920 background image. We used the improved Gaussian algorithm and optimize the algorithm by increasing the number of iterations and using functions to optimize the distance between points of beam. So that we can get different expected light fields. Then, loading the gray-scale phase holograms on the SLM can transform the light field of any geometric pattern. The shaped beam was focused by an Olympus objective(20×, NA = 0.45).

### Characterization of LIG/MnO_2_ MSCs

We used an Olympus metallographic microscope to take optical microscopy images. The confocal laser scanning microscopy used an MPLAPONLEXT 20× lens. Scanning electron microscope (SEM) images were obtained by a Hitachi SEM. The crystal phase composition was determined by XRD using a D8 Advance (Bruker) with CuKa radiation. XPS analysis was carried out on a PHI Quantera X-ray photoelectron spectrometer. The Raman spectroscopy investigations were performed using a Renishaw inVia Reflex spectrometer with laser wavelength of 532 nm. CV, GCD, the electrochemical impedance spectra measurements were carried out by a computer-controlled electrochemical workstation (CHI 760D). The TEM was performed using a JEM-2100 TEM.

### Electrochemical characterization of the MSCs

A Precision Probe Station (MPS-100S) with a microscopic system was used to test the electrochemical characterizations, the tungsten probes (tip diameter ≈ 5 µm) were able to make accurate contact with the electrode material. The aqueous electrolyte was 0.5 M Na_2_SO_4_. The capacitance of LIG/MnO_2_ MSCs was calculated by using:

5 $$C = \frac{1}{{\vartheta \times V}}\int _{V_{\mathrm{i}}} ^{V_{\mathrm{f}}} I(V){\mathrm{{d}}}V,$$

where *I* is the current applied, *ϑ* is the scan rate and *V* corresponds to the voltage range (*V*_f_ and *V*_i_ represent final voltage and the initial voltage, respectively). The capacitance of the LIG/MnO_2_ MSCs was also calculated from the galvanostatic charge/discharge curves at different current densities using:

6 $$C = \frac{I}{{( - {\mathrm{d}}V/{\mathrm{d}}t)}},$$

where *I* is the discharging current and d*V*/d*t* is the slope of the discharge curves. We need to pay attention to the fact that the size of our capacitor is tens of microns, so we need to be very careful in the calculation of capacitance. There was no extra collector involved, so we used the above formula to simultaneously calculate the capacitance of LIG/MnO_2_ MSCs (*C*_LIG/MnO2_) and GO hybrid film (*C*_GO_), which were respectively measured with the tungsten probe. The result *C*_cell_ = *C*_LIG/MnO2_ − *C*_GO_ eliminates interference from the tungsten probe and unreduced GO. The volumetric energy density of the LIG/MnO_2_ MSCs were obtained from the equation:

7 $$E_{{\mathrm{{cell}}}} = C_{{\mathrm{{cell}}}}\Delta E^2/(2 \times 3600),$$

where ∆*E* is the operating voltage window. So, the volumetric power density of the LIG/MnO_2_ MSCs was calculated from the equation:

8 $$P_{{\mathrm{{cell}}}} = E_{{\mathrm{{cell}}}} \times 3600/t,$$

where *t* is the discharge time, with *t* = ∆*V*/*ϑ*.

## Supplementary information

Supplementary Information.

Peer Review File

Description of Additional Supplementary Files

Supplementary Movie 1

Supplementary Movie 2

## Data Availability

The data that support the plots within this paper and other findings of this study are available in this Article and its Supplementary Information or from the corresponding author upon reasonable request. Source data are provided with this paper.

## Source data

Source Data

## References

[1] Raccichini R, Varzi A, Passerini S, Scrosati B (2015). The role of graphene for electrochemical energy storage. Nat. Mater..

[2] Yu DS (2014). Scalable synthesis of hierarchically structured carbon nanotube-graphene fibres for capacitive energy storage. Nat. Nanotechnol..

[3] Pech D (2010). Ultrahigh-power micrometre-sized supercapacitors based on onion-like carbon. Nat. Nanotechnol..

[4] Chmiola J (2010). Monolithic carbide-derived carbon films for micro-supercapacitors. Science.

[5] Sirringhaus H (2000). High-resolution inkjet printing of all-polymer transistor circuits. Science.

[6] Chen ZP (2011). Three-dimensional flexible and conductive interconnected graphene networks grown by chemical vapour deposition. Nat. Mater..

[7] Wu ZS (2012). Three-dimensional nitrogen and boron co-doped graphene for high-performance all-solid-state supercapacitors. Adv. Mater..

[8] Yang XW, Cheng C, Wang YF, Qiu L, Li D (2013). Liquid-mediated dense integration of graphene materials for compact capacitive energy storage. Science.

[9] Beidaghi M, Gogotsi Y (2014). Capacitive energy storage in micro-scale devices: recent advances in design and fabrication of micro-supercapacitors. Energy Environ. Sci..

[10] El-Kady MF, Strong V, Dubin S, Kaner RB (2012). Laser scribing of high-performance and flexible graphene-based electrochemical capacitors. Science.

[11] Gao W (2011). Direct laser writing of micro-supercapacitors on hydrated graphite oxide films. Nat. Nanotechnol..

[12] Li R-Z (2016). High-rate in-plane micro-supercapacitors scribed onto photo paper using in situ femtolaser-reduced graphene oxide/Au nanoparticle microelectrodes. Energy Environ. Sci..

[13] Hales JM (2019). Using Bessel beams and two-photon absorption to predict radiation effects in microelectronics. Opt. Express.

[14] Gang Q, Fan LD, Watanabe A (2016). Formation of indium tin oxide film by wet process using laser sintering. J. Mater. Process. Tech..

[15] Augustyn V (2013). High-rate electrochemical energy storage through Li^+^ intercalation pseudocapacitance. Nat. Mater..

[16] Lukatskaya MR (2013). Cation intercalation and high volumetric capacitance of two-dimensional titanium carbide. Science.

[17] Allen MJ, Tung VC, Kaner RB (2010). Honeycomb carbon: a review of graphene. Chem. Rev..

[18] Novoselov KS (2005). Two-dimensional gas of massless Dirac fermions in graphene. Nature.

[19] Ma WJ (2017). Flexible all-solid-state asymmetric supercapacitor based on transition metal oxide nanorods/reduced graphene oxide hybrid fibers with high energy density. Carbon.

[20] Wang XY (2018). Large-area reduced graphene oxide composite films for flexible asymmetric sandwich and microsized supercapacitors. Adv. Funct. Mater..

[21] Chen S, Zhu JW, Wu XD, Han QF, Wang X (2010). Graphene oxide-MnO_2_ nanocomposites for supercapacitors. ACS Nano.

[22] Zhang YL, Chen QD, Xia H, Sun HB (2010). Designable 3D nanofabrication by femtosecond laser direct writing. Nano Today.

[23] Jiang L, Wang AD, Li B, Cui TH, Lu YF (2018). Electrons dynamics control by shaping femtosecond laser pulses in micro/nanofabrication: modeling, method, measurement and application. Light Sci. Appl..

[24] Zhang H, Miyamoto Y (2012). Graphene production by laser shot on graphene oxide: an ab initio prediction. Phys. Rev. B.

[25] Xu M, Kong L, Zhou W, Li H (2007). Hydrothermal synthesis and pseudocapacitance properties of alpha-MnO2 hollow spheres and hollow urchins. J. Phys. Chem. C.

[26] Devaraj S, Munichandraiah N (2008). Effect of crystallographic structure of MnO_2_ on its electrochemical capacitance properties. J. Phys. Chem. C.

[27] Toupin M, Brousse T, Belanger D (2002). Influence of microstucture on the charge storage properties of chemically synthesized manganese dioxide. Chem. Mater..

[28] Skeren M, Richter I, Fiala P (2002). Iterative Fourier transform algorithm: comparison of various approaches. J. Mod. Opt..

[29] Zhang CC (2016). Optimized holographic Femtosecond laser patterning method towards rapid integration of high-quality functional devices in microchannels. Sci. Rep..

[30] Wang AD (2015). Mask-free patterning of high-conductivity metal nanowires in open air by spatially modulated femtosecond laser pulses. Adv. Mater..

[31] El-Kady MF, Kaner RB (2013). Scalable fabrication of high-power graphene micro-supercapacitors for flexible and on-chip energy storage. Nat. Commun..

[32] Zhang CF (2018). Stamping of flexible, coplanar micro-supercapacitors using MXene inks. Adv. Funct. Mater..

[33] Liu YQ (2016). Facile fabrication of flexible microsupercapacitor with high energy density. Adv. Mater. Technol..

[34] Liu ZY (2016). Ultraflexible in-plane micro-supercapacitors by direct printing of solution-processable electrochemically exfoliated graphene. Adv. Mater..

[35] Purkait T (2020). Electrochemically customized assembly of a hybrid xerogel material via combined covalent and non-covalent conjugation chemistry: an approach for boosting the cycling performance of pseudocapacitors. J. Mater. Chem. A.

[36] Jeon H (2018). Facile and fast microwave-assisted fabrication of activated and porous carbon cloth composites with graphene and MnO_2_ for flexible asymmetric supercapacitors. Electrochim. Acta.

[37] Kim F (2010). Self-propagating domino-like reactions in oxidized graphite. Adv. Funct. Mater..

[38] Hwang JY (2015). Direct preparation and processing of graphene/RuO_2_ nanocomposite electrodes for high-performance capacitive energy storage. Nano Energy.

[39] Larciprete R (2011). Dual path mechanism in the thermal reduction of graphene oxide. J. Am. Chem. Soc..

[40] Zhang X (2014). Recent advances in porous graphene materials for supercapacitor applications. Rsc Adv..

[41] Sun HT (2019). Hierarchical 3D electrodes for electrochemical energy storage. Nat. Rev. Mater..

[42] Lobo DE, Banerjee PC, Easton CD, Majumder M (2015). Miniaturized supercapacitors: focused ion beam reduced graphene oxide supercapacitors with enhanced performance metrics. Adv. Energy Mater..

[43] Sumboja A, Foo CY, Wang X, Lee PS (2013). Large areal mass, flexible and free-standing reduced graphene oxide/manganese dioxide paper for asymmetric supercapacitor device. Adv. Mater..

[44] Gao T (2008). Microstructures and spectroscopic properties of cryptomelane-type manganese dioxide nanofibers. J. Phys. Chem. C.

[45] He YM (2013). Freestanding three-dimensional graphene/MnO_2_ composite networks as ultralight and flexible supercapacitor electrodes. ACS Nano.

[46] Lv P, Feng YY, Li Y, Feng W (2012). Carbon fabric-aligned carbon nanotube/MnO_2_/conducting polymers ternary composite electrodes with high utilization and mass loading of MnO_2_ for super-capacitors. J. Power Sources.

[47] Sun M (2013). Controlled synthesis of nanostructured manganese oxide: crystalline evolution and catalytic activities. CrystEngComm.

[48] Galakhov VR (2002). Mn 3 s exchange splitting in mixed-valence manganites. Phys. Rev. B.

[49] Li WY (2014). Effect of temperature on the performance of ultrafine MnO_2_ nanobelt supercapacitors. J. Mater. Chem. A.

[50] Zhu YW (2011). Carbon-based supercapacitors produced by activation of graphene. Science.

[51] Lin J (2014). Laser-induced porous graphene films from commercial polymers. Nat. Commun..

[52] Kamboj N (2019). Ultralong cycle life and outstanding capacitive performance of a 10.8 V metal free micro-supercapacitor with highly conducting and robust laser-irradiated graphene for an integrated storage device. Energy Environ. Sci..

[53] Wu ZS, Parvez K, Feng XL, Mullen K (2014). Photolithographic fabrication of high-performance all-solid-state graphene-based planar micro-supercapacitors with different interdigital fingers. J. Mater. Chem. A.

[54] Wu ZS, Parvez K, Feng XL, Mullen K (2013). Graphene-based in-plane micro-supercapacitors with high power and energy densities. Nat. Commun..

[55] Lin J (2013). 3-dimensional graphene carbon nanotube carpet-based microsupercapacitors with high electrochemical performance. Nano Lett..

[56] Zhao L (2014). Honeycomb porous MnO_2_ nanofibers assembled from radially grown nanosheets for aqueous supercapacitors with high working voltage and energy density. Nano Energy.

[57] Jiang LL (2015). Functional pillared graphene frameworks for ultrahigh volumetric performance supercapacitors. Adv. Energy Mater..

[58] Li HY (2018). Scalable fabrication of hierarchically porous N-doped carbon electrode materials for high-performance aqueous symmetric supercapacitor. J. Mater. Sci..

[59] Chen D, Tang LH, Li JH (2010). Graphene-based materials in electrochemistry. Chem. Soc. Rev..

[60] Zhang F (2013). A high-performance supercapacitor-battery hybrid energy storage device based on graphene-enhanced electrode materials with ultrahigh energy density. Energy Environ. Sci..

[61] Cao JY (2013). High voltage asymmetric supercapacitor based on MnO_2_ and graphene electrodes. J. Electroanal. Chem..

[62] El-Kady (2015). Engineering three-dimensional hybrid supercapacitors and microsupercapacitors for high-performance integrated energy storage. Proc. Natl Acad. Sci. USA.

[63] Cai J, Lv C, Watanabe A (2016). Cost-effective fabrication of high-performance flexible all-solid-state carbon micro-supercapacitors by blue-violet laser direct writing and further surface treatment. J. Mater. Chem. A.

[64] Moon GD, Joo JB, Yin YD (2013). Stacked multilayers of alternating reduced graphene oxide and carbon nanotubes for planar supercapacitors. Nanoscale.

[65] Hwang JY (2017). Boosting the capacitance and voltage of aqueous supercapacitors via redox charge contribution from both electrode and electrolyte. Nano Today.

