# Supplementary material for: Laser photonic-reduction stamping for graphene-based micro-supercapacitors ultrafast fabrication
Source: Nat Commun. 2020 Dec 3;11:6185. doi: 10.1038/s41467-020-19985-2 (PMC7712890; doi:10.1038/s41467-020-19985-2)
Supplement: Supplementary file 1 — Supplementary Information. [file 41467_2020_19985_MOESM1_ESM.docx]

**Supplementary Information**

**Laser photonic-reduction stamping for graphene-based micro-supercapacitors ultrafast fabrication**

YongjiuYuan^1,2^, Lan Jiang^1,2🖂^, Xin Li^1,2^, Pei Zuo^1,2^, Chenyang Xu^1,2^, Mengyao Tian^1,2^, Xueqiang Zhang^1,2^, Sumei Wang^1,2^, Bing Lu^3^, Changxiang Shao^3^, Bingquan Zhao^4^, Jiatao Zhang^5^, Liangti Qu^3,6^&Tianhong Cui^7^

^1^Laser Micro/Nano-Fabrication Laboratory, School of Mechanical Engineering, Beijing Institute of Technology, 10081 Beijing, P. R. China.

^2^Beijing Institute of Technology Chongqing Innovation Center, 401120 Chongqing, P. R. China.

^3^Key Laboratory of Cluster Science Ministry of Education of China, School of Chemistry and Chemical Engineering, Beijing Institute of Technology, 102488 Beijing, P. R. China.

^4^Tianjin Navigation Instruments Research Institute, 300131 Tianjin, P. R. China.

^5^Beijing Key Laboratory of Construction-Tailorable Advanced Functional Materials and Green Applications, School of Materials Science & Engineering, Beijing Institute of Technology, 102488 Beijing, P. R. China.

^6^MOE Key Laboratory of Bioorganic Phosphorus Chemistry & Chemical Biology, Department of Chemistry, Tsinghua University, 100084 Beijing, P. R. China.

^7^Department of Mechanical Engineering, University of Minnesota, Minneapolis, MN 55455, USA. ^🖂^Corresponding author E-mail: [jianglan@bit.edu.cn](mailto:jianglan@bit.edu.cn)

**1. Supplementary Discussion**

**1.1 Experiment setup**

Compared with the Gaussian beam, the shaped beam offers substantial advantages in high-precision laser machining^1,2^. We utilized a spatial light modulator (SLM) as a programmable diffractive optical device. An arbitrary intensity distribution beam was then realized by loading a phase hologram onto the SLM. Before adjusting the properties of the beam, we needed to construct an optical path so that the beam coming into the SLM was an ideal Gaussian beam. This ensured it matched our simulated incident light (standard Gaussian beam). A Ti: sapphire laser regenerative amplifier system (Spectra Physics, Spitfire Ace-35F) provided a Gaussian beam (800 nm central wavelength, 35 fs pulse width) that passed through attenuators, two diaphragms, a shutter, and a half-wave plate. The high quality Gaussian beam was reflected by an SLM (Holoeye, Pluto NIR-2, resolution of 1920 pixels × 1080 pixels, diagonal of 0.7 inch) with 10 angles of incidence. The modulated laser beam was then transmitted through a 4f relay system to avoid distortion of the light beam. This relay system consisted of two apertures and two lenses (with focal lengths of 100 and 150 mm, respectively). The sample was mounted on a six-axis translation stage (M840.5DG, PI, Inc.). A CCD camera with a white light source was placed perpendicular to the sample to observe the entire fabrication process.

**1.2 Light field distribution of the shaped beam after SLM**

The purpose of beam shaping is to provide the input and target light fields and then obtain the phase screen. We can therefore build a mathematical model to represent the process of beam shaping.

The amplitude of the input light field of the beam is formulated as

(Supplementary Equation 1)

The corresponding target field oscillating amplitude distribution is formulated as

(Supplementary Equation 2)

The beam shaping principle is as depicted as follows:


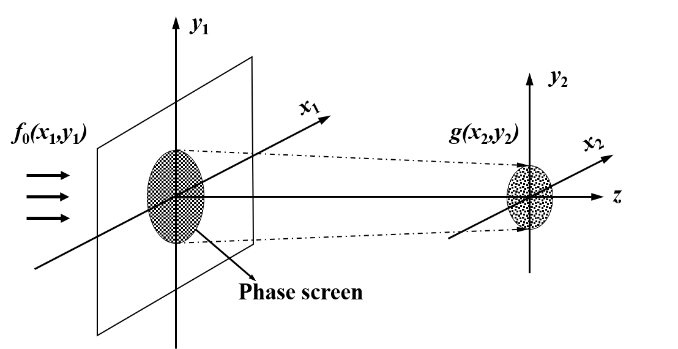


**Supplementary Figure 1: The schematic diagram of the change of input light to output light.** Adding the phase screen to the input light field yields

(Supplementary Equation 3)

The output field obtained after diffraction transmission is as follows:

$$g(x_{2},y_{2})=\frac{\exp(ikr_{01})}{i\lambda z}\int\int_{-\infty}^{\infty} f(x_{1},y_{1})\exp\left\{i\frac{k}{2z}\left[(x_{2}-x_{1})^{2}+(y_{2}-y_{1})^{2}\right]\right\}dx_{1}dy_{1}$$

(Supplementary Equation 4)

We needed to find a suitable phase screen to solve the shaping of the beam. Therefore, when we calculated the beam, we used Fresnel diffraction theory to obtain the intensity distribution at the focusing plane under the paraxial approximation^3^. The laser field at the focus was obtained under this approximation using Fresnel diffraction integration:

$A(x_{f},y_{f},z_{f})=\frac{\exp(ikz_{f})}{i\lambda z_{f}}\iint_{x^{2}+y^{2}<R^{2}} A_{2}(x,y)\exp\left[ik\frac{(x_{f}-x)^{2}+(y_{f}-y)^{2}}{2z_{f}}\right]dxdy$ (Supplementary Equation 5)

where *f* is the focal length of the objective, *k=2π/λ*, *R* is the radius of the objective aperture, and $x_{f},y_{f},z_{f}$are the coordinates on the focusing plane.

**1.3 Optimization of the Gerchberg–Saxton (GS) algorithm**

In the spatial shaping of the femtosecond laser, an ideal optical field pattern can be designed using the algorithm in advance. Then, we can realize the output of the pattern optical field through SLM. To obtain the best machining effect, we optimized the GS algorithm to achieve a high-quality light field to fabricate a high-resolution micro-supercapacitor (MSC) rapidly.

The GS algorithm is an iterative optimization algorithm based on Fourier transform. The algorithm can be used to calculate the phase distribution of the hologram to generate arbitrary light intensity distribution in the mirror. The flow chart of the algorithm is shown below,


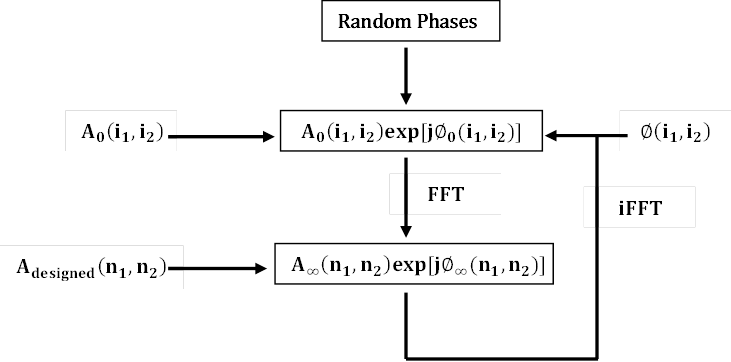


**Supplementary Figure 2: The flow chart of the algorithm.** The incident light field distribution$A_{0}(i_{1},i_{2})$and initial phase were used to obtain the object plane light field$^{\sim}A_{\infty}(i_{1},i_{2})$, and the forward Fourier transform was then used to obtain the light field distribution$^{\sim}A_{\infty}(n_{1},n_{2})$on the image plane. The amplitude value of the design field$A_{design}(n_{1},n_{2})$ was used to replace the calculated amplitude value, and the phase was kept unchanged. Next, we applied inverse Fourier transform and determined the amplitude$^{\sim}A_{01}(i_{1},i_{2})$ and phase distribution of the input plane. The amplitude of the incident light was substituted for the amplitude calculated in the previous step, and the phase was kept unchanged to obtain the object square light field. Finally, the iteration was repeated until the target light field whose amplitude distribution satisfied the requirements was obtained.

On this basis, we dynamically regulate the amplitude of the target light field. Firstly, we calculate the difference between the average amplitude obtained by the Fourier transform and the target amplitude, as$\Delta=A_{\infty}(n_{1},n_{2})-^{\sim}A_{\infty}(n_{1},n_{2})$, then multiply it by the weight factor$\xi$and replace it with the amplitude of the plane light field. We continuously modify the amplitude of the desired target light field to accelerate the iteration process and improve uniformity.


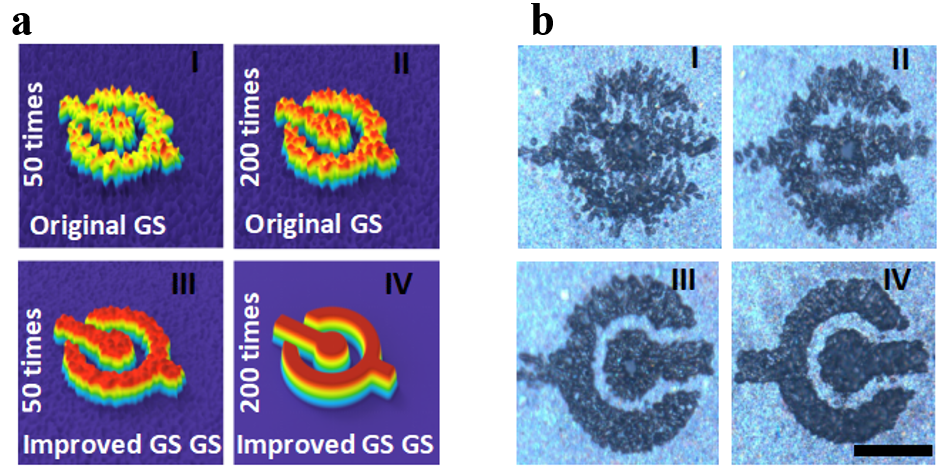


**Supplementary Figure 3: (a) The optical field distributions affected by the algorithm and the number of iterations, (b) The optical micrographs of the different patterns (scale bar, 20 μm).** To obtain a high-quality light field, we set the cycle coefficient such that the number of iterations of the algorithm increased. Figure 3 (a) depicts the optimization process of the algorithm and the influence of the number of iterations on the quality of the distributions at the focal plane. The quality of the output optical field differed considerably between the optimized algorithm and the original algorithm after 50 and 200 iterations. The optimized algorithm had a high utilization ratio of light and uniform distribution of the light field. Increasing the number of iterations resulted in better optical field distribution. Figure 3 (b) illustrates the optical micrographs of the different patterns (I–IV) corresponding to Figure 3 (a) influenced by optimization parameters.

**Supplementary Figure 4: The optical field distribution diagrams optimized by the algorithm.** We conclude that optimizing the GS algorithm results in a superior resolution optical field distribution. Figure 4 depicts the optical field distribution diagram optimized by the algorithm. The spatially shaped femtosecond laser can be used to directly pattern MSCs on the hybrid GO film. Furthermore, such a spatially shaped laser can be used to fabricate high-resolution MSCs in a very short time. The very small narrow gap of the high-resolution MSCs facilitates charge transfer and has excellent electrochemical properties^4^.

All in all, our technology relies on the design of a perfect light field to achieve one-step ultra-fast processing by reshaping the SSFL. And the meaning of optimization of the GS algorithm is to get more uniform laser spots which are more conducive to achieve high-resolution processing of MSCs.

**1.4 Preparation and selection of graphene oxide (GO) hybrid films**

A few layers of GO dispersion at a concentration of 0.5 mg mL^-1^ were mixed with the manganese acetate solution at different ratios. However, because the flocculent material of manganese acetate affects the self-supporting property of the graphene mixed film, we added as much manganese acetate as possible to improve the capacitance characteristics and ensure the excellent mechanical properties of the mixed film. Acetone solution was utilized to easily remove the filter and transfer the graphene hybrid film to different substrates. This film can be stored for dozens of days without wrinkles and has excellent toughness.

**1.5 Fabrication of micro-supercapacitors with a spatially shaped laser**

The laser repetition frequency must be adjusted according to the size of the MSCs to satisfy the machining requirements for the reported experimental equipment and conditions (an X–Y stage with a maximum speed of 2,000 μm s^-1^), otherwise the obtained MSCs overlap. When the scanning speed of the X-axis is 2,000 μm s^-1^, the translation platform can accurately move 2,000 μm per second. A total of 20, 40, and 100 MSCs can be realized on a single path of 2,000 μm in 1 s when the size of the laser spots is 100 × 100, 50 × 50, and 20 × 20 μm^2^, respectively. The laser repetition frequency at this time is also adjusted to 20, 40, and 100 shaped laser subpulses per second. Accordingly, the laser was kept on at all times. We could achieve the highest machining efficiency by adjusting the repetition frequency of the laser pulse and moving the stage as quickly as possible.

**1.6 Electrochemical performance testing and methods**

Because our MSC fabricated by spatially shaped femtosecond laser (SSFL) is only 50×50 μm^2^ in size, high-precision instrumentation is needed for measurement. We therefore built a high-precision probe station equipped with a 5-μm-diameter probe to detect conduction. A confocal imaging system and a three-dimensional translation stage were also used to assemble a device for testing MSCs. The electrochemical performance of our MSCs was measured in a two-electrode system. Cyclic voltammetry (CV), galvanostatic charge-discharge (GCD), and electrochemical impedance spectra measurements were recorded using a computer-controlled electrochemical workstation (CHI 760D). Open-circuit potential (Eocp) measurements were tested for two hours to ensure a stable electrochemical environment (the fluctuation of which was less than 10 mV in the 30 minutes prior to testing).


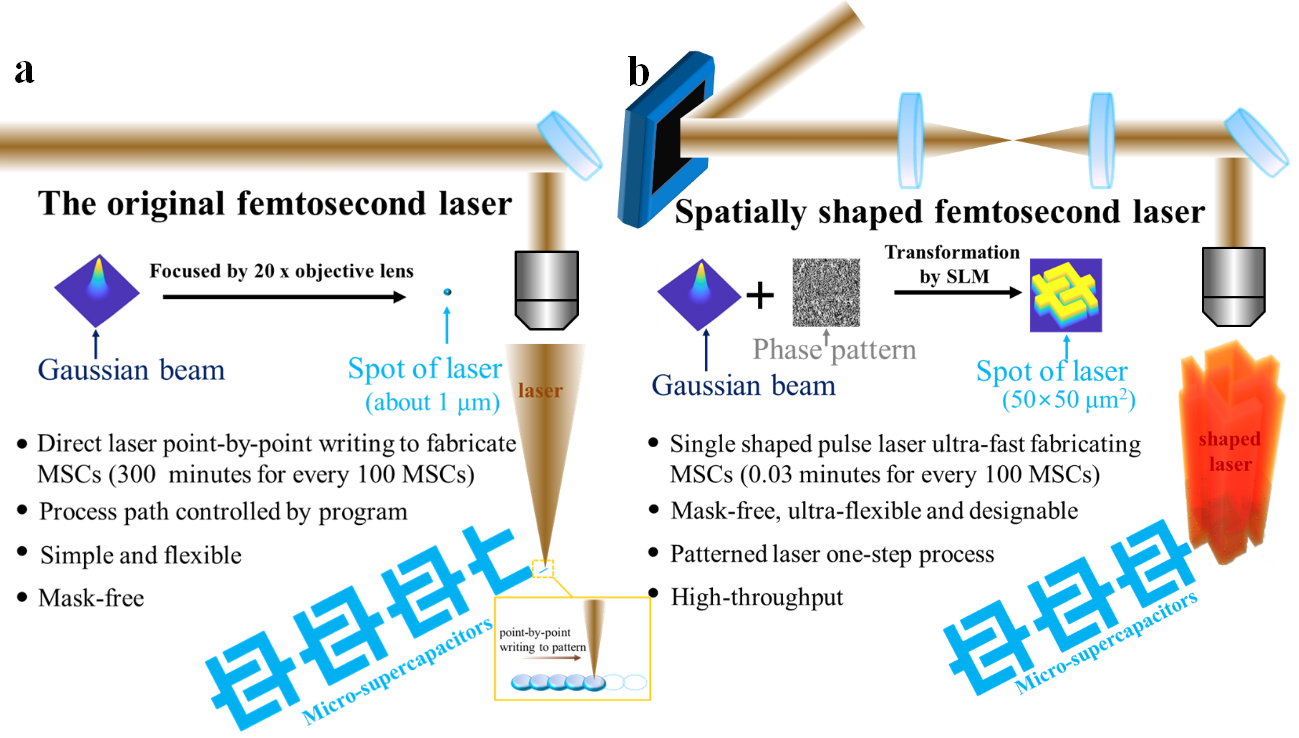


**Supplementary Figure 5: Schematics of the fabrication of MSCs using direct laser writing and** **a** **spatially shaped laser.** Traditional laser point-by-point writing of the focal spot is realized by controlling the movement of the translation stage. Therefore, in actual processing, we consider the stability of the translation stage and repeated positioning accuracy to ensure the consistency of processing; multipatterned rapid processing is difficult to achieve. The incident beams (Gaussian beams) were transformed into arbitrary geometric target beams in SLM by programming phase patterns; the shaped beams were then transmitted on the hybrid GO films. The shaped femtosecond laser can be formed in one step and alters the shape by changing the phase pattern. The spot of the shaped laser is a designable pattern that can directly and instantaneously complete patterned MSCs. In conjunction with the advantages of laser direct writing, this technology greatly improves processing efficiency and achieves consistency for large-area processing. In theory, this process is instantaneous.


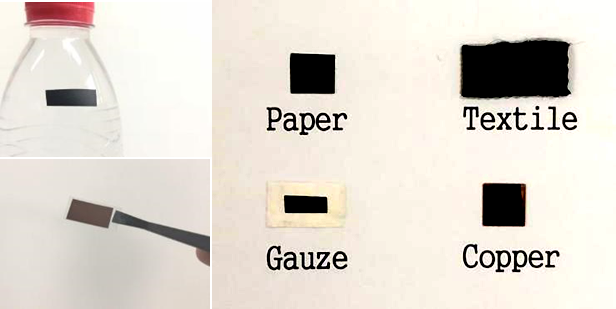


**Supplementary Figure 6**: **Hybrid films can be transferred to bottle, glass, paper, textiles, gauze, and metal substrates.** The hybrid films have good flexibility and mechanical properties; thus, they self-adhere to any substrate after detachment from the filter. This greatly enhances the promotion and application of our MSCs to meet the needs of various conditions.


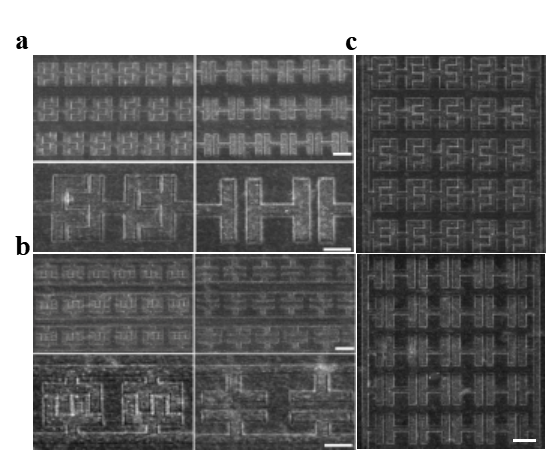


**Supplementary Figure 7: Different light fields and SEM of arrays of MSCs in series and in parallel.** (a) The light fields and SEM images of MSCs in series with different shapes. (b) The light fields and the SEM image of MSCs in parallel with different shapes (scale bars of Figures a and b are 100 and 50 μm, respectively). (c) SEM image of 5 × 5 MSCs arrays with different shapes (scale bar, 100 μm). Only a simple operation was required to process serial and parallel MSCs arrays, and the processing area can be adjusted to meet the needs of microdevices in different sizes.

**Supplementary Figure 8:** **Optical micrographs of MSCs with varying sizes and narrow gaps.** For a clearer display, we observed the shape of MSCs in different sizes. (a–c) (scale bar, 50 μm) We quickly prepared MSCs ranging from 15×15 μm^2^ to 100×100 μm^2^ in size and maintained a regular shape. Because of this flexibility, our technology has potential in the field of micro-nano manufacturing. We also observed circular capacitors with different spacings arranged by the array through the optical microscope image. (d–e) (scale bar, 50 μm) We continuously adjusted the light field distribution to reduce the spacing, thus achieving high-precision manufacturing. (f) An enlarged electron micrograph illustrating the high-precision processing of this technique (scale bar, 50 μm). We used an SLM to quickly convert the target light field for the fast preparation of MSCs of different shapes and sizes. For example, with an array of 3 × 4 MSCs in different sizes, we were able to complete all the processing in 1 minute. This demonstrates that technology can process patterned MSCs with high efficiency and maintain extremely high precision.


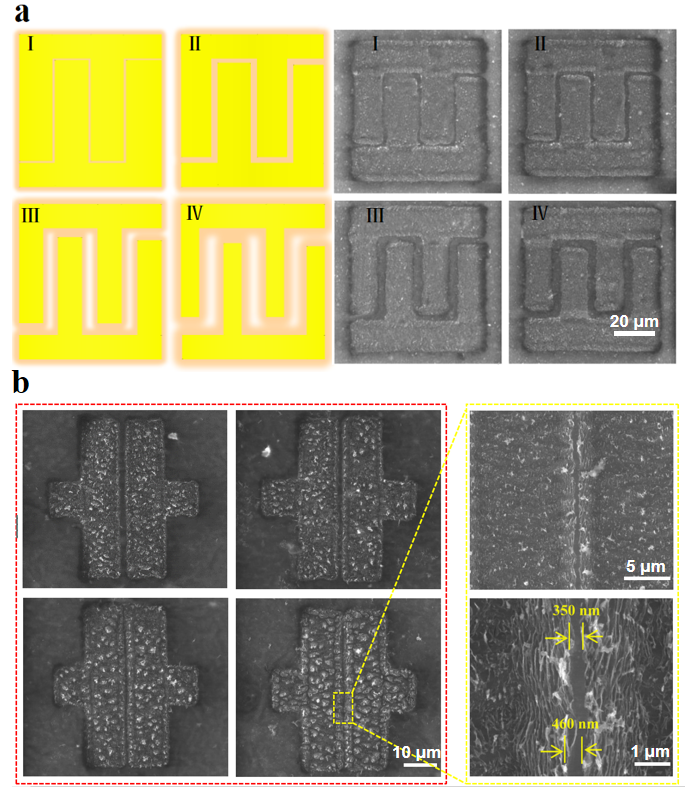


**Supplementary Figure 9: (a) Light field and SEM of interdigital MSCs with different narrow gaps.** **(b) SEM images of MSCs with different narrow gaps and partial magnification of the narrow gap.** We adjusted the shaped laser by controlling the distribution of the light field, thereby achieving high-resolution fabrication. By simulating the distribution, the resulting target light field can be finely regulated. As shown, the resolution of the slit is less than 500 nm, and close to 350 nm in some parts. Our resolution was realized through the pattern design of light field shaping, where the slit between design patterns was used to achieve the highest resolution but was not limited by the laser wavelength.

**Supplementary Figure 10 (a) Nitrogen adsorption-desorption isotherms of the GO/Mn^2+^ hybrid and laser-induced graphene (LIG)/MnO_2_ and the (b) pore size distribution of LIG/MnO_2_.** (a) Type II N_2_ adsorption isotherms were exhibited by LIG/MnO_2_. The BET specific surface area of LIG/MnO_2_ (217.56 m^2^ g^−1^) was approximately eight-times higher than the specific surface area of GO/Mn^2+^ (27.23 m^2^ g^-1^). (b) Numerous mesopores with sizes of <5 nm can be observed, which is consistent with findings of other studies. Most pores had a size of 3.35, 3.70, or 4.05 nm.


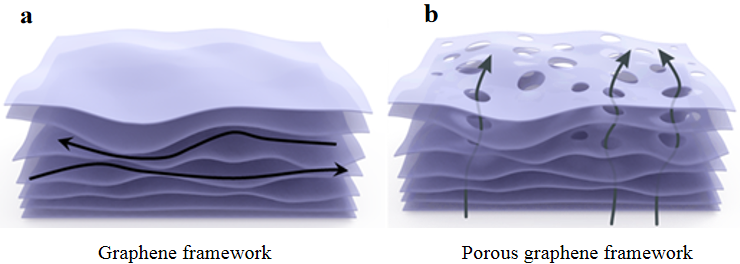


**Supplementary Figure 11: Illustration of a compressed graphene framework (a) and porous graphene framework (b) with arrows depicting the ion transport pathways.** The porous structure provided a faster path and more path choices for the ion transfer. This enabled the ions to contact the electrode material quickly and fully, thus charging and discharging more quickly. This ability to optimize charge transport is shown in Nyquist plots and Bode plots.


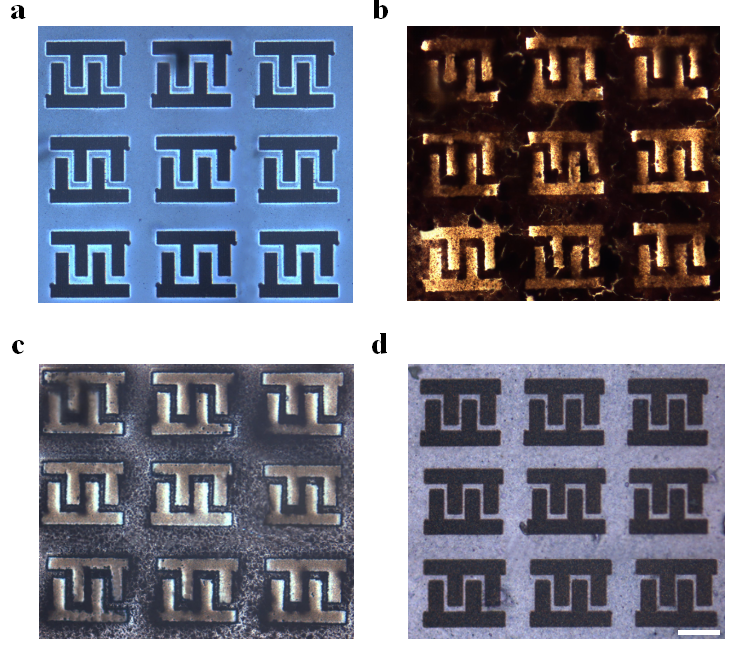


**Supplementary Figure 12:** **Optical micrographs of MSCs of different materials.** To show that SSFL can process ideal patterns in various material systems, we selected several common supercapacitor electrode materials for processing. (a–d) represent PEDOT, metal organic framework, MoS_2_, and Ti_3_C_2_ MXene stamped by SSFL, respectively (scale bar, 50 μm). As shown, the designed electrode patterns were regularly processed.


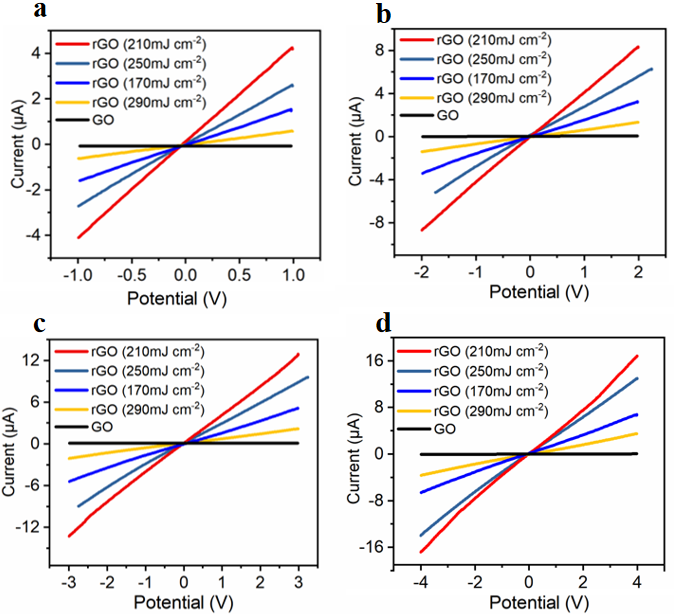


**Supplementary Figure 13: Resistance of the hybrid films** **induced by different laser influences at a potential of 1–4 V (a-d).** To further demonstrate the influence of laser parameters on the processed materials, the laser fluence suitable for processing is explored. The resistance of the material measured under different laser fluences is depicted in the figure.





**Supplementary Figure 14: Conductivity changes as the laser fluence increases.** As the laser fluence increases, the conductivity first increases and then decreases. We therefore divided the laser ablation process into three stages: modified, reduction, and amorphization. The efficiency of photosynthetic LIG/MnO_2_ is most pronounced during the reduction stage. In the modification stage, the material cannot be sufficiently photoreduced. In the amorphization phase, the material is destroyed when the laser fluence is too high. Thus, we obtained the optimal laser fluence needed to improve conductivity.





**Supplementary Figure 15**: **CV curves at 50 mV s^-1^ of MSCs processed by different laser fluences.** To further illustrate the effect of laser fluences on electrochemical performance, we processed the corresponding MSCs based on the previous laser fluences. At the same scan rate, the respective CV curves were measured. We found that the measured area specific capacitance is largest under the laser fluence of 210 mJ cm^-2^, which is consistent with the rules explored earlier.


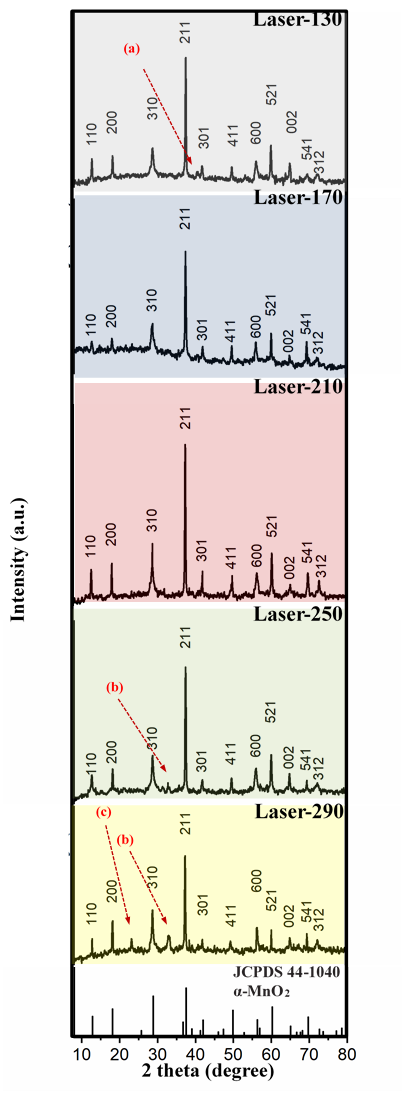


**Supplementary Figure 16: XRD patterns of LIG/Manganese oxides processed under different femtosecond laser fluences.** We defined the composite materials synthesized under different laser fluences as Laser-130, 170, 210, 250 and 290 LIG/Manganese oxide. In the XRD pattern of Laser-130 LIG/Manganese oxide, a peak (a) appeared at 2*θ* = 40.5° as shown in the figure. This corresponds to the expected diffraction peaks of the (200) crystal plane of MnO standard data, following the JCPDS card PDF file no. 74-4748. When the laser fluences are 250 and 290 mJ cm^-2^, peaks (b) and (c) appear at 2*θ* = 33.05° and 23.12°, respectively. These were assigned to the (222) and (211) crystal planes of α-Mn_2_O_3_, following the JCPDS card PDF file no. 41-1442.





**Supplementary Figure 17: Raman spectra of the GO hybrid film processed by different laser fluences.** To further explore the effects of laser fluence on materials, we performed Raman spectroscopy on LIG/MnO_2_ processed under different laser fluences. The high intensity of the G peak demonstrates the high C–C bond ratio, confirming the formation of new graphite domains under four kinds of laser fluence, and the change in oxygen manganese peak at 641 cm^-1^ is regular. When the laser fluence is 210 mJ cm^-2^, the peak value is the most obvious, which indicates that the manganese dioxide content was relatively high under this laser fluence. This may have increased its electrochemical performance.


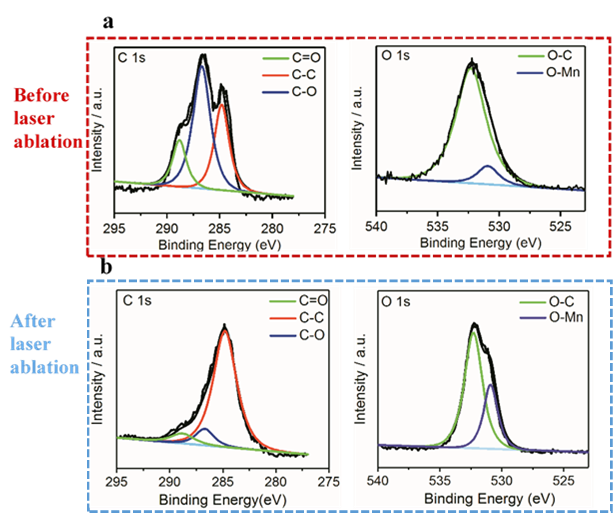


**Supplementary Figure 18:** **Comparison of C 1s and O 1s XPS core level spectra before and after laser ablation.** (a) C 1s and O 1s XPS core level spectra of GO hybrid film before laser ablation. (b) C 1s and O 1s XPS core level spectra of GO/MnO_2_ film after laser ablation. The peaks of C-O (286.7 eV) and C=O (288.8 eV) are consistent with the characteristics of GO. These results fully verified that GO had a large number of oxygen-containing functional groups before laser ablation. After laser ablation, the C–O and C=O bonds of laser-induced graphene (LIG)/MnO_2_ clearly decreased, indicating that some oxygen-containing groups on the GO hybrid film were removed during the reduction. The peaks centered at 530.9 and 532.3 eV also correspond to O-Mn and O-C bonds. Compared with the O-Mn and O-C bonds before laser ablation, the ratio of O-C peak and O-Mn peak is greatly reduced and increase. This indicated that during photosynthesis, the oxygen functional group of GO gradually decreased and formed an oxygen manganese bond, suggesting the formation of manganese dioxide.

**Supplementary Figure 19: XPS spectra of Mn 2p for different laser-LIG/Manganese oxide.** The figure displays the high-resolution XPS spectra of Mn 2p spectra for Laser-130 LIG/Manganese oxide, Laser-170 LIG/Manganese oxide, Laser-210 LIG/Manganese oxide, Laser-250 LIG/Manganese oxide, and Laser-290LIG/Manganese oxide. Because the duration of the ultrashort pulse was smaller than the time scale of most of the physical and chemical characteristics, this resulted in chemical reaction pathways; chemically selective molecular excitation can be effectively controlled by a femtosecond laser. The Mn 2p XPS spectrum exhibited three characteristic peaks at 641.2, 642.5, and 646 eV, corresponding to spin-orbit peaks of manganese with mixed valence states of +3, +4, and +2, respectively. When the fluence of the laser increased to 130 mJ cm^-2^, the percentage of Mn^4+^ in the LIG/Manganese oxides exceeded 50%. The percentage of Mn^3+^ in the LIG/Manganese oxides materials remained high when the percentage of Mn^2+^ was at its lowest. As the fluence of the laser gradually increases, the percentage of manganese in different valence states changes.


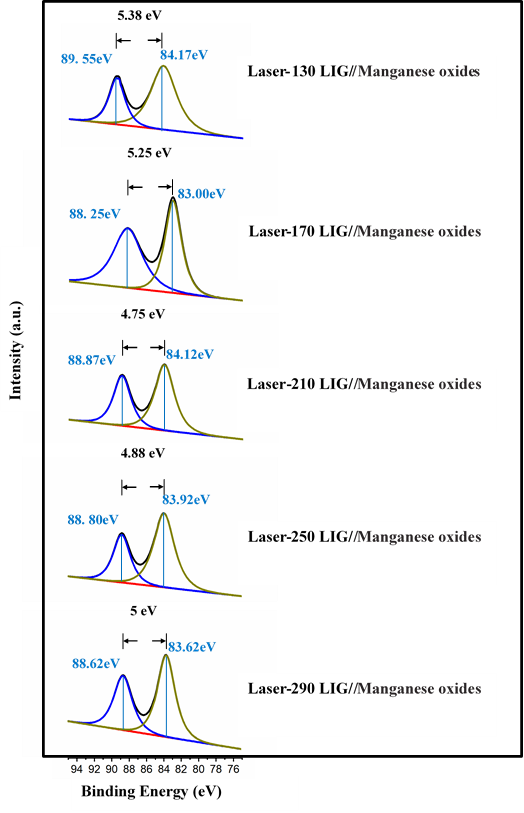


**Supplementary Figure 20: XPS spectra of Mn 3s for Laser-130, 170, 210, 250 and 290 LIG/Manganese oxide.** Mn 3s is more sensitive to the average oxidation state of manganese than Mn 2p is. The energy separation between the two peaks (ΔE) is closely related to the mean manganese oxidation state. The Mn 3s XPS spectra reveal that the ΔE values of a Laser-130 LIG/Manganese oxide, Laser-170 LIG/Manganese oxide, Laser-210 LIG/Manganese oxide, Laser-250 LIG/Manganese oxide, and Laser-290 LIG/Manganese oxide are 5.38, 5.25, 4.75, 4.88, and 5.0 eV, respectively The position of the lower binding energy peak gradually shifts to higher energy as the oxidation number of the sample increases, which is consistent with previous results. The standard ΔE values for MnO_2_, Mn_2_O_3_, and MnO are 4.7, 5.2, and 5.8 eV, respectively. When the laser fluence was 210 mJ cm^-2^, ΔE was 4.75, which is extremely close to the MnO_2_ value. As the laser fluence increases or decreases, the value of ΔE increases, which indicates the presence of manganese oxides of lower-valence states. There are typical defects in the interlayers, such as vacancies and Mn^4+^ substitution by Mn^3+^ and Mn^2+^.


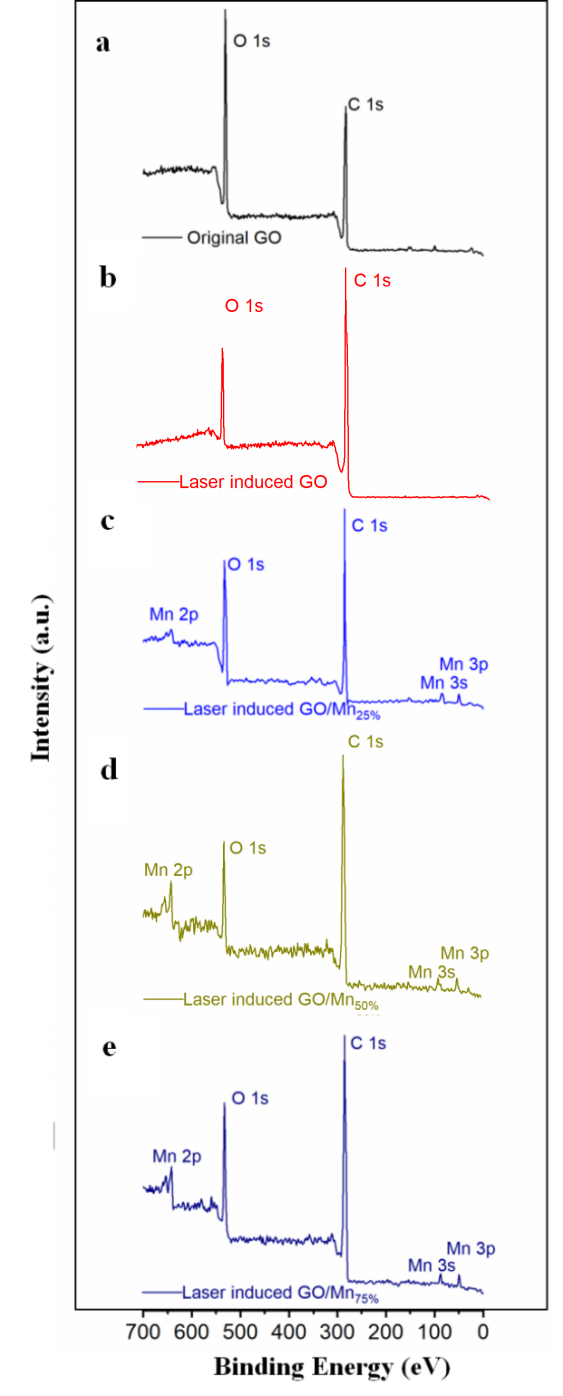


**Supplementary Figure 21 XPS survey spectra for the original GO, laser-induced GO, laser-induced GO/Mn_25%_, laser-induced GO/Mn_50%_, and laser-induced GO/Mn_75%_.** The survey spectrum reveals the presence of C, O, and Mn elements, derived from GO, LIG, and LIG/MnO_2_. As indicated in (a) and (b), both GO and laser-induced GO have signals of carbon and oxygen. After laser reduction, the O1s peak intensity of laser-induced GO is significantly reduced compared with that of GO, demonstrating a loss of oxygen. (c)-(e) The laser-induced GO composite doped with different percentages of Mn^2+^. Manganese ion caused a change in the oxygen and carbon content compared with (a) and (b).


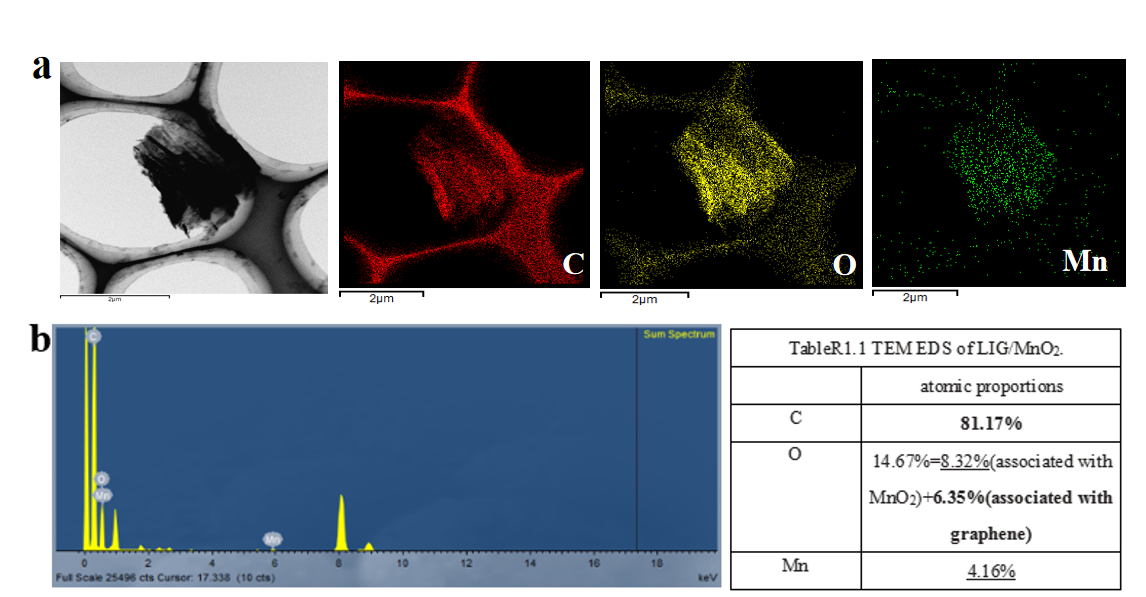


**Supplementary Figure 22: TEM mapping and EDS of LIG/MnO_2_.** This shows the proportion of the three elements (C, O, and Mn). To explain the element distribution and proportion of the composite material more vividly in microscopic terms, we created the surface division of C, O, and Mn. This clearly shows that the sample has the most numerous and densest carbon content distribution. The results of the EDS spectrum are also in line with our predictions. Most of the elements are carbon and the oxygen in GO is largely removed in the form of manganese dioxide. Therefore, the oxygen content also has a certain ratio. The lower manganese content is attributed to the fact that the manganese dioxide produced is scattered on the graphene layer in the form of particles.


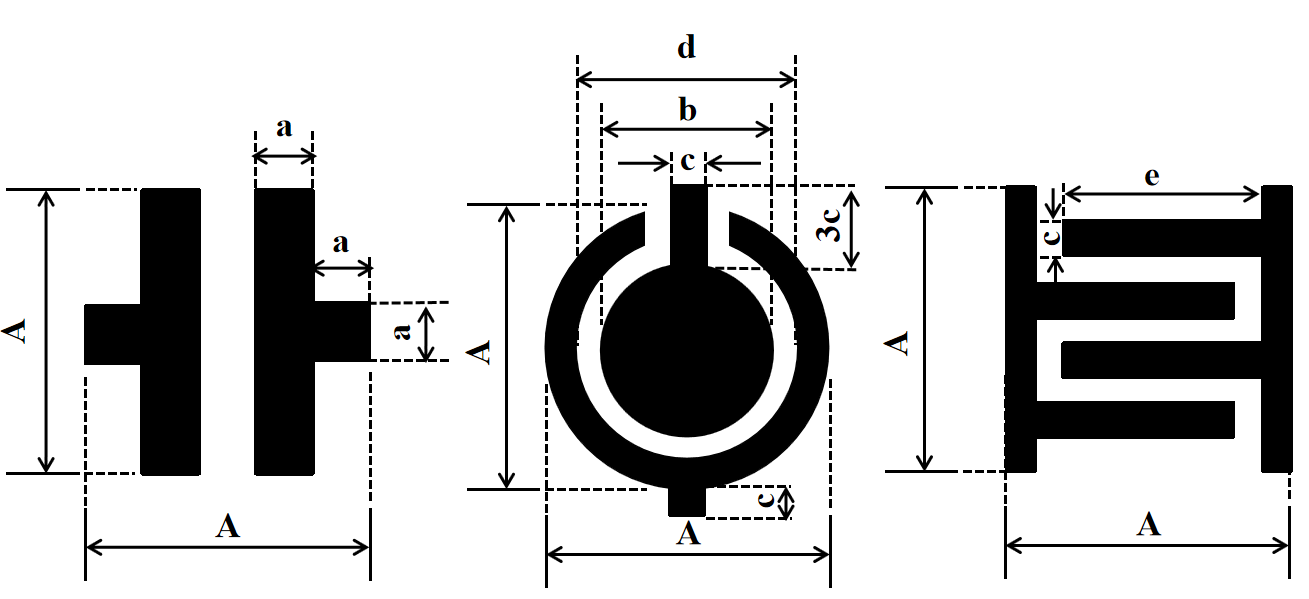


**Supplementary Figure 23: Schematics of various shapes of our MSCs (parallel strip, concentric circle, and interdigital) with size parameters. The values of A, a, b, c, d and e are 50 μm, 10 μm, 32 μm, 5 μm, 45 μm and 30 μm, respectively.** Versatile structural MSCs devices with parallel strip, concentric circle, and interdigital were fabricated with specific size parameters, while keeping a constant geometrical area of the different structures. The same mass loading or area can be verified by simple mathematical calculation:


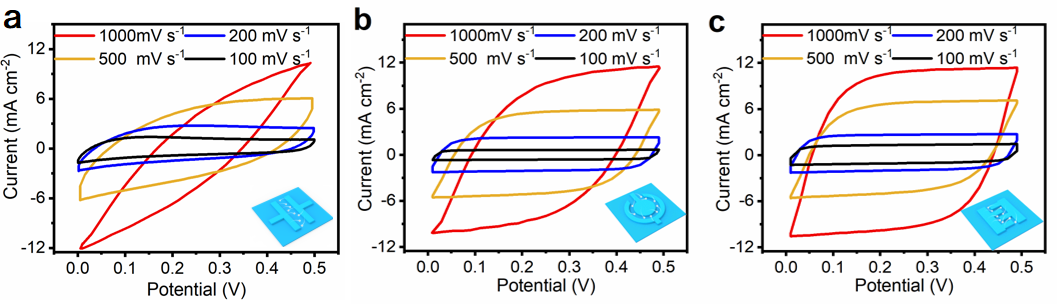


**Supplementary Figure 24: CV curves of (a) parallel strip, (b) concentric circle, and (c) interdigital MSCs at scan rates from 100 to 1000 mV s^-1^ at a voltage of 0.5 V in 0.5 M Na_2_SO_4_.** Measuring the CV curves at high scan rates shows that the three different shapes of MSCs maintain an extremely regular rectangle. This demonstrates that the MSC fabricated by this technique exhibits good rate performance.


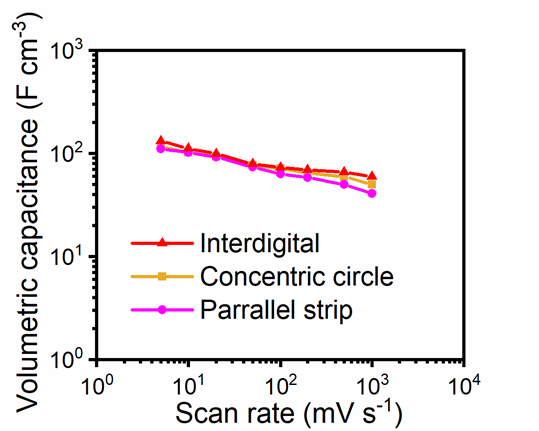


**Supplementary Figure 25: Volumetric capacitance of three different geometries of MSCs at diverse scan rates.** The figure depicts the volumetric capacitance of versatile-shaped MSCs at diverse scan rates. At 131 F cm^-3^, interdigital MSCs have the highest volumetric capacitance compared with the parallel strip (110 F cm^-3^) and concentric circle (116 F cm^-3^) MSCs. We concluded that our MSCs are probably micron-scale and that efficient ion and charge transfer between electrode materials and electrolyte solutions is more important. Interdigital MSCs utilize the electrode material area and volume more efficiently and increase the contact area between the electrode material and electrolyte; due to the short diffusion distance of ions, electrolyte ions are placed in the narrow gap between electrodes. This makes them easy to transport and results in higher performance.


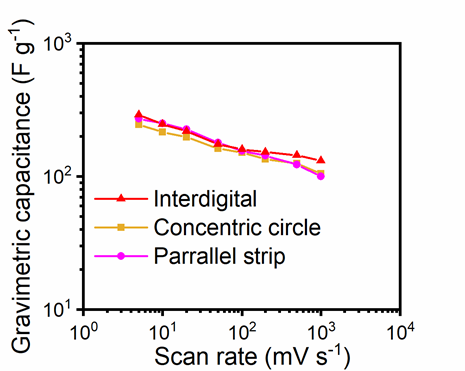


**Supplementary Figure 26:** **Gravimetric capacitance of three different geometries of MSCs at diverse scan rates.** As shown, the interdigital MSC has the highest gravimetric capacitance of up to 290 F g^-1^ at a voltage of 0.5 V. Confounding our expectations, the parallel strip MSCs exhibited excellent gravimetric capacitance (272 F g^-1^), which was even higher than that of the concentric circle MSCs at the same scan rates.





**Supplementary Figure 27: Capacitance retention of the MSCs of different geometries.** We then compared these with the maximum area specific capacitance (at a scanning speed of 50 mV s^-1^). When the scan rates increased 20 fold from 50 to 1000 mV s^-1^, the capacitance retention of interdigital MSCs reached 75.5%; higher than that of both the concentric circle (65%) and parallel strip (55.4%). Thus, interdigital MSCs have the best capacitance retention.


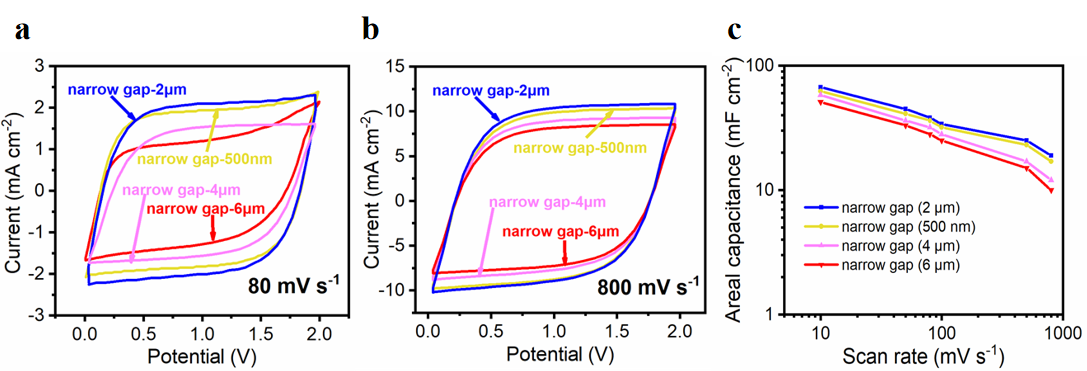


**Supplementary Figure 28: CV characterizations at 80 (a) and 800 mV s^-1^ (b) MSCs with different narrow gaps, (c) Areal capacitance of MSCs with different narrow gaps as a function of scan rates.** Figure a and b were recorded at variable scan rates to evaluate the effect of different microscale interdigital narrow gaps in the MSCs. As shown, the CVs of the high-resolution MSCs maintained a rectangular shape at different scan rates. When the interdigital narrow gaps were 500 nm, 2 μm, 4 μm, and 6 μm, different electrochemical performances are obtained. Of these, the MSCs with a narrow gap of 2 μm exhibited the optimal electrochemical performance, slightly higher than that with a gap of 500 nm and higher than those with gaps of 4 and 6 μm. Our measurements and calculations showed that MSCs with different narrow gaps exhibited differences from areal capacitance under a lower scan rate (Figure c). The highest areal capacitance of 67 mF cm^-2^ was obtained when the narrow gap was 2 μm. Areal capacitances of 62, 58, and 51 mF cm^-2^ were obtained for narrow gaps of 500 nm, 4 μm, and 6 μm, respectively.


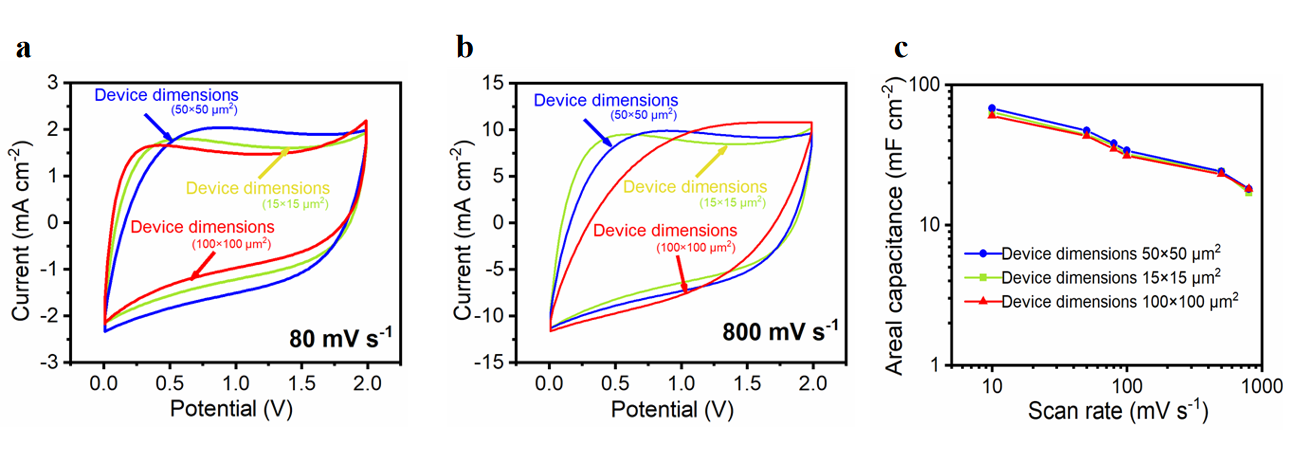


**Supplementary Figure 29： CV characterizations at 80 (a) and 800 mV s^-1^ (b) of MSCs with different device dimensions, (c) Areal capacitance of MSCs with different device dimensions as functions of the scan rate.** We tested three sizes (15 × 15 μm^2^, 50 × 50 μm^2^, 100 × 100 μm^2^) of MSCs. Figure a and b are shown at low and high scan rates for MSCs with different device dimensions. The areal capacitances of different device dimensions were extremely similar at high or low scan rates. An areal capacitance of 38 mF cm^-2^ was achieved at 80 mV s^-1^ when the device dimension was 50 × 50 μm^2^, which was slightly higher than the areal capacitances of 37 and 35 mF cm^-2^ achieved when the device dimensions were 15 × 15 μm^2^ and 100 × 100 μm^2^, respectively. When the scan rate was increased to 800 mV s^-1^, the areal capacitance was almost the same. Figure c shows the effect of device dimensions on electrochemical performance. When the scan rate is low, the dimension has little influence on the performance of the MSC. Thus, when the scan rate is 10 mV s^-1^, the MSC with dimensions of 50×50 μm^2^ has an areal capacitance of 68 mF cm^-2^. Those with dimensions of 15 × 15 μm^2^ and 100 × 100 μm^2^ have areal capacitances of 64 mF cm^-2^ and 60 mF cm^-2^, respectively. Notably, as the scan rate increases, these differences in electrochemical performance disappear.


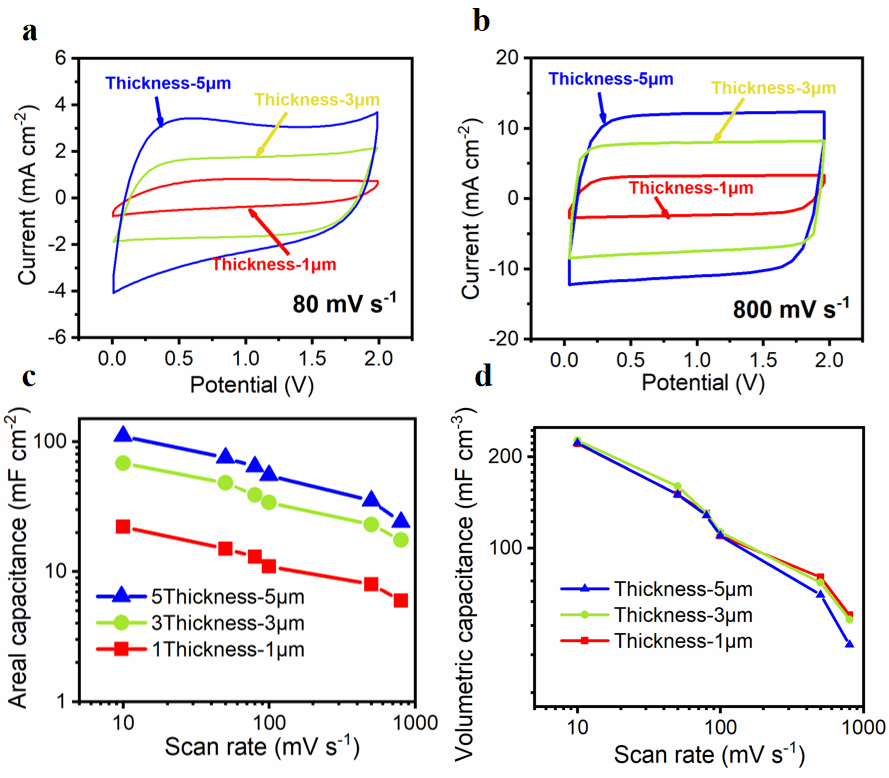


**Supplementary Figure 30：Comparison of CV characterizations at (a) low and (b) high scan rates of MSCs with different thicknesses, as well as (c) areal and (d) volumetric capacitances of MSCs with different thicknesses as functions of the scan rate.** Figure (a) and (b) depict the CV characterizations of MSCs with different thicknesses at 80 mV s^-1^ and 800 mV s^-1^. As expected, the thicker the MSC, the larger the areal capacitance. The areal capacitances of MSCs with thicknesses of 1, 3, and 5 μm were 13, 38, and 64 mF cm^-2^, respectively, at 80 mV s^-1^. As the scan rate increases, the difference in areal capacitance caused by thickness gradually decreases. The MSC with a thickness of 5 μm exhibited the highest areal capacitance. Figure (c) and (d) depict the areal and volumetric capacitances of MSCs with different thicknesses as functions of the scan rate. With respect to areal capacitance, the higher the thickness of the MSC, the higher the capacitance. At a low scan rate, the volumetric capacitance of an MSC with a thickness of 3 μm is slightly higher than that of the other MSCs. At a high scan rate, the MSC with a thickness of 1 μm has the advantage.





**Supplementary Figure 31**: **CV curves of interdigital MSCs at scan rates ranging from 1 to 50 mV s^-1^ at a voltage of 2 V in 0.5 M Na_2_SO_4_.** Although MSCs can maintain a stable shape at high scan rates, we tested CV curves at scan rates of 1 to 50 mV s^-1^ to obtain a larger area specific capacitance and to explore the performance of the MSCs at extremely low scan rates. A symmetrical rectangular shape was obtained at different low scan rates. The areal capacitance and volumetric capacitance of LIG/MnO_2_ MSCs were calculated as 128 mF cm^-2^ and 426 F cm^-3^, respectively.


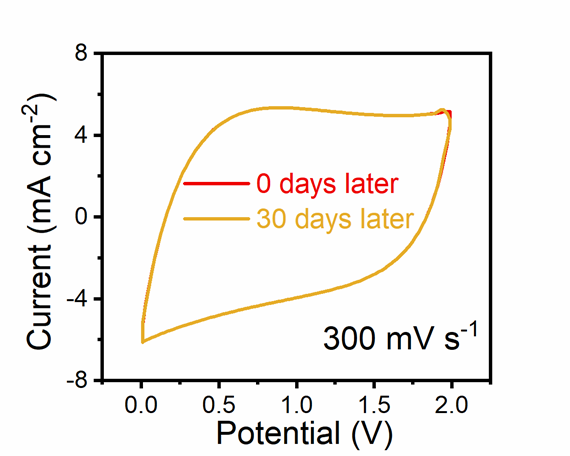


**Supplementary Figure 32:** **CV curves of the fabricated MSCs after 0 and 30 days.** To verify the stability of our MSC, we conducted electrochemical tests on the miniature supercapacitor fabricated after 30 days. We found that it exhibited almost the same electrochemical performance as the initial MSC.


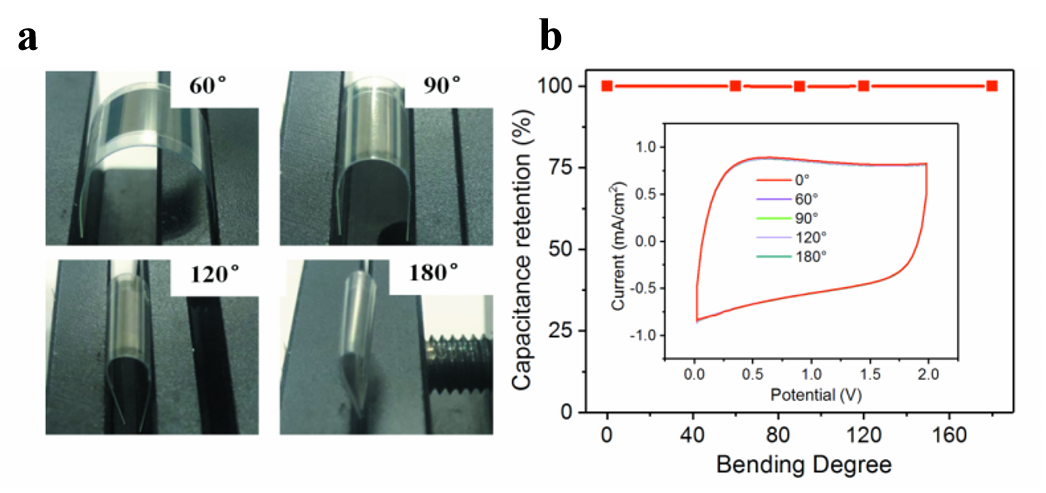


**Supplementary Figure 33**: **Displaying the mechanical flexibility of MSCs.** **(a)** Photographs of MSCs tested in different bending states. **(b)** Capacitance retention of the MSC obtained in different bending states compared with that in the flat state. Inset: CV curves measured in different bending states at 30 mV s^-1^. Contrary to expectations, the CV curves are almost coincident. This outstanding flexibility will expand the use of our MSCs across numerous fields, including integrated circuits, wearable microelectronics, and medical devices.

| **Supplementary Table 1: Comparison of various manufacturing methods in manufacturing efficiency, size，narrow gap and cycle life of fabricated micro-supercapacitors**  **Manufacturing methods**  **Cycle life**  **(capacitance retention)**  **Manufacturing efficiency**  **/30min**  **Size of supercapacitors**  **(narrow gap)** |
| --- |
| Light scribe DVD^5^  0.5×1 cm^2^ (150 μm) 100 10,000 (96%)  3D-Printed stamping^6^ 1×2 cm^2^ (400 μm) 24 10,000 (93.7)  Coating and laser etching^7^  1×1 cm^2^ (500 μm) 180 10,000 (94.3%)  Inkjet-printing^8^ 1×2 cm^2^ (1000 μm) 100 1,000 (98.5%)  Microwave radiation^9^ 1×2 cm^2^ 180 10,000 (95%)  Electrochemical synthesis  /laser irradiation^10^ 5×7 mm^2^ (200 μm) -- 100,000 (100%)  Electrochemical polymerization^11^ 2×2 cm^2^ (150 μm) -- 10,000 (92%)  SSFL strategy (Our work) 50×50 μm ^2^ (0.5 μm) 90,000 12,000 (95%) |

| **Supplementary Table 2.** Summary of the XPS data for the LIG/Manganese oxides induced by different laser fluence | | | |
| --- | --- | --- | --- |
| **Material Mn^4+^2p [eV] Mn^2+^ 2p [eV] Mn^3+^2p [eV]** | | | |
| Laser-130LIG/Manganese oxides | 642.4(53.9%) | 646.0(15.5%) | 641.1(30.6%) |
| Laser-170LIG/Manganese oxides | 642.6(66.4%) | 646.1(23.7%) | 641.2(9.9%) |
| Laser-210LIG/Manganese oxides | 642.5**(79.4%)** | 646.0**(16.6%)** | 641.1**(4.0%)** |
| Laser-250LIG/Manganese oxides | 642.6(69.1%) | 646.2(26.7%) | 641.2(4.2%) |
| Laser-290LIG/Manganese oxides | 642.6(71.4%) | 646.2(24.2%) | 641.2(4.2%) |

**Supplementary Table 2. Characteristic peaks and percentages of Mn^2+^, Mn^3+^ and Mn^4+^.** The Mn 2p spectra of LIG/Manganese oxides photoinduced by lasers with different fluences indicate that the electron BE assigned to Mn^2+^, Mn^3+^ and Mn^4+^ 2p are close to 646.0 eV, 641.2 eV, and 642.5 eV, respectively. The percentages of Mn^4+^ and Mn^3+^ were the highest (79.4%) and lowest (4%) when the laser fluence was 210 mJ cm^-2^. Conversely, the percentage of Mn^2+^ ions decreased from 23.7% to 16.6% and then increased from 16.6% to 26.7% as the laser fluence increased. Altering the laser fluence thus enables various material compositions of LIG/Manganese oxides to be successfully synthesized through photomodulation of the reaction mechanisms. The percentage of manganese in different valence states can be adjusted flexibly.

| **Supplementary Table 3. Atomic concentration table of different samples** | | | | |
| --- | --- | --- | --- | --- |
| Sample | C 1s | O 1s | | Mn 2p |
|  |  | O (associated with graphene) | O (associated with MnO_2_) |  |
| Original GO | 67.38% | 32.62% | **/** | **/** |
| Laser induced GO | 87.28% | 12.72% | / | / |
| Laser induced GO/Mn_25%_ | 80.20% | 7.89% | 7.94% | 3.97% |
| Laser induced GO/Mn_50%_ | 80.33% | 6.08% | 9.06% | 4.53% |
| Laser induced GO/Mn_75%_ | 78.69% | 5.98% | 10.22 | 5.11% |

**Supplementary Table 3. Atomic concentrations of different samples.** In the reduction of GO, the laser and XPS survey scans of GO under optimal conditions demonstrated that the C:O ratio increased from 2.06 (GO) to 6.86 (laser-induced GO), 10.16 (laser-induced GO/Mn_25%_), 13.21 (laser-induced GO/Mn_50%_), and 13.15 (laser-induced GO/Mn_75%_). Therefore, the presence of manganese ions contributed to the increase of the C:O ratio in graphene. This finding indicates a definite improvement of GO reduction in composites that contain manganese ions. This six-fold change represents a marked decrease in the presence of oxygen-containing functional groups at the surface after GO/Mn^2+^ was processed into laser-induced rGO/MnO_2_.

**2. Supplementary References**

1. Qian, D. S. et al. Microstructures induced by excimer laser surface melting of the SiCp/Al metal matrix composite*. Appl. Surf. Sci.* **412,** 436–446 (2017).

2. Xue, Z. W. et al. Actively compensation of low order aberrations by refractive shaping system for high power slab lasers. *Opt. Laser. Technol.* **75, 71–75** (2015).

3. He, F. et al. Fabrication of microfluidic channels with a circular cross section using spatiotemporally focused femtosecond laser pulses. *Opt. Lett.* **35,** 1106-1108 (2010).

4. Anaïs Ferris. et al. Atypical properties of FIB-patterned RuOx nanosupercapacitors. *ACS Energy Lett.* **2,** 1734−1739 (2017).

5. El-Kady, M. F. & Kaner, R. B. Scalable fabrication of high-power graphene micro- supercapacitors for flexible and on-chip energy storage. *Nat. Commun*. **4,** 1475(2013).

6. Zhang, C. F. et al. Stamping of flexible, coplanar MSCs using MXene inks. *Adv. Funct. Mater.* **28,** 1705506 (2018).

7. Liu, Y. Q. et al. Facile fabrication of flexible MSC with high energy density. *Adv. Mater. Technol.* **1,** 1600166 (2016).

8. Liu, Z. Y. et al. Ultraflexible in-plane MSCs by direct printing of solution-processable electrochemically exfoliated graphene. *Adv. Mater.* **28,** 2217−2222 (2016).

9. Jeon, H. et al. Facile and fast microwave-assisted fabrication of activated and porous carbon cloth composites with graphene and MnO_2_ for flexible asymmetric supercapacitors. *Electrochim. Acta.* **280,** 9-16 (2018).

10. Kamboj, N. et al. Ultralong cycle life and outstanding capacitive performance of a 10.8 V metal free micro-supercapacitor with highly conducting and robust laser-irradiated graphene for an integrated storage device. *Energy Environ. Sci*. **12,** 2507-2517 (2019).

11. Purkait, T. et al. Electrochemically customized assembly of a hybrid xerogel material via combined covalent and non-covalent conjugation chemistry: an approach for boosting the cycling performance of pseudocapacitors. *J. Mater. Chem. A*. **8**, 6740-6756 (2020).
