# Supplementary material for: Laser photonic-reduction stamping for graphene-based micro-supercapacitors ultrafast fabrication
Source: Nat Commun. 2020 Dec 3;11:6185. doi: 10.1038/s41467-020-19985-2 (PMC7712890; doi:10.1038/s41467-020-19985-2)
Supplement: Supplementary file 3 — Description of Additional Supplementary Files [file 41467_2020_19985_MOESM3_ESM.pdf]

Description of Additional Supplementary Files

1. File Name: Supplementary Movie 1

   Description: To present the processing more clearly, the video is played at 20% speed. The video shows the machining of the series of micro-supercapacitors in the longitudinal direction.

2. File Name: Supplementary Movie 2

   Description: This video is played at 50% speed, and it shows the horizontal fabrication of micro-supercapacitors.
