# Supplementary material for: Laser photonic-reduction stamping for graphene-based micro-supercapacitors ultrafast fabrication
Source: Nat Commun. 2020 Dec 3;11:6185. doi: 10.1038/s41467-020-19985-2 (PMC7712890; doi:10.1038/s41467-020-19985-2)
Supplement: Supplementary file 2 — Peer Review File [file 41467_2020_19985_MOESM2_ESM.pdf]

Reviewers' comments:

Reviewer #1 (Remarks to the Author):

this work reported a reduced graphene oxide/MnO_2 micro supercapacitors fabricated by a spatially shaped ultrafast laser. The authors declare that more than 30,000 capacitors can be "stamped" within 10 min and a single capacitor displays the energy density of 0.23 Wh/cm3, outstanding specific capacitance of 128 mFcm-2/426.7 Fcm3. I found that the manuscript is presented in a very unclear mode with wrong descriptions and lots of parts are misled. These mistakes limit the novelty this work could display. This manuscript should be rejected or at least rewritten/resubmitted for new review by elucidating at least the following issues:
1. Physics and chemistry of GO reduction and MnO_2 for enhanced capacitance is well investigated. This work does not present any new knowledge on these fields. The laser reduction (so-called stamping in this manuscript) through a holographic mirror (SLM) in Figs 1&2 are not presented in a proper way. Fig. 2 (c-f) are basically conducted by a patterned mirror. The simultaneous reduction from the patterned beam is effective and valued. But the authors failed to explain why they need 10 min. This indeed unveil if the authors properly explain the laser reduction mechanism, either the same as or different from the well understanding results (photothermal, or non-thermal mechanism). Fig. 3s display a lateral resolution is 500 nm, slightly bigger than half of wavelength (800 nm). This indicates a limited thermal reduction by short pulses. Unfortunately, this manuscript does not unveil this physics. It is fine to focus on manufacturing novelty (if there is any in this work). For a holographic mirror, it is critical to explain the mechanism to complete a 3D reduction without z-scan as a conventional 3D stage. In another word, the authors should present the thickness if they electrically create a flat mirror in SLM. Again unfortunately, in fig. 3 (a, b), authors do not display either the thickness resolution or total thickness they create. This is very misled. The thickness resolution can tell how thin they can write with a holographic mirror and the total thickness directly relates to the intrinsic advantage of a holographic mirror. I do not need guess or deduce which values they should be. But without these, it is hard to know if their calculation of energy density/specific capacitance are correct. Even worse, these formulae provided in the main text and the supplementary materials do not automatically explain how a holographic mirror complete a z-scan. Therefore, Fig. 1 are totally misled. The "stamp" in the title is also misled since the physics is still photonic reduction. A minor mistake is that the authors have to document how the gas releases from the reduced body. If they are trapped, a porous structure will be formed and how this porosity affects the energy storage performance.
2. Fig. 4 (a) displays that their capacitor is pretty worse even at 1V/s, a pretty low scanning rate. The unit of Fig. 4 (h) is ohm/cm2. Without the thickness a unit in ohm is better to display the internal resistance.
3. Fig. 5 (a) is not properly presented. The discharge time of only 60 s at a 4 mA/cm2 is alos display the capacitance is very limited.
4. There is nothing new from Raman/XPS/TEM in Fig. 3.

Reviewer #2 (Remarks to the Author):

Yuan et al. have developed a femtosecond laser stamp method to fabricate rGO/MnO_2 based micro-supercapacitors. It's fascinating that this work demonstrates an ultrafast method to reduce graphene oxide and produce patterned micro-supercapacitors in a very short time. However, this work still needs a lot of improvement before its publication in a high-level scientific journal such as Nature Communications. For such a high profile journal, the current discussion and data presentation need significant improvement. Here are some detailed comments:
1. The authors should provide more discussion about the reason for reshaping the ss-fs laser and the meaning of optimization of the GS (Gerchberg–Saxton?) algorithm. What's the relationship to achieve "ultra-fast patterning of laser induced graphene/MnO_2 electrodes"? Is it to get better resolution or faster pattern speed? More discussion and experimental results should be provided to support the

calculation at the beginning of the discussion part.
2. What's the spot size of the femtosecond laser used in this research work? The authors claimed that the SFLS system could achieve 500 nm resolution. However, based on the SEM images in Supplementary Figure 2, the laser induced patterns exhibited very rough edges even with an electrode gap of about 3-8 μm. The pattern demonstrated in Supplementary Figure 3, with a claimed "500 nm" narrow gap, showed random curved gap channels. The evidence to prove that the high resolution of "500 nm" has been achieved is insufficient.
3. The physicochemical characterization of the laser induced LIG/MnO2 electrode needs further improvement. XRD characterization should be supplemented to further study the crystalline structure of the laser induced Mn-based compound(s). Although the Raman and O 1s XPS data demonstrate the presence of Mn-O covalent bonds, it's still insufficient to prove the preparation of MnO2. In addition, the Mn 2p XPS data also show a large percentage of Mn3+ and Mn2+ in the total Mn amount. Thus, the structure of manganese in the final laser induced composite appears to be more complicated than pure MnO2.
4. Although the laser induced fabrication of metal oxides is not new, the authors should provide more details about the mechanism(s) related to the transition from Mn2+ to MnO2.
5. The conductivity of 3.2 S m-1 of the laser induced graphene is moderate. However, for the micro-supercapacitor, the measured time constant of 10.6 μs, is extremely short time. The author should provide more explanation about how such a fast time constant is possible with such relatively poor conductivity.
6. More details about the dimensional parameters, such as the interdigital gap width and the electrode and device dimensions, should be provided in the device electrochemical performance discussion. These parameters can critically affect electrochemical performance.
7. Either the "2 nm" scale bar in Figure 3 n and o or the indicated lattice spacings (0.29 nm and 0.33 nm) in these two TEM images are incorrect.
8. The English in this manuscript, especially the materials characterization discussion and electrochemical performance evaluation could be further improved by a professional English polishing service team. There are still some typos that need further revision before submission to another journal, such as "surfacearea" on page 2.

Reviewer #3 (Remarks to the Author):

This paper deals with laser-stamping mcrosupercapacitor technology and its manufacturing. The authors have used the femtosecond laser source for patterning the electrodes. Although this a new finding in the microsupercapacitor field by femtosecond laser, however, several issues are still there as a matured technology. A few comments are given as appended below.

I have a crucial comment on the title of this paper. What does ultrafast signify?
Is it a very fast method or the authors used ultrafast laser sources? Clarify.

If it is the very fast production of the device, then one has to justify with literature and explain with the statement '1 cm2 within 10 minutes', which is not a fast procedure.

Several optimization parameters are missing. e.g. spot size and speed of the laser or the film quality and how it is changing during that optimization.

Manufacturing technology is not the proper phrase for this manuscript. To do this several large scale optimization required at different batch.

In my opinion, this work is actually narrowing down the device geometry and area rather focusing the bulk scale production. In this respect, I am curious to know about the gravimetric capacitance and volumetric capacitance, which an Industry may look into it.

In Fig. 4, authors have demonstrated shape versatility of MSC devices. I doubt all the devices that have the same geometrical are or not. Although they have normalized it with the area, however different areas will contribute to different mass loading of the devices. Therefore it is recommended to keep a similar area of the devices and compare the gravimetric capacitance as well during the optimization of shape versatility.

When MSC manufacturing is focusing on the manuscript, one should check the stability of the devices with respect to cycling performances.

I can easily found that several other works with better performances for laser irradiated graphene in the literature, however, authors didn't consider them for comparison with their data.

Point-by-Point Responses to Reviewers' Comments

Manuscript ID: NCOMMS-20-02591-T

Title: Ultrafast laser-stamping for MSCs manufacturing

Authors: YongjiuYuan, Lan Jiang, Xin Li, Pei Zuo, Chenyang Xu, Mengyao Tian, Xueqiang Zhang, Sumei Wang, Bing Lu, Changxiang Shao, Bingquan Zhao, Jiatao Zhang, Liangti Qu and Tianhong Cui

We are indebted to you and the reviewers for the time and efforts in reviewing our paper. The detailed comments from the referees are very impressive and greatly valued. We are happy to have such excellent advice for improving the paper's quality. With our best efforts and best knowledge, we have made changes by following the referees' comments. Our attached report summarizes our understanding of the recommendations in response. According to the suggestions of reviewers, we have rewritten the problematic parts to get a clear expression, and modified the description of the article. You can see that our manuscript has undergone a large number of revisions, as well as other relevant experiments and literature research in response to the comments. The reviewers' comments are reproduced following by the corresponding modifications to the manuscript.

In our response, the reviewers' comments were divided into several sections. Each section consists of three parts: reviewers' comments, our response, and modifications. The references were coded independently for each section. For example, the first reference in Section 1 was coded as R1.1; the first figure in Section 1 was named as Figure R1.1；the first equation in Section 1 was named as E1-1. Throughout this document, the following conventions are used:

- ◊ *Black text in Italics = The reviewers' comments.*
- ◊ Black text = The author's response.
- ◊ Red text = Quotations from the manuscript.
- ◊ Blue text = New additions to the manuscript.

1. REVIEWER NO. 1

1.0 Reviewer's Comments

"This work reported a reduced graphene oxide/MnO_2 micro supercapacitors fabricated by a spatially shaped ultrafast laser. The authors declare that more than 30,000 capacitors can be "stamped" within 10 min and a single capacitor displays the energy density of 0.23 Wh/cm^3, outstanding specific capacitance of 128 mF cm^{-2}/426.7 F cm^3. I found that the manuscript is presented in a very unclear mode with wrong descriptions and lots of parts are misled. These mistakes limit the novelty this work could display. This manuscript should be rejected or at least rewritten/resubmitted for new review by elucidating at least the following issues:

Our Response

Thank you for your detailed and invaluable feedback and for your time and effort in reviewing our manuscript. Your constructive feedback has helped us revise our manuscript, which has enabled us to improve the quality of our manuscript.

Per your comments, we have revised the instances of unclear and incorrect phrasing in our manuscript. Therefore, starting from the title and abstract, we have rearranged the manuscript and extensively modified and rewritten it to highlight the innovative points of our study in our responses from 1.1 to 1.4.

1.1 Reviewer's Comments

1.1 Physics and chemistry of GO reduction and MnO_2 for enhanced capacitance is well investigated. This work does not present any new knowledge on these fields. The laser reduction (so-called stamping in this manuscript) through a holographic mirror (SLM) in Figs 1&2 are not presented in a proper way. Fig. 2 (c-f) are basically conducted by a patterned mirror. The simultaneous reduction from the patterned beam is effective and valued. But the authors failed to explain why they need 10 min. This indeed unveil if the authors properly explain the laser reduction mechanism, either the same as or different from the well understanding results

(photothermal, or non-thermal mechanism). Fig. 3s display a lateral resolution is 500 nm, slightly bigger than half of wavelength (800 nm). This indicates a limited thermal reduction by short pulses. Unfortunately, this manuscript does not unveil this physics. It is fine to focus on manufacturing novelty (if there is any in this work). For a holographic mirror, it is critical to explain the mechanism to complete a 3D reduction without z-scan as a conventional 3D stage. In another word, the authors should present the thickness if they electrically create a flat mirror in SLM. Again unfortunately, in fig. 3 (a, b), authors do not display either the thickness resolution or total thickness they create. This is very misled. The thickness resolution can tell how thin they can write with a holographic mirror and the total thickness directly relates to the intrinsic advantage of a holographic mirror. I do not need guess or deduce which values they should be. But without these, it is hard to know if their calculation of energy density/specific capacitance are correct. Even worse, these formulae provided in the main text and the supplementary materials do not automatically explain how a holographic mirror complete a z-scan. Therefore, Fig. 1 are totally misled. The "stamp" in the title is also misled since the physics is still photonic reduction. A minor mistake is that the authors have to document how the gas releases from the reduced body. If they are trapped, a porous structure will be formed and how this porosity affects the energy storage performance.

Our Response

Thank you for your constructive suggestions, which have helped us improve the quality of our manuscript. Because this problem involves several aspects, we shall segregate this question into several parts to supplement our response and appropriate revisions.

1.1.1 Reviewer's Comments

1.1.1 "Physics and chemistry of GO reduction and MnO_2 *for enhanced capacitance is well investigated. This work does not present any new knowledge on these fields."*

Our Response

First, in this study, we achieved photochemical synthesis of graphene and manganese dioxide by using a spatially shaped femtosecond laser (SSFL) for micro-supercapacitors (MSCs), with a three-dimensional (3D) porous composite structure constructed in only one step. This is indeed a novel method of fabricating laser-induced graphene (LIG)/MnO_2 MSCs. The photoinduced reaction mechanism of graphene oxide (GO) and Mn^{2+} is different from the traditional reduction mechanism. The obtained 3D porous composite material is more conducive to improved capacitance.

(1) Technological superiority in the fabrication of LIG/MnO_2 composite materials

Graphene/manganese dioxide composites can improve the capacitive performance of supercapacitors. To ensure the combination of the two materials is highly robust, GO and manganese salt precursors are often used in combination, and redox reactions are used to dope graphene and manganese dioxide. The traditional method involves the use of strong oxidants to promote reactions leading to the synthesis of LIG/manganese dioxide. For example, when using the strong oxidizer potassium permanganate[R1.1-3], the reaction can be described as follows:

$$4KMnO_4+3C+2H_2SO_4 \rightarrow 4MnO_2+2K_2SO_4+3CO_2+2H_2O \qquad \text{(E1-1)}$$

The C atoms of LIG can convert Mn^{7+} into Mn^{4+}, leading to the initial formation of MnO_2 nanostructures on LIG, which, in turn, facilitate further growth[R1.4]. GO undergoes thermal reduction, leading to the formation of LIG. However, it is difficult to precisely regulate the reduction process and meet the needs of microdevice fabrication. Additionally, this method of chemical reduction often requires the reaction process to be conducted using an extra current collector and relies on the current collector for supercapacitor assembly. Chen et al[R1.5] reported a composite of GO and MnO_2 fabricated on a nickel foam current collector with high capacitance through the interaction between GO and MnO_2, which is possible because the oxygen

functional groups of GO act as anchor sites for MnO_2 growth. In other studies[R1.6,7], graphene/MnO_2 composites have been fabricated through spinning or rolling, which require the construction of a current collector or stripping of the gap through other processes.

However, to construct better structures and thus enhance the performance of supercapacitors, studies have reported 3D heterogeneous structures. He et al[R1.8] reported 3D graphene/MnO_2 MSCs with high areal capacitance. A specially treated Ni foam template can play the role of the skeleton for graphene grown through chemical vapor deposition, aiding construction of a highly porous graphene network for a high loading mass of electrodeposited MnO_2. Fei et al[R1.9] used a metal foam as a 3D skeleton, performed hydrothermal growth of manganese dioxide nanoparticles on the metal foam, and used plasma-enhanced chemical vapor precipitation to produce graphene quantum dot/MnO_2 heterostructural materials.

By contrast, we used a novel SSFL method to fabricate LIG/MnO_2 MSCs. In this study, MSCs were rapidly fabricated using a SSFL in one step in situ. The mixed sample underwent simultaneous oxidation of Mn^{2+} to MnO_2 and reduction of GO to 3D laser induced graphene (LIG). LIG-wrapped MnO_2 was thus successfully synthesized. During this process, oxidants, current collectors, and metal foam for the 3D structure were not required. More importantly, a femtosecond laser was employed to control the chemical reaction process to synthesize composites.

(2) Photochemical regulation of the LIG/MnO_2 synthesis mechanism

Femtosecond lasers have ultrahigh peak power ($>10^{13}$ W cm^{-2}) and ultrashort irradiation periods, and femtosecond laser fabrication has the unique advantage of nonlinear nonequilibrium processing[R1.10-12]. When light and matter interact, at such high peak intensity, seed electrons are primarily generated through strong electric field ionization (multiphoton ionization and tunneling ionization) and are unrelated to the initial state of the material. Ultrafast localized nonlinear absorption leads to spatial confinement of radiation-induced material changes in a focal volume[R1.13].

Moreover, the ultrashort pulse is shorter than most of the relevant physical and chemical characteristic time scales, resulting in chemical reaction pathways and

chemically selective molecular excitation that can be effectively controlled using the femtosecond laser. On adjusting the laser fluence to control photoinduced or photothermally induced reduction and oxidation, the valence state of manganese oxide can be well regulated.

Hence, femtosecond laser fabrication is certain and repeatable. Furthermore, the nonequilibrium effects of the femtosecond laser potentially facilitate photon–electron coupling before the lattice is altered. Electrons are excited from the bonding state to the antibonding state, weakening C–O electron bonding near the top of the valence band, resulting in direct removal of oxygen atoms. However, as the number of femtosecond laser pulses increases, the thermal effect becomes even stronger, resulting in photothermal lysis of the carbon bond structure and an increase in the number of lattice defects[R1.14]. The intensity of femtosecond laser pulses provides adequate kinetic energy to oxygen atoms such that they exit the graphene sheet in gaseous form without imparting large kinetic energy to carbon atoms[R1.15].

We propose an interesting photosynthetic route for synthesizing LIG–MnO_2 in Figure R1.1.

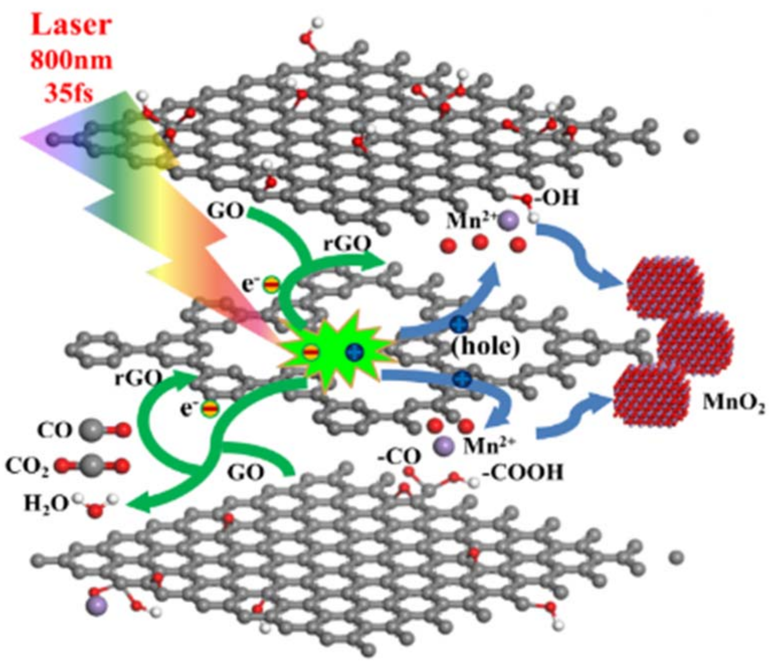

Figure R1.1 Schematic of the resultant morphology change and phase transformation by a femtosecond laser under the proposed photosynthetic routes

Femtosecond lasers provide numerous photons that excite electrons to create free-moving electrons and holes. Through their action, GO reduced to LIG and Mn^{2+} was absorbed on the negatively charged region of the hydrophilic oxygen functional groups of GO owing to electrostatic effects. Before laser processing, a mixture of GO and manganese acetate solutions was completely degraded ultrasonically. GO-wrapped Mn^{2+} particles were thus obtained. Mn^{2+} facilitates GO reduction because of its higher oxidation potential[R1.16,17], and Mn^{2} uses anchor sites in GO for oxidation to MnO_2 nanoparticles in situ[R1.18]. Simultaneously, GO deoxygenation releases energy, which functions as an in situ power source driving the new oxidation reaction of Mn^{2+} [R1.19].

Thermal GO deoxygenation is initiated by a small amount of heat, and as the process continues, both temperature and energy increase, corresponding to the energy released during the reduction of GO and sufficient to propagate and sustain GO reduction[R1.20]. The amount of heat released during the GO deoxygenation reaction is several times that required to drive the endothermic oxidation of metal ions[R1.19,21].

The equations for the reaction are as follows:

$$GO+h\Upsilon \rightarrow rGO\ (h^{+}+e^{-}) \qquad (E1\text{-}2)$$

$$Mn^{2+} \rightarrow Mn^{4+}+2e^{-} \qquad (E1\text{-}3)$$

$$(GO)\text{-}OH+(GO)\text{-}COOH+Mn^{2+}+e^{-} \rightarrow rGO+MnO_2+CO_2\uparrow+H_2O \qquad (E1\text{-}4)$$

$$(GO)\text{-}O\text{-}(GO)+(GO)\text{-}COOH+Mn^{2+}+e^{-} \rightarrow rGO+MnO_2+CO\uparrow \qquad (E1\text{-}5)$$

As the reaction continues, photothermal reduction begins to occur, and the cumulative effect of the pulsed laser results in heat generation and thus the pyrolysis of GO, wherein oxygen-containing functional groups including hydroxyls (-OH), carboxyls (-COOH), and oxygen bridges (C–O–C) are lysed into CO, CO_2, and H_2O, which can be eliminated. Upon GO lysis, the energy and accumulated heat in the reaction provide the original energy for oxidation to the manganese ions, thus

completing the formation of manganese oxide.

$$GO+Mn^{2+} \xrightarrow{\Delta} rGO+MnO_2+CO_2\uparrow+CO\uparrow+H_2O\uparrow \qquad (E1\text{-}6)$$

Thus, the femtosecond laser reduces GO, simultaneously facilitating the action of metal ions, which is a novel mechanism of action. Consequently, GO reduction and MnO_2 synthesis are mutually reinforcing processes in shaped femtosecond laser radiation. By altering the pulse energy, materials of various LIG/manganese oxide compositions were successfully synthesized through photomodulation of reaction mechanisms. By using an SSFL, the Mn^{4+} percentage could be maximized, and MnO_2 is the primary component of LIG/manganese oxide.

To demonstrate that femtosecond laser irradiation can be used to control the synthesis of manganese oxide, we performed XPS measurements to investigate the manganese oxide and graphene composites. We defined the composite materials synthesized under different laser fluences as Laser-130, 170, 210, 250 and 290 LIG/Manganese oxide, corresponding to laser fluences of 130, 170, 210, 250, and 290 mJ/cm^2, respectively.

Figure R1.2 displays the high-resolution XPS spectra of Mn 2p spectra for Laser-130, 170, 210, 250 and 290 LIG/Manganese oxide. Because the duration of the ultrashort pulse was smaller than the time scale of most of the physical and chemical characteristics, this resulted in chemical reaction pathways; chemically selective molecular excitation can be effectively controlled by a femtosecond laser. The Mn 2p XPS spectrum exhibited three characteristic peaks at 641.2, 642.5, and 646 eV, corresponding to spin–orbit peaks of manganese with mixed valence states of +3, +4, and +2, respectively.

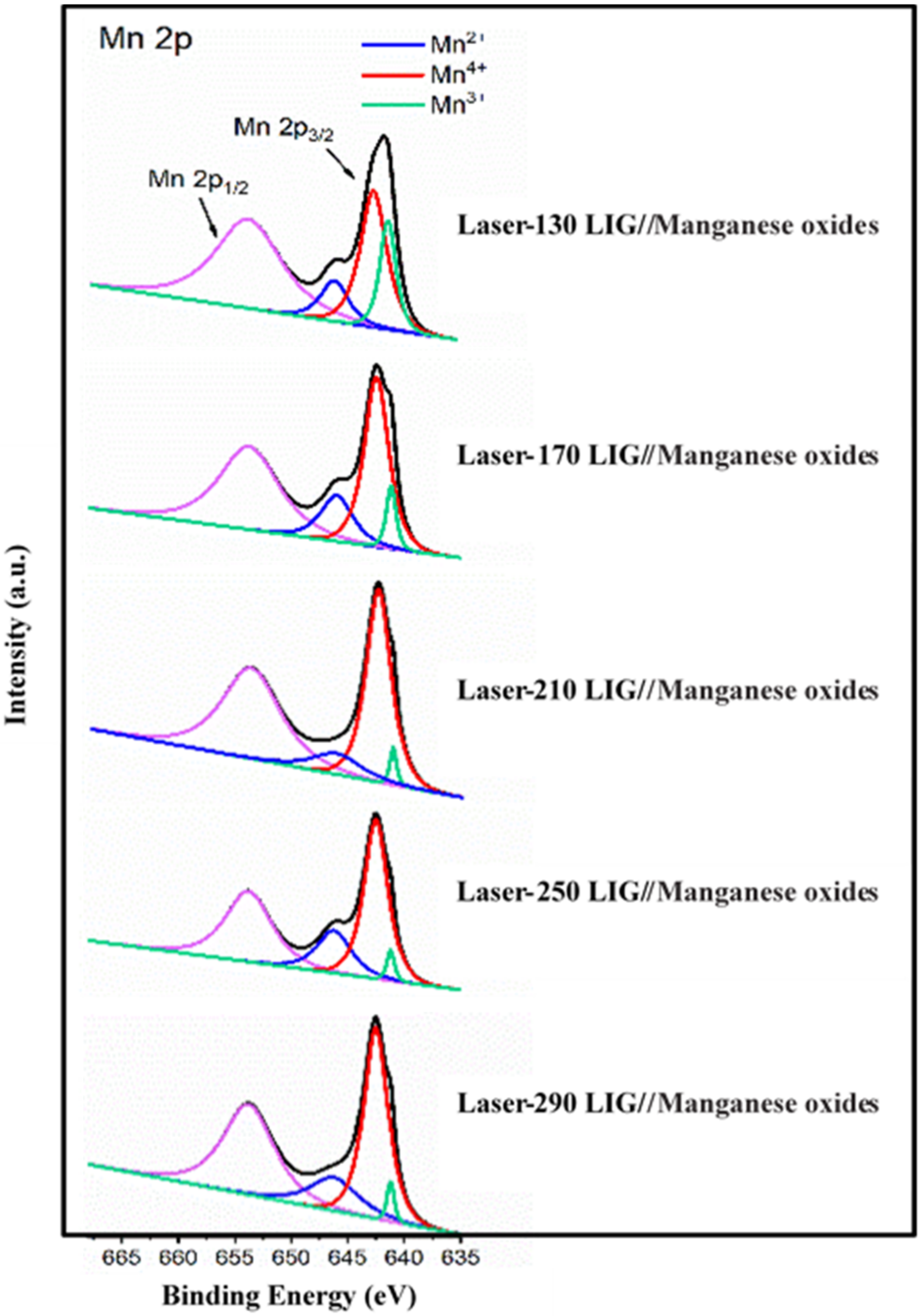

Figure R1.2 XPS spectra of Mn 2p for Laser-130 LIG/Manganese oxide,

Laser-170LIG/Manganese oxide, Laser-210 LIG/Manganese oxide, Laser-250 LIG/Manganese oxide, Laser-290LIG/Manganese oxide

When the fluence of the laser increased to 130 mJ/cm^2, the percentage of Mn^{4+} in the LIG/Manganese oxides exceeded 50%. The percentage of Mn^{3+} in the LIG/Manganese oxides materials remained high when the percentage of Mn^{2+} was at its lowest. As the fluence of the laser gradually increases, the percentage of manganese in different valence states changes.

TableR1.1 Summary of the XPS data for the LIG/Manganese oxides induced by different laser fluence

Material	Mn^{4+}2p [eV]	Mn^{2+} 2p [eV]	Mn^{3+}2p [eV]
Laser-130LIG/Manganese oxides	642.4(53.9%)	646.0(15.5%)	641.1(30.6%)
Laser-170LIG/Manganese oxides	642.6(66.4%)	646.1(23.7%)	641.2(9.9%)
Laser-210LIG/Manganese oxides	642.5**(79.4%)**	646.0**(16.6%)**	641.1**(4.0%)**
Laser-250LIG/Manganese oxides	642.6(69.1%)	646.2(26.7%)	641.2(4.2%)
Laser-290LIG/Manganese oxides	642.6(71.4%)	646.2(24.2%)	641.2(4.2%)

Table R1.1 indicates that the characteristic peaks and the percentage of Mn2+, Mn3+ and Mn4+. The Mn 2p spectra of LIG/Manganese oxides photoinduced by lasers with different fluences indicate that the electron BE assigned to Mn2+, Mn3+ and Mn4+ 2p is close to 646.0 eV, 641.2 eV, and 642.5 eV, respectively. The percentages of Mn4+ and Mn3+ were the highest (79.4%) and lowest (4%) when the laser fluence was 210 mJ/cm2. Conversely, the percentage of Mn2+ ions decreased from 23.7% to 16.6% and then increased from 16.6% to 26.7% as the laser fluence increased. Altering the laser fluence thus enables various material compositions of LIG/Manganese oxides to be successfully synthesized through photomodulation of the reaction mechanisms. The percentage of manganese in different valence states can be adjusted flexibly.

Mn 3s is more sensitive to the average oxidation state of manganese than Mn 2p is. The energy separation between the two peaks (ΔE) is closely related to the mean

manganese oxidation state. The Mn 3s XPS spectra (Fig. R1.3) reveals that the ΔE values of aLaser-130LIG/Manganese oxide, Laser-170LIG/Manganese oxide, Laser-210 LIG/Manganese oxide, Laser-250LIG/Manganese oxide, and Laser-290LIG/Manganese oxide are 5.38, 5.25, 4.75, 4.88, and 5.0 eV, respectively The position of the lower binding energy peak gradually shifts to higher energy as the oxidation number of the sample increases, which is consistent with previous results. The standard ΔE values for MnO_2, Mn_2O_3, and MnO are 4.7, 5.2, and 5.8eV, respectively. When the laser fluence was 210 mJ/cm^2, ΔE was 4.75, which is extremely close to the MnO_2 value.

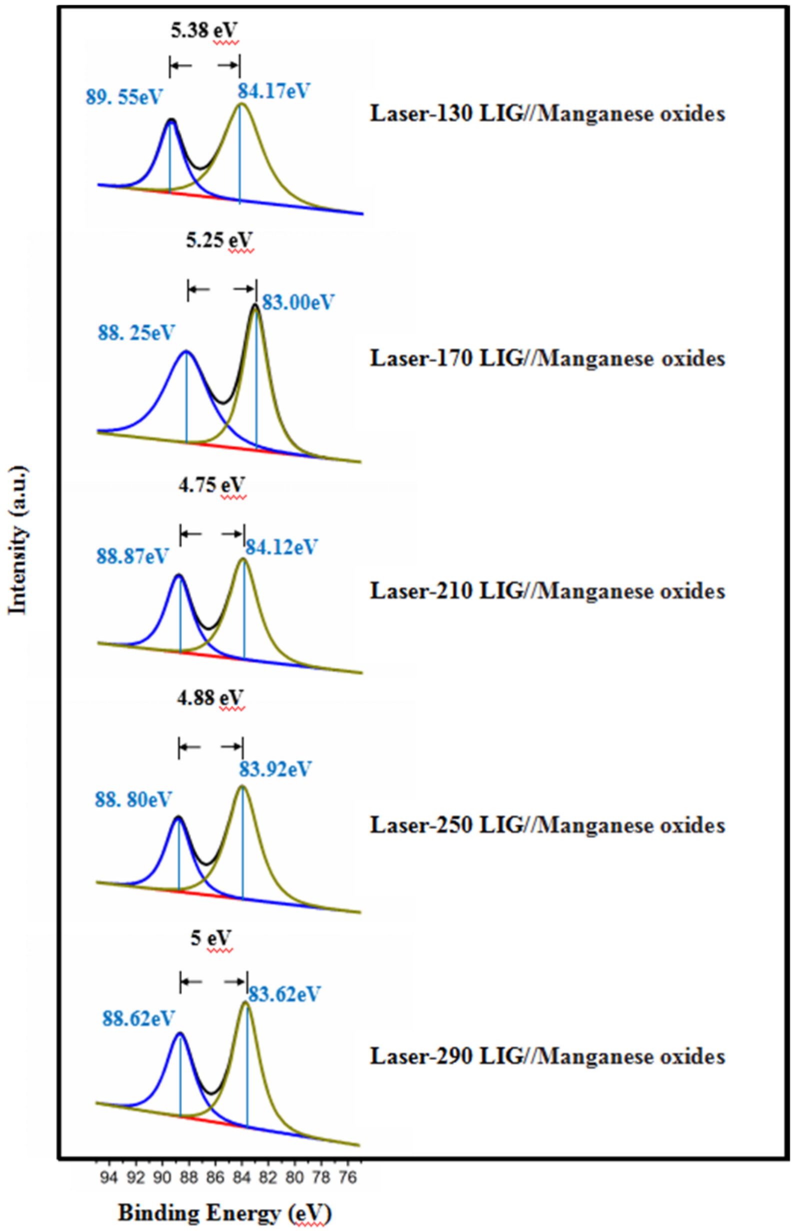

Figure R1.3 XPS spectra of Mn 3s forLaser-130 LIG/Manganese oxide, Laser-170 LIG/Manganese oxide, Laser-210 LIG/Manganese oxide, Laser-250 LIG/Manganese oxide, Laser-290LIG/Manganese oxide

As the laser fluence increases or decreases, the value of ΔE increases, which indicates the presence of manganese oxides of lower-valence states. There are typically defects in the interlayers, such as vacancies and Mn^{4+} substitution by $Mn^{3+}$and Mn^{2+}. This result is consistent with those discussed earlier.

In this study, by altering the pulse energy, materials with various compositions of

LIG/Manganese oxides were successfully synthesized through photomodulation of the reaction mechanisms. By using a spatially shaped femtosecond laser, the ratio of the Mn^{4+} could be maximized, and MnO_2 as the main component is in the LIG/Manganese oxides.

Since the process of laser-regulated material synthesis is very complex, we should make a comprehensive study and exploration of laser-induced products. Therefore, XRD tests were added to characterize the composites irradiated by femtosecond laser, so as to verify the existence of the composites, especially manganese, and observe whether there are new phenomena. The XRD patterns of the as-prepared GO and LIG-MnO_2 nanocomposite crystal structure were analyzed and are shown in figure R 1.4. According to Fig. R 1.4 (a), GO has an intense peak at 2θ = 11.2° that is related to the (001) diffraction. The diffraction peaks of Laser-210 LIG/Manganese oxide appeared at 2θ = 12.73°, 18.14°, 28.82°, 37.46°, 41.95°, 49.86°, 56.32°, 60.27°, 65.11°, 69.76° and 72.73° as shown in Figure R1.4 (b), which correspond to the expected diffraction peaks of (110), (200), (310), (211), (301), (411), (600), (521), (002), (541) and (312) crystal planes of α-MnO_2 standard data, respectively, following the JCPDS card PDF file no. 44-0141. No peaks for other types of MnO_2 are observed, indicating that the as-prepared products composed only of one tetragonal α-MnO_2 phase.

The diffraction peaks of Laser-210 LIG/Manganese oxide are very similar to those of a nanotetragonal phase of α-MnO_2, where the (001) reflection peak of layered GO has almost disappeared. Our results correlate well with the previous studies that the diffraction peaks become weakened or even disappear whenever the regular stacks of GO are exfoliated.

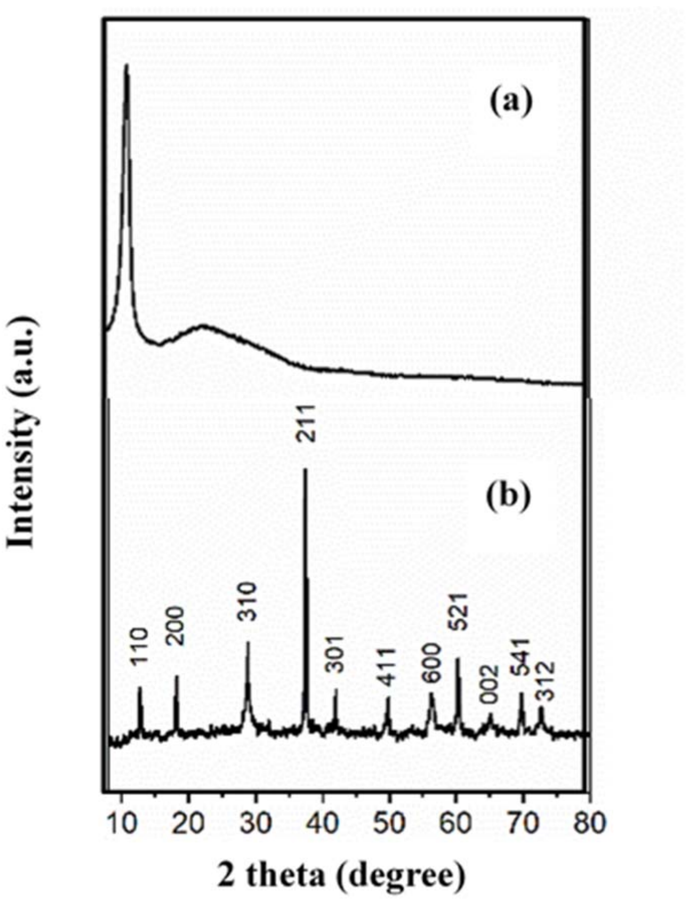

Figure R1.4 (a) XRD patterns of GO and (b) Laser-210 LIG/Manganese oxide

To gain some insights into the structures of LIG/ Manganese oxides, we collected XRD patterns at different composites (Laser-130LIG/Manganese oxides, Laser-170LIG/Manganese oxide, Laser-210 LIG/Manganese oxide, Laser-250LIG/Manganese oxide, Laser-290LIG/Manganese oxide.) processed under different laser fluence (Figure R1.5).

The XRD patterns of the LIG/Manganese oxides LIG showed peaks that were similar, which can be well indexed to plane of the tetragonal α-MnO_2 structure. However, we can still find differences through careful observation. The most obvious one is that the peak intensities of the XRD patterns of Laser-210 LIG/Manganese oxide and Laser-250 LIG/Manganese oxide are stronger than others'.

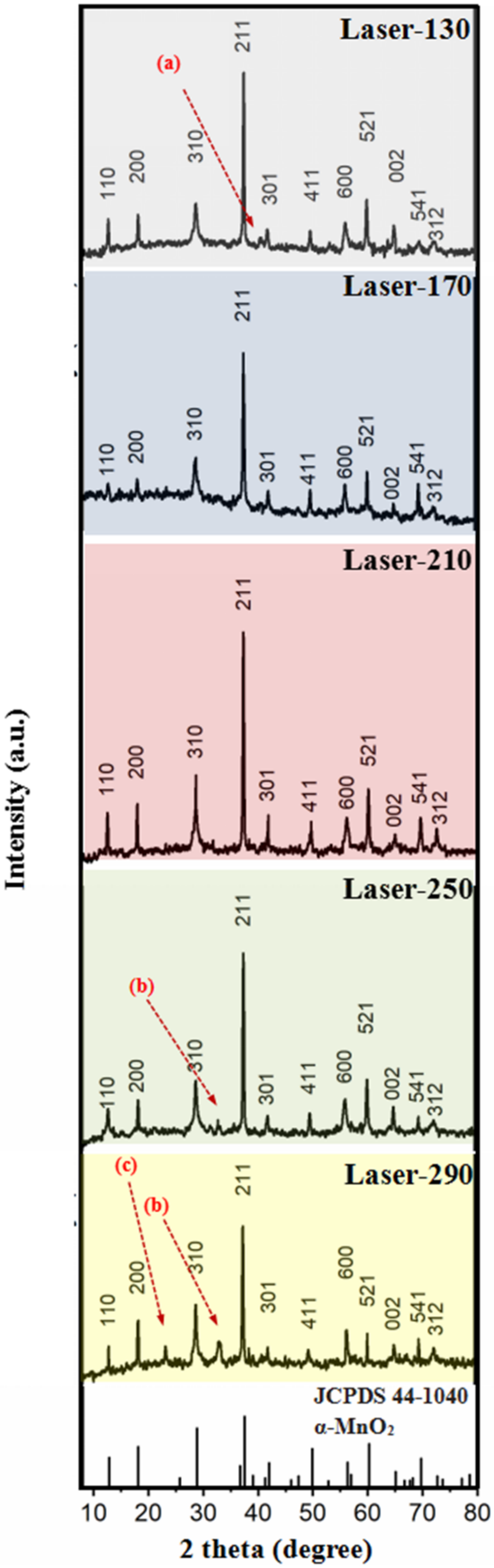

Laser-130
(a)
Laser-170
Laser-210
Laser-250
(b)
Laser-290
(c)
(b)
110
200
310
211
301
411
600
521
002
541
312
JCPDS 44-1040
α-MnO₂
Intensity (a.u.)
2 theta (degree)
10
20
30
40
50
60
70
80

Figure R 1.5 XRD patterns of LIG/Manganese oxides processed under different femtosecond laser fluence

By altering the laser fluence, the diffraction peaks of LIG/Manganese oxides correspond to the expected diffraction peaks of the (110), (200), (310), (211), (301), (411), (600), (521), (002), (541) and (312) crystal planes of α-MnO_2 standard data. However, in the XRD pattern of Laser-130 LIG/Manganese oxide, a peak (a) appeared at 2θ = 40.5° as shown in the figure. This corresponds to the expected diffraction peaks of the (200) crystal plane of MnO standard data, following the JCPDS card PDF file no. 74-4748. This is lower than other peaks, and gradually disappears as the laser fluence increases. When the laser fluences are 250 and 290 mJ/cm^2, peaks (b) and (c) appear at 2θ = 33.05° and 23.12°, respectively. These were assigned to the (222) and (211) crystal planes of α-Mn_2O_3, following the JCPDS card PDF file no. 41-1442. The resulting diffractogram concludes the formation of α-MnO_2 (major) and α-Mn_2O_3 (minor) phases. This may be due to the conversion of MnO_2 to Mn_2O_3 as the laser fluence increases. MnO_2 continues to constitute an extremely high proportion of our laser-induced synthesis materials, especially when the laser fluence is 210 mJ/cm^2.

Thus, the femtosecond laser reduces GO, simultaneously facilitating the action of metal ions, which is a novel mechanism of action. Consequently, GO reduction and MnO_2 synthesis are mutually reinforcing processes in shaped femtosecond laser radiation. By altering the pulse energy, materials of various LIG/manganese oxide compositions were successfully synthesized through photomodulation of reaction mechanisms. By using an SSFL, the Mn^{4+} percentage could be maximized, and MnO_2 is the primary component of LIG/manganese oxide.

(3) Unique laser-induced graphene/MnO_2 composites for enhanced capacitance

The unique femtosecond laser regulated the chemical reaction, enabling synthesis of an excellent composite material with 3D structure. Capacitance is enhanced using an approach that employs materials wherein ions are inserted between atomic layers, resulting in charge storage[R1.22]. These materials can be described as a

2D structure designed to achieve rapid ion transfer between atomic layers. Graphene is a crucial 2D material and has attracted increasing attention owing to its theoretically large specific surface area (~2630 m^2 g^{-1}) [R1.23], excellent electrical conductivity[R1.24] and mechanical flexibility, and other desirable characteristics. However, pure graphene sheets continue to demonstrate certain limitations in terms of practical application because of their low capacitance. As an alternative to precious metal oxides, MnO_2 has exceptional performance, including high theoretical capacity, and other advantages, such as low price, abundant reserves, and environmental friendliness. However, the poor electronic conductivity of manganese dioxide results in high internal resistance of the electrode[R1.25-27]. To foster strengths and circumvent weaknesses, we designed the LIG/manganese dioxide composite electrode. Moreover, for the 3D patterned structure fabricated through a one-step reaction by using a shaped laser, graphene is used as a solid skeleton, and the uniform effect of the light field facilitates favorable distribution of MnO_2 nanoparticles on the graphene skeleton. As shown in Figure R1.6, the porous structure provides a rapid path and several path choices for ion transfer, facilitating rapid and complete interaction of ions and thus quicker charging and discharging.

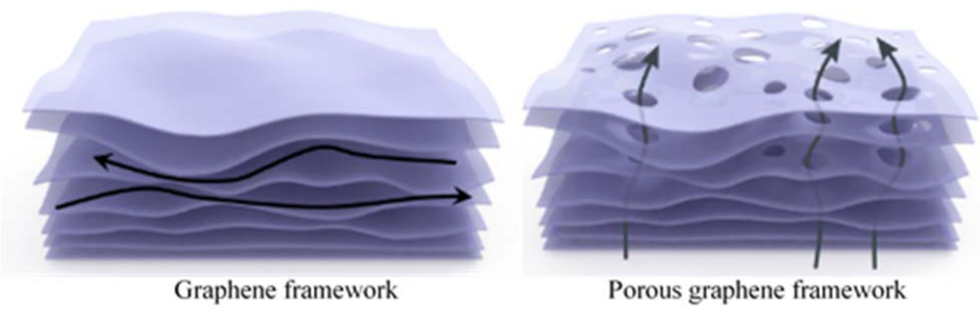

Figure R1.6 Illustration of a compressed graphene framework and porous graphene framework with arrows depicting the ion transport pathways

The 3D porous scaffold is very stable, potentially mitigating mechanical stress within the electrode and enabling long-term cycling stability of electrochemical energy storage systems[R1.28-30]. Our 3D LIG network is supported by MnO_2 nanoparticles, such that the composite provides adequate space for electrolyte ions to

interact with the entire electroactive surface of the electrode, facilitating efficient charge storage. Graphene not only supports MnO_2 nanoparticles but also has a strong connection with each MnO_2 nanoparticle, preventing the aggregation of MnO_2 nanoparticles and resolving the graphene layer restacking issue, thus enhancing electron transport and stability during cycling[R1.19]. The two types of composite material are conducive to high capacitance.

Modifications

According to the comments of the reviewer, we have revised the corresponding part in our manuscript.

The original part form Page 3, Paragraph 2, Line 7 to Line 20 is as flowing:

"The SFLS strategy can simultaneously realize high-precision and ultra-efficient processing of large-scale multiple L-S-MSCs. Benefiting from the straightforward technology, the efficiency of processing L-S-MSCs has been unprecedentedly improved, and thousands of flexible L-S-MSCs could be precisely fabricated in 1 minute. Due to the special uniform light field effect brought by the spatially shaped femtosecond (ss-fs) laser, we have created the structure different from Gaussian light processing which is conducive to the formation of three-dimensional structures and avoid the incomplete processing caused by the uneven light intensity regionalization. Therefore, we transiently synthesize LIG with the uniformly distributed MnO_2 nanoparticles in situ, and simultaneously pattern the novel fluffy and porous three-dimensional composite structures with ultrahigh specific surface area and toughness. Mn^{2+} can facilitate the reduction process of graphene oxide (GO) due to the higher oxidation potential[22,23] and use the anchor sites provided by GO to be oxidized to MnO_2 nanoparticle in situ[24]. In this way, the fabricated LIG/MnO_2 L-S-MSCs are collector-free, arbitrary graphics, ultra-flexible, high-resolution, tens of micrometers in size with high specific capacitance and ultrahigh energy density."

These parts are revised as following:

"The SSFL strategy can perform high-precision and ultraefficient processing of large-scale multiple MSCs in one step. Femtosecond lasers are uniquely characterized by their ultrahigh peak power (>10^{13} w cm^{-2}) and ultrashort irradiation period[22,23]. The

composites of LIG/MnO_2 are synthesized through photomodulation of the reaction mechanisms (photochemical and photothermal reduction/oxidation)[24]. Mn^{2+} can facilitate the graphene oxide (GO) reduction process of because of its high oxidation potential[25,26], and it ca use the anchor sites provided by GO to be oxidized to MnO_2 nanoparticle in situ[27]. At the same time, a fluffy porous three-dimensional structure with ultrahigh specific surface area and toughness can be obtained. The process does not require oxidants, current collectors, demanding reaction conditions, addition of chemicals or metal foam. Furthermore, this SSFL technique is particularly attractive because it is suitable for numerous material systems benefiting from the universality of materials for femtosecond laser processing. The SSFL strategy has ultrahigh efficiency and consistently fabricates high-performance, high-resolution, and flexible MSCs, which is promising for application to advanced miniaturized electronics, such as microelectromechanical systems. This strategy also provides a pathway for high throughput in the industry and designable large-scale flexible energy storage devices."

The original part in Page 10, Figure 3 (figure 3i):

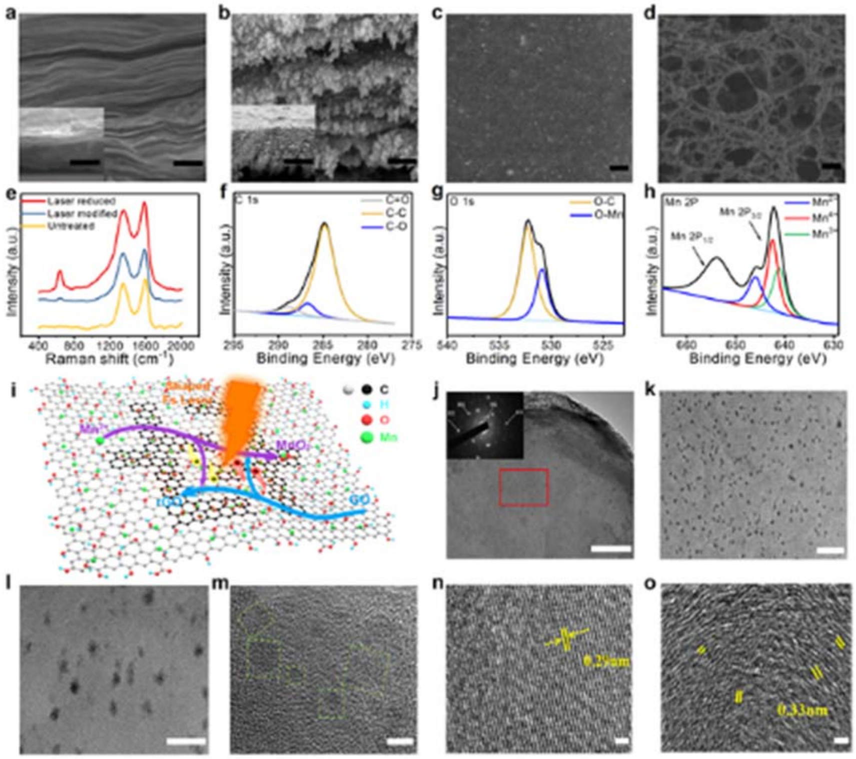

“Fig. 3 |LIG/MnO_2 electrodes material processed by SFLS. a and b, SEM image of cross section view of the hybrid film before and after ss-fs laser ablation(scale bar, 100 nm； inset scale bar, 2 µm). c and d, SEM image of surface of the hybrid film before and after ss-fs laser ablation (scale bar, 500 nm). e, Raman spectra of GO hybrid film untreated, modified by ss-fs laser and reduced by ss-fs laser. f-h, C 1s, O 1s and Mn 2p XPS core level spectra of LIG/MnO_2 film. i, Schematic illustration showing the photosynthetic routes based on redox shuttle for LIG/MnO_2. j, TEM image taken at the edge of the nanocomposite (scale bar, 400 nm). k, Large area of uniform MnO_2 nanoparticles scattered on LIG (scale bar, 100 nm). l, Partially enlarged MnO_2 nanoparticles scattered on LIG (scale bar, 50nm). m, High-resolution TEM (HRTEM) image of MnO_2 nanoparticles scattered on LIG (scale bar, 10nm). n and o, HRTEM image of selected area indicated lattice space (scale bar, 2nm).”

The original part form Page 11, Paragraph 1, Line 1 to Line 10 is as flowing:

“Fig. 3 proposed an enlightening photosynthetic route for synthesizing LIG/MnO_2 at the atomic level. From the schematic diagram, we can clearly observe that in the area irradiated by ss-fs laser, the carbon-oxygen bond is significantly reduced, and a large amount of MnO_2 is generated. In the areas without laser irradiation, more carbon-oxygen bonds and Mn^{2+} exist on GO film. Therefore, the role of ss-fs lasers in synthesizing materials and building three-dimensional structures is very obvious. The ss-fs laser provides a large amount of photon energy, which promotes the autocatalysis of GO, which in turn produces electrons for the reduction of GO. Mn^{2+} will be absorbed on the negatively charged part of the hydrophilic oxygen functional groups of GO. During the reduction of graphene oxide, Mn^{2+} undergo oxidation to become MnO_2 using the energy and oxygen functional groups provided by the GO deoxidation process.”

These parts are revised as following:

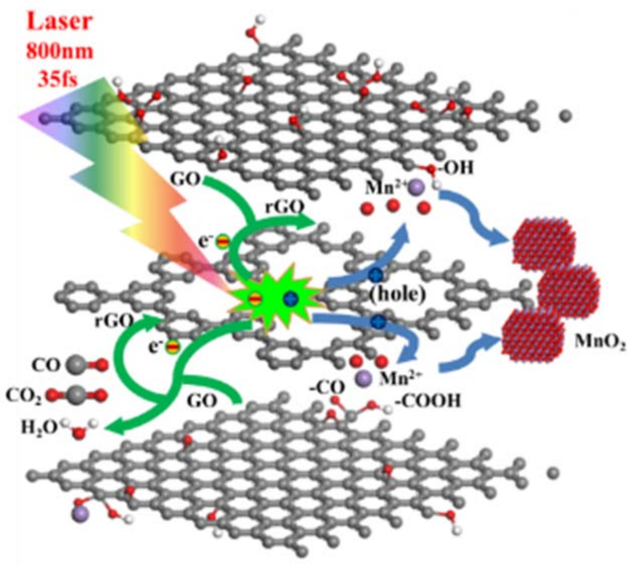

Figure 3a Schematic of the resultant morphology change and phase transformation by SSFL under the proposed photosynthetic routes

Fig. 3a illustrates our proposed photosynthetic route for synthesizing LIG/MnO_2. SSFL provides numerous excited photons that form free-moving electrons and holes, which reduce GO to rGO. Under their action, GO was reduced to rGO. Mn^{2+} is absorbed on the negatively charged part of the hydrophilic oxygen functional groups of GO because of the electrostatic effect. GO-wrapped Mn^{2+} then facilitates the reduction of GO caused by the high oxidation potential and then uses the anchor sites provided by GO to be oxidized into manganese oxide nanoparticles in situ. The energy released from the deoxygenation of GO serves as an effective and in situ power source driving the oxidation of Mn^{2+} into MnO_2. The amount of heat released during the deoxygenation of GO is several times that required to drive the endothermic oxidation of metal ions[38,39]. The equations for the reaction is as follows,

$$\text{(GO)-OH+(GO)-O-(GO)+(GO)-COOH+Mn}^{2+}\text{+e}^{-} \xrightarrow{hv} \text{rGO+MnO}_2\text{+CO}_2\uparrow\text{+CO}\uparrow \quad (3)$$

As the reaction continues, the photothermal reduction gradually emerges, and the cumulative effect of the pulsed laser produces heat, which results in the pyrolysis of GO, whereby oxygen-containing functional groups such as hydroxyl (-OH), carboxyl (-COOH), and oxygen Bridges (C-O-C) are break up into CO, CO_2 and H_2O, which can be removed[40]. When the GO is cracked, the energy and accumulated heat in the

reaction provide the original energy for oxidation to the manganese ions, thus completing the formation of manganese oxide.

$$GO+Mn^{2+} \xrightarrow{\Delta} rGO+MnO_2+CO_2\uparrow+CO\uparrow+H_2O\uparrow \quad (4)$$

Therefore, the SSFL reduction in the experiment was caused by the combined effect of photochemical and photothermal reactions. Our findings indicated that gas was released when the SSFL reduced GO composite film, which is crucial in the formation of 3D porous structure composites. The 3D porous patterned structure fabricated in one step using the SSFL employed graphene as a solid skeleton, and the uniform effect of the light field enabled manganese dioxide nanoparticles to be evenly distributed on the graphene skeleton. The pores functioned as shortcuts for rapid ion diffusion between graphene layers, which increased the speed of ion transport across the compressed film. The 3D porous scaffold was very stable, which would mitigate mechanical stress within the electrode and thus ensure the stability of long-term cycling of electrochemical energy storage systems[41,42]. Therefore, the LIG/MnO_2 composite had improved capacitance."

The original part in Page 9, Paragraph 1:

"**Analytical characterization of the LIG/MnO_2.** Fig. 3a and b depict the cross-sectional view of the hybrid film before and after the ss-fs laser patterning. The original graphene oxide layers were stacked tightly together. Interestingly, after ss-fs laser ablation, the lamellar graphene fluffed up and many MnO_2 nanoparticles were attached to LIG. The SEM revealed the surface of the graphene film also became porous and fluffier after ss-fs laser ablation (Fig. 3c and d). The fluffy and wrinkled structure resulted in larger specific surface area, fast ion transport, and excellent electrochemical performance."

These parts are revised as following:

"**Mechanism and analytical characterization of LIG/MnO_2 3D composite synthesis using the SSFL.** The mechanisms of laser reduction presented in this study fall into two main categories: photochemical and photothermal reduction/oxidation. In the initial stage of SSFL interaction with materials, the photochemical

reduction/oxidation induces nonthermal ultrafast electron excitation, nonlinear absorption, and subsequent oxygen group removal because of the ultrashort pulse width and ultrahigh intensity of the SSFL. The instantaneous intensity of the SSFL provides sufficient kinetic energy to enable oxygen atoms to leave the graphene sheet without damaging it[37]."

The added part is inserted following Page 9, Paragraph 1, Line 1 is as following:

"During the SSFL reduction process, the effect of photothermal reduction/oxidation became increasingly apparent as the laser fluence was increased. By altering the laser fluence, LIG/Manganese oxides were successfully synthesized through photomodulation of the reaction mechanisms (photochemical and photothermal reduction/oxidation). We varied the laser fluence (170-290 mJ cm^{-2}) of the SSFL to investigate differences in conductivity and electrochemical characterization and identify the optimal reduction/oxidation effect. The resistance and conductivity of the material reached their minimum and maximum, respectively, when the laser fluence was 210 mJ cm^{-2} (Supplementary Fig. 7 and 8), which implies that the LIG/Manganese oxides composite has large potential capacitance[43]. As expected, the measured area-specific capacitance of the LIG/MnO_2 MSC was highest under the laser fluence of 210 mJ cm^{-2} (Supplementary Fig. 9). We investigated whether the high-performance electrode materials were successfully synthesized under the interaction of photochemical and photothermal reduction/oxidation at this laser fluence.

The X-ray diffraction (XRD) patterns of the as-prepared GO and LIG/MnO_2 nanocomposite synthesized under the laser fluence of 210 mJ cm^{-2} were analysed (Fig. 3b). The most intensive peak of GO at $2\theta = 11.2°$ corresponds to the (001) reflection. The diffraction peaks of LIG/Manganese oxides appeared at 2θ = 12.73°, 18.14°, 28.82°, 37.46°, 41.95°, 49.86°, 56.32°, 60.27°, 65.11°, 69.76° and 72.73°, which correspond to the expected diffraction peaks of (110), (200), (310), (211), (301), (411), (600), (521), (002), (541) and (312) crystal planes of α-MnO_2 standard data, respectively, following the Joint Committee on Powder Diffraction Standards card PDF file no. 44-0141. We then collected XRD patterns of different composites

(LIG/MnO_2) synthesized under different laser fluence (Supplementary Fig. 10). The different XRD patterns exhibited similar peaks, which could be all well indexed to plane of the α-MnO_2 structure. However, when the laser fluence was considerably lower or higher than 210 mJ cm^{-2}, one or two other weak peaks appeared, and the peak intensities in the XRD patterns were lower.

The Raman spectra were extremely similar when the laser fluence was between 170 and 290 mJ cm^{-2} (Supplementary Fig. 11). We compared the Raman spectra in three cases-untreated, fs-laser modified, and fs-laser reduced-on the basis of the laser fluence (Fig. 3c). Clear G bands were characteristics of sp^2 hybridized C–C bonds at 1580 cm^{-1}. D bands of residual oxygen functional groups and other defects[44] in the graphite layer were detected at 1350 cm^{-1}, and the high intensity of G peak demonstrated a high C–C bond ratio, confirming the formation of new graphite domains. The peak value at 641 cm^{-1} was caused by Mn-O vibrations perpendicular to the direction of the MnO_6 octahedral double chains of MnO_2, indicating the presence of MnO_2 in the mixed electrode material[45]."

The added part is inserted following Page 9, Paragraph 2, Line 6 is as following:

"X-ray photoelectron spectroscopy (XPS) was used to assess the difference before (Supplementary Fig.12) versus after SSFL ablation under a laser fluence of 210 mJ cm^{-2} (Fig. 3d-f). The C 1s spectrum consisted of three peaks: C–C/C=C (284.8 eV), C–O (286.7 eV), and C=O (288.8 eV). After SSFL ablation, the C–O and C=O bonds of LIG/MnO_2 decreased, indicating that certain oxygen-containing groups on the GO hybrid film were removed during the reduction. The O 1s and Mn 2p spectrum further confirmed the presence of MnO_2. Fig. 3g illustrates the peaks centered at 530.9 and 532.3 eV, which correspond to the O–Mn and O–C bonds, respectively[46]. The peaks centered at 653.8 eV could be assigned to the Mn $2p_{1/2}$, and Mn $2p_{3/2}$ peaks were observed at three peaks: 641.2, 642.5, and 646, corresponding to +3, +4 and +2 Mn cations, respectively[47] (Fig. 3f). We also obtained high resolution Mn 2p spectra of LIG/MnO_2 under various laser fluence (Supplementary Fig 13). Supplementary Table presents the characteristic peaks and corresponding percentages of Mn^{2+}, Mn^{3+} and Mn^{4+}. The percentage of the Mn^{4+} and Mn^{3+} are the highest (79.4%) and lowest (4%)

when the laser fluence was 210 mJ cm^{-2}. The percentage of manganese in different valence states could also be adjusted by altering the laser fluence. Mn 3s is more sensitive to the average oxidation state of manganese than Mn 2p was. The energy separation between the two peaks (ΔE) was closely related to the mean manganese oxidation state[48,49]. The Mn 3s XPS spectra (Supplementary Fig. 14) were used to assess the ΔE of the LIG/MnO_2 synthesized under various laser fluence. When the laser fluence is 210 mJ cm^{-2}, the ΔE is 4.75, which is very close to the value of MnO_2."

The corresponding content in Supporting Information is as following:

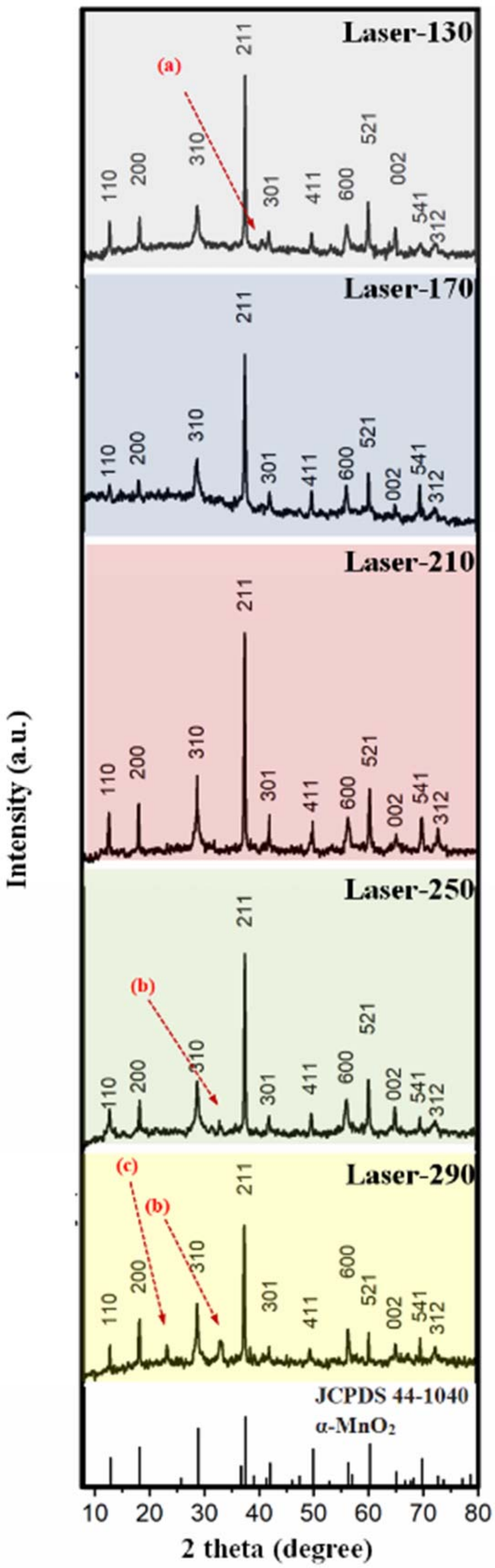
Laser-130
(a)
Laser-170
Laser-210
Laser-250
(b)
Laser-290
(c)
(b)
110
200
310
211
301
411
600
521
002
541
312
JCPDS 44-1040
α-MnO2
Intensity (a.u.)
2 theta (degree)
10
20
30
40
50
60
70
80

Supplementary Figure 10: XRD patterns of LIG/Manganese oxides processed under different femtosecond laser fluences. We defined the composite materials synthesized under different laser fluences as Laser-130, 170, 210, 250 and 290 LIG/Manganese oxide, corresponding to laser fluences of 130, 170, 210, 250, and 290 mJ/cm^2, respectively. By altering the laser fluence, the diffraction peaks of LIG/Manganese oxides correspond to the expected diffraction peaks of the (110), (200), (310), (211), (301), (411), (600), (521), (002), (541) and (312) crystal planes of α-MnO_2 standard data. However, in the XRD pattern of Laser-130 LIG/Manganese oxide, a peak (a) appeared at $2\theta = 40.5°$ as shown in the figure. This corresponds to the expected diffraction peaks of the (200) crystal plane of MnO standard data, following the JCPDS card PDF file no. 74-4748. This is lower than other peaks, and gradually disappears as the laser fluence increases. When the laser fluences are 250 and 290 mJ/cm^2, peaks (b) and (c) appear at $2\theta = 33.05°$ and 23.12°, respectively. These were assigned to the (222) and (211) crystal planes of α-Mn_2O_3, following the JCPDS card PDF file no. 41-1442. The resulting diffractogram concludes the formation of α-MnO_2 (major) and α-Mn_2O_3 (minor) phases. This may be due to the conversion of MnO_2 to Mn_2O_3 as the laser fluence increases. MnO_2 continues to constitute an extremely high proportion of our laser-induced synthesis materials, especially when the laser fluence is 210 mJ/cm^2.

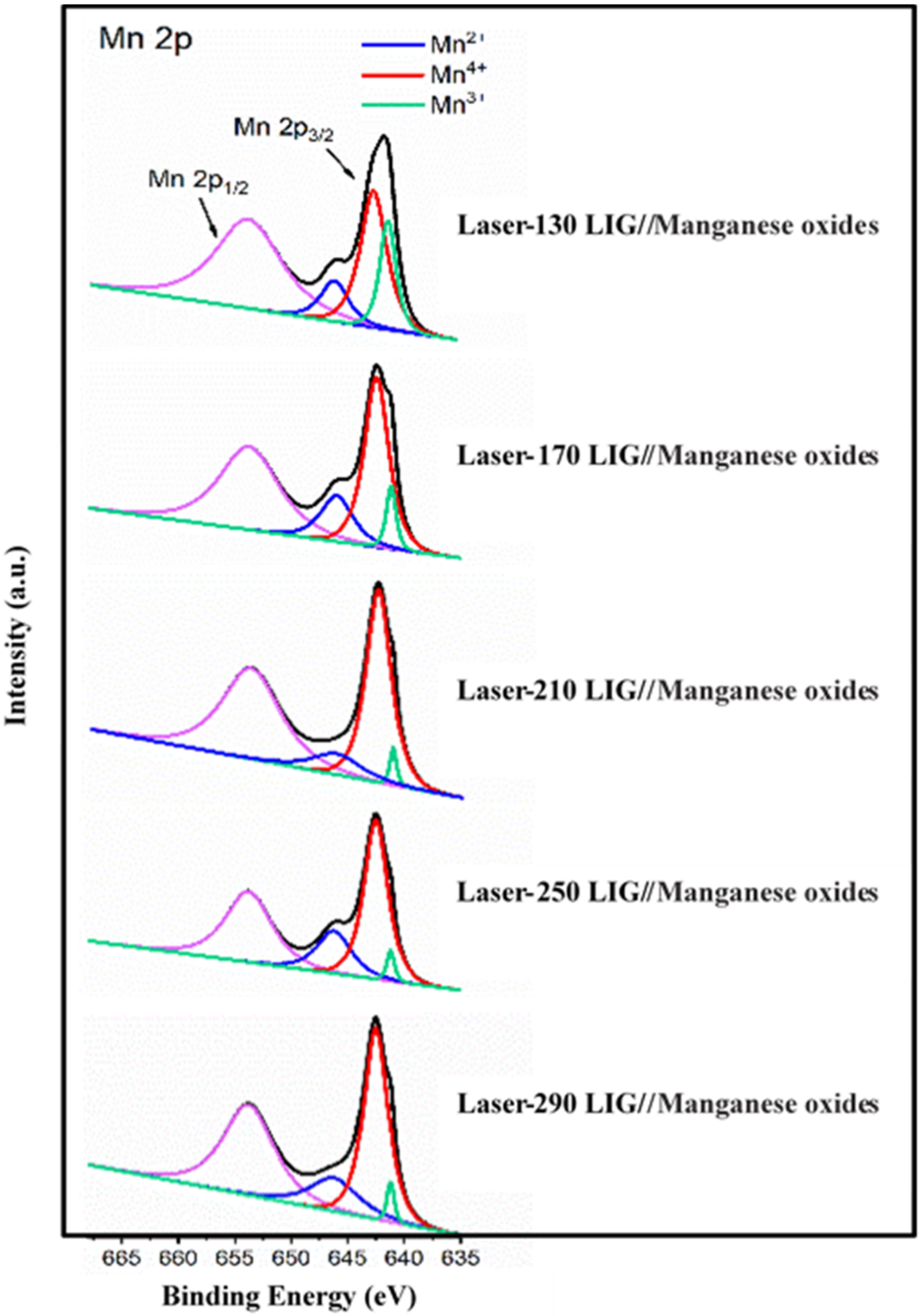

Supplementary Figure 13: XPS spectra of Mn 2p for Laser-130LIG/Manganese oxide, Laser-170LIG/Manganese oxide, Laser-210 LIG/Manganese oxide, Laser-250 LIG/Manganese oxide, and Laser-290LIG/Manganese oxide. To demonstrate that femtosecond laser irradiation can be used to control the synthesis of manganese oxide, we performed XPS measurements to investigate the manganese

oxide and graphene composites. The figure displays the high-resolution XPS spectra of Mn 2p spectra for Laser-130 LIG/Manganese oxide, Laser-170LIG/Manganese oxide, Laser-210 LIG/Manganese oxide, Laser-250LIG/Manganese oxide, and Laser-290LIG/Manganese oxide. Because the duration of the ultrashort pulse was smaller than the time scale of most of the physical and chemical characteristics, this resulted in chemical reaction pathways; chemically selective molecular excitation can be effectively controlled by a femtosecond laser. The Mn 2p XPS spectrum exhibited three characteristic peaks at 641.2, 642.5, and 646 eV, corresponding to spin–orbit peaks of manganese with mixed valence states of +3, +4, and +2, respectively. When the fluence of the laser increased to 130 mJ/cm^2, the percentage of Mn^{4+} in the LIG/Manganese oxides exceeded 50%. The percentage of Mn^{3+} in the LIG/Manganese oxides materials remained high when the percentage of Mn^{2+} was at its lowest. As the fluence of the laser gradually increases, the percentage of manganese in different valence states changes.

Supplementary Table 2 Summary of the XPS data for the LIG/Manganese oxides induced by different laser fluence

Material	Mn^{4+}2p [eV]	Mn^{2+} 2p [eV]	Mn^{3+}2p [eV]
Laser-130LIG/Manganese oxides	642.4(53.9%)	646.0(15.5%)	641.1(30.6%)
Laser-170LIG/Manganese oxides	642.6(66.4%)	646.1(23.7%)	641.2(9.9%)
Laser-210LIG/Manganese oxides	642.5**(79.4%)**	646.0**(16.6%)**	641.1**(4.0%)**
Laser-250LIG/Manganese oxides	642.6(69.1%)	646.2(26.7%)	641.2(4.2%)
Laser-290LIG/Manganese oxides	642.6(71.4%)	646.2(24.2%)	641.2(4.2%)

Supplementary Table 2. Characteristic peaks and percentages of Mn^{2+}, Mn^{3+} and Mn^{4+}. The Mn 2p spectra of LIG/Manganese oxides photoinduced by lasers with different fluences indicate that the electron BE assigned to Mn^{2+}, Mn^{3+} and Mn^{4+} 2p is close to 646.0 eV, 641.2 eV, and 642.5 eV, respectively. The percentages of Mn^{4+} and

Mn^{3+} were the highest (79.4%) and lowest (4%) when the laser fluence was 210 mJ/cm^2. Conversely, the percentage of Mn^{2+} ions decreased from 23.7% to 16.6% and then increased from 16.6% to 26.7% as the laser fluence increased. Altering the laser fluence thus enables various material compositions of LIG/Manganese oxides to be successfully synthesized through photomodulation of the reaction mechanisms. The percentage of manganese in different valence states can be adjusted flexibly.

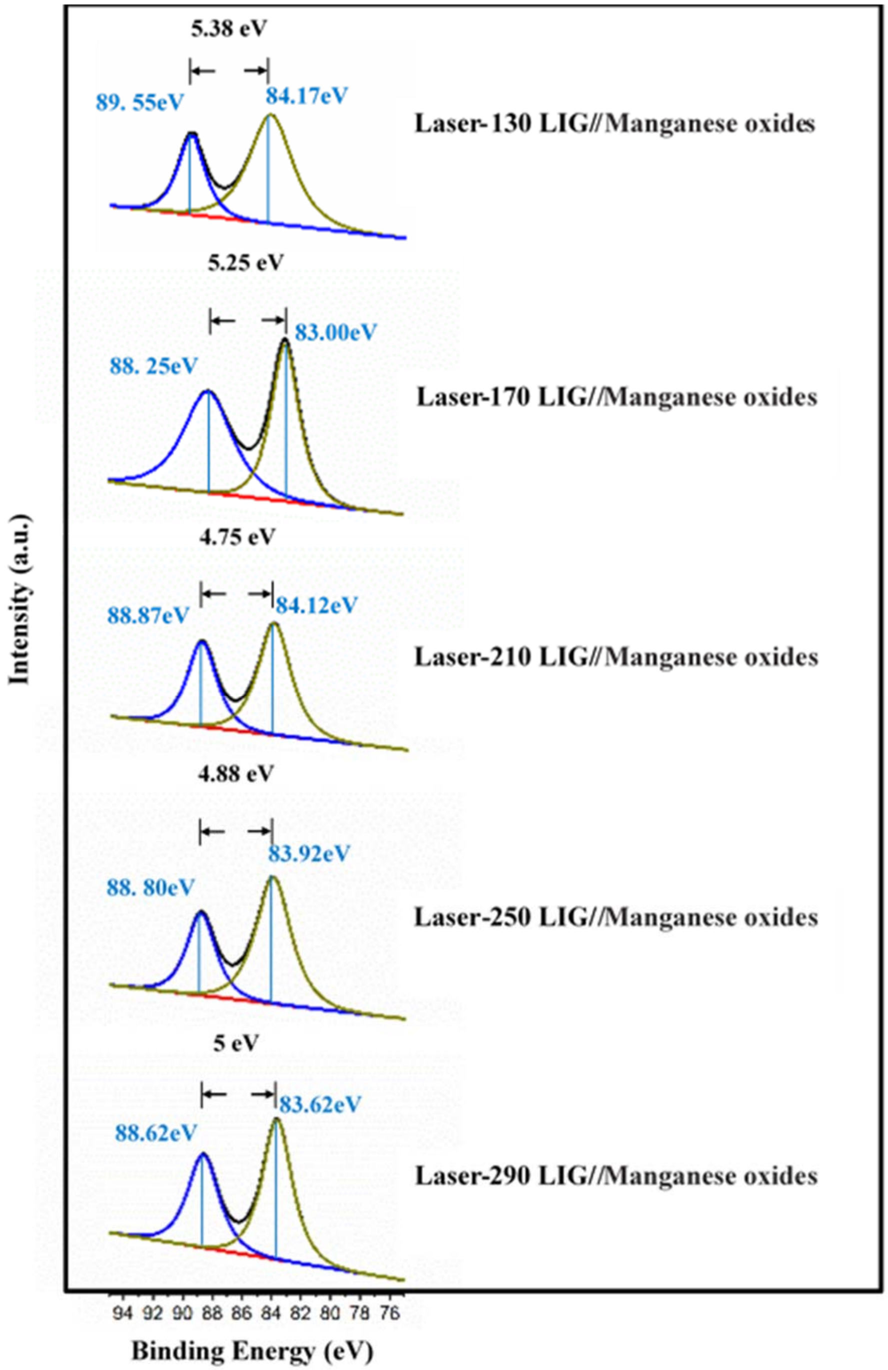

Supplementary Figure 14: XPS spectra of Mn 3s for Laser-130, 170, 210, 250 and 290 LIG/Manganese oxide. Mn 3s is more sensitive to the average oxidation state of manganese than Mn 2p is. The energy separation between the two peaks (ΔE) is closely related to the mean manganese oxidation state. The Mn 3s XPS spectra reveals that the ΔE values of a Laser-130LIG/Manganese oxide, Laser-170LIG/Manganese oxide, Laser-210 LIG/Manganese oxide, Laser-250LIG/Manganese oxide, and Laser-290LIG/Manganese oxide are 5.38, 5.25, 4.75, 4.88, and 5.0 eV, respectively The position of the lower binding energy peak gradually shifts to higher energy as the oxidation number of the sample increases, which is consistent with previous results. The standard ΔE values for MnO_2, Mn_2O_3, and MnO are 4.7, 5.2, and 5.8eV, respectively. When the laser fluence was 210 mJ/cm^2, ΔE was 4.75, which is extremely close to the MnO_2 value. As the laser fluence increases or decreases, the value of ΔE increases, which indicates the presence of manganese oxides of lower-valence states. There are typically defects in the interlayers, such as vacancies and Mn^{4+} substitution by $Mn^{3+}$and Mn^{2+}. This result is consistent with those discussed earlier.

1.1.2 Reviewer's Comments

1.1.2 "The laser reduction (so-called stamping in this manuscript) through a holographic mirror (SLM) in Figs 1&2 are not presented in a proper way. Fig. 2 (c-f) are basically conducted by a patterned mirror. The simultaneous reduction from the patterned beam is effective and valued. But the authors failed to explain why they need 10 min. This indeed unveil if the authors properly explain the laser reduction mechanism, either the same as or different from the well understanding results (photothermal, or non-thermal mechanism)."

Our Response

Thank you for your careful review of our manuscript and your suggestions for the improvement of its quality.

In this work, we mentioned that the processing time of 10 minutes is only to form a specific concept of our technology to compare with other work. This study demonstrated that a processing duration of only 10 min could be achieved using the proposed technology ("More than 30,000 laser-stamped MSCs can be produced in 1 cm^2 within 10 min"), which, when compared with the fabrication speed when other methods are used, reflects the ultrahigh speed of the proposed processing technology. We use the Ti:sapphire laser regenerative amplifier system, which can generate 1,000 single pulses per second. Theoretically, each pulse can fabricate a single MSC, resulting in the fabrication of a single MSC in only 0.001 s. During actual processing, we could fabricate >50 MSCs per second. To help the reader understand our super high processing efficiency as specifically as possible, 10 min was considered a suitable duration to report; hence, we state that >30,000 MSCs can be fabricated within 10 min. To intuitively illustrate the technology, Figures R1.7 and 1.8 illustrate the differences between laser direct writing and our shaped laser processing of MSCs. Figure 1 a shows the whole processing path, in which the transformation of the light field at SLM is magnified. A single laser pulse was innovated into a 3D patterned laser beam to fabricate a compete MSC. Figure 1 b displays that the spatially shaped 3D light field can quickly realize the processing of patterned MSCs. This process realizes the one-step plasticity and reduction of the MSC, which is very close to the form of the stamping, so we call it the laser stamping. Figure 1 c illustrates that the 3D patterned light field is irradiated on the surface of GO hybrid film, thus realizing the three-dimensional reduction and structure of laser-induced graphene/MnO_2. Considering the opinions of the reviewer, we have defined the previous statements more specifically to eliminate misunderstandings. We used the laser photonic-reduction stamping to more vividly represent our processing technology. This laser photonic-reduction stamping process does not require masks and can be arbitrarily changed to achieve ultrafast fabrication of high-solution MSCs with different shapes.

Each individual patterned MSC in Figure 3(c-f) is processed by a single shaped laser. Since a thousand spatially shaped laser pulses can be generated in one second,

we can process many MSCs by controlling the movement of the translation stage. As shown in Figure 3e and f, we can easily fabricate tens of thousands of MSCs and make them integrated into a specific pattern by controlling the movement of the translation platform.

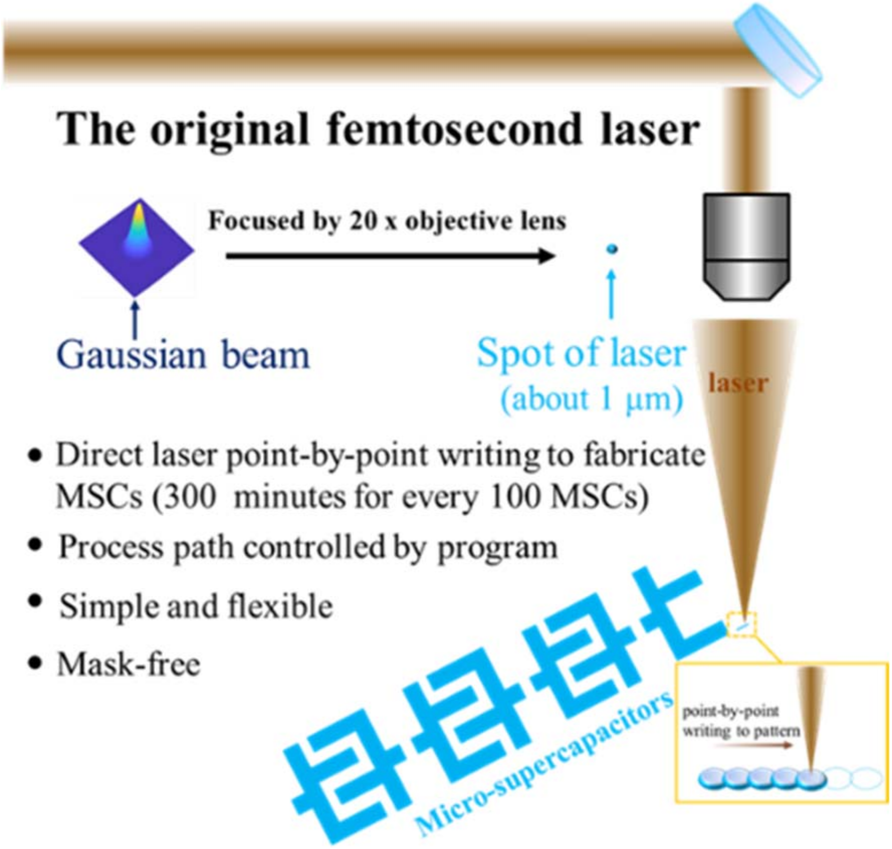

Figure R1.7 Schematic of the fabrication of micro-supercapcacitors using direct laser writing

As shown in the figure R1.7, the traditional laser direct writing does improve the efficiency of the fabrication of MSCs and other micro devices, while also bringing the advantages of the maskless process, flexibility and high resolution. The laser point-by-point writing of the focal spot is realized by controlling the movement of the translation stage. Therefore, in the actual processing, we need to consider the stability of the translation stage and the repeated positioning accuracy to ensure the consistency of the processing, and it is difficult to achieve multi-patterned rapid processing.

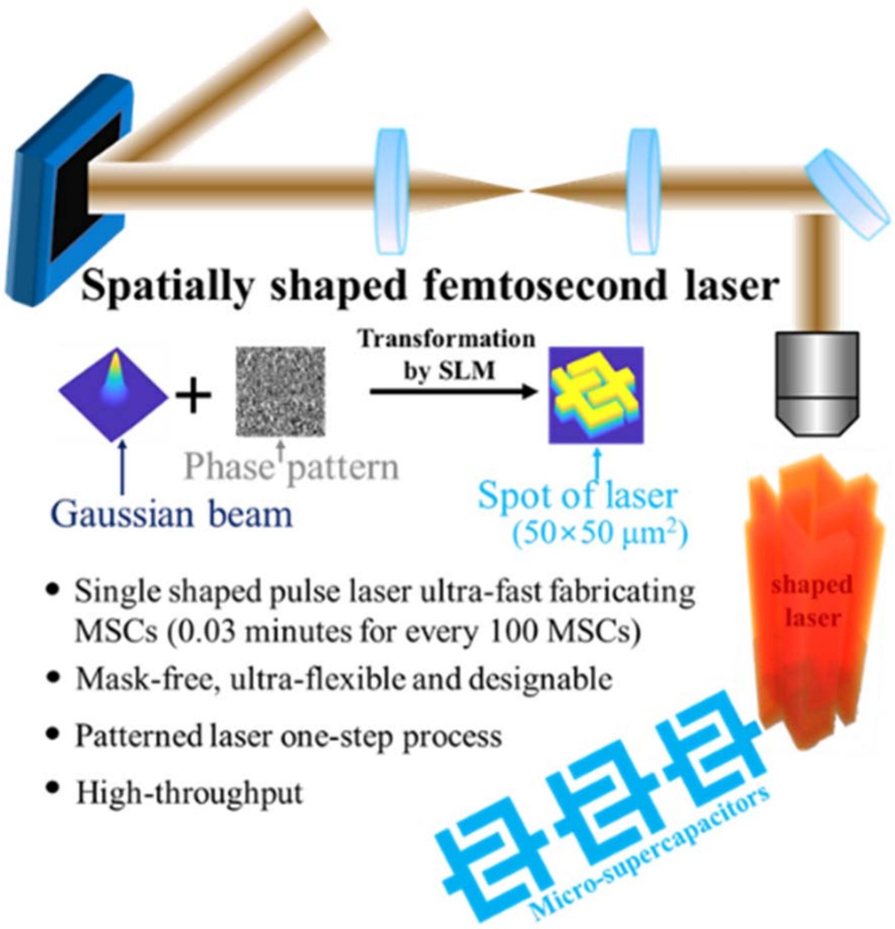

Figure R1.8 Schematic of the fabrication of micro-supercapcacitors using shaped laser

Figure R1.8 shows that the shaped femtosecond laser can be formed in one step and its shape changed by altering the phase pattern. The spot of the shaped laser is a designable pattern spot, which can directly and instantaneously complete patterned MSCs. Along with the advantages of laser direct writing, the proposed approach greatly improves processing efficiency and can achieve consistency in large-area processing. Theoretically, this process can be performed in an instant.

Under the reported experimental equipment and conditions, we generated >30,000 MSCs in less than 10 min. If the translation stage proceeds at an infinite pace with an accuracy of <1 μm, 1.8 million MSCs can be fabricated within 30 min. When comparing the processing efficiency of the present method with that of other methods, we determined the number of MSCs processed within 10 min (or 30 min) as a comparison standard to reflect differences in processing efficiency.

Therefore, "10 min" is simply a form of expression. We apologize for the unclear text and have revised it in the manuscript. Under the reported conditions, we processed 3,000 MSCs in 1 min. This rate is tens or hundreds of times higher than in previously reported MSC fabrication processes[R1.31-36]. As illustrated in the table, our method is comparable with the previously reported methods with respect to manufacturing efficiency, size, and narrow gap. The technology proposed herein has unprecedented manufacturing efficiency and could be employed to fabricate 90,000 MSCs in 30 min.

Table R1.2: Comparison of various manufacturing methods in manufacturing efficiency, size and narrow gap of fabricated MSCs

Manufacturing methods	Size of supercapacitors (narrow gap)	Manufacturing efficiency /30min
Light scribe DVD[32]	0.5×1 cm^2 (150 μm)	100
3D-Printed stamping[33]	1×2 cm^2 (400 μm)	24
Coating and laser etching[34]	1×1 cm^2 (500 μm)	180
Inkjet-printing[35]	1×2 cm^2 (1000 μm)	100
Electrochemical activation[36]	1×1 cm^2	10
Microwave radiation[37]	1×2 cm^2	180
SFLS strategy (Our work)	50×50 $μm^2$ (0.5 μm)	90,000

We deeply appreciate the detailed and constructive suggestions of the reviewer. Regarding the mechanism underlying laser reduction, we did not explain this sufficiently clearly in the manuscript. As indicated in our previous response, the femtosecond laser has ultrahigh peak power ($>10^{13}$ W cm^{-2}) and ultrashort irradiation period, and femtosecond laser fabrication has the unique advantage of nonlinear nonequilibrium processing, providing numerous photons that trigger the generation of electrons and holes.

Numerous thermal treatment approaches including near-field scanning hot tips[R1.37,38] and continuous-wave or quasicontinuous pulsed laser irradiation[R1.31,39] have been previously reported. In those methods, a high-temperature deoxygenation

process through constant thermal treatment is essential to fabrication of LIG. Along with the difficulty in regulating the morphology and structure of LIG, it is challenging to confine heat diffusion.

In this study, the mechanisms underlying GO photoreduction fall under two primary categories: photochemical reduction and photothermal reduction. In femtosecond laser reduction, during the initial stage of the laser's interaction with materials, the primary process is photochemical reduction. The general mechanism involves the removal of oxygen-containing functional groups owing to photoinduction of electron–hole pairs. The energy of laser photons is greater than the bandgap; hence, the electron receives the energy of a photon and jumps from the valence band to the conduction band. The resulting free-moving electron and hole facilitates the reduction of GO to LIG[R1.40,41]. Some studies[R1.42] have proposed electronic excitation with a femtosecond laser as an alternative for GO reduction by performing first-principles simulations. In the present simulations, the laser shot was mimicked using an alternating electric field (E field) and electron–ion dynamics were simulated for an oxidized graphene layer. Furthermore, we analyzed the simulations in accordance with the time-dependent Kohn–Sham equation as follows:

$$i\hbar \frac{d\psi_n(r,t)}{dt} = [H_{KS}(r,t) + V_{wxt}(r,t)]\psi_n(r,t) \quad \text{(E1-7)}$$

Where $H_{KS}(r,t)$ is the Kohn-Sham Hamiltonian as a function of the time-varying charge density $\rho(r,t)$, which is uniquely determined accordingly with a time and spatial dependence of an external field[R1.43] and $\psi_n(r,t)$ is the time-dependent nth Kohn-Sham orbital. This approach is essentially different from past theoretical ideas of enhancing thermal desorption of species by changing the profile of the potential-energy surface with static E field[R1.44,45]. Finally, our study indicates that the femtosecond laser pulse is adequately robust to cleave the C–O covalent bond[R1.46]. Furthermore, the most efficient shape and intensity of the pulse imparts adequate kinetic energy to oxygen atoms, such that the O atoms exit the graphene sheet without imparting significant kinetic energy to carbon atoms despite the heavier mass of the O

atom than the C atom, thus providing conceptual clarity regarding the mechanism underlying the shaped femtosecond laser reduction of GO. This process is suitable for femtosecond lasers with a small pulse width and does not damage the graphene C–C structure or cause local thermal effects.

Upon laser irradiation on the plane of the GO/Mn^{2+} film, the electrons in the valence band absorb the photon energy of the laser and jump to the conduction band. Under the effect of these mobile electrons and holes, GO hybrid films undergo a reduction reaction.

The relevant equation is as follows:

$$(GO)\text{-}OH+(GO)\text{-}O\text{-}(GO)+(GO)\text{-}COOH+Mn^{2+}+e^{-}\xrightarrow{hv}rGO+MnO_2+CO_2\uparrow+CO\uparrow \quad (E1\text{-}8)$$

This reaction mechanism is schematically illustrated in Figure R1.9. The femtosecond laser provides numerous photons that excite electrons to create free-moving electrons and holes. Under their action, GO is reduced to LIG, and Mn^{2+} is absorbed on the negatively charged region of the hydrophilic oxygen functional groups of GO owing to electrostatic effects. Mn^{2+} facilitates GO reduction owing to a higher oxidation potential[R1.16,17] and uses the anchor sites provided by GO for oxidation to MnO_2 nanoparticles in situ[R1.18]. Simultaneously, GO deoxygenation releases energy that serves as an in situ power source driving the novel oxidation reaction of Mn^{2+} [R1.19].

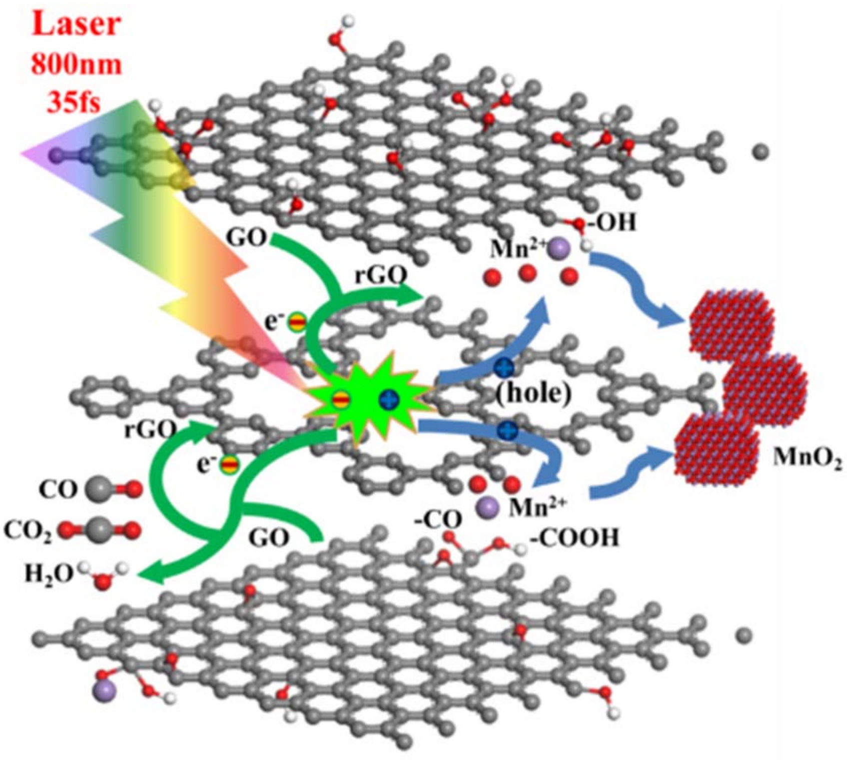

Figure R1.9 Schematic of the resultant morphology change and phase transformation by the SSFL under the proposed photosynthetic routes

On progression of the reaction, photothermal reduction gradually occurs. The cumulative effect of the pulsed laser generates heat, resulting in GO pyrolysis, wherein oxygen-containing functional groups including hydroxyls (-OH), carboxyls (-COOH), and oxygen bridges (C–O–C) are dissociated into CO, CO_2, and H_2O, which can be eliminated[R1.47,48]. On GO cleavage, the energy and accumulated heat in the reaction provide the energy for oxidation to Mn^{2+} ions, thus concluding MnO_2 generation.

$$GO+Mn^{2+} \xrightarrow{\Delta} rGO+MnO_2+CO_2\uparrow+CO\uparrow+H_2O\uparrow \qquad (E1\text{-}9)$$

The high power of the shaped femtosecond laser used herein means that GO is adequately irradiated locally on several occasions. This process results in the photothermal reduction of GO and a porous graphene structure. Because two-photon or multiphoton absorption may occur under the effect of certain compact focusing ultrafast lasers and laser-induced thermal relaxation is common, the femtosecond laser

reduction achieved in this study results from the combinatorial effect of photochemical and photothermal effects.

Modifications

According to the comments of the reviewer, we have revised the corresponding text in our manuscript to better illustrate the ultrahigh efficiency of our technology without confusion.

The original abstract form Page 1, Paragraph 1, Line 3 to Line 6 is as flowing:

"Here, a versatile spatially shaped femtosecond laser stamp strategy is proposed to ultrafastly manufacture the designable flexible laser-stamping MSCs from the graphene oxide based film. More than 30,000 laser-stamping MSCs are produced in 1 cm2 within 10 minutes."

This part is revised as following:

"Here, a flexible, designable MSC can be fabricated by a single pulse laser photonic-reduction stamping. A thousand spatially shaped laser pulses can be generated in one second, and over 30,000 MSCs are produced within 10 minutes. The MSC and narrow gaps were dozens of microns and 500 nm, respectively."

To better highlight the advantages of our technology through comparisons and to enhance the text's comprehensibility to the reader, we have revised some portions of the manuscript and have included appropriate figures for comparison in the supplementary materials.

The original part in Page 2, Paragraph 2, Line 8 is as flowing:

"laser scribed"

This part is revised as following:

"laser direct writing"

The more precise statement added to the original part in Page 2, Paragraph 2, Line 10 is as flowing:

"multiple-spot laser direct writing"

The added part is inserted in Page 3, Paragraph 2, Line 5, the detail is as following:

"The initial Gaussian beam can be made to various beam shapes by using phase modulations, similar to a variable 3D "photonic stamp", which can pattern the MSCs with designable shapes and photoregulate the chemical reactions to synthesize LIG/MnO_2."

The added part is inserted in Page 9, Paragraph 2, Line 3, the detail is as following:

"Each individual patterned MSC in Figure 3 c and f is processed by a single pulse SSFL. We can easily fabricate tens of thousands of MSCs and make them integrated into a specific pattern by controlling the movement of the translation platform."

The original part form Page 6, Paragraph 1, Line 1to Line 9 is as flowing:

"The ss-fs laser can achieve ultra-fast fabrication of various-shapes L-S-MSCs (Fig. 1b). Notably, such high fabrication efficiency for flexible L-S-MSCs is rare, and ss-fs laser also demonstrates high machining accuracy and near faultless processing consistency, which enable rapid and large-scale applications. Under present conditions, we could process 3,000 L-S-MSCs in 1 minute. This rate was tens or hundreds of times more efficient than previously reported processes of MSCs[28-33]. As the Table shows, our SFLS strategy is compared with the methods that have been reported before in manufacturing efficiency, size and narrow gap. The technology we proposed has unprecedented manufacturing efficiency and could fabricate 90,000 L-S-MSCs in thirty minutes. Our processing video is included in the Supplementary Information."

This part is revised as following:

"The SSFL method achieved ultrafast fabrication of variously shaped MSCs (Fig. 1b) and revolutionizes the traditional processing method of direct laser writing (Supplementary Fig. 1). Traditional laser point-by-point writing of the focal spot is realized by controlling the movement of the translation stage, which considerably limits the method's application in the ultrafast fabrication of MSCs. In our work, the spot of each laser pulse can be a designable pattern spot shaped by a spatial light modulator that directly and instantaneously completes a patterned MSC. The SSFL

strategy not only retains the advantages of being mask-free, flexibility and high-resolution, it also achieves the ultrafast fabrication of MSCs.

We used the Ti: sapphire laser regenerative amplifier system, which can generate 1,000 single pulses per second. In theory, a single MSC could be fabricated in only 1 millisecond. The actual processing speed observed was more than 3,000/min, which is tens or hundreds of times more efficient than previously reported processes for fabricating MSCs[31-36]. The SSFL also has the advantages of high machining accuracy and near faultless processing consistency, enabling rapid and large-scale application. As illustrated in the Table, our SSFL strategy was compared with the methods previously reported regarding fabrication efficiency, size, and narrow gap. The proposed technique has unprecedented fabrication efficiency and fabricated high-resolution and micron-scale supercapacitors (a processing video is included in the Supplementary Information)."

The corresponding content in Supporting Information is as following:

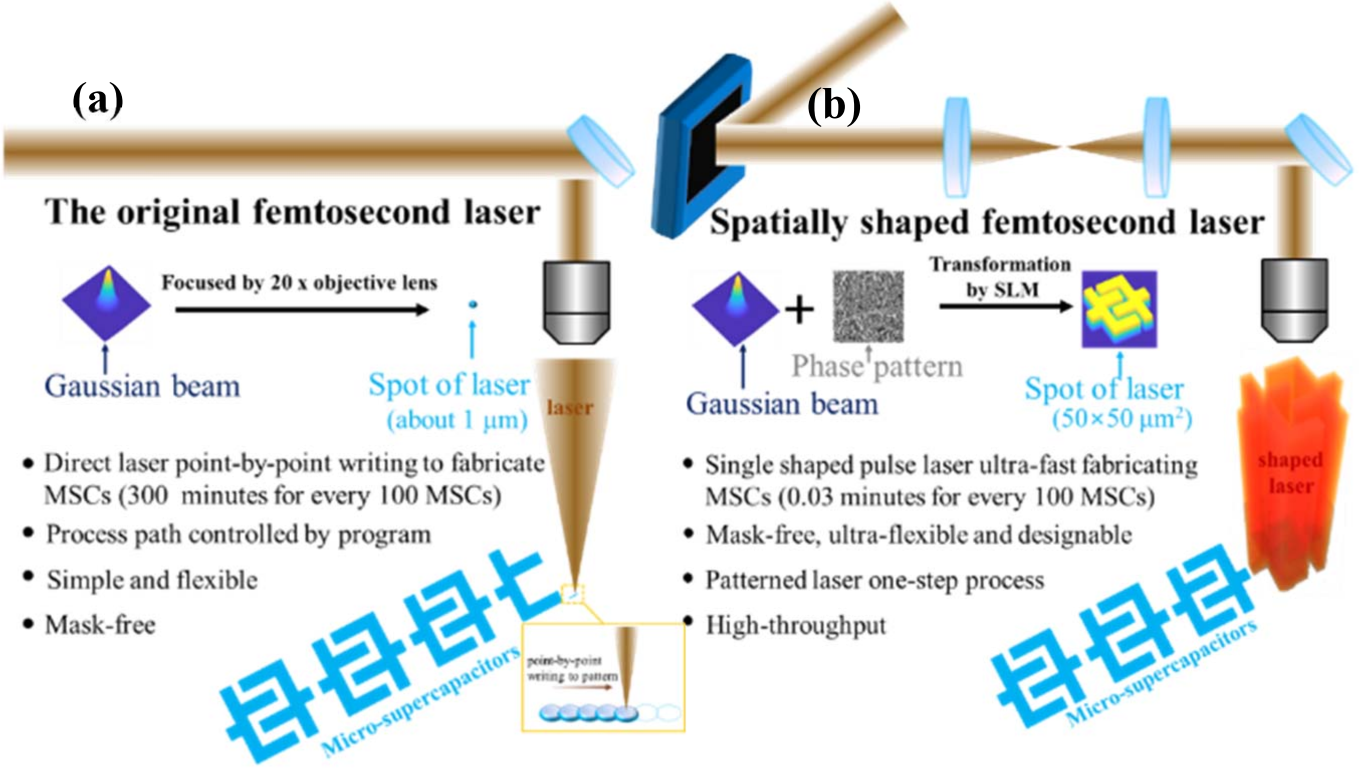

"**Supplementary Figure 1: Schematics of the fabrication of MSCs using (a) direct laser writing and (b) a spatially shaped laser.** Traditional laser point-by-point

writing of the focal spot is realized by controlling the movement of the translation stage. Therefore, in actual processing, we consider the stability of the translation stage and repeated positioning accuracy to ensure the consistency of processing; multipatterned rapid processing is difficult to achieve. The incident beams (Gaussian beams) were transformed into arbitrary geometric target beams in SLM by programming phase patterns; the shaped beams were then transmitted on the hybrid GO films. The shaped femtosecond laser can be formed in one step and alters the shape by changing the phase pattern. The spot of the shaped laser is a designable pattern that can directly and instantaneously complete patterned MSCs. In conjunction with the advantages of laser direct writing, this technology greatly improves processing efficiency and achieves consistency for large-area processing. In theory, this process is instantaneous.

The more explanations of the laser reduction mechanism are added into the new chapter

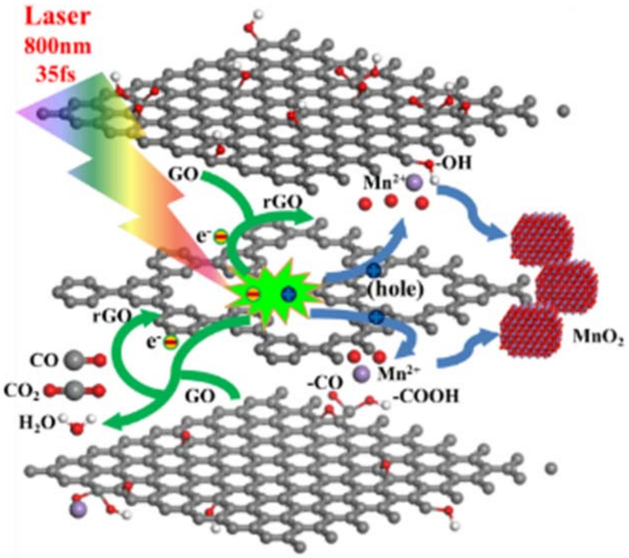

Figure 3a Schematic of the resultant morphology change and phase transformation by SSFL under the proposed photosynthetic routes

Mechanism and analytical characterization of LIG/MnO_2 3D composite synthesis using the SSFL. The mechanisms of laser reduction presented in this study fall into two main categories: photochemical and photothermal reduction/oxidation. In the initial stage of SSFL interaction with materials, the photochemical

reduction/oxidation induces nonthermal ultrafast electron excitation, nonlinear absorption, and subsequent oxygen group removal because of the ultrashort pulse width and ultrahigh intensity of the SSFL. The instantaneous intensity of the SSFL provides sufficient kinetic energy to enable oxygen atoms to leave the graphene sheet without damaging it[37].

Fig. 3a illustrates our proposed photosynthetic route for synthesizing LIG/MnO2. SSFL provides numerous excited photons that form free-moving electrons and holes, which reduce GO to rGO. Under their action, GO was reduced to rGO. Mn2+ is absorbed on the negatively charged part of the hydrophilic oxygen functional groups of GO because of the electrostatic effect. GO-wrapped Mn2+ then facilitates the reduction of GO caused by the high oxidation potential and then uses the anchor sites provided by GO to be oxidized into manganese oxide nanoparticles in situ. The energy released from the deoxygenation of GO serves as an effective and in situ power source driving the oxidation of Mn^{2+} into MnO_2. The amount of heat released during the deoxygenation of GO is several times that required to drive the endothermic oxidation of metal ions[38,39]. The equations for the reaction is as follows,

$$(GO)\text{-}OH+(GO)\text{-}O\text{-}(GO)+(GO)\text{-}COOH+Mn^{2+}+e^{-}\xrightarrow{hv}rGO+MnO_2+CO_2\uparrow+CO\uparrow \quad (3)$$

As the reaction continues, the photothermal reduction gradually emerges, and the cumulative effect of the pulsed laser produces heat, which results in the pyrolysis of GO, whereby oxygen-containing functional groups such as hydroxyl (-OH), carboxyl (-COOH), and oxygen Bridges (C-O-C) are break up into CO, CO_2 and H_2O, which can be removed[40]. When the GO is cracked, the energy and accumulated heat in the reaction provide the original energy for oxidation to the manganese ions, thus completing the formation of manganese oxide.

$$GO+Mn^{2+}\xrightarrow{\Delta}rGO+MnO_2+CO_2\uparrow+CO\uparrow+H_2O\uparrow \quad (4)$$

Therefore, the SSFL reduction in the experiment was caused by the combined effect of photochemical and photothermal reactions. Our findings indicated that gas was released when the SSFL reduced GO composite film, which is crucial in the

formation of 3D porous structure composites. The 3D porous patterned structure fabricated in one step using the SSFL employed graphene as a solid skeleton, and the uniform effect of the light field enabled manganese dioxide nanoparticles to be evenly distributed on the graphene skeleton. The pores functioned as shortcuts for rapid ion diffusion between graphene layers, which increased the speed of ion transport across the compressed film. The 3D porous scaffold was very stable, which would mitigate mechanical stress within the electrode and thus ensure the stability of long-term cycling of electrochemical energy storage systems[41,42]. Therefore, the LIG/MnO2 composite had improved capacitance.

During the SSFL reduction process, the effect of photothermal reduction/oxidation became increasingly apparent as the laser fluence was increased. By altering the laser fluence, LIG/Manganese oxides were successfully synthesized through photomodulation of the reaction mechanisms (photochemical and photothermal reduction/oxidation). We varied the laser fluence (170-290 mJ cm^{-2}) of the SSFL to investigate differences in conductivity and electrochemical characterization and identify the optimal reduction/oxidation effect. The resistance and conductivity of the material reached their minimum and maximum, respectively, when the laser fluence was 210 mJ cm^{-2} (Supplementary Fig. 7 and 8), which implies that the LIG/Manganese oxides composite has large potential capacitance[43]. As expected, the measured area-specific capacitance of the LIG/MnO2 MSC was highest under the laser fluence of 210 mJ cm^{-2} (Supplementary Fig. 9). We investigated whether the high-performance electrode materials were successfully synthesized under the interaction of photochemical and photothermal reduction/oxidation at this laser fluence."

1.1.3 Reviewer's Comments

1.1.3 "Fig. 3s display a lateral resolution is 500 nm, slightly bigger than half of wavelength (800 nm). This indicates a limited thermal reduction by s3hort pulses. Unfortunately, this manuscript does not unveil this physics. It is fine to focus on

manufacturing novelty (if there is any in this work). For a holographic mirror, it is critical to explain the mechanism to complete a 3D reduction without z-scan as a conventional 3D stage. In another word, the authors should present the thickness if they electrically create a flat mirror in SLM. Again unfortunately, in fig. 3 (a, b), authors do not display either the thickness resolution or total thickness they create. This is very misled. The thickness resolution can tell how thin they can write with a holographic mirror and the total thickness directly relates to the intrinsic advantage of a holographic mirror. I do not need guess or deduce which values they should be. But without these, it is hard to know if their calculation of energy density/specific capacitance are correct. Even worse, these formulae provided in the main text and the supplementary materials do not automatically explain how a holographic mirror complete a z-scan. Therefore, Fig. 1 are totally misled. The "stamp" in the title is also misled since the physics is still photonic reduction."

Our Response

Thank you very much for your detailed comments on this article.

As indicated in the comments, for a femtosecond laser (wavelength, 800 nm), the diffraction limit should be half the wavelength. During laser direct writing, breaching the diffraction limit is difficult using the line width accuracy of ablation. However, the SSFL can be used to breach the diffraction limit.

In general, we can transform Gaussian light into shaped light to form patterned light spots, resulting in the laser extruding a narrow gap, breaching the diffraction limit. As indicated in Figure R1.10, we designed the light field with different narrow gaps to obtain processed MSCs with differing narrow gaps. As indicated in our manuscript, the size and shape of our SSFL can be controlled, including the narrow gap of the interdigital MSCs.

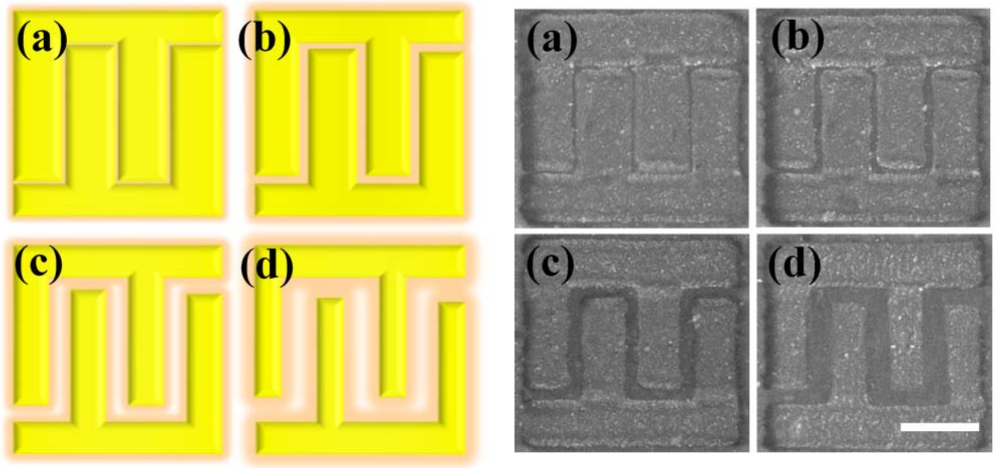

Figure R1.10 The light filed and SEM of interdigital MSCs with different narrow gaps (scale bar, 20 μm). The narrow gaps of the figures (a), (b), (c) and (d) are represented as 500 nm, 2μm, 4μm and 6μm, respectively

During practical processing, the sensitivity of two- dimensional graphene composites to femtosecond lasers is not as high as that of other metallic materials, and their layered structures would be slightly stacked upon laser irradiation. Therefore, we performed spatial light field shaping to transform the original Gaussian light beam into a 3D laser for processing. Our pattern is a complete MSC pattern; hence, patterns with an ultranarrow gap of even a few nanometers can be theoretically designed. However, during actual processing, we achieved a narrow gap of 500 nm for the convenience of subsequent electrochemical analysis, considering light field interference.

We report a method of one-step processing of MSCs by using a patterned shaped laser. When shaping the light field, the laser is a 3D light field. As shown Figure R1.12, different 3D light fields were designed using algorithms.

In order to demonstrate the ability of high-resolution fabrication of our technology, we patterned a simple shape of MSCs to demonstrate a higher resolution of electrode gaps (Figure R1.11-1). We can see that we patterned parallel strip-shaped MSCs with different narrow gaps so that the gap is a straight line and can be shown more clearly. In the figure, we can see that the resolution of the slit is less than 500 nm, and even close to 350 nm in some parts.

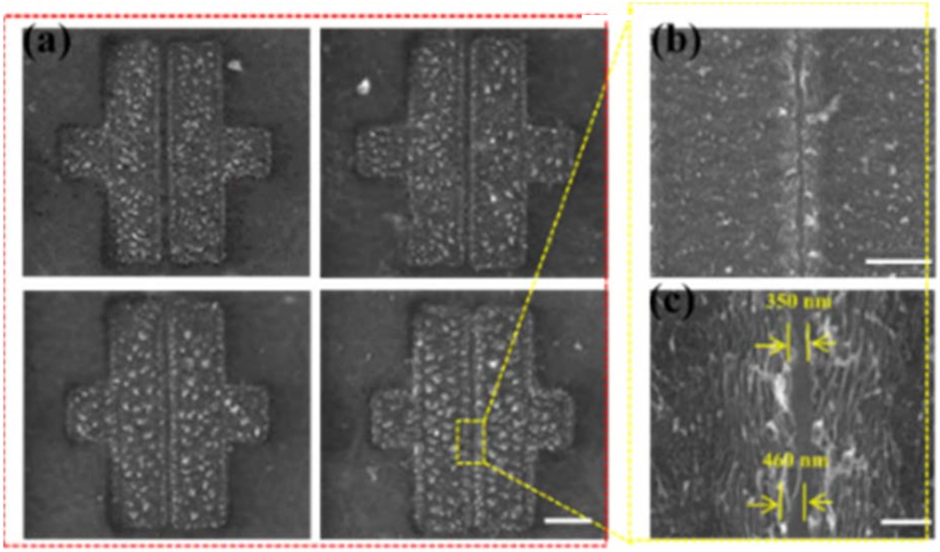

Figure R1.11-1 (a) The SEM images of the MSCs with different narrow gaps, (b) and (c) The SEM images of partial magnification of the narrow gap (scale bar of (a), 10 μm. scale bar of (b), 5 μm. scale bar of (c), 1 μm)

Our resolution is realized through the pattern design of light field shaping. In this way, the slit between design patterns is used to achieve the highest resolution, which is not limited by the laser wavelength. It maybe can explain the way to complete a 3D reduction by the 3D shaped laser. In order to demonstrate the machining resolution of our technology, we carried out higher-resolution experiments using the SSFL. We designed the MSC with the shape of two rings, and the distribution of the light field is shown in figure R1.11-2 (a). The high-resolution patterning is realized by adjusting the narrow gap between the two rings, the figure R1.11-2 (b) shows the SEM images of the patterns with different narrow gaps. We can clearly see that the narrow gap can be designed with the shaped laser, and we got the ultra-small narrow gap of 11.6 nm, which is well beyond the traditional diffraction limit. The results of nanometer precision benefit from the design of shaping light fields.

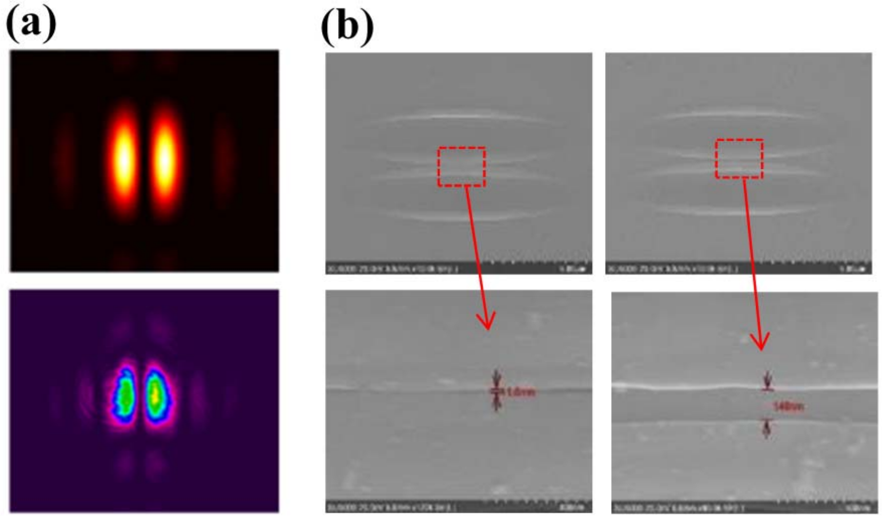

Figure R1.11-2 The light filed (a) and SEM images (b) of the two-rings electrodes with different narrow gaps (11.6 nm and 146 nm)

We report a method of one-step processing of MSCs by using a patterned shaped laser. When shaping the light field, the laser is a 3D light field. As shown Figure R1.12, different 3D light fields were designed using algorithms.

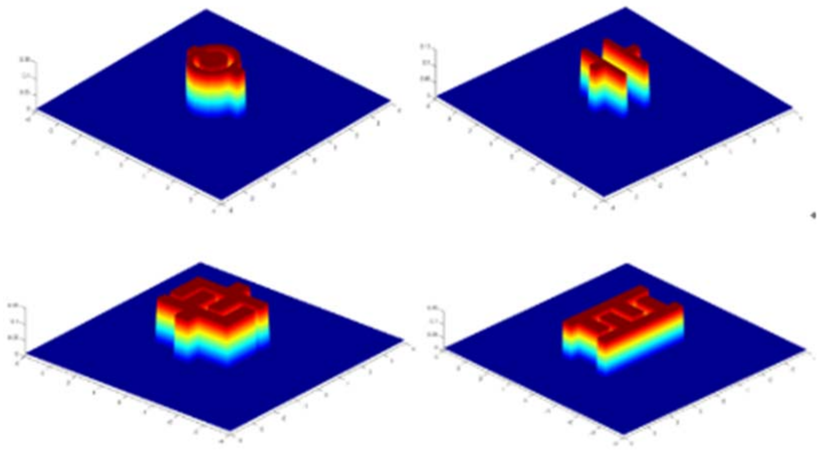

Figure R1.12 Three-dimensional light fields with different shapes are designed by algorithms

We regulated the size, narrow gap, and depth of the 3D light field by using an algorithm and software. We designed a 3D light field of 15 × 15 μm^2 to 100 × 100 μm^2 with a depth of 15 μm. As observed in Figure R1.12, the light fields had a regular shape and could be extended along the z-axis. Because the SSFL has a uniform light field on each plane along the z-axis, excellent 3D reduction could be achieved when

processing the GO hybrid film.

During actual processing, the thickness and actual processing depth of the GO film, determined using the shaping laser, should be considered. As shown in Figure R1.13, the shaping laser can simultaneously reduce graphene and synthesize manganese oxide particles in a layered material.

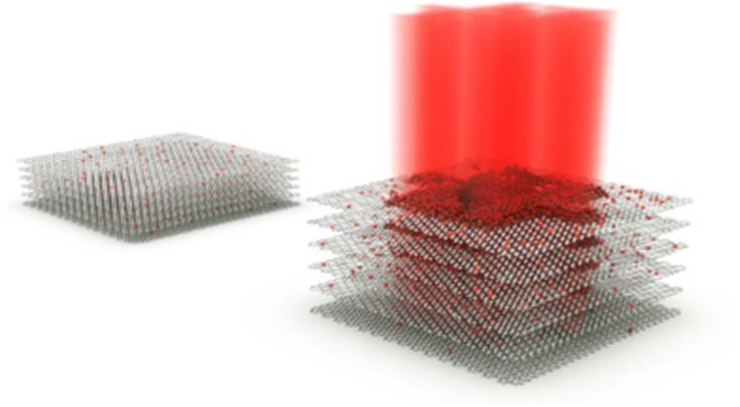

Figure R1.13 The original GO/manganous acetate composite film and the shaped laser processed composite LIG/MnO_2 film.

Owing to the extremely high peak energy of the femtosecond laser, the GO film can be reduced along three dimensions. During reduction, MnO_2 nanoparticles are generated and become attached to the graphene layered structure, increasing the spacing in the graphene layer and yielding a 3D composite material. Furthermore, gases are released during the reduction, which is one of the reasons for the 3D porous structure of graphene. To determine the actual processing depth, we analyzed scanning electron microscopy (SEM) images of the GO hybrid film before and after shaped laser processing (Figure R1.14). The cross-sectional images verified our previous conclusion that upon laser irradiation, the layer spacing of GO hybrid films was increased and MnO_2 nanoparticles became adhered.

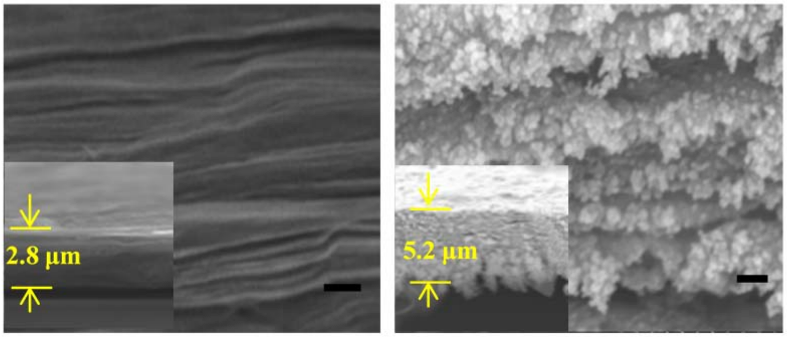

Figure R1.14 SEM image of cross section view of the GO hybrid film before and after shaped laser ablation (scale bar, 100 nm)

Accordingly, we verified the ability of a 3D shaping laser to process 3D-structured graphene composites in one step. We failed to clearly explain the 3D processing method, especially along the z-axis, and we have now included numerous additions and modifications.

To better explain the effect of MSC thickness on electrochemical performance, we generated composite graphene films of different thicknesses (1, 3, and 5 μm) for processing MSCs. The cross-sectional SEM images of hybrid GO films of different thicknesses are displayed in Figure R1.15.

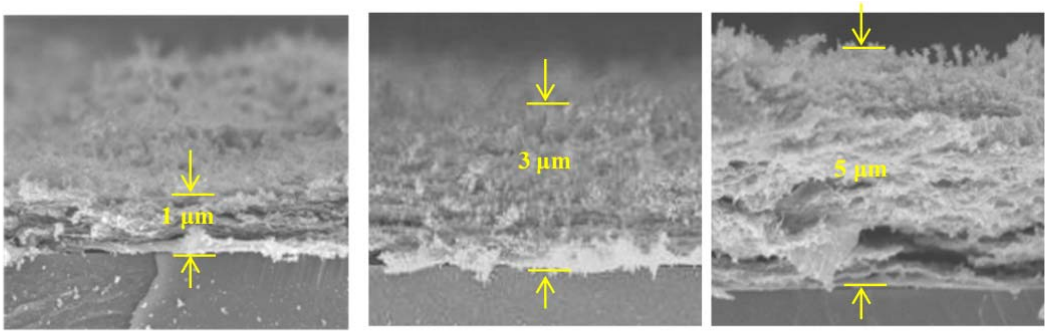

Figure R1.15 SEM image of cross section view of the hybrid film with different thicknesses

To obtain an intuitive view of the 3D morphology of the MSCs, we have included laser confocal microscopic images obtained using a MPLAPONLEXT 100× lens (Figure R1.16). In these images, the hybrid GO film after SSFL patterning displays a clear bulge, and under the effect of the light field, uniform patterning and

z-axis processing are revealed.

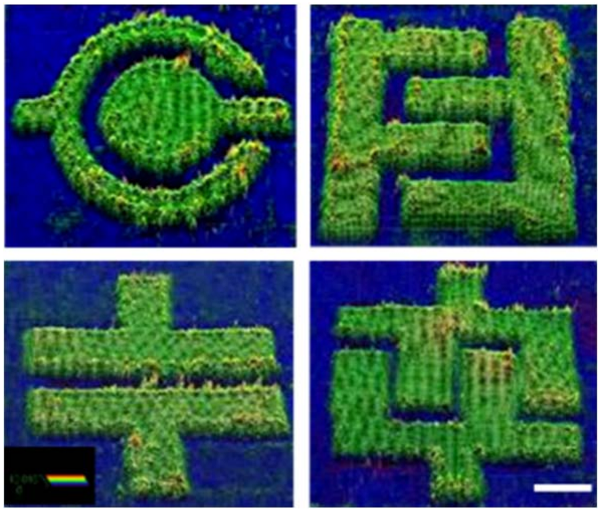

Figure R1.16 The laser confocal microscope images of the MSCs with different shapes for the three-dimensional morphology (scale bar, 20 μm)

Figure R1.16 displays marked differences in thick and thin morphologies through the color fractions symbolizing height. The patterned areas were thicker than the unprocessed areas, indicating the potential of SSFL technology to fabricate hybrid GO films in three dimensions. Furthermore, we determined the exact distance along the z-axis. The thickness in the selected region was determined using laser real-time feedback signals. Figure R1.17 indicates the distribution of roughness along the lines in each laser confocal microscopic image of the MSCs with different shapes. Line roughness was automatically determined using software. The yellow slope lines in Figure R1.17 correspond to the measured area in the images of the MSCs with different shapes.

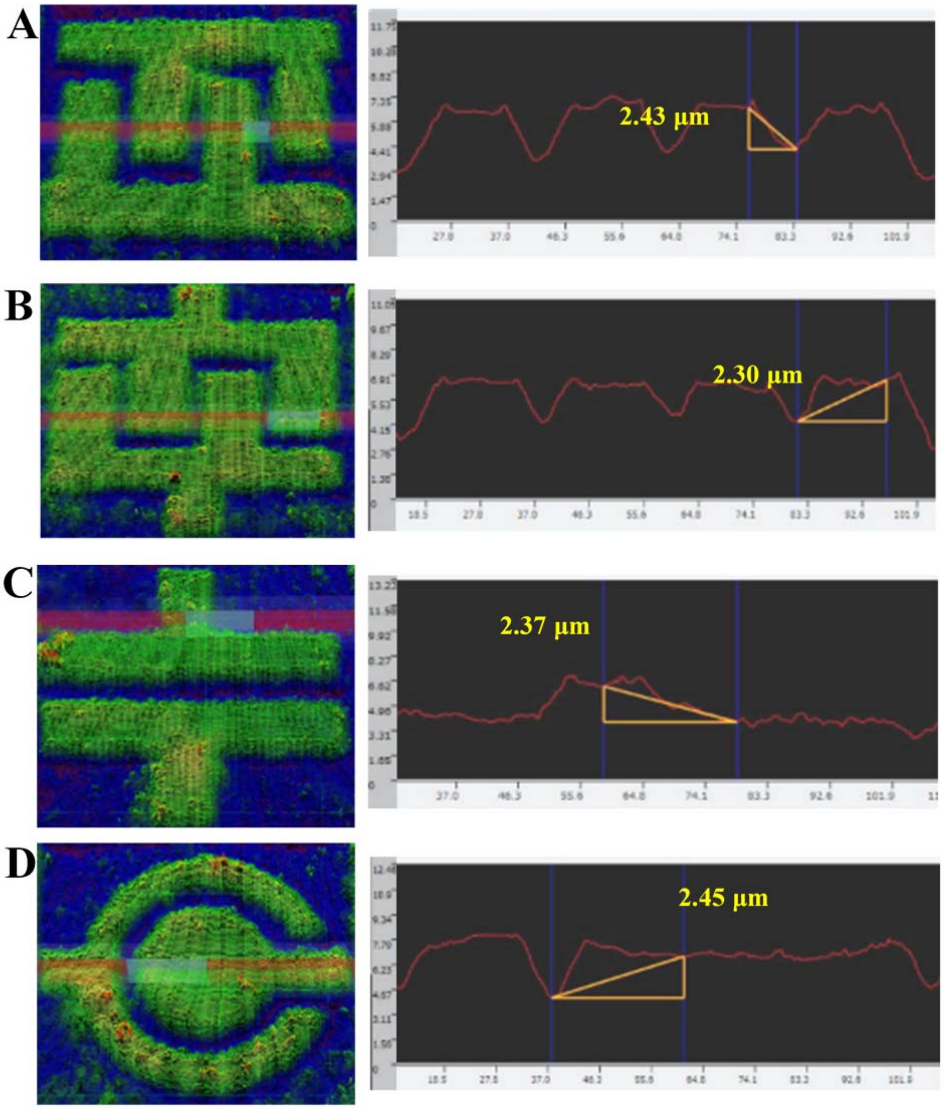

Figure R1.17 Laser scanning confocal microscopic images of the MSCs and the distribution of roughness along the line, determined automatically using software.

Patterns A, B, C, and D have height differences of 2.43, 2.30, 2.37, and 2.45 μm, respectively, all within 2.3 to 2.5 μm, thus reflecting that upon SSFL irradiation of the surface of the GO hybrid film, the spacing of the GO film layer is increased and laser-induced graphene/MnO_2 was synthesized. On the surface, differences in the z-axis between the laser-reduced heterostructure and region without laser processing are obvious. Combined with the aforementioned cross-sectional SEM images, we thus proved the processing capacity of the SSFL along the z-axis, and a porous 3D composite material was synthesized using an SSFL.

For a microscale energy storage device, complete harnessing of the limited potential to boost areal capacitance and energy density is critical. In brief, the 3D expandability of MSCs based on the planar configuration is of great significance. The increase in thickness improves the areal capacitance and energy density, concurrent with our aforementioned objectives. Additionally, we must consider the volumetric capacitance and energy density.

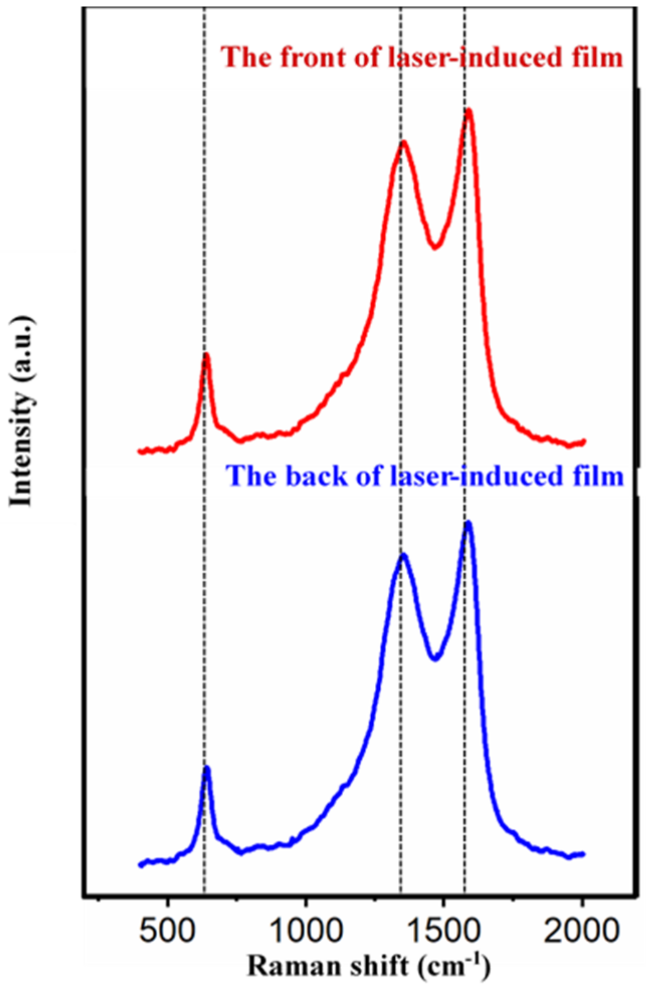

Figure R1.18 Raman spectra of the front and back of the hybrid film irradiated by frontal incident femtosecond laser

To elucidate the 3D machining potential of our technology, we used SSFL irradiation only on one surface of the 5-μm-thick hybrid film. Because the film is very

flexible, it can be easily stripped away. After processing using the shaped femtosecond laser, characterization tests were performed using Raman spectroscopy, XPS, and XRD on both surfaces of the irradiated film. On comparing the ablative conditions of both surfaces of the film processed using a laser, it could be determined whether our SSFL completed 3D processing, especially along the z-axis.

In the Raman spectrum of both surfaces of the irradiated film (Figure R1.18), the clear G bands are characteristic of sp^2 hybridized C–C bonds at 1580 cm^{-1}. D bands of residual oxygen functional groups and other defects in the graphite layer were detected at 1350 cm^{-1}, and the high intensity of the G peak revealed a high C–C bond ratio, confirming the formation of new graphite domains. The peak at 641 cm^{-1} corresponded to Mn–O vibrations perpendicular to the direction of the MnO_6 octahedral double chains of MnO_2, indicating the presence of MnO_2 in the mixed electrode material. The peaks and intensity were very similar for the two surfaces.

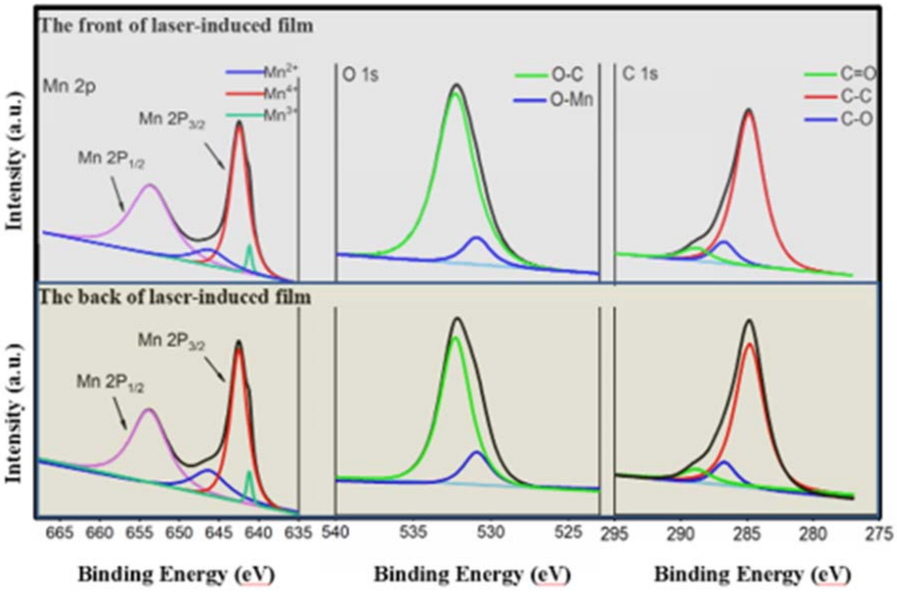

Figure R1.19 Mn 2p, O 1s and C 1s XPS core level spectra of the front and back of the hybrid film irradiated by frontal incident femtosecond laser

XPS measurements were used to reveal the two surfaces of the hybrid film after laser frontal ablation (Figure R1.19). The C1s spectrum could be deconvoluted into three peaks: C–C/C=C (284.8 eV), C–O (286.7 eV), and C=O (288.8 eV). The peaks

corresponding to C–O and C=O bonds on the two sides of film were markedly similar. The O 1s and Mn 2p spectra further confirmed the presence of MnO_2. Peaks at 530.9 and 532.3 eV corresponded to the O–Mn and O–C bonds, respectively. The peaks at 653.8 eV could be assigned to Mn $2p_{1/2}$, and Mn $2p_{3/2}$ peaks were observed at three peaks: those at 641.2, 642.5, and 646 eV, corresponding to Mn (□), Mn (□) and Mn (□), respectively. Thus, MnO_2 was the major MnO_2 phase. Consistent with the XPS results, the two sides of the composite films had similar peaks and intensities in the O 1s and Mn 2p spectra.

XRD patterns of both surfaces of the as-prepared GO hybrid film after laser frontal ablation are displayed in Figure R1.20. The spectrum of the original GO hybrid film had an intense peak at 2θ = 11.2°, which was associated with (001) diffraction. The diffraction peaks of both surfaces of the hybrid film after laser frontal ablation were observed at 2θ = 12.73°, 18.14°, 28.82°, 37.46°, 41.95°, 49.86°, 56.32°, 60.27°, 65.11°, 69.76°, and 72.73°, corresponding to the expected diffraction peaks of the (110), (200), (310), (211), (301), (411), (600), (521), (002), (541), and (312) crystal planes in α-MnO_2 standard data, respectively, in accordance with the JCPDS card PDF file no. 44-0141. No peaks for other types of MnO_2 were observed, indicating that the as-prepared products comprised only one tetragonal α-MnO_2 phase.

XRD patterns obtained after laser frontal ablation indicate the presence of diffraction peaks at 2θ values consistent with those reported previously for planes of the tetragonal α-MnO_2 phase. The XRD patterns of the film's two surfaces were very similar in both peaks and intensity, differing considerably from the XRD patterns of the original GO film.

Using different characterization methods, we concluded that when the shaped femtosecond laser irradiated GO film with a certain thickness from the anterior surface, the processing effect was consistent on both sides of the irradiated composite film, indicating that our 3D femtosecond laser can process materials along the z-axis to complete 3D processing, and the material on the underside of the film still has a favorable reduction effect.

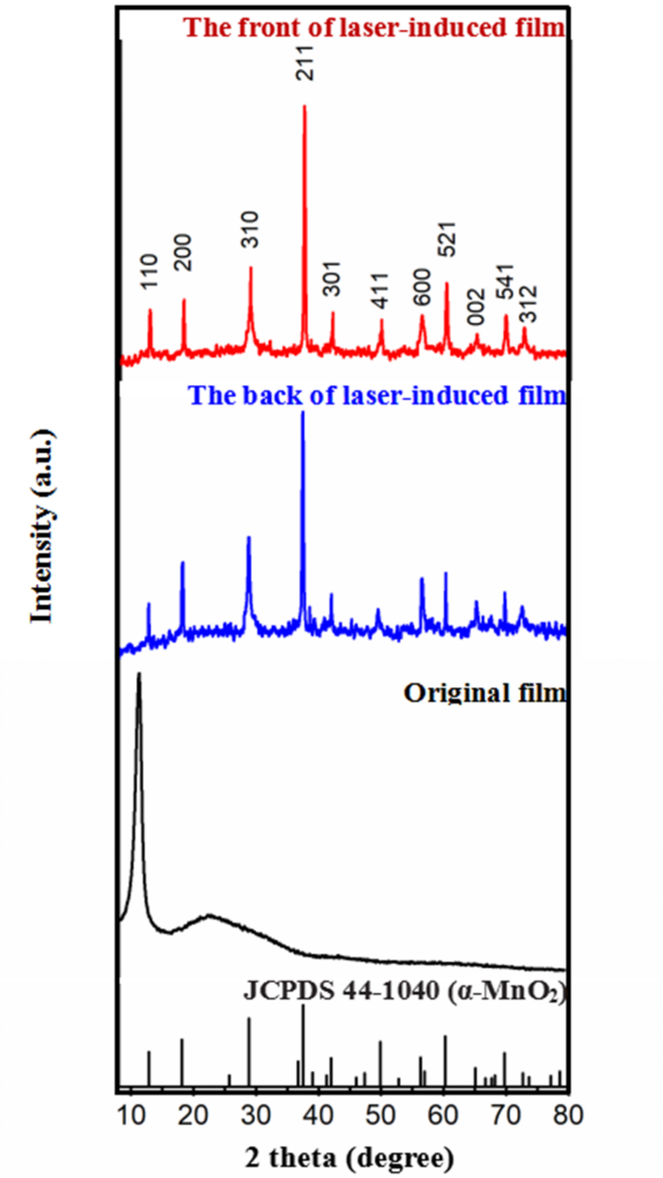

Figure R1.20 XRD patterns of the front and back of laser-induced film and original film

Figures R1.21(a) and (b) display cyclic voltammetry (CV) characterizations of MSCs with different thicknesses at 80 and 800 mV s^{-1}. As expected, the thicker the MSC, the larger the areal capacitance. The areal capacitances of the MSCs with thicknesses 1, 3, and 5 μm were 13, 38, and 64 mF cm^{-2} at 80 mV s^{-1}, respectively. With an increase in the scan rate, the difference in the areal capacitance caused by thickness gradually decreased. The 5-μm-thick MSC still had the largest areal capacitance.

Figures R1.21(c) and (d) show the areal and volumetric capacitance of MSCs

with different thicknesses as functions of the scan rate. Regarding areal capacitance, the greater the thickness of the MSC, the higher the capacitance performance. Regarding the determination of the volumetric capacitance, its effect on its thickness was intuitive. Therefore, volumetric capacitance can better explain the true influence of thickness on performance than can capacitance. The volumetric capacitance of MSCs of different thicknesses was highly similar. In particular, at low scan rates, the volumetric capacitance of the 3-μm-thick MSC was slightly greater than that of MSCs of the other two thicknesses. At high scan rates, the 1-μm-thick MSC had the advantage in the comparison. According to comprehensive analysis, the 3-μm-thick MSC could maintain favorable performance in both areal and volumetric capacitance.

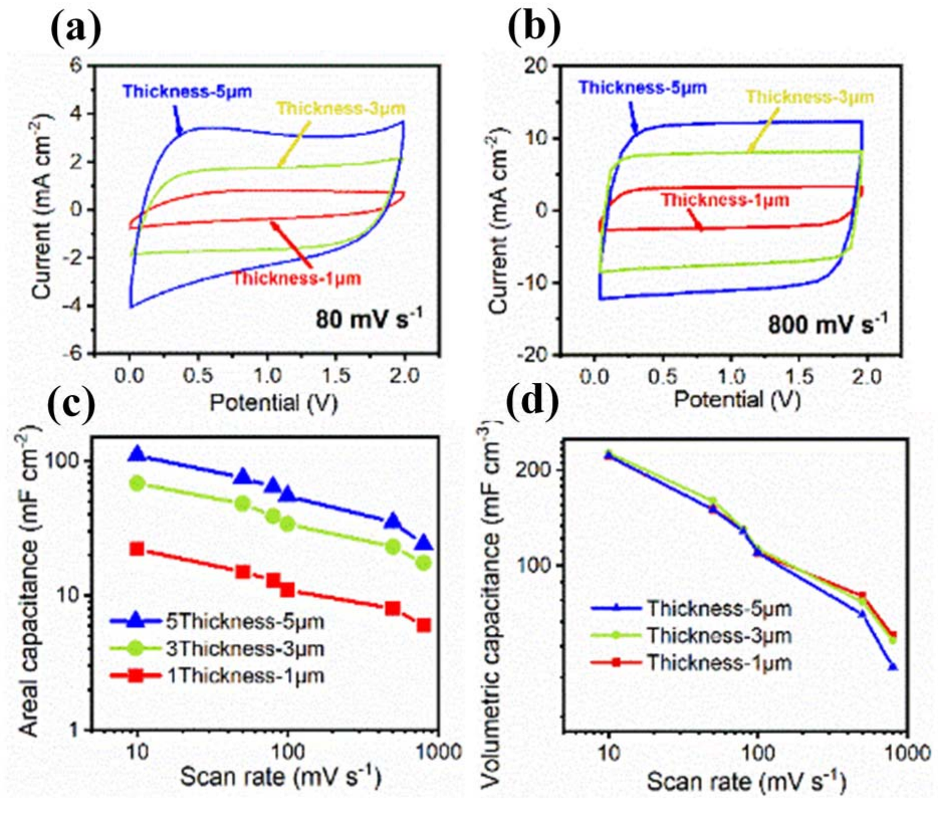

Figure R1.21 The comparison of CV characterizations at low (a) and high (b) scan rates of the MSCs with different thicknesses. (c) and (d) Areal and volumetric capacitance of MSCs with different thicknesses as functions of the scan rate

Thank you for your meticulous efforts and kind assistance in reviewing our

manuscript. As previously stated, our study used designable 3D spatial light to achieve processing along the z-axis. To reinforce the concept of simple, one-step molding, we used a "stamp," which is similar to a postmark, to describe this technology. Compared with the traditional stamp, our optical "stamp" can be arbitrarily changed. Considering the opinions of the reviewer, we have defined the previous statements more specifically to eliminate misunderstandings. We used the laser photonic-reduction stamping to more vividly represent our processing technology. This laser photonic-reduction stamping process does not require masks and can be arbitrarily changed to achieve ultrafast fabrication of high-solution MSCs with different shapes. And our laser photonic-reduction is different from the traditional stamping, which is not a mechanical process but a one-step photonic-reduction process. In order to more vividly reflect the ultrfast, one-step shaping characteristics of our technology, we define the laser photonic-reduction stamping.

Modifications

According to the comments of the reviewer, we have added the corresponding part in our manuscript to better illustrate the high-resolution strategy for fabricating MSCs.

The original part form Page 7, Paragraph 1, Line 13 to Line 18 is as flowing:

"L-S-MSCs of various shapes are in the tens to hundreds of micrometers in length. In such a machining precision, our technology has obtained a satisfactory machining effect. Supplementary Fig. 2 clearly displays the regular electrode morphology. The spacing could be adjusted by designing different light field, and the narrow gap of the L-S-MSCs is only 500 nm (Supplementary Fig. 3). The varying sizes can be controlled by designable light fields from 15×15 μm^2 to 100×100 μm^2 (Supplementary Fig. 4)."

This part is revised as following:

"The shape of the spot could be designed, and the size of the spot could be regulated by transforming the target light field. The size of the spot determined the size of the entire MSC, because the MSCs of various shapes were patterned using the

SSFL in one step. The varying sizes can be controlled by designable laser spots from 15×15 μm^2 to 100×100 μm^2 (Supplementary Fig. 3). We could transform Gaussian light into shaped light to form the patterned light spots and use the SSFL to extrude the narrow gap of patterns that could break the diffraction limit. MSCs with different narrow gaps could be fabricated by designing different light fields, and a MSC with a narrow gap of 500 nm was fabricated using SSFL on a GO hybrid film (Supplementary Fig. 4)."

According to the comments of the reviewer, we have added the corresponding part in our manuscript to explain the 3D reduction of the SSFL.

The added part is inserted in Page 5, Paragraph 1, Line 3, the detail is as following:

"We used the optimized algorithm to regulate the optical field region, in which the beam width and z-axis propagation distance represented the basic parameters of a 3D optical field. We arbitrarily altered the parameter design to control the size of the optical field and the fixed depth for 3D processing within a certain range. An excellent 3D reduction was achieved in the processing of GO hybrid film because the SSFL had a uniform light field on each plane in the z-axis direction."

The original part form Page 9, Paragraph 1, Line 1 to Line 4 is as flowing:

"Fig. 3a and b depict the cross-sectional view of the hybrid film before and after the ss-fs laser patterning. The original graphene oxide layers were stacked tightly together. Interestingly, after ss-fs laser ablation, the lamellar graphene fluffed up and many MnO_2 nanoparticles were attached to LIG."

This part is revised as following:

"The 3D porous structure irradiated by SSFL could be clearly observed on the cross-sectional view and the surface of the hybrid film before and after the SSFL patterning (Fig. 2d). The original GO layers were stacked tightly. Notably, after SSFL laser ablation, the lamellar graphene fluffed up, and numerous MnO_2 nanoparticles were attached to LIG. The porous structure resulting from the gas release and the action of 3D light field in the reduction process has the advantages of high surface area, remarkable thermal conductivity, excellent electronic conductivity and and

favorable mechanical properties. More importantly, these pores function as shortcuts for rapid ion diffusion between graphene layers and increase the speed of ion transport across the compressed film. The detailed analysis and schematic diagram are in the Supplementary Fig.5. The superiority of this special 3D porous structure can be fully demonstrated in the subsequent electrochemical performance tests."

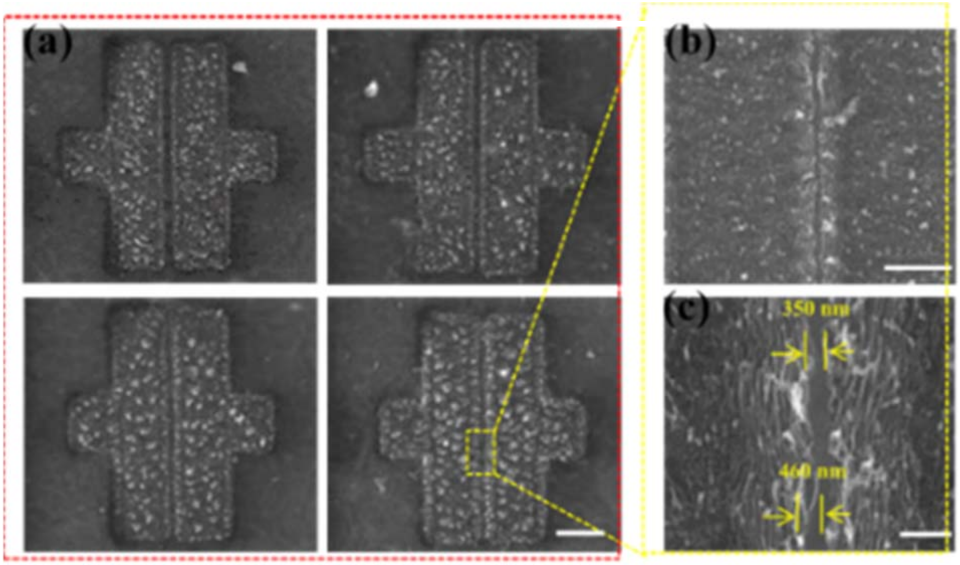

"Supplementary Figure 4-2: (a) SEM images of MSCs with different narrow gaps; (b) and (c) SEM images of partial magnification of the narrow gap (scale bar of (a) 10 µm, (b) 5 µm, and (c) 1 µm). To demonstrate the high-resolution fabrication of our technology, we patterned a simply shaped MSC to demonstrate the higher resolution of the electrode gaps. We patterned parallel strip-shaped MSCs with different narrow gaps so that the gap is a straight line and is clearer to see. As shown, the resolution of the slit is less than 500 nm, and close to 350 nm in some parts. Our resolution was realized through the pattern design of light field shaping, where the slit between design patterns was used to achieve the highest resolution but was not limited by the laser wavelength. This potentially explains how to complete a 3D reduction using the 3D shaped laser."

The original title is as following:

"Ultrafast laser-stamping for MSCs manufacturing"

The title is revised as following:

"Laser photonic-reduction stamping for graphene-based MSCs ultrafast fabrication"

The added part is inserted in Page 3, Paragraph 2, Line 5, the detail is as following:

"The initial Gaussian beam can be made to various beam shapes by using phase modulations, similar to a variable 3D "photonic stamp", which can pattern the MSCs with designable shapes and photoregulate the chemical reactions to synthesize LIG/MnO_2."

1.1.4 Reviewer's Comments

1.1.4 "A minor mistake is that the authors have to document how the gas releases from the reduced body. If they are trapped, a porous structure will be formed and how this porosity affects the energy storage performance."

Our Response

As indicated in our previous response regarding the mechanism underlying graphene reduction, the shaped femtosecond laser induces photochemical reduction and photothermal reduction during graphene reduction[R1.49,50]. When the shaped laser is used to irradiate GO film, electrons in the valence band absorb the energy of laser photons and jump into the conduction band. Owing to the action of freely moving electrons and holes, GO films undergo reduction. Femtosecond lasers offer numerous photons that can excite electrons to create free-moving electrons and holes. Through their action, GO is reduced to LIG. CO_2 and H_2O are released during photochemical GO reduction. The energy released from GO deoxygenation serves as an effective power source in situ, driving the oxidation reaction of Mn^{2+} to MnO_2. This reaction has been described in a schematic presented in Figure R1.22.

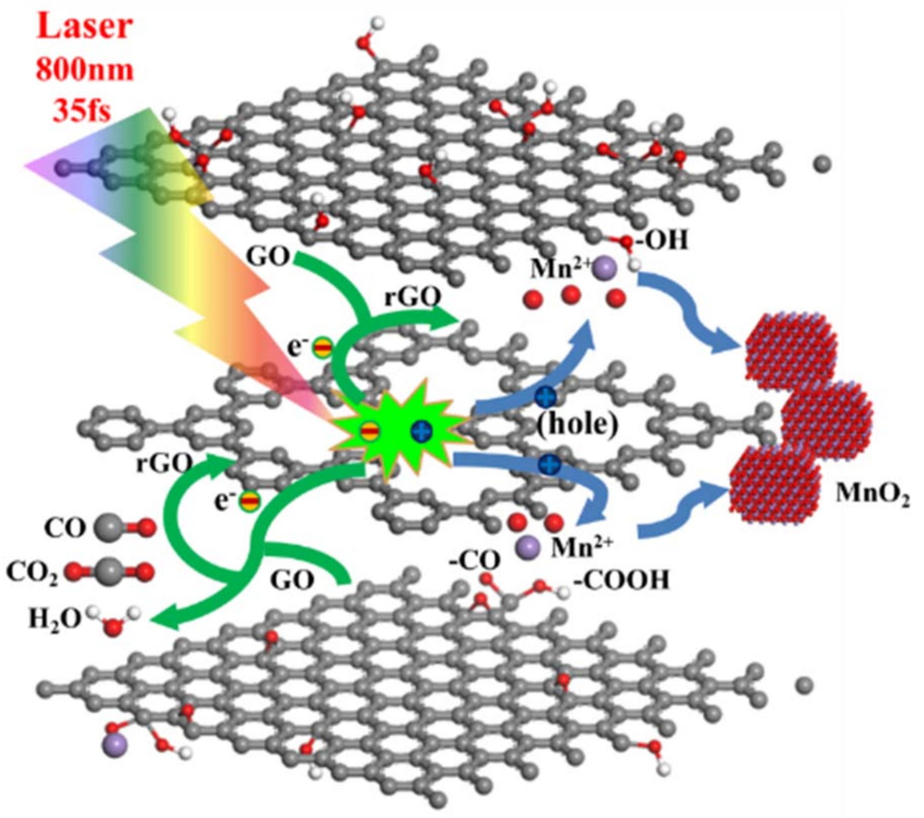

Figure R1.22 Schematic of the resultant morphology change and phase transformation by a femtosecond laser under the proposed photosynthetic routes

The equation can be expressed as follows

$$\text{(GO)-OH+(GO)-COOH+2e}^- \rightarrow \text{2rGO+CO}_2\uparrow\text{+H}_2\text{O}\uparrow \quad \text{(E1-10)}$$

$$\text{(GO)-O-(GO)+(GO)-COOH+ne}^- \rightarrow \text{3rGO+3CO}\uparrow \quad \text{(E1-11)}$$

A few free electrons are present in the air and absorbed by air's constituent gases. Of the gases comprising air, oxygen has the highest capacity for adsorbing free electrons. In the presence of these free electrons, GO films are also reduced. The primary equation can be expressed as follows:

$$\text{(GO)-OH+(GO)-COOH+2O}_2^- \rightarrow \text{2rGO+CO}_2\uparrow\text{+H}_2\text{O}\uparrow\text{+2O}_2\uparrow \quad \text{(E1-12)}$$

$$\text{(GO)-O-(GO)+(GO)-COOH+nO}_2^- \rightarrow \text{3rGO+3CO}\uparrow\text{+nO}_2\uparrow \quad \text{(E1-13)}$$

When laser power causes photothermal reduction, the temperature of the femtosecond laser is relatively low, and the cycloaddition reaction of oxygen bridges

(C–O–O) close to each other primarily occurs on the surface of the GO film, and O_2 is generated after GO reduction[R1.51].

$$GO \xrightarrow{\Delta} rGO + O_2 \uparrow \quad \text{(E1-14)}$$

If the power density of the laser continues to increase and the surface temperature of the GO film is high, the GO film undergoes a drastic cracking reaction at high temperature, wherein oxygen-containing functional groups including hydroxyls (-OH), carboxyls (-COOH), and oxygen bridges (C–O–C) are decomposed into CO, CO_2, and H_2O, being eliminated. Upon elimination of the oxygen-containing functional groups, GO is reduced.

$$GO \xrightarrow{\Delta} rGO + CO_2 \uparrow + CO \uparrow + H_2O \uparrow \quad \text{(E1-15)}$$

When the SSFL reduces the GO hybrid film, gas is released, which is a critical factor underlying the formation of 3D composite porous structures.

We explain the effect of the characteristics of the porous structure on energy storage performance from several perspectives as follows.

Owing to their high surface area, remarkable thermal conductivity, and excellent electronic conductivity and mechanical properties, porous materials have received increasing attention and their capacitance has been increased compared with solvated electrode materials, particularly at high current density. Porous structures are favorable for fast ion and electron transport and facilitate sufficient contact between electrolytes and porous materials[R1.52].

Pores facilitate rapid ion diffusion between graphene layers and increase the speed of ion transport across a compressed film. As indicated in Figure R1.23, the porous structure provides a rapid and numerous pathways for ion transfer, enabling rapid and complete interactions between ions and the electrode material, meaning that charging and discharging can occur more rapidly. This ability to optimize charge transport was reflected through Nyquist and Bode plots[R1.53]. In our study, the MSC displayed an ultraquick response (0.01 ms) and low equivalent series resistance (0.85 $m\Omega\ cm^{-2}$).

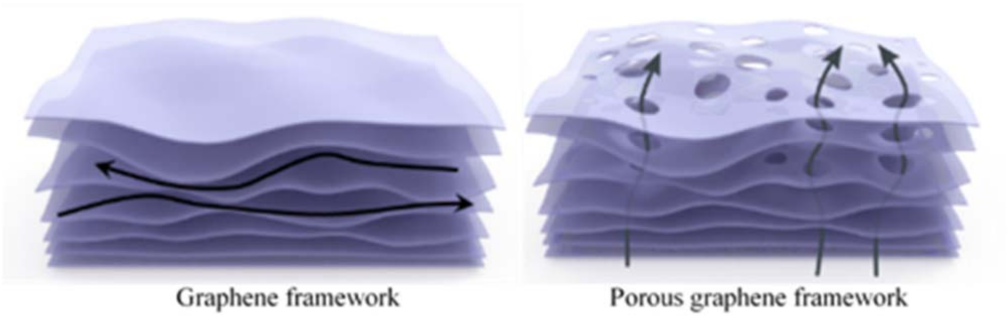

Figure R1.23 Illustration of a compressed graphene framework and porous graphene framework with arrows depicting the ion transport pathways

3D porous structures are attractive scaffolds for other electrode materials because of their excellent charge transport kinetics through 3D hybrid electrodes. Compared with 3D porous structure generation using certain metallic foams[R1.28-30], in situ synthesis of complex 3D porous structures yields more stable materials. Graphene and MnO_2 synthesized and strongly interacted through the action of the SSFL in this study, resulting in a complex that was unlikely to be cleaved. For instance, electrochemical analyses were performed using MSCs fabricated more than 30 days previously, and the capacitance performance was almost the same as that of newly prepared MSCs (Figure R1.24).

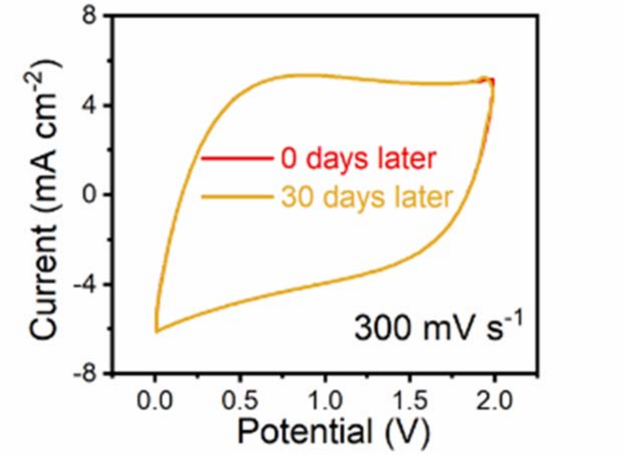

Figure R1.24 The CV curves of the fabricated MSCs after 0 and 30 days

As indicated in Figure R1.24, under the same scanning rate, the CV curves of the two supercapacitors almost coincided, indicating that the supercapacitor generated using the SSFL had highly stable capacitance characteristics. Figure R1.25 highlights the capacitance retention of the MSCs under different voltage windows. The inset of

Figure R1.25 shows five GCD curves of interdigital MSCs before and after 6,000 cycles.

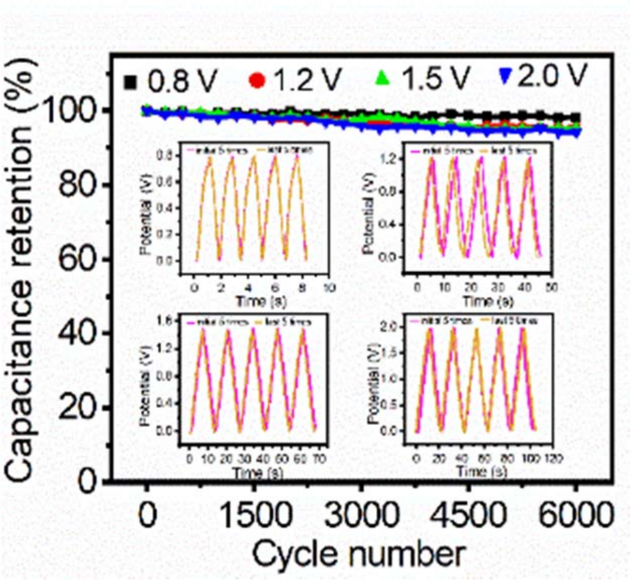

Figure R1.25 Cycle life of the MSCs under different voltage windows.

After 6,000 cycles, the GCD curve was still almost identical to the initial curve. When the voltage window was small, the capacitance retention was almost 100%, and when the voltage window was 2 V, the capacitance retention rate remained >93%, indicating that the MSCs had excellent cycling stability.

The obtained 3D porous scaffold is highly stable and can mitigate mechanical stress within the electrode, ensuring the long-term cycling stability of electrochemical energy storage systems[R1.54,55]. Our 3D structure is supported by the MnO_2 nanoparticles, which serve as nanospacers for the LIG network and provide adequate space for electrolyte ions to interact with the entire electroactive surface of the electrode, facilitating efficient charge storage. Not only does graphene serve as a support for the MnO_2 nanoparticles, it also interacts robustly with the MnO_2 nanoparticles, preventing their aggregation and resolving the graphene layer restacking issue, which enhances electron transport and stability during cycling[R1.56].

Modifications

According to the comments of the reviewer, we have revised the corresponding

part in our manuscript.

The original part form Page 9, Paragraph 1, Line 3 to Line 6 is as flowing:

"The SEM revealed the surface of the graphene film also became porous and fluffier after ss-fs laser ablation (Fig. 3c and d). The fluffy and wrinkled structure resulted in larger specific surface area, fast ion transport, and excellent electrochemical performance."

This part is revised as following:

"The 3D porous structure irradiated by SSFL could be clearly observed on the cross-sectional view and the surface of the hybrid film before and after the SSFL patterning (Fig. 2d). The original GO layers were stacked tightly. Notably, after SSFL laser ablation, the lamellar graphene fluffed up, and numerous MnO_2 nanoparticles were attached to LIG. The porous structure resulting from the gas release and the action of 3D light field in the reduction process has the advantages of high surface area, remarkable thermal conductivity, excellent electronic conductivity and and favorable mechanical properties. More importantly, these pores function as shortcuts for rapid ion diffusion between graphene layers and increase the speed of ion transport across the compressed film. The detailed analysis and schematic diagram are in the Supplementary Fig.5. The superiority of this special 3D porous structure can be fully demonstrated in the subsequent electrochemical performance tests."

The corresponding content added in Supporting Information is as following:

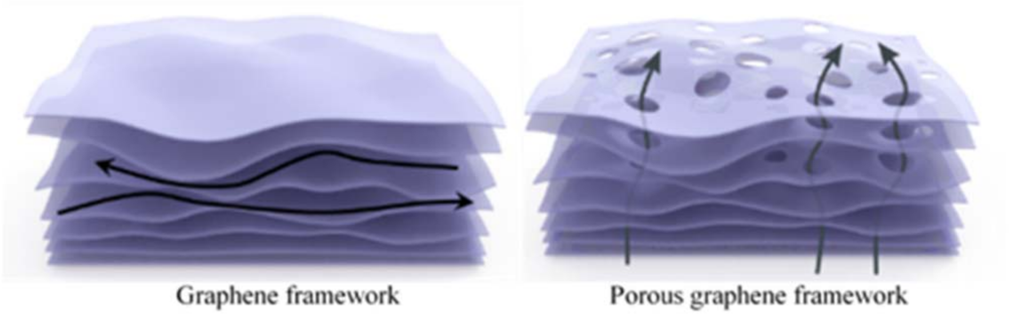

"Supplementary Figure 5: Illustration of a compressed graphene framework and porous graphene framework with arrows depicting the ion transport pathways. The porous structure provided a faster path and more path choices for the ion transfer.

This enabled the ions to contact the electrode material quickly and fully, thus charging and discharging more quickly. This ability to optimize charge transport is shown in Nyquist plots and Bode plots. Our MSCs exhibited an ultra-small time response (0.01 ms) and low equivalent series resistance (0.85 mΩ/cm^2)."

The added part is inserted in Page 11, Paragraph 1, Line 1, the detail is as following:

"Therefore, the SSFL reduction in the experiment was caused by the combined effect of photochemical and photothermal reactions. Our findings indicated that gas was released when the SSFL reduced GO composite film, which is crucial in the formation of 3D porous structure composites. The 3D porous patterned structure fabricated in one step using the SSFL employed graphene as a solid skeleton, and the uniform effect of the light field enabled manganese dioxide nanoparticles to be evenly distributed on the graphene skeleton. The pores functioned as shortcuts for rapid ion diffusion between graphene layers, which increased the speed of ion transport across the compressed film. The 3D porous scaffold was very stable, which would mitigate mechanical stress within the electrode and thus ensure the stability of long-term cycling of electrochemical energy storage systems[41,42]. Therefore, the LIG/MnO2 composite had improved capacitance."

The added part is inserted in Page 19, Paragraph 3, Line, 1 the detail is as following:

"Furthermore, our MSCs exhibited excellent performance in power density, reaching 136 W cm^{-3}. The electrochemical tests were carried on the MSC fabricated over 30 days and the capacitance performance is almost the same as the newly prepared MSC, which demonstrates the super stability of our MSCs (Supplementary Fig, 27). Furthermore, we obtained the CV curves of the same single."

The corresponding content added in Supporting Information is as following:

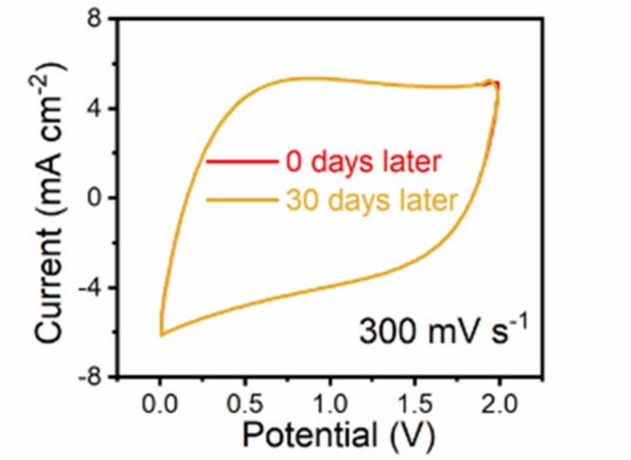

"Supplementary Figure 27: CV curves of the fabricated MSCs after 0 and 30 days. To verify the stability of our MSC, we conducted electrochemical tests on the miniature supercapacitor fabricated after 30 days. We found that it exhibited almost the same electrochemical performance as the initial MSC. As shown, under the same scanning rate the CV curves of the two supercapacitors almost coincided. This also indicated that the supercapacitor prepared using a shaped femtosecond laser has highly stable capacitance characteristics."

1.2 Reviewer's Comments

"1.2. Fig. 4 (a) displays that their capacitor is pretty worse even at 1V/s, a pretty low scanning rate. The unit of Fig. 4 (h) is ohm/cm2. Without the thickness a unit in ohm is better to display the internal resistance."

Our Response

We deeply appreciate the careful efforts and kind help of the reviewer in commenting on our paper. These detailed comments will be extremely valuable in making improvements.

We found that the parallel strip MSC exhibits poor performance at 1 V/s in the experiment. Our detailed explanation for this phenomenon is as follows.

Our technology can fabricate the one-step processing of MSCs of any shape. To further highlight the shape diversity and performance uniformity of our MSCs, we constructed several shape-designable MSCs; namely concentric circle, parallel strip,

and interdigital. We then recorded the true CV curves of these MSCs at the different scan rates shown in Figure 4.

Regrettably, we placed the worst-performing parallel strip MSC at the beginning of Figure 4, which may have led the reviewer to misunderstand the performance. In fact, after comparing the experimental results, we found that the performances of the concentric circle (Figure 4b) and interdigital (Figure 4c) MSCs exceeded those of the parallel strip (Figure 4a) MSCs. The purpose of Figure 4a is provide a true reflection of the performance of the parallel strip MSC by showing that it is much poorer than that shown in Figures 4b and 4c. It is this difference that makes our research on the shape design of MSCs meaningful. From this, we conclude that the shape of the MSC does have an effect on performance. For a more intuitive representation, Figure R1.26 compares the CV curves of the versatile-shaped MSCs at 1V/s.

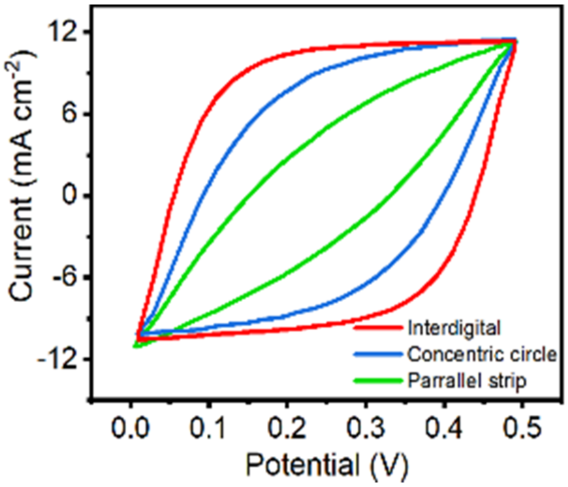

Figure R1.26 CV curves of versatile-shaped MSCs obtained at 1 V s^{-1}

We concluded that our MSC was micron-scale and that efficient ion and charge transfer between electrode materials and electrolyte solutions was extremely important. Interdigital MSCs use the electrode material area more efficiently and increase the contact area between the electrode material and the electrolyte. Because of their short diffusion distances, electrolyte ions are placed in the narrow gap between electrodes, which makes it easy to transport and results in higher performance[R1.57]. The interdigital MSCs are interlaced with electrode materials; narrowing the width of the fingers in these MSCs can shorten the ion diffusion

pathway and increase the length of the interface between the active-material electrode and the electrolyte. This contributes to the improved double layer storage and enhanced rate capability of MSCs.[R1.58] The parallel strip MSCs fabricated by our technology have a simple structure, a small effective contact area between the active electrode material and the electrolyte, and a few transfer paths between ions. This disadvantage is more obvious in three-dimensional electrode materials. Consequently, our parallel strip MSCs have the worst capacitance of the three MSCs.

The resolution of our MSCs can reach micro and nano levels, and the charging and discharging speeds are especially fast under the high-speed scanning rate[R1.59]. The low performance of the parallel strip MSC at 1 V/s is due to insufficient contact between the electrode material and the dielectric during the rapid charging and discharging process. Capacitance retention can explain the influence of the scan rate on electrochemical performance. When the scan speed increased 20 times from 50 to 1000 mV/s (Figure R1.27), the capacitance retention of interdigital MSCs reached 75.5%—higher than that of both the concentric circle (65%) and parallel strip (55.4%) MSCs. Thus, the interdigital MSCs exhibit the best capacitance retention. This is consistent with the previous finding that shape diversity can affect the performance uniformity of MSCs.

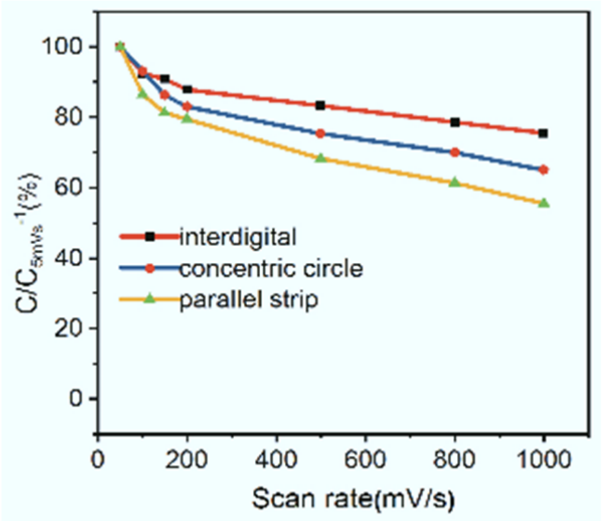

Figure R1.27 The capacitance retention of the L-S-MSCs of different geometries

The equivalent series resistance, which is a measure of the total resistance of a system, is determined from the Nyquist plots. As mentioned in our previous response, the thickness of the MSC is 3 μm. Because preparation of the area of electrode materials is extremely small (a few dozen microns) for a miniature supercapacitor, the measured current in the electrochemical test is also extremely small. We therefore frequently use mA/cm^2 and $\Omega\ cm^2$ in the original manuscript.

To improve the quality of the article, we follow the reviewer's opinion and use ohms to display the internal resistance (Figure R1.28).

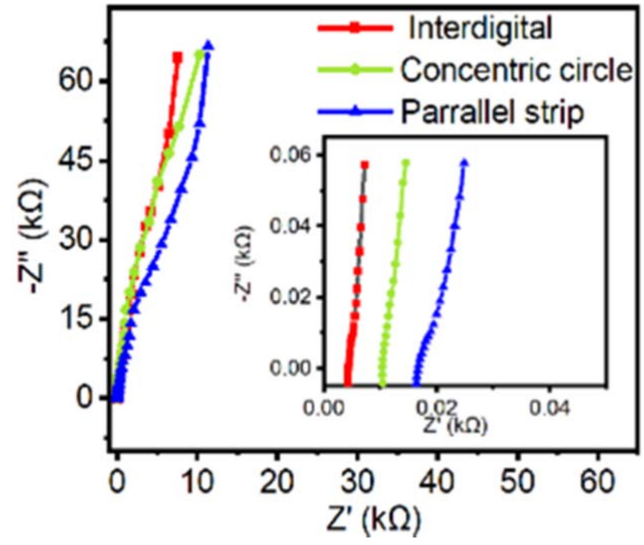

Figure R1.28 Nyquist plots of the versatile-shaped MSCs

Modifications

Based on the comments of the reviewer, we have revised the corresponding section in our manuscript. The original section on Page 15, Figures 4a to 4c, is as follows:

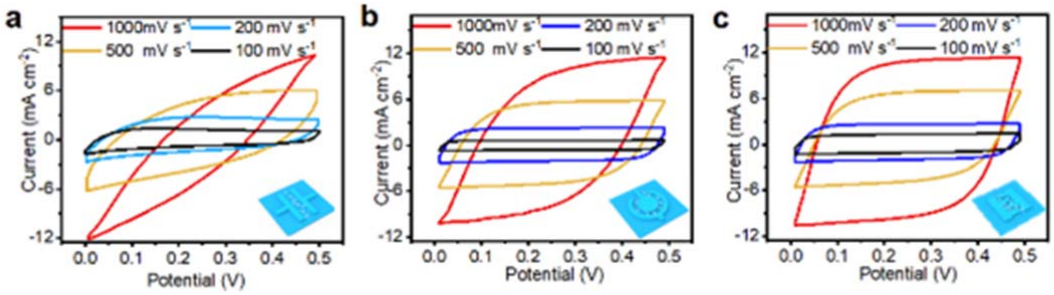

Fig. 4 a-c, CV curves of parallel strip (a), concentric circle (b), and interdigital (c) L-S-MSCs at the scan rate from 100 to 1000 mV s^{-1} in 0.5 M Na_2SO_4.

This part is revised as following:

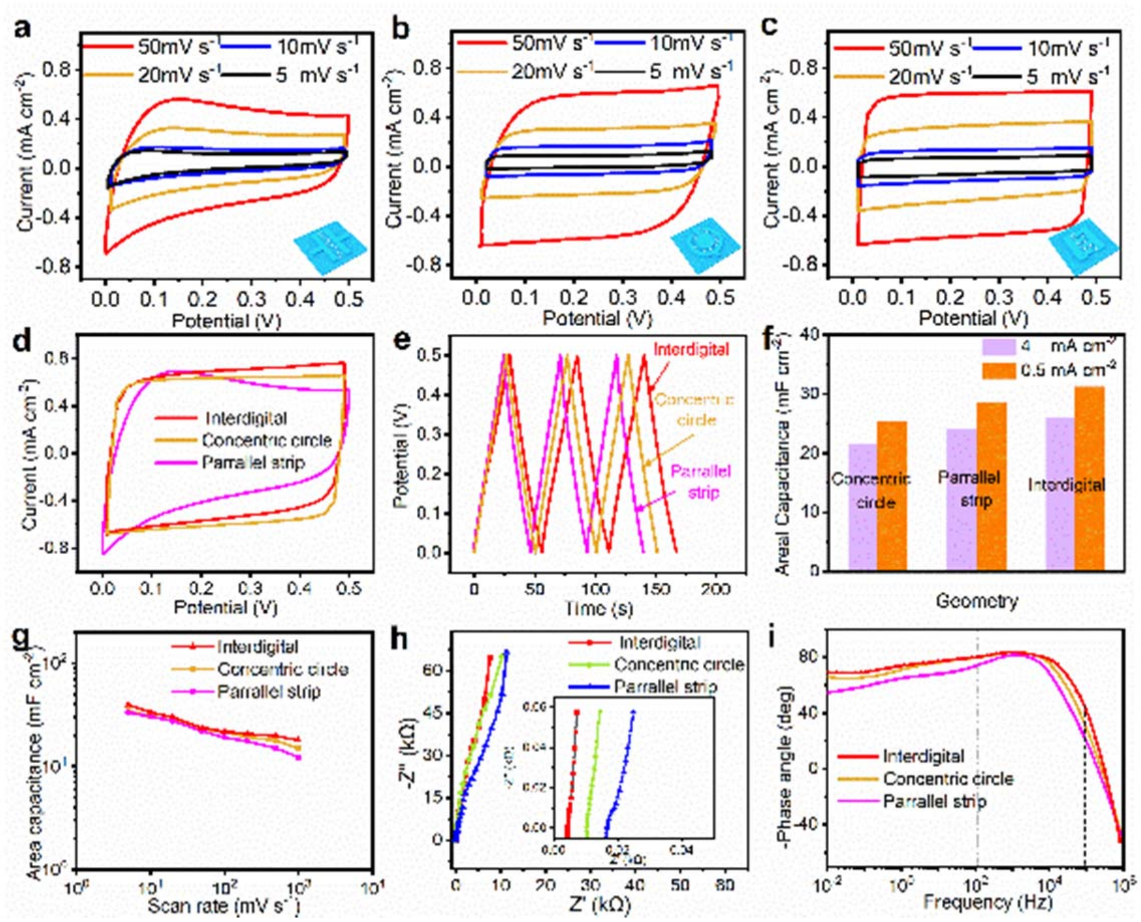

Fig. 4 a-c, CV curves of parallel strip (a), concentric circle (b), and interdigital (c) L-S-MSCs at the scan rate from 5 to 50 mV s^{-1} in 0.5 M Na_2SO_4.

The original part form Page 14, Paragraph 1, Line 12 to Line 13 is as flowing:

“We concluded that our L-S-MSCs were probably micron-scale and that efficient ion and charge transfer between electrode materials and electrolyte solutions was more important. Interdigital L-S-MSCs can use electrode material area more efficiently and increase the contact area between electrode material and electrolyte; due to the short diffusion distance of ions, electrolyte ions are placed in the narrow gap between electrodes, which makes it easy to transport and results in higher performance[48].”

This part is revised as following:

"The interdigital MSC exhibited the optimal gravimetric capacitance; however, the parallel strip MSC exhibited excellent gravimetric capacitance, higher than the gravimetric capacitance of the concentric circle MSC (Supplementary Fig). This finding indicates that the shape design of MSCs affected performance. In interdigital MSCs, electrode material areas are used more efficiently and the contact area between the electrode material and electrolytes is greater. Furthermore, interdigital MSCs are interlaced with electrode materials that can shorten the ion diffusion pathway by narrowing the width of the fingers in the MSC and increasing the length of the interface between the active-material electrode and the electrolyte. Therefore, appropriately designing the shapes of an MSC is conducive to fast ion transfer rate, rapid charge and discharge, improved double layer storage, and enhanced rate capability[53]."

According to the comments of the reviewer, we used a unit in ohm to display the internal resistance.

The original part form Page 15, Figure 4 h is as flowing:

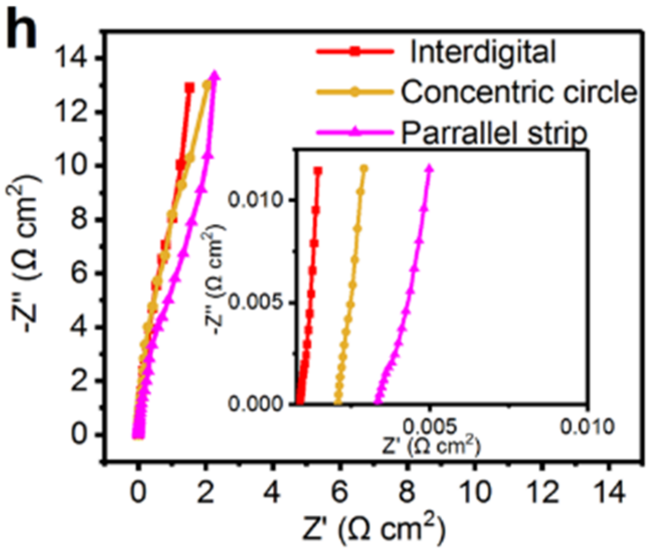

Fig 4 h, Nyquist plots of the versatile-shaped L-S-MSCs

This part is revised as following:

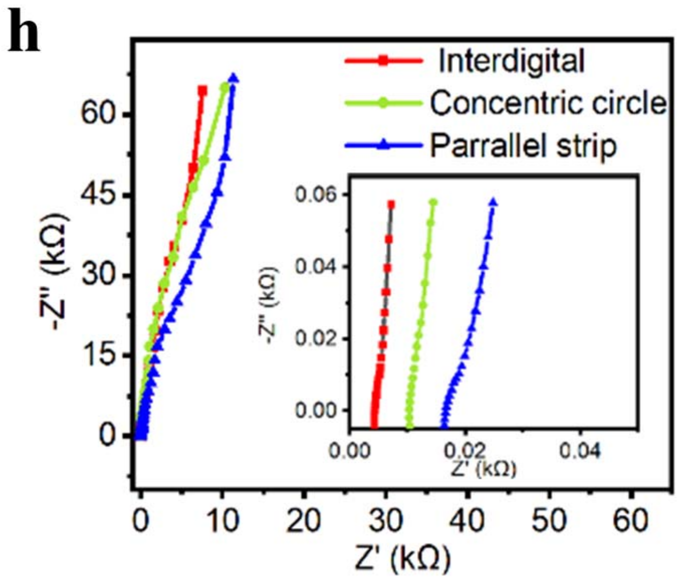

Fig 4 h, Nyquist plots of the versatile-shaped MSCs

1.3 Reviewer's Comments

"1.3. Fig. 5 (a) is not properly presented. The discharge time of only 60 s at a 4 mA/cm2 is alos display the capacitance is very limited."

Our Response

We thank the reviewer for their valuable advice, which has been extremely important in improving the quality of the paper.

In our work, the current density of 4 mA/cm^2 is equal to 1.33×10^4 mA/cm^3 or 2857 mA/g, which is relatively high for a miniature thin supercapacitor. Because our MSCs are tens of microns in size and their narrow gap is minute, they can be charged and discharged rapidly at this density. The miniature supercapacitor has its own unique advantages; for example, it can be perfectly matched to numerous niche applications. Although the area and volumetric capacity is higher than that of other supercapacitors, the total amount of capacitance stored is limited due to the small size and area required for miniaturization. Therefore, in the GCD profiles, the charge and

discharge times are shorter than for ordinary supercapacitors. We also investigated a large number of studies[R1.60-68] on miniature supercapacitors and found the same results.

The charge and discharge times increase when we reduce the current density, reaching approximately 450 s at 1 mA/cm^2, which demonstrates the excellent performance of our MSCs (Figure R1.29 (a)).

In Figure 5a, we focus on the expansion effect of the voltage window and neglect the short charge and discharge time. We therefore retested the galvanostatic charge–discharge curve of the MSC at a current density of 2 mA/cm^2 and carried out a similar voltage window expansion experiment (Figure R1.29 (b)). The galvanostatic charge–discharge curves of the MSC form triangular shapes at an increasing voltage window from 0.8 V to 2.0 V, and the discharge time of 250 s at a current density of 2 mA/cm^2 displays the capacitance of the MSCs.

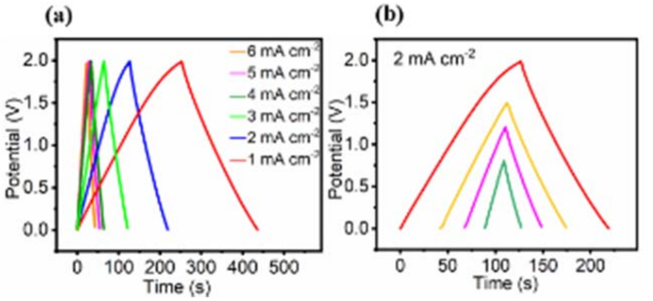

Figure R1.29 (a) The GCD profiles of MSC at current density from 1 to 6 mA cm^{-2} under 2 V voltage window (b) GCD profiles of MSC with operating voltages from 0.8 to 2 V at 2 mA cm^{-2}

Modifications

Based on the comments of the reviewer, we have revised the corresponding section in our manuscript. The original section from Page 17, Figure 5 a is as follows:

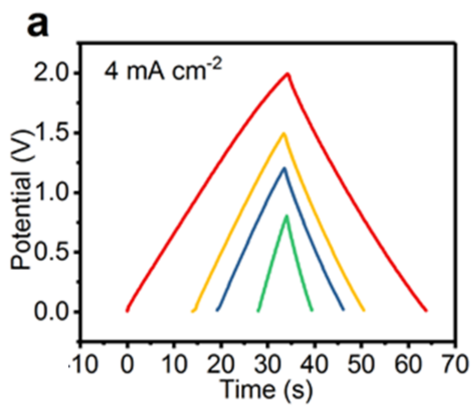

Fig 5 a, GCD profiles of LIG/MnO_2 L-S-MSCs in 0.5 M Na_2SO_4 with different operating voltages from 0.8 to 2 V at 4 mA cm^{-2}.

This part is revised as following:

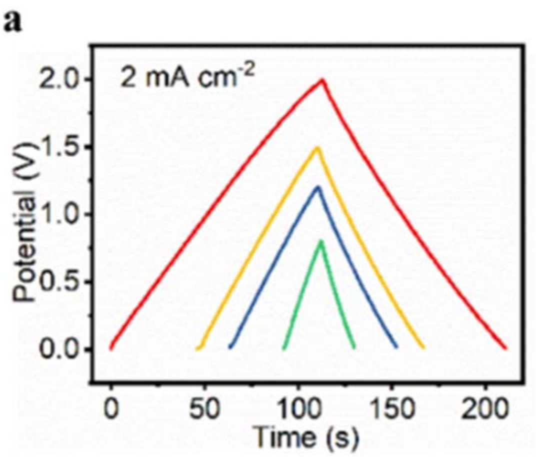

Fig 5 a, GCD profiles of LIG/MnO_2 MSCs in 0.5 M Na_2SO_4 with different operating voltages from 0.8 to 2 V at 2 mA cm^{-2}.

The original part form Page 16, the last Paragraph, line 5 is as flowing:

"GCD curves at 80mV s^{-1} and 4 mA cm^{-2}"

This part is revised as following:

"GCD curves at 80mV s^{-1} and 2 mA cm^{-2}"

1.4 Reviewer's Comments

"1. 4.There is nothing new from Raman/XPS/TEM in Fig. 3."

Our Response

We are grateful for your valuable advice which has inspired us to explore further the mechanism of laser reduction of graphene and manganese oxide materials. The purpose of our experiment was to utilize the shaped femtosecond laser to reduce the GO in-situ while simultaneously synthesizing the manganese dioxide complex. The main purpose of our Raman and XPS tests was to demonstrate the reduction effect of GO and the generation of manganese dioxide products. Notably, the nanoparticles of MnO_2 are large-scale and uniformly distributed on the graphene sheet layer from the TEM.

Through detailed analysis using XPS and XRD, we found that the shaped femtosecond laser can regulate the synthesis of materials and control photoinduced and photothermal-induced reduction/oxidation and the valence state of manganese. Altering the laser fluence can also yield heterogeneous junctions of different compositions. To demonstrate that femtosecond laser irradiation can be used to control the synthesis of manganese oxide, we performed XPS measurements to investigate the manganese oxide and graphene composites. We defined the composite materials synthesized under different laser fluences as Laser-130, 170, 210, 250 and 290 LIG/Manganese oxide, corresponding to laser fluences of 130, 170, 210, 250, and 290 mJ/cm^2, respectively.

Figure R1.30 displays the high-resolution XPS spectra of Mn 2p spectra for Laser-130, 170, 210, 250 and 290 LIG/Manganese oxide. Because the duration of the ultrashort pulse was smaller than the time scale of most of the physical and chemical characteristics, this resulted in chemical reaction pathways; chemically selective molecular excitation can be effectively controlled by a femtosecond laser. The Mn 2p XPS spectrum exhibited three characteristic peaks at 641.2, 642.5, and 646 eV, corresponding to spin–orbit peaks of manganese with mixed valence states of +3, +4, and +2, respectively.

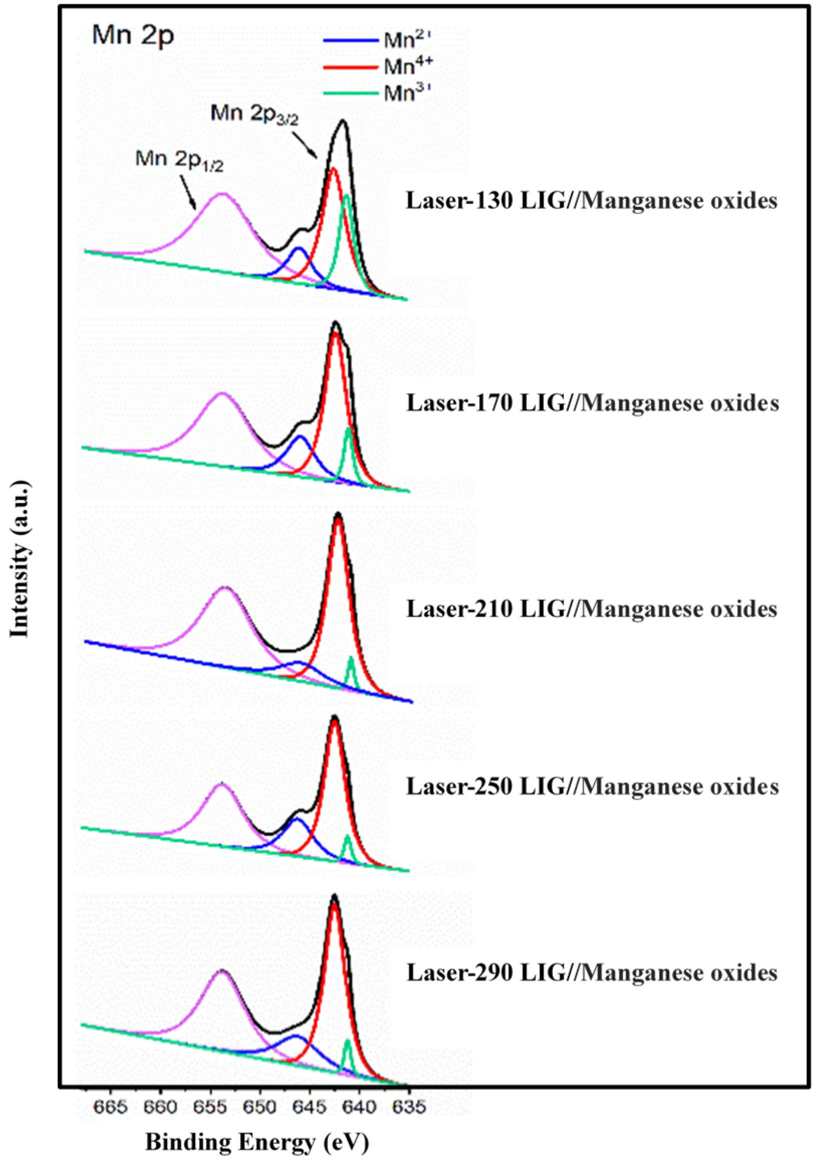

Figure R1.30 XPS spectra of Mn 2p for Laser-130 LIG/Manganese oxide, Laser-170LIG/Manganese oxide, Laser-210 LIG/Manganese oxide, Laser-250 LIG/Manganese oxide, Laser-290LIG/Manganese oxide

When the fluence of the laser increased to 130 mJ/cm^2, the percentage of Mn^{4+} in the LIG/Manganese oxides exceeded 50%. The percentage of Mn^{3+} in the LIG/Manganese oxides materials remained high when the percentage of Mn^{2+} was at its lowest. As the fluence of the laser gradually increases, the percentage of manganese in different valence states changes.

TableR1.3 Summary of the XPS data for the LIG/Manganese oxides induced by different laser fluence			
Material	Mn^{4+}2p [eV]	Mn^{2+} 2p [eV]	Mn^{3+}2p [eV]
Laser-130LIG/Manganese oxides	642.4(53.9%)	646.0(15.5%)	641.1(30.6%)
Laser-170LIG/Manganese oxides	642.6(66.4%)	646.1(23.7%)	641.2(9.9%)
Laser-210LIG/Manganese oxides	642.5**(79.4%)**	646.0**(16.6%)**	641.1**(4.0%)**
Laser-250LIG/Manganese oxides	642.6(69.1%)	646.2(26.7%)	641.2(4.2%)
Laser-290LIG/Manganese oxides	642.6(71.4%)	646.2(24.2%)	641.2(4.2%)

Table R1.3 indicates that the characteristic peaks and the percentage of Mn^{2+}, Mn^{3+} and Mn^{4+}. The Mn 2p spectra of LIG/Manganese oxides photoinduced by lasers with different fluences indicate that the electron BE assigned to Mn^{2+}, Mn^{3+} and Mn^{4+} 2p is close to 646.0 eV, 641.2 eV, and 642.5 eV, respectively. The percentages of Mn^{4+} and Mn^{3+} were the highest (79.4%) and lowest (4%) when the laser fluence was 210 mJ/cm^2. Conversely, the percentage of Mn^{2+} ions decreased from 23.7% to 16.6% and then increased from 16.6% to 26.7% as the laser fluence increased. Altering the laser fluence thus enables various material compositions of LIG/Manganese oxides to be successfully synthesized through photomodulation of the reaction mechanisms. The percentage of manganese in different valence states can be adjusted flexibly.

Mn 3s is more sensitive to the average oxidation state of manganese than Mn 2p is. The energy separation between the two peaks (ΔE) is closely related to the mean manganese oxidation state. The Mn 3s XPS spectra (Fig. R1.31) reveals that the ΔE values of aLaser-130LIG/Manganese oxide, Laser-170LIG/Manganese oxide, Laser-210 LIG/Manganese oxide, Laser-250LIG/Manganese oxide, and Laser-290LIG/Manganese oxide are 5.38, 5.25, 4.75, 4.88, and 5.0 eV, respectively The position of the lower binding energy peak gradually shifts to higher energy as the oxidation number of the sample increases, which is consistent with previous results. The standard ΔE values for MnO_2, Mn_2O_3, and MnO are 4.7, 5.2, and 5.8eV, respectively. When the laser fluence was 210 mJ/cm^2, ΔE was 4.75, which is

extremely close to the MnO_2 value.

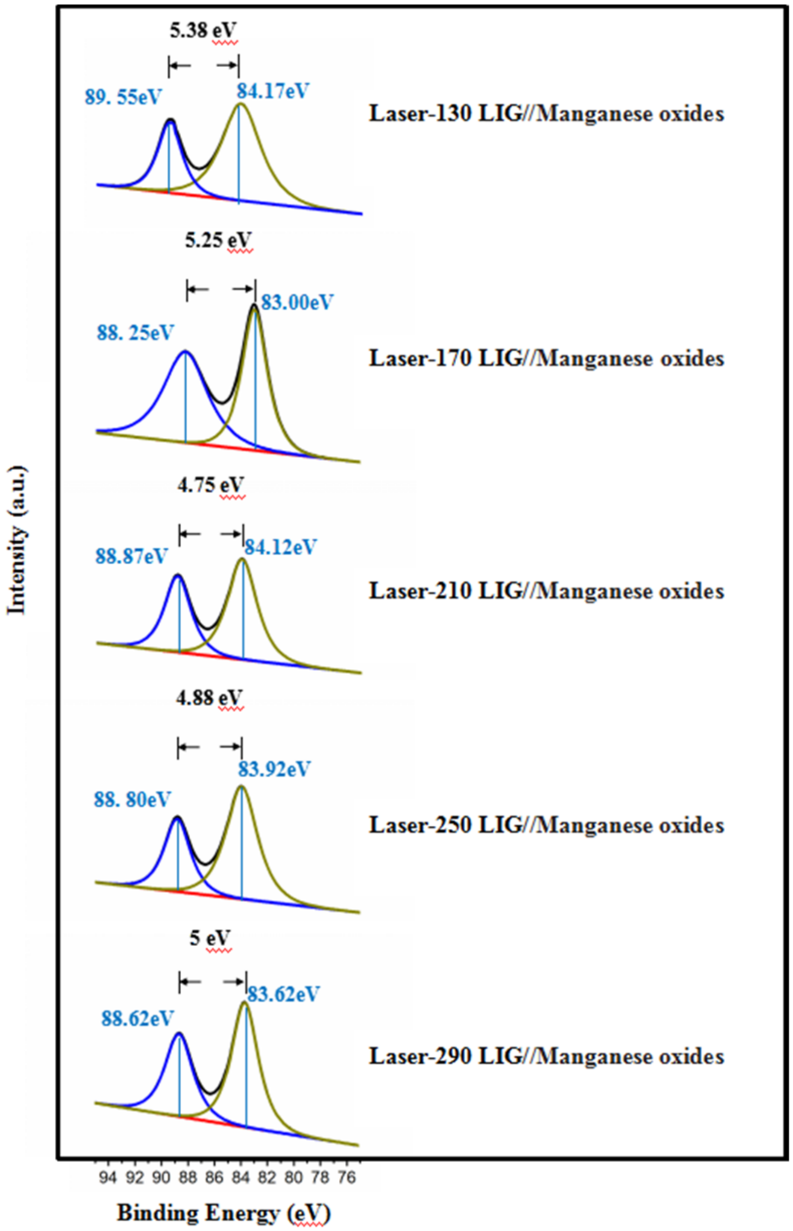

Figure R1.31 XPS spectra of Mn 3s forLaser-130 LIG/Manganese oxide, Laser-170 LIG/Manganese oxide, Laser-210 LIG/Manganese oxide, Laser-250 LIG/Manganese oxide, Laser-290LIG/Manganese oxide

As the laser fluence increases or decreases, the value of ΔE increases, which indicates the presence of manganese oxides of lower-valence states. There are typically defects in the interlayers, such as vacancies and Mn^{4+} substitution by $Mn^{3+}$and Mn^{2+}. This result is consistent with those discussed earlier.

In this study, by altering the pulse energy, materials with various compositions of LIG/Manganese oxides were successfully synthesized through photomodulation of the reaction mechanisms. By using a spatially shaped femtosecond laser, the ratio of the Mn^{4+} could be maximized, and MnO_2 as the main component is in the LIG/Manganese oxides.

Since the process of laser-regulated material synthesis is very complex, we should make a comprehensive study and exploration of laser-induced products. Therefore, XRD tests were added to characterize the composites irradiated by femtosecond laser, so as to verify the existence of the composites, especially manganese, and observe whether there are new phenomena. The XRD patterns of the as-prepared GO and LIG-MnO_2 nanocomposite crystal structure were analyzed and are shown in figure R 1.32. According to Fig. R 1.32 (a), GO has an intense peak at $2\theta = 11.2°$ that is related to the (001) diffraction. The diffraction peaks of Laser-210 LIG/Manganese oxide appeared at 2θ = 12.73°, 18.14°, 28.82°, 37.46°, 41.95°, 49.86°, 56.32°, 60.27°, 65.11°, 69.76° and 72.73° as shown in Figure R1.32 (b), which correspond to the expected diffraction peaks of (110), (200), (310), (211), (301), (411), (600), (521), (002), (541) and (312) crystal planes of α-MnO_2 standard data, respectively, following the JCPDS card PDF file no. 44-0141. No peaks for other types of MnO_2 are observed, indicating that the as-prepared products composed only of one tetragonal α-MnO_2 phase.

The diffraction peaks of Laser-210 LIG/Manganese oxide are very similar to those of a nanotetragonal phase of α-MnO_2, where the (001) reflection peak of layered GO has almost disappeared. Our results correlate well with the previous studies that the diffraction peaks become weakened or even disappear whenever the regular stacks of GO are exfoliated.

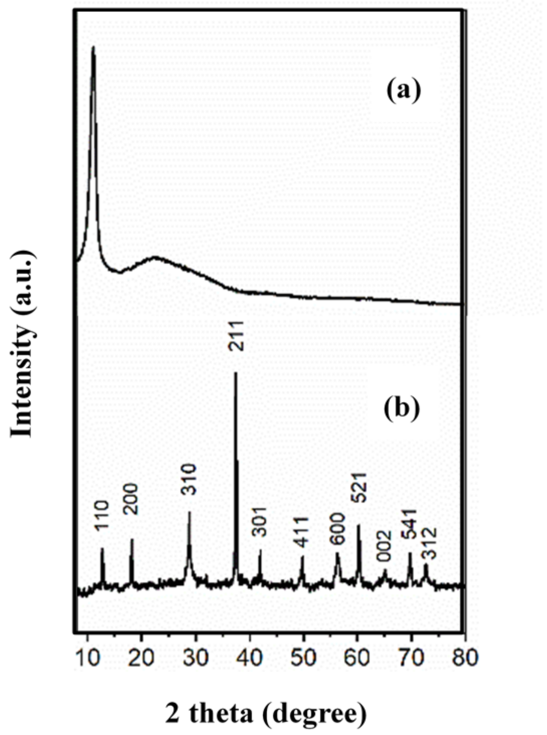

Figure R1.32 (a) XRD patterns of GO and (b) Laser-210 LIG/Manganese oxide

To gain some insights into the structures of LIG/ Manganese oxides, we collected XRD patterns at different composites (Laser-130LIG/Manganese oxides, Laser-170LIG/Manganese oxide, Laser-210 LIG/Manganese oxide, Laser-250LIG/Manganese oxide, Laser-290LIG/Manganese oxide.) processed under different laser fluence (Figure R1.33).

The XRD patterns of the LIG/Manganese oxides LIG showed peaks that were similar, which can be well indexed to plane of the tetragonal α-MnO_2 structure. However, we can still find differences through careful observation. The most obvious one is that the peak intensities of the XRD patterns of Laser-210 LIG/Manganese oxide and Laser-250 LIG/Manganese oxide are stronger than others'.

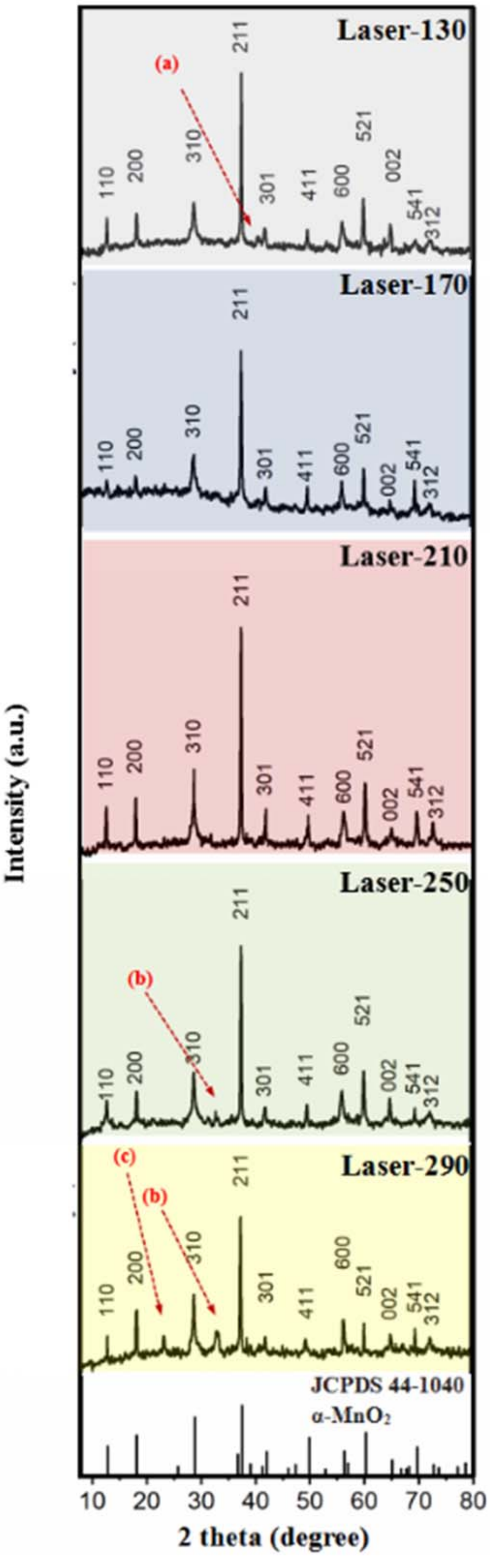

Figure R 1.33 XRD patterns of LIG/Manganese oxides processed under different femtosecond laser fluence

By altering the laser fluence, the diffraction peaks of LIG/Manganese oxides correspond to the expected diffraction peaks of the (110), (200), (310), (211), (301), (411), (600), (521), (002), (541) and (312) crystal planes of α-MnO_2 standard data. However, in the XRD pattern of Laser-130 LIG/Manganese oxide, a peak (a) appeared at 2θ = 40.5° as shown in the figure. This corresponds to the expected diffraction peaks of the (200) crystal plane of MnO standard data, following the JCPDS card PDF file no. 74-4748. This is lower than other peaks, and gradually disappears as the laser fluence increases. When the laser fluences are 250 and 290 mJ/cm^2, peaks (b) and (c) appear at 2θ = 33.05° and 23.12°, respectively. These were assigned to the (222) and (211) crystal planes of α-Mn_2O_3, following the JCPDS card PDF file no. 41-1442. The resulting diffractogram concludes the formation of α-MnO_2 (major) and α-Mn_2O_3 (minor) phases. This may be due to the conversion of MnO_2 to Mn_2O_3 as the laser fluence increases. MnO_2 continues to constitute an extremely high proportion of our laser-induced synthesis materials, especially when the laser fluence is 210 mJ/cm^2.

Thus, the femtosecond laser reduces GO, simultaneously facilitating the action of metal ions, which is a novel mechanism of action. Consequently, GO reduction and MnO_2 synthesis are mutually reinforcing processes in shaped femtosecond laser radiation. By altering the pulse energy, materials of various LIG/manganese oxide compositions were successfully synthesized through photomodulation of reaction mechanisms. By using an SSFL, the Mn^{4+} percentage could be maximized, and MnO_2 is the primary component of LIG/manganese oxide.

In our work, through the detailed analysis of XPS and XRD, we found that the shaped femtosecond laser can regulate the synthesis of materials, control photoinduced and/or photothermal-induced reduction/oxidation and the valence state of manganese. Altering the laser fluence also can obtain the heterogeneous junctions of different compositions.

Modifications

The added part is inserted following Page 9, Paragraph 1, Line 1 is as following:

"During the SSFL reduction process, the effect of photothermal

reduction/oxidation became increasingly apparent as the laser fluence was increased. By altering the laser fluence, LIG/Manganese oxides were successfully synthesized through photomodulation of the reaction mechanisms (photochemical and photothermal reduction/oxidation). We varied the laser fluence (170-290 mJ cm^{-2}) of the SSFL to investigate differences in conductivity and electrochemical characterization and identify the optimal reduction/oxidation effect. The resistance and conductivity of the material reached their minimum and maximum, respectively, when the laser fluence was 210 mJ cm^{-2} (Supplementary Fig. 7 and 8), which implies that the LIG/Manganese oxides composite has large potential capacitance[43]. As expected, the measured area-specific capacitance of the LIG/MnO2 MSC was highest under the laser fluence of 210 mJ cm^{-2} (Supplementary Fig. 9). We investigated whether the high-performance electrode materials were successfully synthesized under the interaction of photochemical and photothermal reduction/oxidation at this laser fluence.

The X-ray diffraction (XRD) patterns of the as-prepared GO and LIG/MnO_2 nanocomposite synthesized under the laser fluence of 210 mJ cm^{-2} were analyzed (Fig. 3b). The most intensive peak of GO at $2\theta = 11.2°$ corresponds to the (001) reflection. The diffraction peaks of LIG/Manganese oxides appeared at 2θ = 12.73°, 18.14°, 28.82°, 37.46°, 41.95°, 49.86°, 56.32°, 60.27°, 65.11°, 69.76° and 72.73° , which correspond to the expected diffraction peaks of (110), (200), (310), (211), (301), (411), (600), (521), (002), (541) and (312) crystal planes of α-MnO_2 standard data, respectively, following the Joint Committee on Powder Diffraction Standards card PDF file no. 44-0141. We then collected XRD patterns of different composites (LIG/MnO_2) synthesized under different laser fluence (Supplementary Fig. 10). The different XRD patterns exhibited similar peaks, which could be all well indexed to plane of the α-MnO_2 structure. However, when the laser fluence was considerably lower or higher than 210 mJ cm^{-2}, one or two other weak peaks appeared, and the peak intensities in the XRD patterns were lower.

The Raman spectra were extremely similar when the laser fluence was between 170 and 290 mJ cm^{-2} (Supplementary Fig. 11). We compared the Raman spectra in

three cases-untreated, fs-laser modified, and fs-laser reduced-on the basis of the laser fluence (Fig. 3c). Clear G bands were characteristics of sp^2 hybridized C–C bonds at 1580 cm^{-1}. D bands of residual oxygen functional groups and other defects[44] in the graphite layer were detected at 1350 cm^{-1}, and the high intensity of G peak demonstrated a high C–C bond ratio, confirming the formation of new graphite domains. The peak value at 641 cm^{-1} was caused by Mn-O vibrations perpendicular to the direction of the MnO_6 octahedral double chains of MnO_2, indicating the presence of MnO_2 in the mixed electrode material[45].

The added part is inserted following Page 9, Paragraph 2, Line 6 is as following:

"X-ray photoelectron spectroscopy (XPS) was used to assess the difference before (Supplementary Fig.12) versus after SSFL ablation under a laser fluence of 210 mJ cm^{-2} (Fig. 3d-f). The C 1s spectrum consisted of three peaks: C–C/C=C (284.8 eV), C–O (286.7 eV), and C=O (288.8 eV). After SSFL ablation, the C–O and C=O bonds of LIG/MnO_2 decreased, indicating that certain oxygen-containing groups on the GO hybrid film were removed during the reduction. The O 1s and Mn 2p spectrum further confirmed the presence of MnO_2. Fig. 3g illustrates the peaks centered at 530.9 and 532.3 eV, which correspond to the O–Mn and O–C bonds, respectively[46]. The peaks centered at 653.8 eV could be assigned to the Mn $2p_{1/2}$, and Mn $2p_{3/2}$ peaks were observed at three peaks: 641.2, 642.5, and 646, corresponding to +3, +4 and +2 Mn cations, respectively[47] (Fig. 3f). We also obtained high resolution Mn 2p spectra of LIG/MnO_2 under various laser fluence (Supplementary Fig 13). Supplementary Table presents the characteristic peaks and corresponding percentages of Mn^{2+}, Mn^{3+} and Mn^{4+}. The percentage of the Mn^{4+} and Mn^{3+} are the highest (79.4%) and lowest (4%) when the laser fluence was 210 mJ cm^{-2}. The percentage of manganese in different valence states could also be adjusted by altering the laser fluence. Mn 3s is more sensitive to the average oxidation state of manganese than Mn 2p was. The energy separation between the two peaks (ΔE) was closely related to the mean manganese oxidation state[48,49]. The Mn 3s XPS spectra (Supplementary Fig. 14) were used to assess the ΔE of the LIG/MnO_2 synthesized under various laser fluence. When the laser fluence is 210 mJ cm^{-2}, the ΔE is 4.75, which is very close to the value of

MnO_2."

The corresponding content in Supporting Information is as following:

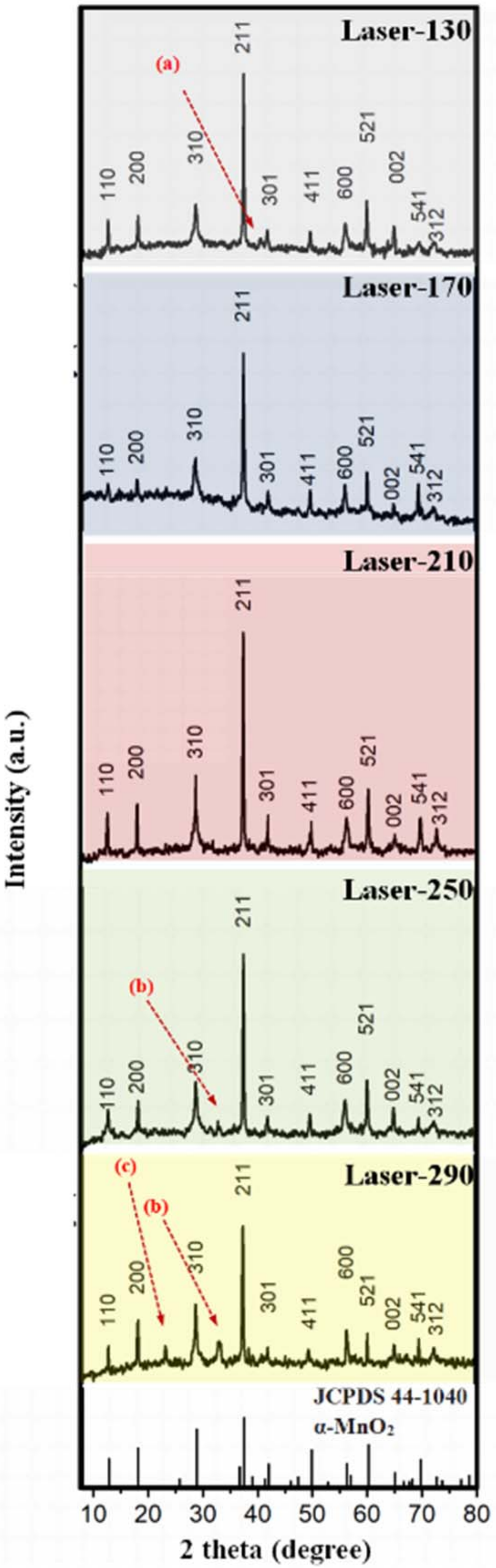

Laser-130
(a)
211
110
200
310
301
411
600
521
002
541
312
Laser-170
Laser-210
Laser-250
(b)
Laser-290
(c)
JCPDS 44-1040
α-MnO2
Intensity (a.u.)
10 20 30 40 50 60 70 80
2 theta (degree)

Supplementary Figure 10: XRD patterns of LIG/Manganese oxides processed under different femtosecond laser fluences. We defined the composite materials synthesized under different laser fluences as Laser-130, 170, 210, 250 and 290 LIG/Manganese oxide, corresponding to laser fluences of 130, 170, 210, 250, and 290 mJ/cm^2, respectively. By altering the laser fluence, the diffraction peaks of LIG/Manganese oxides correspond to the expected diffraction peaks of the (110), (200), (310), (211), (301), (411), (600), (521), (002), (541) and (312) crystal planes of α-MnO_2 standard data. However, in the XRD pattern of Laser-130 LIG/Manganese oxide, a peak (a) appeared at $2\theta = 40.5°$ as shown in the figure. This corresponds to the expected diffraction peaks of the (200) crystal plane of MnO standard data, following the JCPDS card PDF file no. 74-4748. This is lower than other peaks, and gradually disappears as the laser fluence increases. When the laser fluences are 250 and 290 mJ/cm^2, peaks (b) and (c) appear at $2\theta = 33.05°$ and 23.12°, respectively. These were assigned to the (222) and (211) crystal planes of α-Mn_2O_3, following the JCPDS card PDF file no. 41-1442. The resulting diffractogram concludes the formation of α-MnO_2 (major) and α-Mn_2O_3 (minor) phases. This may be due to the conversion of MnO_2 to Mn_2O_3 as the laser fluence increases. MnO_2 continues to constitute an extremely high proportion of our laser-induced synthesis materials, especially when the laser fluence is 210 mJ/cm^2.

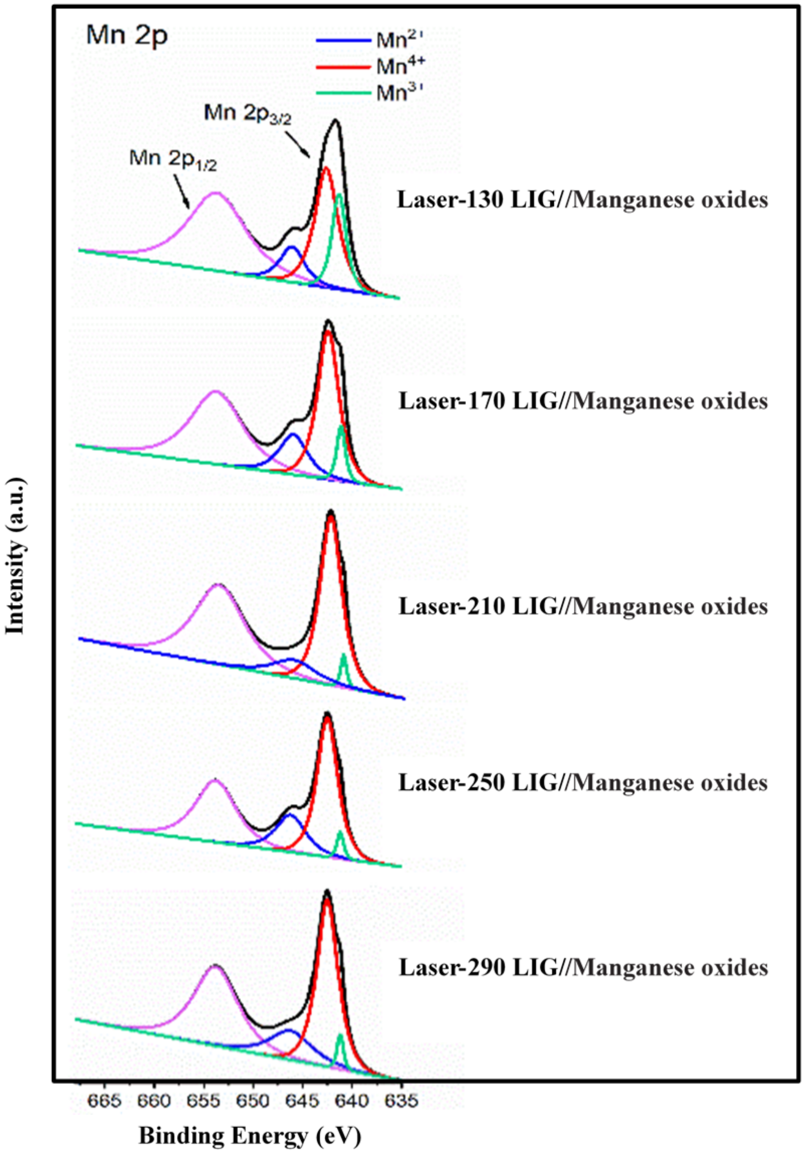

Supplementary Figure 13: XPS spectra of Mn 2p for Laser-130LIG/Manganese oxide, Laser-170LIG/Manganese oxide, Laser-210 LIG/Manganese oxide, Laser-250 LIG/Manganese oxide, and Laser-290LIG/Manganese oxide. To demonstrate that femtosecond laser irradiation can be used to control the synthesis of manganese oxide, we performed XPS measurements to investigate the manganese oxide and graphene composites. The figure displays the high-resolution XPS spectra of Mn 2p spectra for Laser-130 LIG/Manganese oxide, Laser-170LIG/Manganese oxide, Laser-210 LIG/Manganese oxide, Laser-250LIG/Manganese oxide, and Laser-290LIG/Manganese oxide. Because the duration of the ultrashort pulse was

smaller than the time scale of most of the physical and chemical characteristics, this resulted in chemical reaction pathways; chemically selective molecular excitation can be effectively controlled by a femtosecond laser. The Mn 2p XPS spectrum exhibited three characteristic peaks at 641.2, 642.5, and 646 eV, corresponding to spin–orbit peaks of manganese with mixed valence states of +3, +4, and +2, respectively. When the fluence of the laser increased to 130 mJ/cm^2, the percentage of Mn^{4+} in the LIG/Manganese oxides exceeded 50%. The percentage of Mn^{3+} in the LIG/Manganese oxides materials remained high when the percentage of Mn^{2+} was at its lowest. As the fluence of the laser gradually increases, the percentage of manganese in different valence states changes.

Supplementary Table 2. Summary of the XPS data for the LIG/Manganese oxides induced by different laser fluence

Material	Mn^{4+}2p [eV]	Mn^{2+} 2p [eV]	Mn^{3+}2p [eV]
Laser-130LIG/Manganese oxides	642.4(53.9%)	646.0(15.5%)	641.1(30.6%)
Laser-170LIG/Manganese oxides	642.6(66.4%)	646.1(23.7%)	641.2(9.9%)
Laser-210LIG/Manganese oxides	642.5**(79.4%)**	646.0**(16.6%)**	641.1**(4.0%)**
Laser-250LIG/Manganese oxides	642.6(69.1%)	646.2(26.7%)	641.2(4.2%)
Laser-290LIG/Manganese oxides	642.6(71.4%)	646.2(24.2%)	641.2(4.2%)

Supplementary Table 2. Characteristic peaks and percentages of Mn^{2+}, Mn^{3+} and Mn^{4+}. The Mn 2p spectra of LIG/Manganese oxides photoinduced by lasers with different fluences indicate that the electron BE assigned to Mn^{2+}, Mn^{3+} and Mn^{4+} 2p is close to 646.0 eV, 641.2 eV, and 642.5 eV, respectively. The percentages of Mn^{4+} and Mn^{3+} were the highest (79.4%) and lowest (4%) when the laser fluence was 210 mJ/cm^2. Conversely, the percentage of Mn^{2+} ions decreased from 23.7% to 16.6% and then increased from 16.6% to 26.7% as the laser fluence increased. Altering the laser fluence thus enables various material compositions of LIG/Manganese oxides to be successfully synthesized through photomodulation of the reaction mechanisms. The

percentage of manganese in different valence states can be adjusted flexibly.

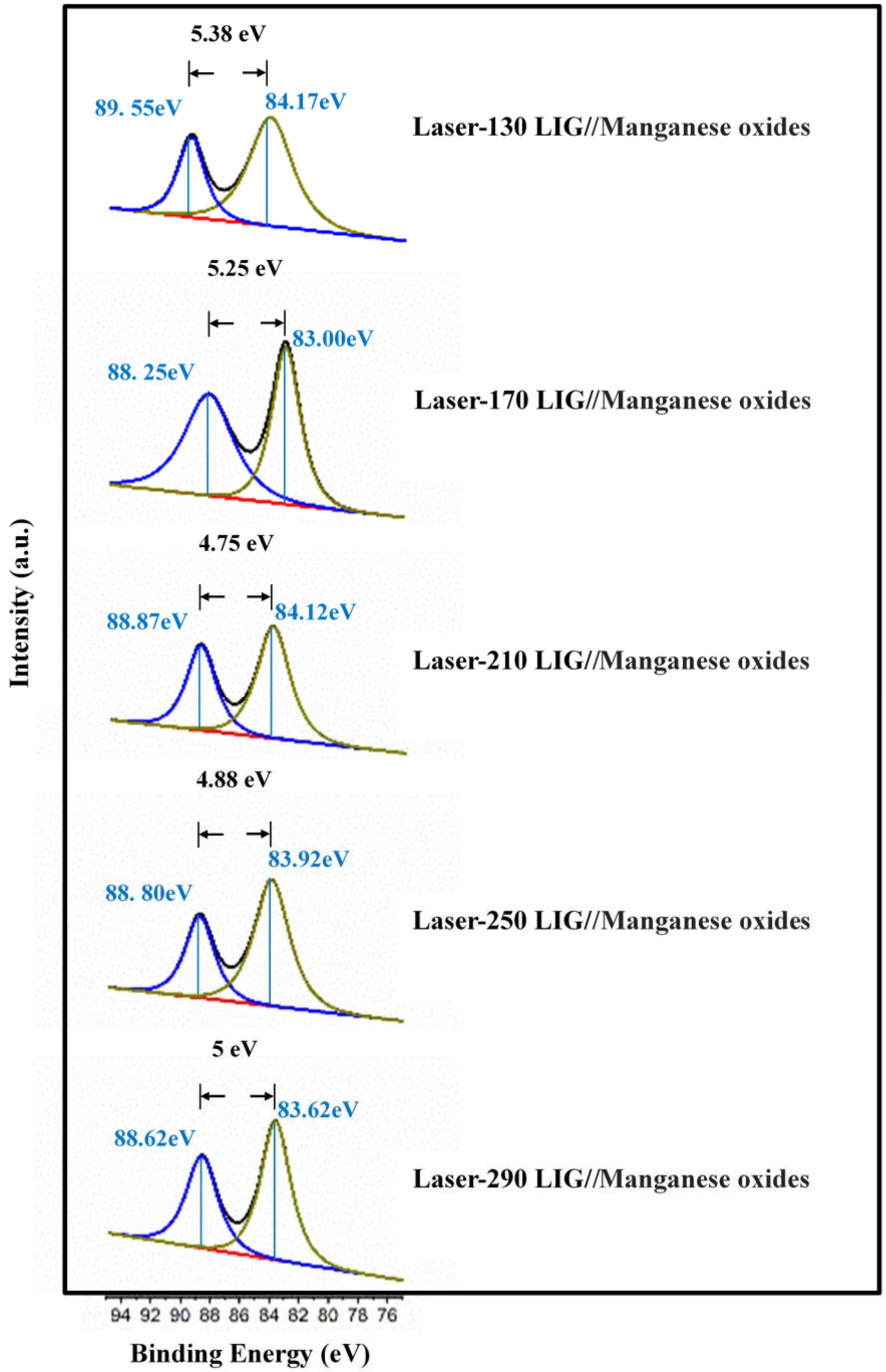

Supplementary Figure 14: XPS spectra of Mn 3s for Laser-130, 170, 210, 250 and 290 LIG/Manganese oxide. Mn 3s is more sensitive to the average oxidation state of manganese than Mn 2p is. The energy separation between the two peaks (ΔE) is closely related to the mean manganese oxidation state. The Mn 3s XPS spectra reveals

that the ΔE values of a Laser-130LIG/Manganese oxide, Laser-170LIG/Manganese oxide, Laser-210 LIG/Manganese oxide, Laser-250LIG/Manganese oxide, and Laser-290LIG/Manganese oxide are 5.38, 5.25, 4.75, 4.88, and 5.0 eV, respectively The position of the lower binding energy peak gradually shifts to higher energy as the oxidation number of the sample increases, which is consistent with previous results. The standard ΔE values for MnO_2, Mn_2O_3, and MnO are 4.7, 5.2, and 5.8eV, respectively. When the laser fluence was 210 mJ/cm^2, ΔE was 4.75, which is extremely close to the MnO_2 value. As the laser fluence increases or decreases, the value of ΔE increases, which indicates the presence of manganese oxides of lower-valence states. There are typically defects in the interlayers, such as vacancies and Mn^{4+} substitution by $Mn^{3+}$and Mn^{2+}. This result is consistent with those discussed earlier.

[1] Lei, Z., Shi, F., & Lu, L. Incorporation of MnO2-coated carbon nanotubes between graphene sheets as supercapacitor electrode. ACS applied materials & interfaces, 4(2), 1058-1064 (2012).

[2] Zhu, S. J., Zhang, J., Ma, J. J., Zhang, Y. X., & Yao, K. X. Rational design of coaxial mesoporous birnessite manganese dioxide/amorphous-carbon nanotubes arrays for advanced asymmetric supercapacitors. Journal of Power Sources, 278, 555-561 (2015).

[3] Singu, B. S., & Yoon, K. R. Synthesis and characterization of MnO2-decorated graphene for supercapacitors. Electrochimica Acta, 231, 749-758 (2017).

[4] Dong, X., Wang, X., Wang, J., Song, H., Li, X., Wang, L., ... & Chen, P. Synthesis of a MnO2–graphene foam hybrid with controlled MnO2 particle shape and its use as a supercapacitor electrode. Carbon, 50(13), 4865-4870 (2012).

[5] Zhao, Y., Ran, W., He, J., Huang, Y., Liu, Z., Liu, W., ... & Gao, F. High‐performance asymmetric supercapacitors based on multilayer MnO2/graphene oxide nanoflakes and hierarchical porous carbon with enhanced cycling stability. Small, 11(11), 1310-1319 (2015).

[6] Peng, L., Peng, X., Liu, B., Wu, C., Xie, Y., & Yu, G. Ultrathin two-dimensional MnO2/graphene hybrid nanostructures for high-performance, flexible planar supercapacitors. Nano letters, 13(5), 2151-2157 (2013).

[7] Zhang, Z., Xiao, F., Qian, L., Xiao, J., Wang, S., & Liu, Y. Facile Synthesis of 3D MnO2–Graphene and Carbon Nanotube–Graphene Composite Networks for High‐Performance, Flexible, All‐Solid‐State Asymmetric Supercapacitors. Advanced Energy Materials, 4(10), 1400064 (2014).

[8] He, Y., Chen, W., Li, X., Zhang, Z., Fu, J., Zhao, C., & Xie, E. Freestanding three-dimensional graphene/MnO2 composite networks as ultralight and flexible supercapacitor electrodes. ACS nano, 7(1), 174-182 (2013).

[9] Jia, H., Cai, Y., Lin, J., Liang, H., Qi, J., Cao, J., ... & Fei, W. Heterostructural graphene quantum dot/MnO2 nanosheets toward high‐potential window electrodes for high‐performance supercapacitors. Advanced Science, 5(5), 1700887 (2018).

[10] Lenzner, M., Krüger, J., Sartania, S., Cheng, Z., Spielmann, C., Mourou, G., ... & Krausz, F. Femtosecond optical breakdown in dielectrics. Physical review letters, 80(18), 4076 (1998).

[11] Joglekar, A. P., Liu, H. H., Meyhöfer, E., Mourou, G., & Hunt, A. J. Optics at critical intensity: Applications to nanomorphing. Proceedings of the National Academy of Sciences, 101(16), 5856-5861 (2004).

[12] Jiang, L., & Tsai, H. L. Repeatable nanostructures in dielectrics by femtosecond laser pulse trains. Applied Physics Letters, 87(15), 151104 (2005).

[13] Gattass, R. R., & Mazur, E. Femtosecond laser micromachining in transparent materials. Nature photonics, 2(4), 219-225 (2008).

[14] Kymakis, E., Savva, K., Stylianakis, M. M., Fotakis, C., & Stratakis, E. Flexible organic photovoltaic cells with in situ nonthermal photoreduction of spin‐coated graphene oxide electrodes. Advanced Functional Materials, 23(21), 2742-2749 (2013).

[15] Sokolov, D. A., Rouleau, C. M., Geohegan, D. B., & Orlando, T. M. Excimer laser reduction and patterning of graphite oxide. Carbon, 53, 81-89 (2013).

[16] Xu, M., Kong, L., Zhou, W., & Li, H. Hydrothermal synthesis and pseudocapacitance properties of α-MnO2 hollow spheres and hollow urchins. The Journal of Physical Chemistry

C, 111(51), 19141-19147 (2007).
[17] Devaraj, S., & Munichandraiah, N. Effect of crystallographic structure of MnO2 on its electrochemical capacitance properties. The Journal of Physical Chemistry C, 112(11), 4406-4417 (2008).
[18] Toupin, M., Brousse, T., & Bélanger, D. Influence of microstucture on the charge storage properties of chemically synthesized manganese dioxide. Chemistry of Materials, 14(9), 3946-3952 (2002).
[19] Hwang, J. Y., El-Kady, M. F., Li, M., Lin, C. W., Kowal, M., Han, X., & Kaner, R. B. Boosting the capacitance and voltage of aqueous supercapacitors via redox charge contribution from both electrode and electrolyte. Nano Today, 15, 15-25 (2017).
[20] Kim, F., Luo, J., Cruz‐Silva, R., Cote, L. J., Sohn, K., & Huang, J. Self‐propagating domino‐like reactions in oxidized graphite. Advanced Functional Materials, 20(17), 2867-2873 (2010).
[21] Hwang, J. Y., El-Kady, M. F., Wang, Y., Wang, L., Shao, Y., Marsh, K., ... & Kaner, R. B. Direct preparation and processing of graphene/RuO2 nanocomposite electrodes for high-performance capacitive energy storage. Nano Energy, 18, 57-70 (2015).
[22] Lukatskaya, M. R., Mashtalir, O., Ren, C. E., Dall'Agnese, Y., Rozier, P., Taberna, P. L., ... & Gogotsi, Y. Cation intercalation and high volumetric capacitance of two-dimensional titanium carbide. Science, 341(6153), 1502-1505 (2013).
[23] Allen, M. J., Tung, V. C., & Kaner, R. B. Honeycomb carbon: a review of graphene. Chemical reviews, 110(1), 132-145 (2010).
[24] Novoselov, K. S., Geim, A. K., Morozov, S. V., Jiang, D., Katsnelson, M. I., Grigorieva, I., ... & Firsov, A. A. Two-dimensional gas of massless Dirac fermions in graphene. Nature, 438(7065), 197-200 (2005).
[25] Ma, W., Chen, S., Yang, S., Chen, W., Weng, W., Cheng, Y., & Zhu, M. Flexible all-solid-state asymmetric supercapacitor based on transition metal oxide nanorods/reduced graphene oxide hybrid fibers with high energy density. Carbon, 113, 151-158 (2017).
[26] Wang, X., Wan, F., Zhang, L., Zhao, Z., Niu, Z., & Chen, J. Large‐area reduced Graphene oxide composite films for flexible asymmetric Sandwich and microsized Supercapacitors. Advanced Functional Materials, 28(18), 1707247 (2018).
[27] Chen, S., Zhu, J., Wu, X., Han, Q., & Wang, X. Graphene oxide− MnO2 nanocomposites for supercapacitors. ACS nano, 4(5), 2822-2830 (2010).
[28] Zhu, Y., Fan, X., Suo, L., Luo, C., Gao, T., & Wang, C. Electrospun FeS2@ carbon fiber electrode as a high energy density cathode for rechargeable lithium batteries. ACS nano, 10(1), 1529-1538 (2016).
[29] Papandrea, B., Xu, X., Xu, Y., Chen, C. Y., Lin, Z., Wang, G., ... & Duan, X. Three-dimensional graphene framework with ultra-high sulfur content for a robust lithium–sulfur battery. Nano research, 9(1), 240-248 (2016).
[30] Zhu, J., Wang, T., Fan, F., Mei, L., & Lu, B. Atomic-scale control of silicon expansion space as ultrastable battery anodes. ACS nano, 10(9), 8243-8251 (2016).
[31] El-Kady, M. F., & Kaner, R. B. Scalable fabrication of high-power graphene MSCs for flexible and on-chip energy storage. Nature communications, 4, 1475 (2013).
[32] Zhang, C., Kremer, M. P., Seral‐Ascaso, A., Park, S. H., McEvoy, N., Anasori, B., ... & Nicolosi, V. Stamping of flexible, coplanar micro‐supercapacitors using MXene inks.

Advanced Functional Materials, 28(9), 1705506 (2018).

[33] Liu, Y., Weng, B., Xu, Q., Hou, Y., Zhao, C., Beirne, S., ... & Chen, J. Facile fabrication of flexible MSC with high energy density. Advanced Materials Technologies, 1(9), 1600166 (2016).

[34] Liu, Z., Wu, Z. S., Yang, S., Dong, R., Feng, X., & Müllen, K. Ultraflexible in - plane micro - supercapacitors by direct printing of solution - processable electrochemically exfoliated graphene. Advanced Materials, 28(11), 2217-2222 (2016).

[35] Wang, W., Liu, W., Zeng, Y., Han, Y., Yu, M., Lu, X., & Tong, Y. A novel exfoliation strategy to significantly boost the energy storage capability of commercial carbon cloth. Advanced materials, 27(23), 3572-3578 (2015).

[36] Jeon, H., Jeong, J. M., Hong, S. B., Yang, M., Park, J., Hwang, S. Y., & Choi, B. G. Facile and fast microwave-assisted fabrication of activated and porous carbon cloth composites with graphene and MnO2 for flexible asymmetric supercapacitors. Electrochimica Acta, 280, 9-16 (2018).

[37] Wei, Z., Wang, D., Kim, S., Kim, S. Y., Hu, Y., Yakes, M. K., ... & King, W. P. Nanoscale tunable reduction of graphene oxide for graphene electronics. Science, 328(5984), 1373-1376 (2010).

[38] Zhang, K., Fu, Q., Pan, N., Yu, X., Liu, J., Luo, Y., ... & Hou, J. Direct writing of electronic devices on graphene oxide by catalytic scanning probe lithography. Nature communications, 3(1), 1-6 (2012).

[39] Gao, W., Singh, N., Song, L., Liu, Z., Reddy, A. L. M., Ci, L., ... & Ajayan, P. M. Direct laser writing of MSCs on hydrated graphite oxide films. Nature nanotechnology, 6(8), 496-500 (2011).

[40] Wu, T., Liu, S., Li, H., Wang, L., & Sun, X. Production of reduced graphene oxide by UV irradiation. Journal of nanoscience and nanotechnology, 11(11), 10078-10081 (2011).

[41] Plotnikov, V. G., Smirnov, V. A., & Alfimov, M. V. Photophysical processes and the photodissociation of chemical bonds in polyatomic molecules. High Energy Chemistry, 41(3), 131-152 (2007).

[42] Zhang, H., & Miyamoto, Y. Graphene production by laser shot on graphene oxide: An ab initio prediction. Physical Review B, 85(3), 033402 (2012).

[43] Runge, E., & Gross, E. K. Density-functional theory for time-dependent systems. Physical Review Letters, 52(12), 997 (1984).

[44] Neugebauer, J., & Scheffler, M. Theory of adsorption and desorption in high electric fields. Surface science, 287, 572-576 (1993).

[45] Liu, W., Zhao, Y. H., Li, Y., Lavernia, E. J., & Jiang, Q. A reversible switch for hydrogen adsorption and desorption: electric fields. Physical Chemistry Chemical Physics, 11(40), 9233-9240 (2009).

[46] Miyamoto, Y., Zhang, H., & Tománek, D. Photoexfoliation of graphene from graphite: an Ab initio study. Physical review letters, 104(20), 208302 (2010).

[47] Larciprete, R., Fabris, S., Sun, T., Lacovig, P., Baraldi, A., & Lizzit, S. Dual path mechanism in the thermal reduction of graphene oxide. Journal of the American Chemical Society, 133(43), 17315-17321 (2011).

[48] Zhou, S., & Bongiorno, A. Origin of the chemical and kinetic stability of graphene oxide. Scientific reports, 3, 2484 (2013).

[49] Zhang, Y. L., Guo, L., Xia, H., Chen, Q. D., Feng, J., & Sun, H. B. Photoreduction of graphene oxides: methods, properties, and applications. Advanced Optical Materials, 2(1), 10-28 (2014).

[50] Li, H., & Bubeck, C. Photoreduction processes of graphene oxide and related applications. Macromolecular Research, 21(3), 290-297 (2013).

[51] Qiu, Y., Guo, F., Hurt, R., & Külaots, I. Explosive thermal reduction of graphene oxide-based materials: mechanism and safety implications. Carbon, 72, 215-223 (2014).

[52] Zhang, X., Zhang, H., Li, C., Wang, K., Sun, X., & Ma, Y. Recent advances in porous graphene materials for supercapacitor applications. Rsc Advances, 4(86), 45862-45884 (2014).

[53] Sun, H., Zhu, J., Baumann, D., Peng, L., Xu, Y., Shakir, I., ... & Duan, X. Hierarchical 3D electrodes for electrochemical energy storage. Nature Reviews Materials, 4(1), 45-60 (2019).

[54] Wu, Z. S., Parvez, K., Feng, X., & Müllen, K. Photolithographic fabrication of high-performance all-solid-state graphene-based planar MSCs with different interdigital fingers. Journal of Materials Chemistry A, 2(22), 8288-8293 (2014).

[55] Shi, X., Pei, S., Zhou, F., Ren, W., Cheng, H. M., Wu, Z. S., & Bao, X. Ultrahigh-voltage integrated MSCs with designable shapes and superior flexibility. Energy & Environmental Science, 12(5), 1534-1541 (2019).

[56] Shi, X., Wu, Z. S., Qin, J., Zheng, S., Wang, S., Zhou, F., ... & Bao, X. Graphene‐Based Linear Tandem Micro‐Supercapacitors with Metal‐Free Current Collectors and High‐Voltage Output. Advanced Materials, 29(44), 1703034 (2017).

[57] Wu, Z. S., Parvez, K., Feng, X., & Müllen, K. Photolithographic fabrication of high-performance all-solid-state graphene-based planar MSCs with different interdigital fingers. Journal of Materials Chemistry A, 2(22), 8288-8293 (2014).

[58] Shi, X., Wu, Z. S., Qin, J., Zheng, S., Wang, S., Zhou, F., ... & Bao, X. Graphene‐Based Linear Tandem Micro‐Supercapacitors with Metal‐Free Current Collectors and High‐Voltage Output. Advanced Materials, 29(44), 1703034 (2017).

[59] Dinh, T. M., Armstrong, K., Guay, D., & Pech, D. High-resolution on-chip supercapacitors with ultra-high scan rate ability. Journal of Materials Chemistry A, 2(20), 7170-7174 (2014).

[60] Wang, S., Wu, Z. S., Zheng, S., Zhou, F., Sun, C., Cheng, H. M., & Bao, X. Scalable fabrication of photochemically reduced graphene-based monolithic MSCs with superior energy and power densities. ACS nano, 11(4), 4283-4291 (2017).

[61] Fu, X. Y., Chen, D. L., Liu, Y., Jiang, H. B., Xia, H., Ding, H., & Zhang, Y. L. Laser reduction of nitrogen-rich carbon nanoparticles@ graphene oxides composites for high rate performance supercapacitors. ACS Applied Nano Materials, 1(2), 777-784 (2018).

[62] Huang, G. W., Li, N., Du, Y., Feng, Q. P., Xiao, H. M., Wu, X. H., & Fu, S. Y. Laser-printed in-plane MSCs: from symmetric to asymmetric structure. ACS applied materials & interfaces, 10(1), 723-732 (2018).

[63] Huang, P., Heon, M., Pech, D., Brunet, M., Taberna, P. L., Gogotsi, Y., ... & Simon, P. MSCs from carbide derived carbon (CDC) films on silicon chips. Journal of Power Sources, 225, 240-244 (2013).

[64] Liu, L., Ye, D., Yu, Y., Liu, L., & Wu, Y. Carbon-based flexible MSC fabrication via mask-free ambient micro-plasma-jet etching. Carbon, 111, 121-127 (2017).

[65] Lin, Y., Gao, Y., & Fan, Z. Printable Fabrication of Nanocoral‐Structured Electrodes for

High‐Performance Flexible and Planar Supercapacitor with Artistic Design. Advanced Materials, 29(43), 1701736 (2017).

[66] Zheng, S., Wu, Z. S., Wang, S., Xiao, H., Zhou, F., Sun, C., ... & Cheng, H. M. Graphene-based materials for high-voltage and high-energy asymmetric supercapacitors. Energy Storage Materials, 6, 70-97 (2017).

[67] Sung, J. H., Kim, S. J., & Lee, K. H. Fabrication of microcapacitors using conducting polymer microelectrodes. Journal of power sources, 124(1), 343-350 (2003).

[68] Li, R. Z., Peng, R., Kihm, K. D., Bai, S., Bridges, D., Tumuluri, U., ... & Hu, A. High-rate in-plane MSCs scribed onto photo paper using in situ femtolaser-reduced graphene oxide/Au nanoparticle microelectrodes. Energy & Environmental Science, 9(4), 1458-1467 (2016).

2. REVIEWER NO.2

2.0 Reviewer's Comments

"Yuan et al. have developed a femtosecond laser stamp method to fabricate LIG/MnO_2 based MSCs. It's fascinating that this work demonstrates an ultrafast method to reduce graphene oxide and produce patterned MSCs in a very short time. However, this work still needs a lot of improvement before its publication in a high-level scientific journal such as Nature Communications. For such a high profile journal, the current discussion and data presentation need significant improvement. Here are some detailed comments:"

Our Response

We appreciate the reviewer's efforts in reviewing our paper. We greatly appreciate your suggestions, and the detailed comments are insightful and critical for improving the manuscript. We are happy to have received such excellent advice for improving the quality of the paper.

2.1 Reviewer's Comments

2.1. "The authors should provide more discussion about the reason for reshaping the ss-fs laser and the meaning of optimization of the GS (Gerchberg–Saxton?) algorithm. What's the relationship to achieve "ultra-fast patterning of laser induced graphene/MnO_2 electrodes"? Is it to get better resolution or faster pattern speed? More discussion and experimental results should be provided to support the calculation at the beginning of the discussion part."

Our Response

The detailed comments proved helpful in improving our paper. We erred in not clearly expressing the importance of reshaping the femtosecond laser and optimization of the Gerchberg–Saxton (GS) algorithm. We had previously introduced

the light field distribution of the shaped beam after SLM and dynamic transformation of high-quality shaped beams in the supplementary materials but we did not discuss it in detail. We have now provided a more detailed discussion of the advantages of shaped femtosecond laser and the optimization of algorithms.

First, we used a spatially shaped femtosecond laser to achieve "ultrafast patterning of laser-induced graphene/MnO_2 electrodes." This innovative technology was used to shape the light field into an arbitrary pattern in space so that it can be used to pattern the complete MSC. "The reason for reshaping the ss–fs laser" was to obtain the patterned light. We implemented this technology to fabricate the MSCs rapidly.

In this way, each single pulse can be achieved a MSC processing. We used the Ti: sapphire laser regenerative amplifier system, it can generate 1,000 single pulses per second, so a single MSC can be fabricated in just 0.001 seconds. The processing efficiency was tens or hundreds of times more efficient than previously reported processes of MSCs[R1-6].

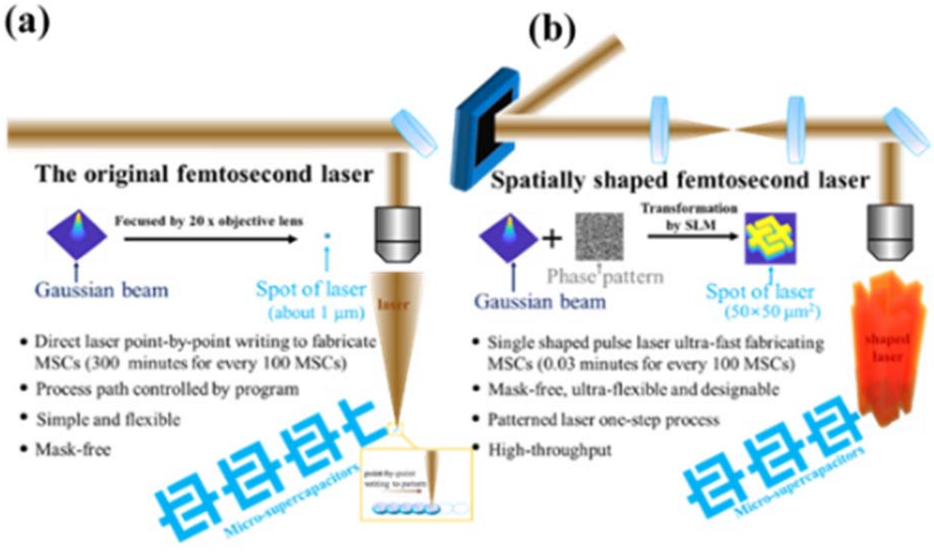

Figure R2.1 Schematics of the fabrication of micro-supercapcacitors using (a) direct laser writing and (b) spatially shaped laser

To explain the ultrahigh processing efficiency of the proposed technology clearly, we compared our technology with direct laser writing of MSCs. Figure R2.1 (a) depicts the schematics of direct laser writing of MSCs.[R1,7] The traditional laser direct

writing improves the efficiency of the fabrication of MSCs and other micro devices and is mask-free and flexible and provides high resolution. The laser point-by-point writing of the focal spot is realized by controlling the movement of the translation stage. However, its efficiency is low. Therefore, in practice, the stability of the translation stage and positioning accuracy should be considered to ensure the consistency of the process. Furthermore, achieving multipatterned rapid processing through this method is difficult.

Figure R2.1 (b) displays the schematics of our strategy to fabricate the MSCs. The incident beam (Gaussian beams) was transformed into arbitrary geometric target beams in SLM by programming phase patterns. Then, the shaped beams were transmitted on the hybrid GO films. The shaped femtosecond laser can be formed in one step, and its shape can be changed using the phase pattern. The spot of the shaped laser is a designable pattern spot that can be directly and instantaneously patterned with MSCs. In addition to exhibiting the advantages of laser direct writing, this technology considerably improved the processing efficiency and achieved a consistency similar to that of large-area processing. In theory, this process can be completed in an instant.

With the proposed experimental equipment and conditions, more than 30,000 MSCs can be produced within 10 min. If the translation stage can move at an infinite speed with an accuracy of less than 1 micron, we can fabricate 1.8 million MSCs in 30 min. This processing efficiency is dozens or even hundreds of times the efficiency of the traditional processing of MSCs. In addition, we processed the MSCs with the size of a dozen microns and used the algorithm to optimize the precision of the light field to fabricate MSCs with ultrahigh resolution.

Figure R2.1 (a) and (b) indicate that laser direct writing requires a program-controlled continuous laser scan of focused spots to complete one pattern, which is time consuming. In our method, the laser was directly changed into a patterned laser and used for processing, and a light field with controllable shape and size was used. Each pulse can achieve complete processing of a supercapacitor, which results in ultrahigh processing efficiency. Reshaping the ss–fs laser can achieve

ultrafast patterning of laser-induced graphene/MnO_2 electrodes.

In spatial shaping of the femtosecond laser, an ideal optical field pattern can be designed using the algorithm in advance. Then, we can realize the output of the pattern optical field through SLM. To obtain the best machining effect, we optimized the GS algorithm to achieve a high-quality light field to fabricate a high-resolution MSC rapidly.

The GS algorithm is an iterative optimization algorithm based on Fourier transform. The algorithm can be used to calculate the phase distribution of the hologram to generate arbitrary light intensity distribution in the mirror plane[R8].

The incident light field distribution $A_0(i_1,i_2)$ and initial phase $\phi_{random}(i_1,i_2)$ were used to obtain the object plane light field $A_\infty(i_1,i_2)$, and the forward Fourier transform was then used to obtain the light field distribution $A_\infty(n_1,n_2)$ on the image plane. The amplitude value of the design field $A_{design}(n_1,n_2)$ was used to replace the calculated amplitude value, and the phase was kept unchanged. Next, we applied inverse Fourier transform and determined the amplitude $A_{01}(i_1,i_2)$ and phase $\phi_{01}(i_1,i_2)$ distribution of the input plane. The amplitude of the incident light was substituted for the amplitude calculated in the previous step, and the phase was kept unchanged to obtain the object square light field. Finally, the iteration was repeated until the target light field whose amplitude distribution satisfied the requirements was obtained. Figure R2.2 depicts the flowchart of the algorithm.

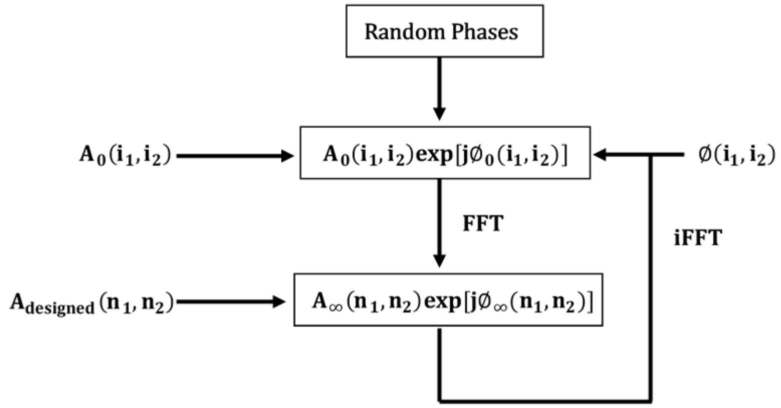

Figure R2.2 The flow chart of the algorithm

We dynamically regulated the amplitude of the target light field. First, we calculated the difference between the average amplitude obtained using the Fourier transform and the target amplitude [$\Delta = A_{\infty}(n_1, n_2) - A_{\infty}(n_1, n_2)$]. Then, we multiplied it by the weight factor ξ and replaced it with the amplitude of the plane light field[R9]. We continuously modified the amplitude of the desired target light field to accelerate the iteration process and improve uniformity. To obtain a high-quality light field, we set the cycle coefficient such that the number of iterations of the algorithm increased. Figure R2.3 (a) depicts the optimization process of the algorithm and the influence of the number of iterations on the quality of the distributions at the focal plane. The quality of the output optical field differed considerably between the optimized algorithm and the original algorithm after 50 and 200 iterations. The optimized algorithm had a high utilization ratio of light and a uniform distribution of the light field. Increasing the number of iterations resulted in better optical field distribution. Figure R2.3 (b) illustrates the optical micrographs of the different patterns (I–IV) corresponding to Figure R2.3 (a) influenced by optimization parameters.

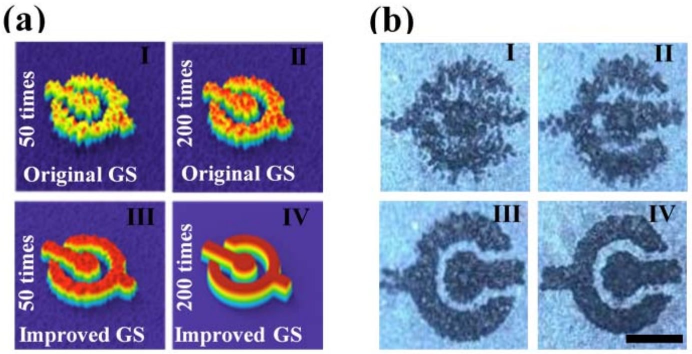

Figure R2.3 (a) The optical field distributions affected by the algorithm and the number of iterations, (b) The optical micrographs of the different patterns (scale bar, 20 μm)

We conclude that optimizing the GS algorithm results in a superior resolution optical field distribution. Figure R2.4 depicts the optical field distribution diagram optimized by the algorithm. The spatially shaped femtosecond laser can be used to directly pattern the hybrid GO film on MSCs. Furthermore, such a spatially shaped laser can be used to fabricate high-resolution MSCs in a very short time.

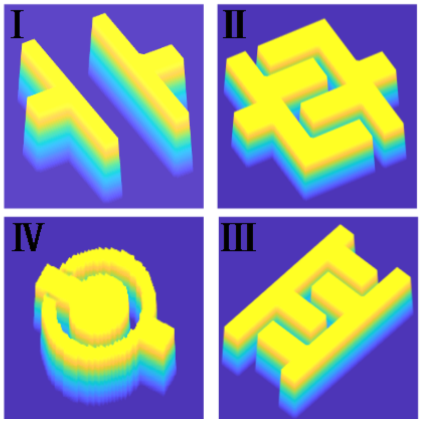

Figure R2.4 The optical field distribution diagrams optimized by the algorithm

In our technology, a perfect light field was used to achieve one-step ultrafast processing by reshaping the ss–fs laser. The objective of optimization of the GS algorithm is to obtain uniform laser spots, which is conducive for achieving high-resolution processing of MSCs. Ultrafast and high-resolution fabrication of miniature supercapacitors can be achieved through this technique, and the specific performance is listed in the following table.

Using the aforementioned conditions, we could process 3,000 MSCs in 1 min. This rate is tens or hundreds of times more efficient than that of previously reported processes of MSCs[R1-6]. The manufacturing efficiency, size, and narrow gap of our strategy were compared with those of previously reported methods. The proposed technology has unprecedented manufacturing efficiency and can be used to fabricate 90,000 MSCs in 30 min.

Table R2.1: Comparison of various manufacturing methods in manufacturing efficiency, size and narrow gap of fabricated MSCs		
**Manufacturing methods**	**Size of supercapacitors (narrow gap)**	**Manufacturing efficiency /30min**
Light scribe DVD[1]	0.5×1 cm^2 (150 μm)	100
3D-Printed stamping[2]	1×2 cm^2 (400 μm)	24
Coating and laser etching[3]	1×1 cm^2 (500 μm)	180
Inkjet-printing[4]	1×2 cm^2 (1000 μm)	100
Electrochemical activation[5]	1×1 cm^2	10
Microwave radiation[6]	1×2 cm^2	180
SFLS strategy (Our work)	50×50 $μm^2$ (0.5 μm)	90,000

Modifications

According to the comments of the reviewer, we have revised the involved part

The original part form Page 4, Paragraph 2, Line 1to Line 19 is as flowing:

"**Manufactured technology for L-S-MSCs via SFLS strategy.** We constructed a complete processing system for flexible and polymorphic L-S-MSCs (Fig. 1), the ss-fs laser beam was processed into the graphene oxide (GO) hybrid film after being reshaped by spatial light modulator. In advance, we used improved GS algorithm to generate recognizable phases through the formula:

$$\varphi_n(x,y)=\varphi^{(m)}(x,y)=\frac{\varphi_n(x,y)\varphi_{n-2}(x,y)-\varphi_{n-1}^2(x,y)}{\varphi_n(x,y)-2\varphi_{n-1}(x,y)+\varphi_{n-2}(x,y)} \quad (1)$$

where n and m are the number of iterations and phase replacements. While n is 0, $\varphi_0(x,y)$ and $\varphi_n(x,y)$ are the initial value and iterative value. Then, we combined the phase values $\varphi_n(x,y)$ obtained with the amplitude of the incident light field $F(x,y)$ to generated a complex amplitude $g_n(x,y)$:

$$g_n(x,y)=G_n(u,v)\exp\left(i\psi_n(u,v)\right)=FT\left[F(x,y)\exp(i\varphi_n(x,y))\right] \quad (2)$$

the $G_n(u,v)$ is replaced in the formula by the expected optical field amplitude $G_e(x,y)$ then combined with the phase value $\psi_n(u,v)$ to get $g_n(x,y)$. According to the Fourier transform and the inverse Fourier transform, phase-only holograms can be computed by iterating the propagation field between the image and the SLM plane[25,26]. We can optimize the results based on making the sum-square error of the diffraction optical element smaller and smaller for better beam shaping. Detailed optimization calculation methods and diagrams are in the Supplementary methods. Fig. 1a reveals that the original Gaussian beam was transformed into a phase pattern after being reshaped and then transmitted by a 4f relay system. This approach can avoid the loss of light in the transmission path[27] and achieve excellent processing results."

This part is revised as following to add the discussion of the reason for reshaping the ss-fs laser and the meaning of optimization of the GS (Gerchberg–Saxton) algorithm.

"A complete processing system for flexible and polymorphic MSCs is illustrated in Fig. 1. We reconstructed the optical field distribution of the original Gaussian beam into an arbitrary patterned light field by using a spatial light modulator. Each single pulse laser with the designable patterned spot can directly and instantaneously completed a patterned MSC. The quality of the patterned spot throughout the process was crucial. We used the improved Gerchberg–Saxton algorithm to generate recognizable phases through the formula:

$$\varphi_n(x,y) = \varphi^{(m)}(x,y) = \frac{\varphi_n(x,y)\varphi_{n-2}(x,y) - \varphi_{n-1}^2(x,y)}{\varphi_n(x,y) - 2\varphi_{n-1}(x,y) + \varphi_{n-2}(x,y)} \quad (1)$$

where n and m are the number of iterations and phase replacements. While n is 0, $\varphi_0(x,y)$ and $\varphi_n(x,y)$ are the initial value and iterative value. Then, we combined the phase values $\varphi_n(x,y)$ obtained with the amplitude of the incident light field $F(x,y)$ to generated a complex amplitude $g_n(x,y)$:

$$g_n(x,y)=G_n(u,v)\exp\left(i\psi_n(u,v)\right)=FT\left[F(x,y)\exp(i\varphi_n(x,y))\right] \quad (2)$$

the $G_n(u,v)$ is replaced in the formula by the expected optical field amplitude $G_e(x,y)$ then combined with the phase value $\psi_n(u,v)$ to get $g_n(x,y)$ .According to the Fourier transform and the inverse Fourier transform, phase-only holograms can be computed by iterating the propagation field between the image and the SLM plane[25,26]. We can obtain a better light field by increasing the number of iterations appropriately and making the sum-square error of the diffraction optical element smaller and smaller. The simulation results of optical field optimization, detailed interpretation and flowchart of the algorithm and the comparison of experimental processing results are in the Supplementary Information 1.3. We used the optimized algorithm regulate the optical field region, in which the beam width and z-axis propagation distance represent the basic parameters of a three-dimensional optical field. We can arbitrarily change the parameter design to control the size of the optical field, and a fixed depth for the 3D processing within a certain range. Since the SSFL has a uniform light field on each plane in the z-axis direction, excellent 3D reduction can be achieved in the processing of GO hybrid film. Fig. 1a reveals that the original Gaussian beam was transformed into a phase pattern after being reshaped and then transmitted by a 4f relay system. This approach can avoid the loss of light in the transmission path[27] and achieve excellent processing results."

The corresponding content added in Supporting Information is as following:

1.3 Optimization of the Gerchberg–Saxton (GS) algorithm

In spatial shaping of the femtosecond laser, an ideal optical field pattern can be designed using the algorithm in advance. Then, we can realize the output of the pattern optical field through SLM. To obtain the best machining effect, we optimized the GS algorithm to achieve a high-quality light field to fabricate a high-resolution MSC rapidly.

The GS algorithm is an iterative optimization algorithm based on Fourier transform. The algorithm can be used to calculate the phase distribution of the

hologram to generate arbitrary light intensity distribution in the mirror. The flow chart of the algorithm is shown below,

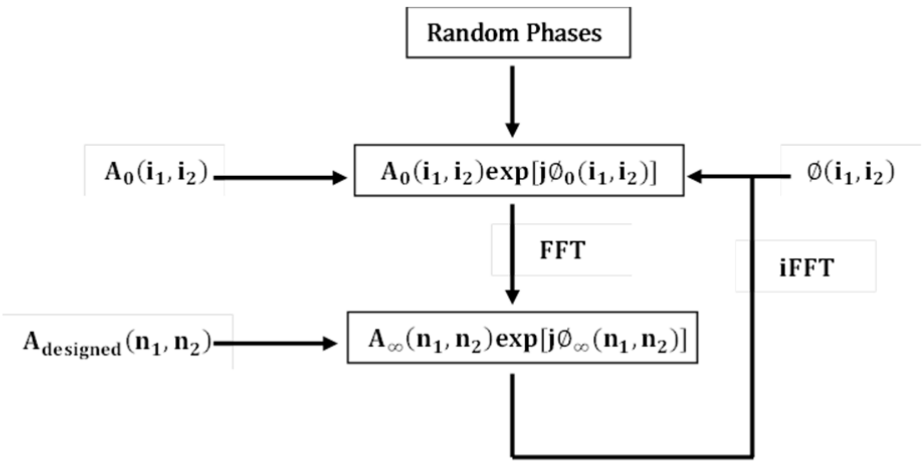

The flow chart of the algorithm

The incident light field distribution $A_0(i_1,i_2)$ and initial phase $\phi_{random}(i_1,i_2)$ were used to obtain the object plane light field $A_\infty(i_1,i_2)$, and the forward Fourier transform was then used to obtain the light field distribution $A_\infty(n_1,n_2)$ on the image plane. The amplitude value of the design field $A_{design}(n_1,n_2)$ was used to replace the calculated amplitude value, and the phase was kept unchanged. Next, we applied inverse Fourier transform and determined the amplitude $A_{01}(i_1,i_2)$ and phase $\phi_{01}(i_1,i_2)$ distribution of the input plane. The amplitude of the incident light was substituted for the amplitude calculated in the previous step, and the phase was kept unchanged to obtain the object square light field. Finally, the iteration was repeated until the target light field whose amplitude distribution satisfied the requirements was obtained.

On this basis, we dynamically regulate the amplitude of the target light field. Firstly, we calculated the difference between the average amplitude obtained by the Fourier transform and the target amplitude, as $\Delta = A_\infty(n_1,n_2) - A_\infty(n_1,n_2)$, then multiply it by the weight factor ξ and replace it with the amplitude of the plane light field. We continuously modify the amplitude of the desired target light field to

accelerate the iteration process and improve uniformity. To obtain a high-quality light field, we set the cycle coefficient such that the number of iterations of the algorithm increased. Figure1 (a) depicts the optimization process of the algorithm and the influence of the number of iterations on the quality of the distributions at the focal plane. The quality of the output optical field differed considerably between the optimized algorithm and the original algorithm after 50 and 200 iterations. The optimized algorithm had a high utilization ratio of light and a uniform distribution of the light field. Increasing the number of iterations resulted in better optical field distribution. Figure1 (b) illustrates the optical micrographs of the different patterns (I–IV) corresponding to Figure1 (a) influenced by optimization parameters.

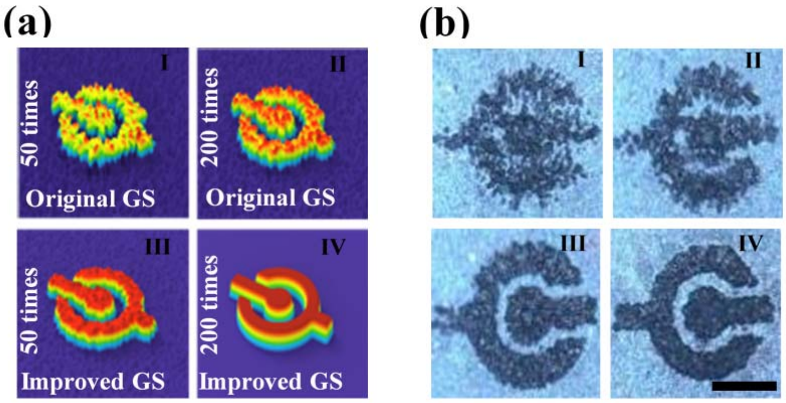

Figure 1 (a) The optical field distributions affected by the algorithm and the number of iterations, (b) The optical micrographs of the different patterns (scale bar, 20 μm)

We conclude that optimizing the GS algorithm results in a superior resolution optical field distribution. Figure R2 depicts the optical field distribution diagram optimized by the algorithm. The spatially shaped femtosecond laser can be used to directly pattern the hybrid GO film on MSCs. Furthermore, such a spatially shaped laser can be used to fabricate high-resolution MSCs in a very short time.

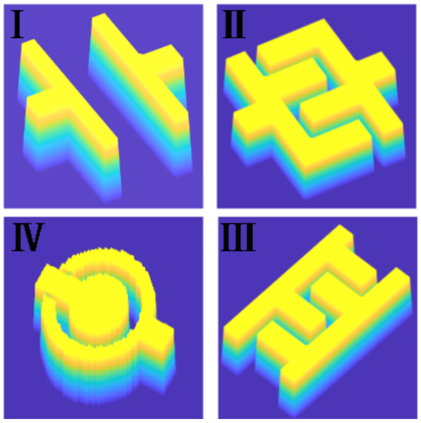

Figure 2 The optical field distribution diagrams optimized by the algorithm

All in all, our technology relies on the design of a perfect light field to achieve one-step ultra-fast processing by reshaping the ss-fs laser. And the meaning of optimization of the GS algorithm is to get more uniform laser spots which are more conducive to achieve high-resolution processing of MSCs.

The original part form Page 6, Paragraph 1, Line 1to Line 9 is as flowing:

"The ss-fs laser can achieve ultra-fast fabrication of various-shapes L-S-MSCs (Fig. 1b). Notably, such high fabrication efficiency for flexible L-S-MSCs is rare, and ss-fs laser also demonstrates high machining accuracy and near faultless processing consistency, which enable rapid and large-scale applications. Under present conditions, we could process 3,000 L-S-MSCs in 1 minute. This rate was tens or hundreds of times more efficient than previously reported processes of MSCs[28-33]. As the Table shows, our SFLS strategy is compared with the methods that have been reported before in manufacturing efficiency, size and narrow gap. The technology we proposed has unprecedented manufacturing efficiency and could fabricate 90,000 L-S-MSCs in thirty minutes. Our processing video is included in the Supplementary Information."

This part is revised as following:

"The SSFL method achieved ultrafast fabrication of variously shaped MSCs (Fig. 1b) and revolutionizes the traditional processing method of direct laser writing (Supplementary Fig. 1). Traditional laser point-by-point writing of the focal spot is realized by controlling the movement of the translation stage, which considerably limits the method's application in the ultrafast fabrication of MSCs. In our work, the

spot of each laser pulse can be a designable pattern spot shaped by a spatial light modulator that directly and instantaneously completes a patterned MSC. The SSFL strategy not only retains the advantages of being mask-free, flexibility and high-resolution, it also achieves the ultrafast fabrication of MSCs.

We used the Ti: sapphire laser regenerative amplifier system, which can generate 1,000 single pulses per second. In theory, a single MSC could be fabricated in only 1 millisecond. The actual processing speed observed was more than 3,000/min, which is tens or hundreds of times more efficient than previously reported processes for fabricating MSCs[31-36]. The SSFL also has the advantages of high machining accuracy and near faultless processing consistency, enabling rapid and large-scale application. As illustrated in the Table, our SSFL strategy was compared with the methods previously reported regarding fabrication efficiency, size, and narrow gap. The proposed technique has unprecedented fabrication efficiency and fabricated high-resolution and micron-scale supercapacitors (a processing video is included in the Supplementary Information)."

The corresponding content added in Supporting Information is as following:

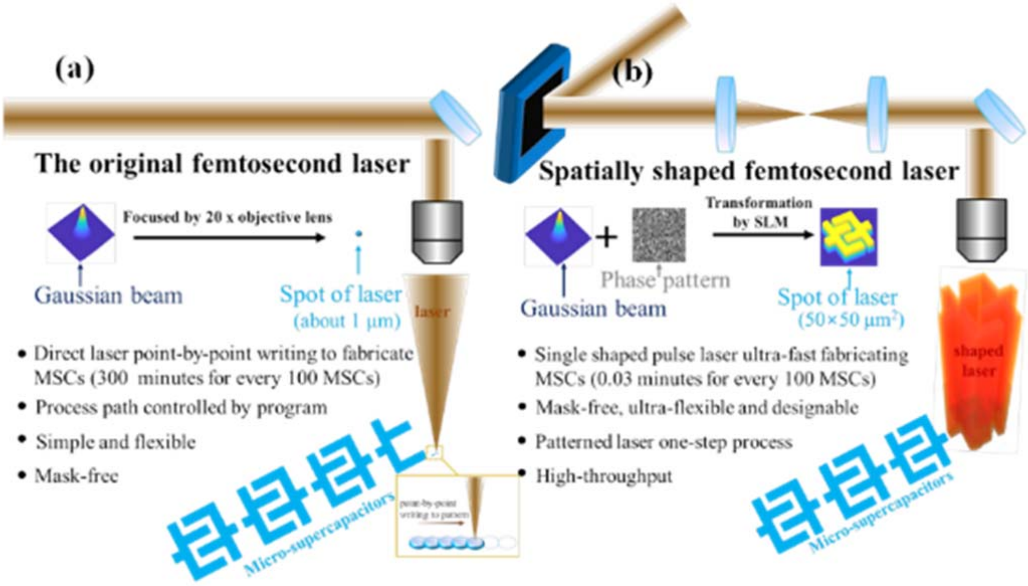

"Supplementary Figure 1: Schematics of the fabrication of MSCs using (a) direct laser writing and (b) a spatially shaped laser. Traditional laser point-by-point

writing of the focal spot is realized by controlling the movement of the translation stage. Therefore, in actual processing, we consider the stability of the translation stage and repeated positioning accuracy to ensure the consistency of processing; multipatterned rapid processing is difficult to achieve. The incident beams (Gaussian beams) were transformed into arbitrary geometric target beams in SLM by programming phase patterns; the shaped beams were then transmitted on the hybrid GO films. The shaped femtosecond laser can be formed in one step and alters the shape by changing the phase pattern. The spot of the shaped laser is a designable pattern that can directly and instantaneously complete patterned MSCs. In conjunction with the advantages of laser direct writing, this technology greatly improves processing efficiency and achieves consistency for large-area processing. In theory, this process is instantaneous."

2.2 Reviewer's Comments

2.2. "What's the spot size of the femtosecond laser used in this research work? The authors claimed that the SFLS system could achieve 500 nm resolution. However, based on the SEM images in Supplementary Figure 2, the laser induced patterns exhibited very rough edges even with an electrode gap of about 3-8 μm. The pattern demonstrated in Supplementary Figure 3, with a claimed "500 nm" narrow gap, showed random curved gap channels. The evidence to prove that the high resolution of "500 nm" has been achieved is insufficient."

Our Response

We thank the reviewer for the efforts in reviewing our paper. In our study, the spot of the femtosecond laser is not a traditional Gaussian beam. As mentioned in the previous response, the femtosecond laser can be used to reshape the patterned light field in space.

The spot size of the single femtosecond laser pulse in our study could be adjusted from 15 × 15 to 100 × 100 μm^2. Furthermore, the narrow gap could be adjusted by designing different light fields. As mentioned earlier, we used the Ti:sapphire laser regenerative amplifier system because it can generate 1,000 single pulses per second.

Therefore, in theory, a single MSC could be fabricated in just 0.001 s.

In practice, we fabricated more than 50 MSCs per second. Different sizes of MSCs were fabricated in one step by changing the spot size. To achieve a superior display, we fabricated MSCs of different sizes. As depicted in Figure R2.5 a-c, we could prepare MSCs ranging from 15 × 15 to 100 × 100 μm^2 in a short time. These capacitors can maintain regular shapes.

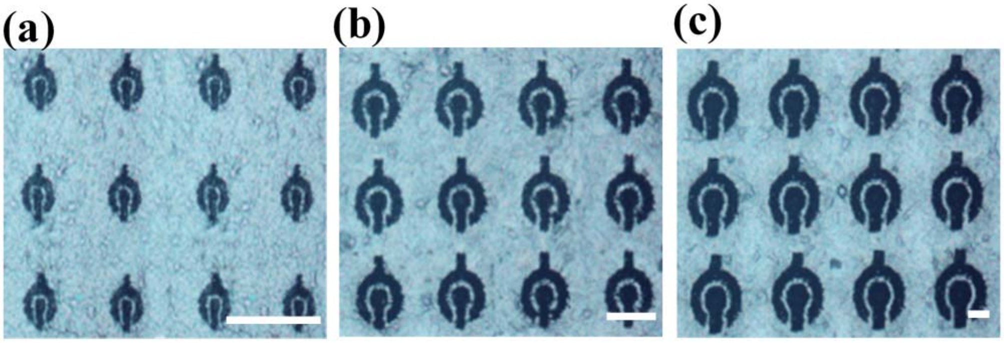

Figure R2.5 The micrographs of MSCs with varying sizes (scale bar, 50 μm)

In tests, we selected MSCs of medium size (approximately 50 × 50 μm^2) for processing because according to the electrochemical test results, medium-sized microcapacitors exhibit superior performance.

We determined that in an aqueous electrolyte solution, if the electrode narrow gap is too small, the electrode materials easily come in contact with each other with the flow of the electrolyte solution, which leads to testing failure (the effect of size and the narrow gap is discussed in detail in question 2.6). The SEM images of the MSCs depicted in Supplementary Figure 2 intuitively and clearly distinguish the shape of MSCs. The electrode gaps of these MSCs are a few microns to ensure the stability of the electrochemical test. The MSCs with such electrode gaps exhibit excellent results in electrochemical tests[R10,11]. The shape of the laser was changed by controlling the distribution of the light field, thereby achieving high-resolution fabrication. By simulating the distribution of the light field, the resulting target light field can be finely regulated (Figure R2.6). The ablation can be influenced by controlling the fluence of the laser. The ablation of the edge directly affects the narrow gaps. The narrow gaps ranged from a few microns to 500 nm (Figure R2.6,

scale bar, 20 μm).

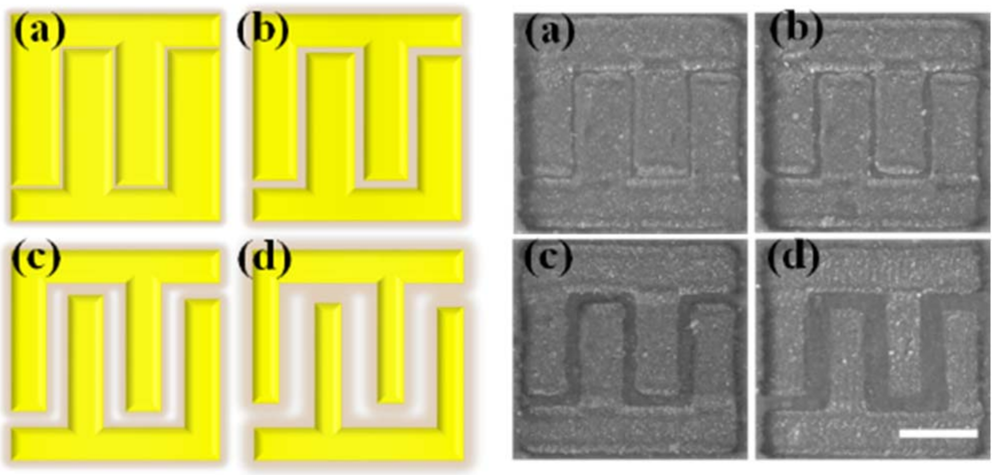

Figure R2.6 The light filed and SEM of interdigital MSCs with different narrow gaps (scale bar, 20 μm)

Notably, 500 nm is the highest resolution achieved in the processing of MSCs in this study for the convenience of subsequent electrochemical testing and studies considering light field interference. Our processing resolution was realized through the pattern design of the light field shape. Thus, the slit between design patterns was used to achieve the highest resolution.

To demonstrate the high-resolution fabrication ability of our technology, we patterned simple-shaped MSCs (Figure R2.7). We patterned parallel strip-shaped MSCs with different narrow gaps to achieve a straight line gap. In Figure R2.7, the resolution of the slit is less than 500 nm and is even close to 350 nm in some parts.

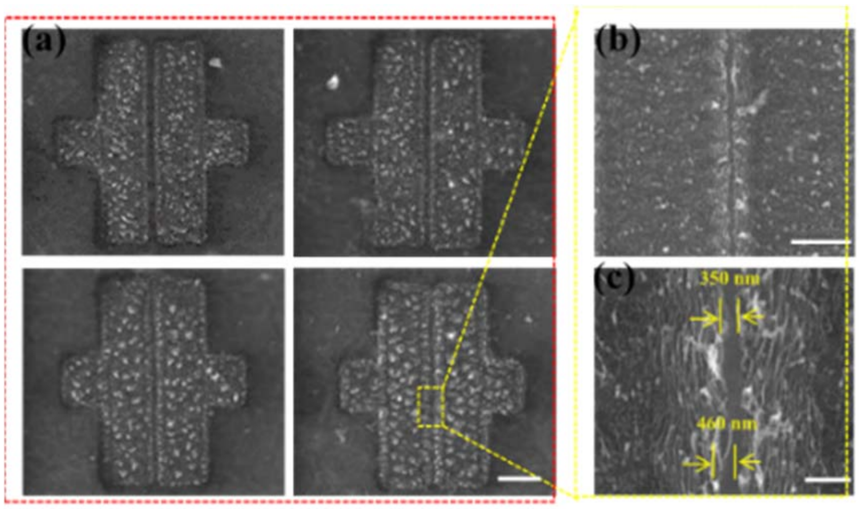

Figure R2.7 (a) The SEM images of the MSCs with different narrow gaps, (b) and (c) The SEM images of partial magnification of the narrow gap (scale bar of (a), 10 µm. scale bar of (b), 5 µm. scale bar of (c), 1 µm)

We achieved a high resolution through the pattern design of the light field shape. Thus, the slit between design patterns was used to achieve the highest resolution, which was not limited by the laser wavelength. Therefore, a 3D reduction could be achieved using the 3D shaped laser. To demonstrate the machining resolution of our technology, we performed high-resolution experiments using SSFL. We designed a two-ringed MSC with a light field distribution as depicted in Figure R2.8 (a). High-resolution patterning was realized by adjusting the narrow gap between the two rings. Figure R2.8 (b) illustrates the SEM images of the patterns with different narrow gaps. The minimum resolution of the slit was achieved at the junction of two rings. The narrow gap can be designed with the laser. We achieved an ultrasmall narrow gap of 11.6 nm, which is beyond the limit of traditional diffraction. Nanometer precision benefits from the design of light fields.

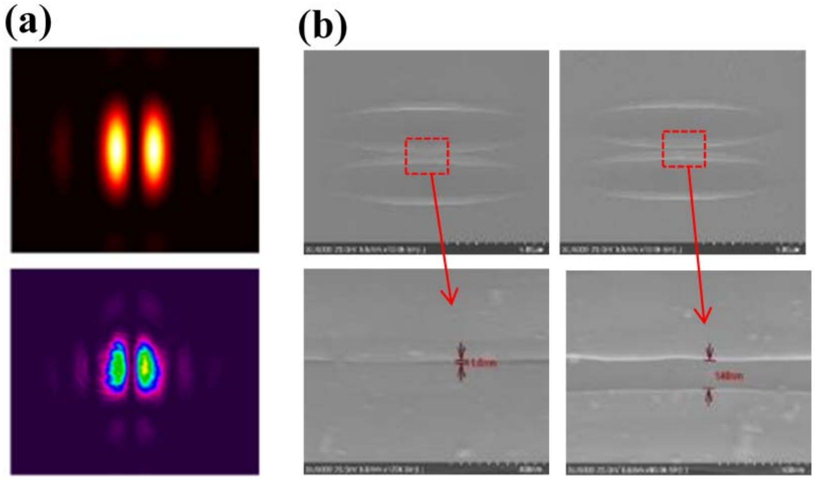

Figure R2.8 The light filed (a) and SEM images (b) of the two-rings electrodes with different narrow gaps (11.6 nm and 146 nm)

Modifications

According to the comments of the reviewer, we have revised the involved part

The original part form Page 7, Paragraph 1, Line 13 to Line 18 is as flowing:

“L-S-MSCs of various shapes are in the tens to hundreds of micrometers in length. In such a machining precision, our technology has obtained a satisfactory machining effect. Supplementary Fig. 2 clearly displays the regular electrode morphology. The spacing could be adjusted by designing different light field, and the narrow gap of the L-S-MSCs is only 500 nm (Supplementary Fig. 3). The varying sizes can be controlled by designable light fields from 15×15 μm^2 to 100×100 μm^2 (Supplementary Fig. 4).”

This part is revised as following:

“The shape of the spot could be designed, and the size of the spot could be regulated by transforming the target light field. The size of the spot determined the size of the entire MSC, because the MSCs of various shapes were patterned using the SSFL in one step. The varying sizes can be controlled by designable laser spots from 15×15 μm^2 to 100×100 μm^2 (Supplementary Fig. 3). We could transform Gaussian light into shaped light to form the patterned light spots and use the SSFL to extrude the narrow gap of patterns that could break the diffraction limit. MSCs with different narrow gaps could be fabricated by designing different light fields, and a MSC with a narrow gap of 500 nm was fabricated using SSFL on a GO hybrid film (Supplementary Fig. 4). These findings indicate that our method achieved extremely high processing efficiency while maintaining high processing accuracy.”

The corresponding content added in Supporting Information is as following:

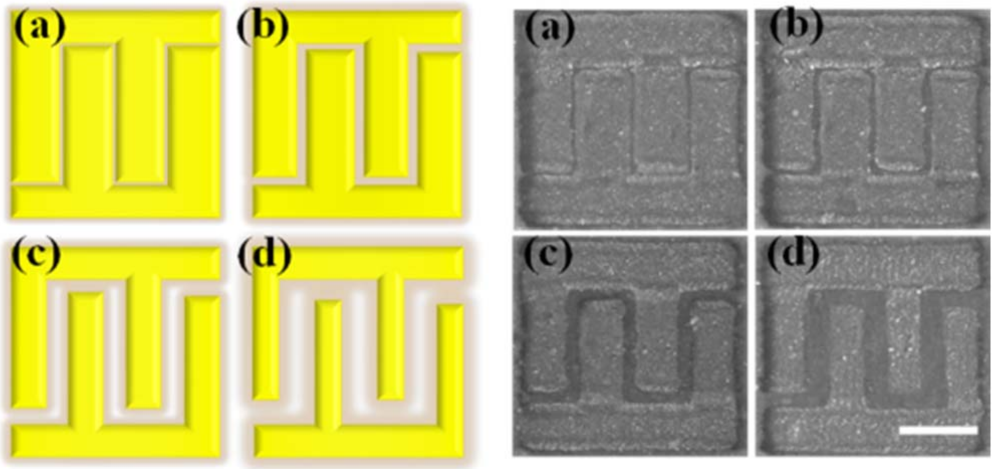

“Supplementary Figure 4-1: Light field and SEM of interdigital MSCs with

different narrow gaps (scale bar, 20 μm). We adjusted the shaped laser by controlling the distribution of the light field, thereby achieving high-resolution fabrication. By simulating the distribution, the resulting target light field can be finely regulated. We simultaneously affected the ablation by controlling the fluence of the laser; the ablation of the edge in particular directly affected the narrow gaps. These narrow gaps thus ranged from a few microns to 500 nm, the latter of which is the highest resolution we have achieved in the processing of MSCs. This is convenient for subsequent electrochemical testing and consideration of light field interference. Processing resolution was realized through the pattern design of light field shaping, where the slit between design patterns was used to achieve the highest resolution.”

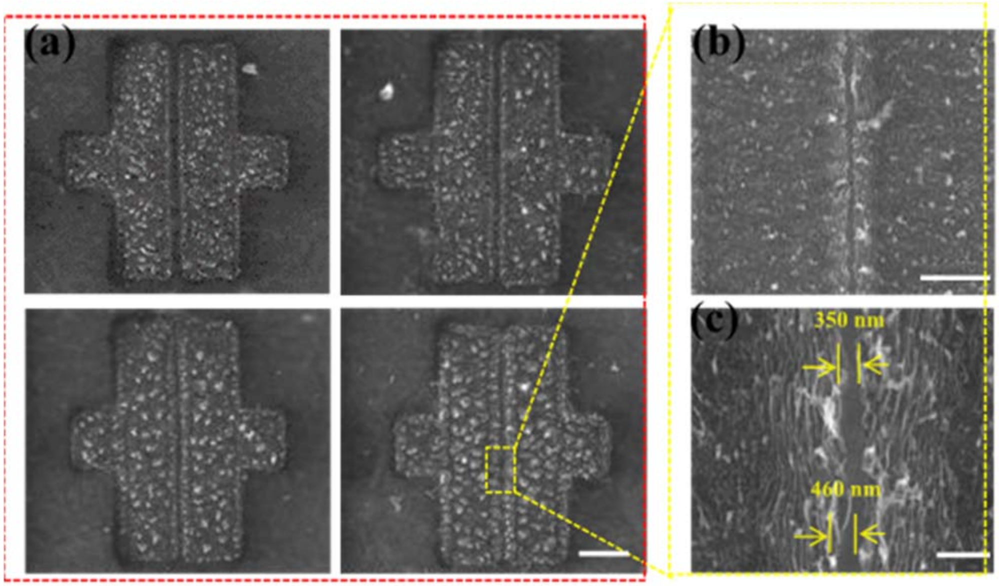

“Supplementary Figure 4-2: (a) SEM images of MSCs with different narrow gaps; (b) and (c) SEM images of partial magnification of the narrow gap (scale bar of (a) 10 μm, (b) 5 μm, and (c) 1 μm). To demonstrate the high-resolution fabrication of our technology, we patterned a simply shaped MSC to demonstrate the higher resolution of the electrode gaps. We patterned parallel strip-shaped MSCs with different narrow gaps so that the gap is a straight line and is clearer to see. As shown, the resolution of the slit is less than 500 nm, and close to 350 nm in some parts. Our resolution was realized through the pattern design of light field shaping, where the slit between design patterns was used to achieve the highest resolution but was not

limited by the laser wavelength. This potentially explains how to complete a 3D reduction using the 3D shaped laser."

2.3 Reviewer's Comments

2.3. "The physicochemical characterization of the laser induced LIG/MnO2 electrode needs further improvement. XRD characterization should be supplemented to further study the crystalline structure of the laser induced Mn-based compound(s). Although the Raman and O 1s XPS data demonstrate the presence of Mn-O covalent bonds, it's still insufficient to prove the preparation of MnO_2. In addition, the Mn 2p XPS data also show a large percentage of Mn3+ and Mn2+ in the total Mn amount. Thus, the structure of manganese in the final laser induced composite appears to be more complicated than pure MnO_2."

Our Response

We thank the reviewer for the valuable advice. As mentioned by the reviewer, the valence changes of manganese ions and the physicochemical characterization of the synthesized LIG/MnO_2 composite electrode should be studied.

Although the percentage of Mn^{4+} is very high, the Mn 2p XPS data revealed a large percentage of Mn^{3+} and Mn^{2+} in the total Mn amount.

Through detailed analysis using XPS and XRD, we found that the shaped femtosecond laser can regulate the synthesis of materials and control photoinduced and photothermal-induced reduction/oxidation and the valence state of manganese. Altering the laser fluence can also yield heterogeneous junctions of different compositions. To demonstrate that femtosecond laser irradiation can be used to control the synthesis of manganese oxide, we performed XPS measurements to investigate the manganese oxide and graphene composites. We defined the composite materials synthesized under different laser fluences as Laser-130, 170, 210, 250 and 290 LIG/Manganese oxide, corresponding to laser fluences of 130, 170, 210, 250, and 290 mJ/cm^2, respectively.

Figure R2.9 displays the high-resolution XPS spectra of Mn 2p spectra for

Laser-130, 170, 210, 250 and 290 LIG/Manganese oxide. Because the duration of the ultrashort pulse was smaller than the time scale of most of the physical and chemical characteristics, this resulted in chemical reaction pathways; chemically selective molecular excitation can be effectively controlled by a femtosecond laser. The Mn 2p XPS spectrum exhibited three characteristic peaks at 641.2, 642.5, and 646 eV, corresponding to spin–orbit peaks of manganese with mixed valence states of +3, +4, and +2, respectively.

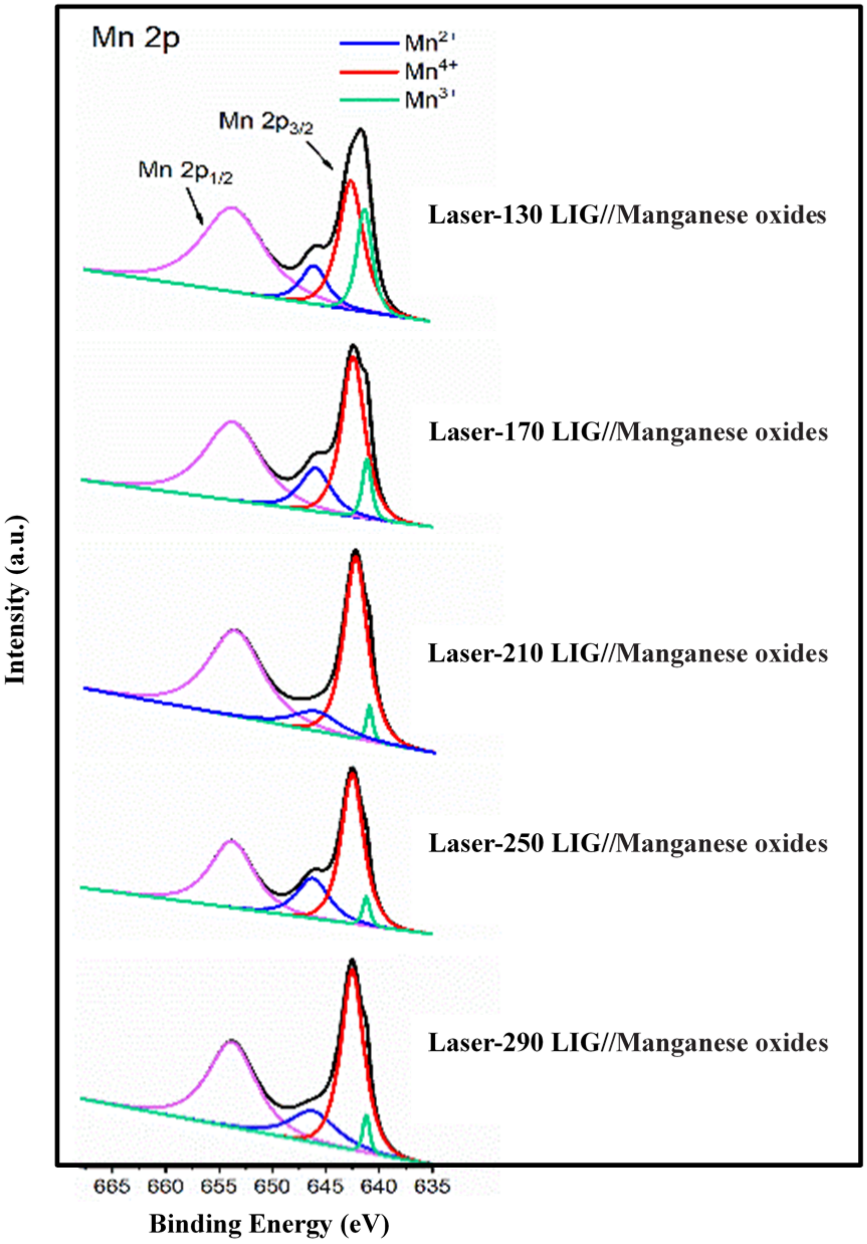

Figure R2.9 XPS spectra of Mn 2p for Laser-130 LIG/Manganese oxide,

Laser-170LIG/Manganese oxide, Laser-210 LIG/Manganese oxide, Laser-250 LIG/Manganese oxide, Laser-290LIG/Manganese oxide

When the fluence of the laser increased to 130 mJ/cm^2, the percentage of Mn^{4+} in the LIG/Manganese oxides exceeded 50%. The percentage of Mn^{3+} in the LIG/Manganese oxides materials remained high when the percentage of Mn^{2+} was at its lowest. As the fluence of the laser gradually increases, the percentage of manganese in different valence states changes.

TableR2.1 Summary of the XPS data for the LIG/Manganese oxides induced by different laser fluence

Material	Mn^{4+}2p [eV]	Mn^{2+} 2p [eV]	Mn^{3+}2p [eV]
Laser-130LIG/Manganese oxides	642.4(53.9%)	646.0(15.5%)	641.1(30.6%)
Laser-170LIG/Manganese oxides	642.6(66.4%)	646.1(23.7%)	641.2(9.9%)
Laser-210LIG/Manganese oxides	642.5**(79.4%)**	646.0**(16.6%)**	641.1**(4.0%)**
Laser-250LIG/Manganese oxides	642.6(69.1%)	646.2(26.7%)	641.2(4.2%)
Laser-290LIG/Manganese oxides	642.6(71.4%)	646.2(24.2%)	641.2(4.2%)

Table R2.1 indicates that the characteristic peaks and the percentage of Mn^{2+}, Mn^{3+} and Mn^{4+}. The Mn 2p spectra of LIG/Manganese oxides photoinduced by lasers with different fluences indicate that the electron BE assigned to Mn^{2+}, Mn^{3+} and Mn^{4+} 2p is close to 646.0 eV, 641.2 eV, and 642.5 eV, respectively. The percentages of Mn^{4+} and Mn^{3+} were the highest (79.4%) and lowest (4%) when the laser fluence was 210 mJ/cm^2. Conversely, the percentage of Mn^{2+} ions decreased from 23.7% to 16.6% and then increased from 16.6% to 26.7% as the laser fluence increased. Altering the laser fluence thus enables various material compositions of LIG/Manganese oxides to be successfully synthesized through photomodulation of the reaction mechanisms. The percentage of manganese in different valence states can be adjusted flexibly.

Mn 3s is more sensitive to the average oxidation state of manganese than Mn 2p is. The energy separation between the two peaks (ΔE) is closely related to the mean manganese oxidation state. The Mn 3s XPS spectra (Fig. R2.10) reveals that the ΔE

values of aLaser-130LIG/Manganese oxide, Laser-170LIG/Manganese oxide, Laser-210 LIG/Manganese oxide, Laser-250LIG/Manganese oxide, and Laser-290LIG/Manganese oxide are 5.38, 5.25, 4.75, 4.88, and 5.0 eV, respectively The position of the lower binding energy peak gradually shifts to higher energy as the oxidation number of the sample increases, which is consistent with previous results. The standard ΔE values for MnO_2, Mn_2O_3, and MnO are 4.7, 5.2, and 5.8eV, respectively. When the laser fluence was 210 mJ/cm^2, ΔE was 4.75, which is extremely close to the MnO_2 value.

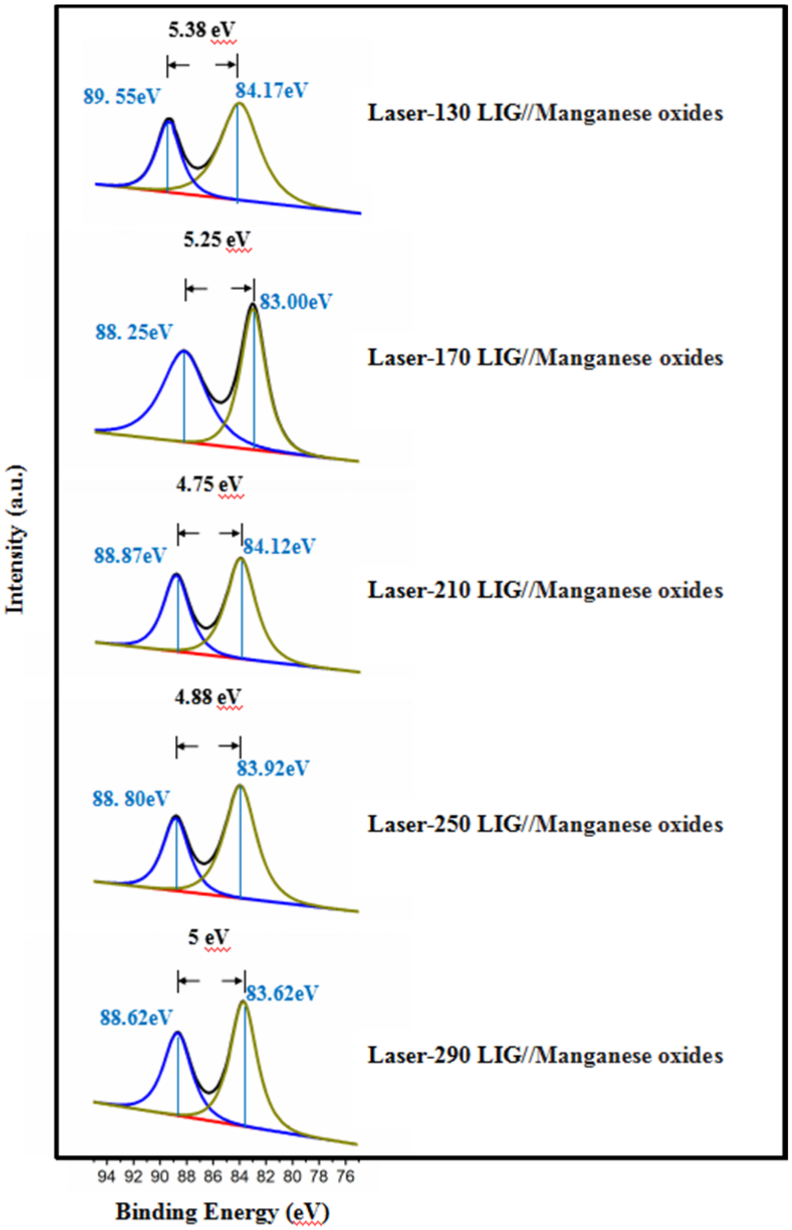

Figure R2.10 XPS spectra of Mn 3s forLaser-130 LIG/Manganese oxide, Laser-170 LIG/Manganese oxide, Laser-210 LIG/Manganese oxide, Laser-250 LIG/Manganese oxide, Laser-290LIG/Manganese oxide

As the laser fluence increases or decreases, the value of ΔE increases, which indicates the presence of manganese oxides of lower-valence states. There are typically defects in the interlayers, such as vacancies and Mn^{4+} substitution by $Mn^{3+}$and Mn^{2+}. This result is consistent with those discussed earlier.

In this study, by altering the pulse energy, materials with various compositions of LIG/Manganese oxides were successfully synthesized through photomodulation of the reaction mechanisms. By using a spatially shaped femtosecond laser, the ratio of the Mn^{4+} could be maximized, and MnO_2 as the main component is in the LIG/Manganese oxides.

Since the process of laser-regulated material synthesis is very complex, we should make a comprehensive study and exploration of laser-induced products. Therefore, XRD tests were added to characterize the composites irradiated by femtosecond laser, so as to verify the existence of the composites, especially manganese, and observe whether there are new phenomena. The XRD patterns of the as-prepared GO and LIG-MnO_2 nanocomposite crystal structure were analyzed and are shown in figure R 2.11. According to Fig. R 2.11 (a), GO has an intense peak at 2θ = 11.2° that is related to the (001) diffraction. The diffraction peaks of Laser-210 LIG/Manganese oxide appeared at 2θ = 12.73°, 18.14°, 28.82°, 37.46°, 41.95°, 49.86°, 56.32°, 60.27°, 65.11°, 69.76° and 72.73° as shown in Figure R2.11 (b), which correspond to the expected diffraction peaks of (110), (200), (310), (211), (301), (411), (600), (521), (002), (541) and (312) crystal planes of α-MnO_2 standard data, respectively, following the JCPDS card PDF file no. 44-0141. No peaks for other types of MnO_2 are observed, indicating that the as-prepared products composed only of one tetragonal α-MnO_2 phase.

The diffraction peaks of Laser-210 LIG/Manganese oxide are very similar to those of a nanotetragonal phase of α-MnO_2, where the (001) reflection peak of layered GO has almost disappeared. Our results correlate well with the previous studies that

the diffraction peaks become weakened or even disappear whenever the regular stacks of GO are exfoliated.

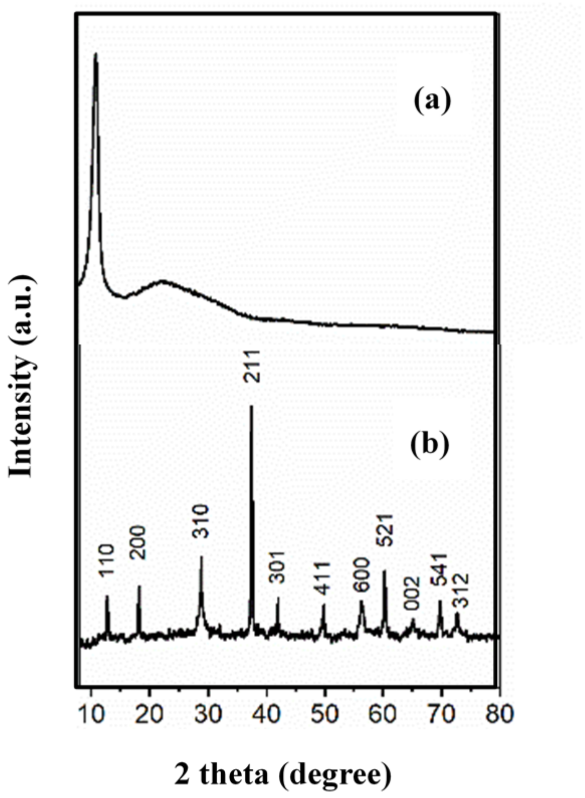

Figure R2.11 (a) XRD patterns of GO and (b) Laser-210 LIG/Manganese oxide

To gain some insights into the structures of LIG/ Manganese oxides, we collected XRD patterns at different composites (Laser-130LIG/Manganese oxides, Laser-170LIG/Manganese oxide, Laser-210 LIG/Manganese oxide, Laser-250LIG/Manganese oxide, Laser-290LIG/Manganese oxide.) processed under different laser fluence (Figure R2.12).

The XRD patterns of the LIG/Manganese oxides LIG showed peaks that were similar, which can be well indexed to plane of the tetragonal α-MnO_2 structure. However, we can still find differences through careful observation. The most obvious one is that the peak intensities of the XRD patterns of Laser-210 LIG/Manganese

oxide and Laser-250 LIG/Manganese oxide are stronger than others'.

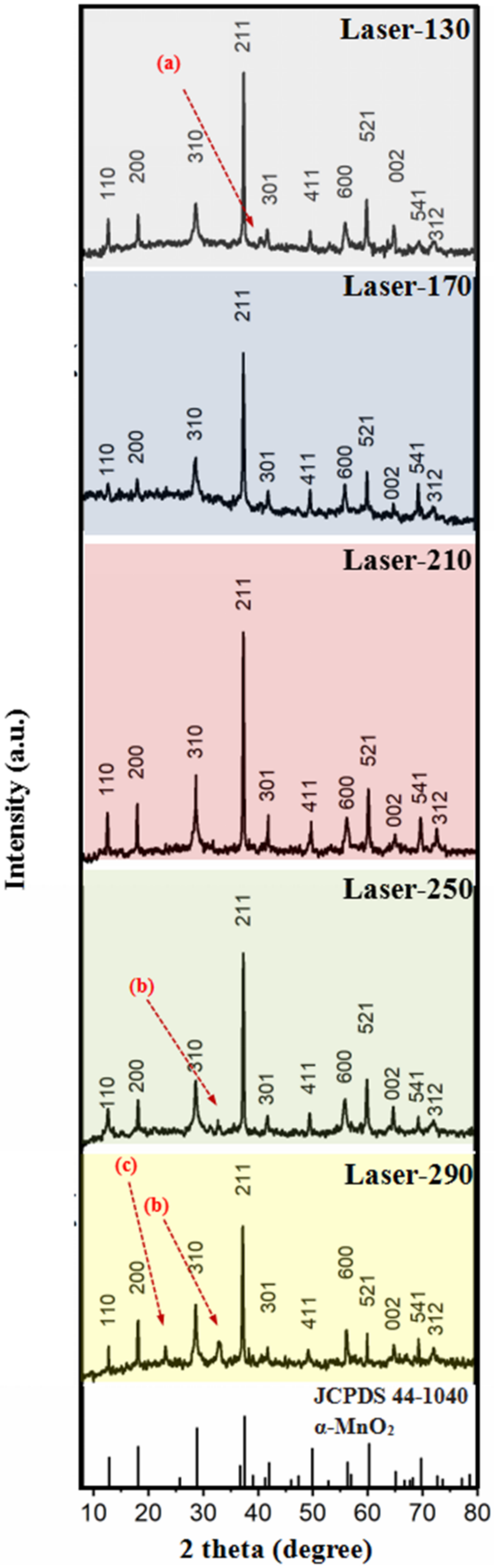
Laser-130
(a)
Laser-170
Laser-210
Laser-250
(b)
Laser-290
(c)
(b)
110
200
310
211
301
411
600
521
002
541
312
JCPDS 44-1040
α-MnO2
Intensity (a.u.)
10
20
30
40
50
60
70
80
2 theta (degree)

Figure R 2.12 XRD patterns of LIG/Manganese oxides processed under different femtosecond laser fluence

By altering the laser fluence, the diffraction peaks of LIG/Manganese oxides correspond to the expected diffraction peaks of the (110), (200), (310), (211), (301), (411), (600), (521), (002), (541) and (312) crystal planes of α-MnO_2 standard data. However, in the XRD pattern of Laser-130 LIG/Manganese oxide, a peak (a) appeared at $2\theta = 40.5°$ as shown in the figure. This corresponds to the expected diffraction peaks of the (200) crystal plane of MnO standard data, following the JCPDS card PDF file no. 74-4748. This is lower than other peaks, and gradually disappears as the laser fluence increases. When the laser fluences are 250 and 290 mJ/cm^2, peaks (b) and (c) appear at $2\theta = 33.05°$ and 23.12°, respectively. These were assigned to the (222) and (211) crystal planes of α-Mn_2O_3, following the JCPDS card PDF file no. 41-1442. The resulting diffractogram concludes the formation of α-MnO_2 (major) and α-Mn_2O_3 (minor) phases. This may be due to the conversion of MnO_2 to Mn_2O_3 as the laser fluence increases. MnO_2 continues to constitute an extremely high proportion of our laser-induced synthesis materials, especially when the laser fluence is 210 mJ/cm^2.

Thus, the femtosecond laser reduces GO, simultaneously facilitating the action of metal ions, which is a novel mechanism of action. Consequently, GO reduction and MnO_2 synthesis are mutually reinforcing processes in shaped femtosecond laser radiation. By altering the pulse energy, materials of various LIG/manganese oxide compositions were successfully synthesized through photomodulation of reaction mechanisms. By using an SSFL, the Mn^{4+} percentage could be maximized, and MnO_2 is the primary component of LIG/manganese oxide.

In our work, through the detailed analysis of XPS and XRD, we found that the shaped femtosecond laser can regulate the synthesis of materials, control photoinduced and/or photothermal-induced reduction/oxidation and the valence state of manganese. Altering the laser fluence also can obtain the heterogeneous junctions of different compositions.

Modifications

The added part is inserted following Page 9, Paragraph 1, Line 1 is as following:

"During the SSFL reduction process, the effect of photothermal reduction/oxidation became increasingly apparent as the laser fluence was increased. By altering the laser fluence, LIG/Manganese oxides were successfully synthesized through photomodulation of the reaction mechanisms (photochemical and photothermal reduction/oxidation). We varied the laser fluence (170-290 mJ cm^{-2}) of the SSFL to investigate differences in conductivity and electrochemical characterization and identify the optimal reduction/oxidation effect. The resistance and conductivity of the material reached their minimum and maximum, respectively, when the laser fluence was 210 mJ cm^{-2} (Supplementary Fig. 7 and 8), which implies that the LIG/Manganese oxides composite has large potential capacitance[43]. As expected, the measured area-specific capacitance of the LIG/MnO_2 MSC was highest under the laser fluence of 210 mJ cm^{-2} (Supplementary Fig. 9). We investigated whether the high-performance electrode materials were successfully synthesized under the interaction of photochemical and photothermal reduction/oxidation at this laser fluence.

The X-ray diffraction (XRD) patterns of the as-prepared GO and LIG/MnO_2 nanocomposite synthesized under the laser fluence of 210 mJ cm^{-2} were analysed (Fig. 3b). The most intensive peak of GO at $2\theta = 11.2°$ corresponds to the (001) reflection. The diffraction peaks of LIG/Manganese oxides appeared at 2θ = 12.73°, 18.14°, 28.82°, 37.46°, 41.95°, 49.86°, 56.32°, 60.27°, 65.11°, 69.76° and 72.73°, which correspond to the expected diffraction peaks of (110), (200), (310), (211), (301), (411), (600), (521), (002), (541) and (312) crystal planes of α-MnO_2 standard data, respectively, following the Joint Committee on Powder Diffraction Standards card PDF file no. 44-0141. We then collected XRD patterns of different composites (LIG/MnO_2) synthesized under different laser fluence (Supplementary Fig. 10). The different XRD patterns exhibited similar peaks, which could be all well indexed to plane of the α-MnO_2 structure. However, when the laser fluence was considerably

lower or higher than 210 mJ cm^{-2}, one or two other weak peaks appeared, and the peak intensities in the XRD patterns were lower.

The Raman spectra were extremely similar when the laser fluence was between 170 and 290 mJ cm^{-2} (Supplementary Fig. 11). We compared the Raman spectra in three cases-untreated, fs-laser modified, and fs-laser reduced-on the basis of the laser fluence (Fig. 3c). Clear G bands were characteristics of sp^2 hybridized C–C bonds at 1580 cm^{-1}. D bands of residual oxygen functional groups and other defects[44] in the graphite layer were detected at 1350 cm^{-1}, and the high intensity of G peak demonstrated a high C–C bond ratio, confirming the formation of new graphite domains. The peak value at 641 cm^{-1} was caused by Mn-O vibrations perpendicular to the direction of the MnO_6 octahedral double chains of MnO_2, indicating the presence of MnO_2 in the mixed electrode material[45]."

The added part is inserted following Page 9, Paragraph 2, Line 6 is as following:

"X-ray photoelectron spectroscopy (XPS) was used to assess the difference before (Supplementary Fig.12) versus after SSFL ablation under a laser fluence of 210 mJ cm^{-2} (Fig. 3d-f). The C 1s spectrum consisted of three peaks: C–C/C=C (284.8 eV), C–O (286.7 eV), and C=O (288.8 eV). After SSFL ablation, the C–O and C=O bonds of LIG/MnO_2 decreased, indicating that certain oxygen-containing groups on the GO hybrid film were removed during the reduction. The O 1s and Mn 2p spectrum further confirmed the presence of MnO_2. Fig. 3g illustrates the peaks centered at 530.9 and 532.3 eV, which correspond to the O–Mn and O–C bonds, respectively[46]. The peaks centered at 653.8 eV could be assigned to the Mn $2p_{1/2}$, and Mn $2p_{3/2}$ peaks were observed at three peaks: 641.2, 642.5, and 646, corresponding to +3, +4 and +2 Mn cations, respectively[47] (Fig. 3f). We also obtained high resolution Mn 2p spectra of LIG/MnO_2 under various laser fluence (Supplementary Fig 13). Supplementary Table presents the characteristic peaks and corresponding percentages of Mn^{2+}, Mn^{3+} and Mn^{4+}. The percentage of the Mn^{4+} and Mn^{3+} are the highest (79.4%) and lowest (4%) when the laser fluence was 210 mJ cm^{-2}. The percentage of manganese in different valence states could also be adjusted by altering the laser fluence. Mn 3s is more sensitive to the average oxidation state of manganese than Mn 2p was. The energy

separation between the two peaks (ΔE) was closely related to the mean manganese oxidation state[48,49]. The Mn 3s XPS spectra (Supplementary Fig. 14) were used to assess the ΔE of the LIG/MnO_2 synthesized under various laser fluence. When the laser fluence is 210 mJ cm^{-2}, the ΔE is 4.75, which is very close to the value of MnO_2."

The corresponding content in Supporting Information is as following:

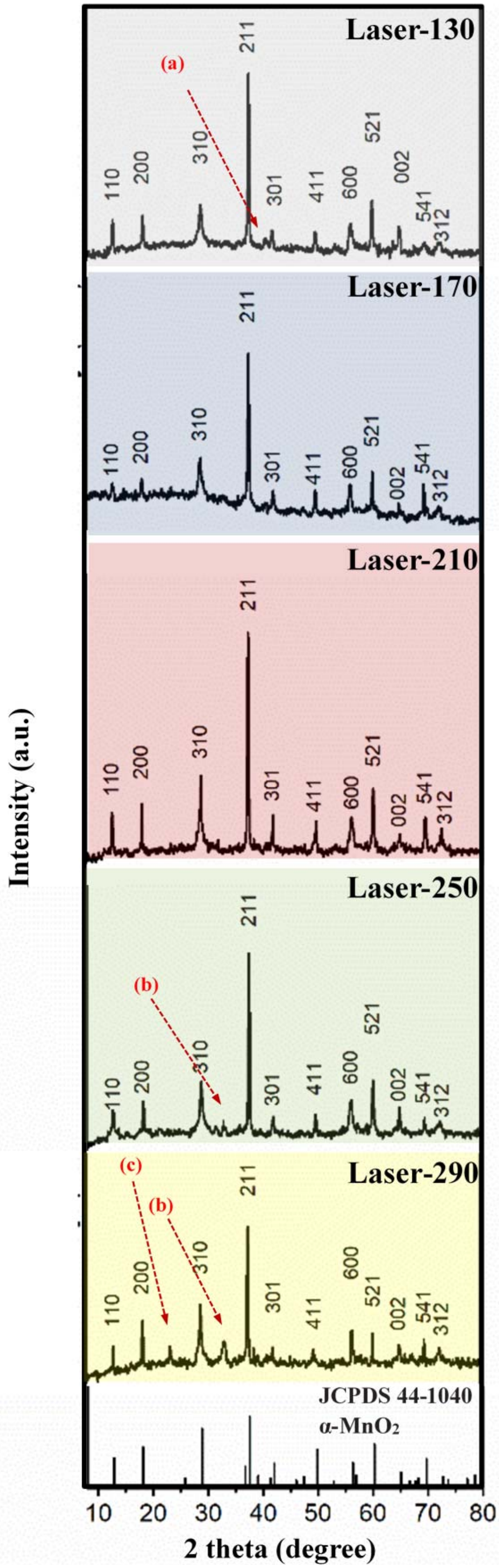
Laser-130
(a)
211
110
200
310
301
411
600
521
002
541
312
Laser-170
Laser-210
Laser-250
(b)
Laser-290
(c)
JCPDS 44-1040
α-MnO₂
Intensity (a.u.)
10
20
30
40
50
60
70
80
2 theta (degree)

Supplementary Figure 10: XRD patterns of LIG/Manganese oxides processed under different femtosecond laser fluences. We defined the composite materials synthesized under different laser fluences as Laser-130, 170, 210, 250 and 290 LIG/Manganese oxide, corresponding to laser fluences of 130, 170, 210, 250, and 290 mJ/cm^2, respectively. By altering the laser fluence, the diffraction peaks of LIG/Manganese oxides correspond to the expected diffraction peaks of the (110), (200), (310), (211), (301), (411), (600), (521), (002), (541) and (312) crystal planes of α-MnO_2 standard data. However, in the XRD pattern of Laser-130 LIG/Manganese oxide, a peak (a) appeared at $2\theta = 40.5°$ as shown in the figure. This corresponds to the expected diffraction peaks of the (200) crystal plane of MnO standard data, following the JCPDS card PDF file no. 74-4748. This is lower than other peaks, and gradually disappears as the laser fluence increases. When the laser fluences are 250 and 290 mJ/cm^2, peaks (b) and (c) appear at $2\theta = 33.05°$ and 23.12°, respectively. These were assigned to the (222) and (211) crystal planes of α-Mn_2O_3, following the JCPDS card PDF file no. 41-1442. The resulting diffractogram concludes the formation of α-MnO_2 (major) and α-Mn_2O_3 (minor) phases. This may be due to the conversion of MnO_2 to Mn_2O_3 as the laser fluence increases. MnO_2 continues to constitute an extremely high proportion of our laser-induced synthesis materials, especially when the laser fluence is 210 mJ/cm^2.

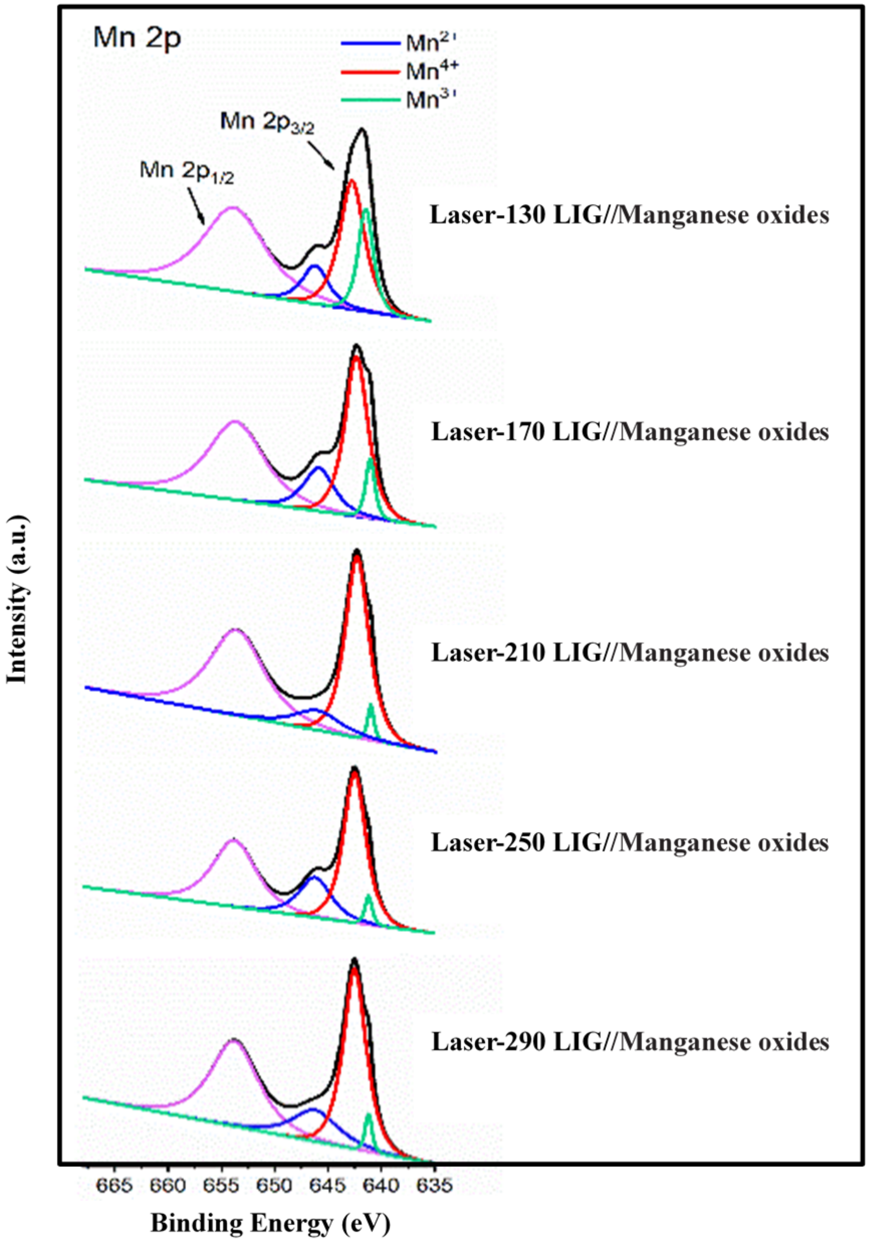

Supplementary Figure 13: XPS spectra of Mn 2p for Laser-130LIG/Manganese oxide, Laser-170LIG/Manganese oxide, Laser-210 LIG/Manganese oxide, Laser-250 LIG/Manganese oxide, and Laser-290LIG/Manganese oxide. To demonstrate that femtosecond laser irradiation can be used to control the synthesis of manganese oxide, we performed XPS measurements to investigate the manganese oxide and graphene composites. The figure displays the high-resolution XPS spectra of Mn 2p spectra for Laser-130 LIG/Manganese oxide, Laser-170LIG/Manganese

oxide, Laser-210 LIG/Manganese oxide, Laser-250LIG/Manganese oxide, and Laser-290LIG/Manganese oxide. Because the duration of the ultrashort pulse was smaller than the time scale of most of the physical and chemical characteristics, this resulted in chemical reaction pathways; chemically selective molecular excitation can be effectively controlled by a femtosecond laser. The Mn 2p XPS spectrum exhibited three characteristic peaks at 641.2, 642.5, and 646 eV, corresponding to spin–orbit peaks of manganese with mixed valence states of +3, +4, and +2, respectively. When the fluence of the laser increased to 130 mJ/cm^2, the percentage of Mn^{4+} in the LIG/Manganese oxides exceeded 50%. The percentage of Mn^{3+} in the LIG/Manganese oxides materials remained high when the percentage of Mn^{2+} was at its lowest. As the fluence of the laser gradually increases, the percentage of manganese in different valence states changes.

Supplementary Table 2. Summary of the XPS data for the LIG/Manganese oxides induced by different laser fluence

Material	Mn^{4+}2p [eV]	Mn^{2+} 2p [eV]	Mn^{3+}2p [eV]
Laser-130LIG/Manganese oxides	642.4(53.9%)	646.0(15.5%)	641.1(30.6%)
Laser-170LIG/Manganese oxides	642.6(66.4%)	646.1(23.7%)	641.2(9.9%)
Laser-210LIG/Manganese oxides	642.5**(79.4%)**	646.0**(16.6%)**	641.1**(4.0%)**
Laser-250LIG/Manganese oxides	642.6(69.1%)	646.2(26.7%)	641.2(4.2%)
Laser-290LIG/Manganese oxides	642.6(71.4%)	646.2(24.2%)	641.2(4.2%)

Supplementary Table 2. Characteristic peaks and percentages of Mn^{2+}, Mn^{3+} and Mn^{4+}. The Mn 2p spectra of LIG/Manganese oxides photoinduced by lasers with different fluences indicate that the electron BE assigned to Mn^{2+}, Mn^{3+} and Mn^{4+} 2p is close to 646.0 eV, 641.2 eV, and 642.5 eV, respectively. The percentages of Mn^{4+} and Mn^{3+} were the highest (79.4%) and lowest (4%) when the laser fluence was 210 mJ/cm^2. Conversely, the percentage of Mn^{2+} ions decreased from 23.7% to 16.6% and then increased from 16.6% to 26.7% as the laser fluence increased. Altering the laser

fluence thus enables various material compositions of LIG/Manganese oxides to be successfully synthesized through photomodulation of the reaction mechanisms. The percentage of manganese in different valence states can be adjusted flexibly.

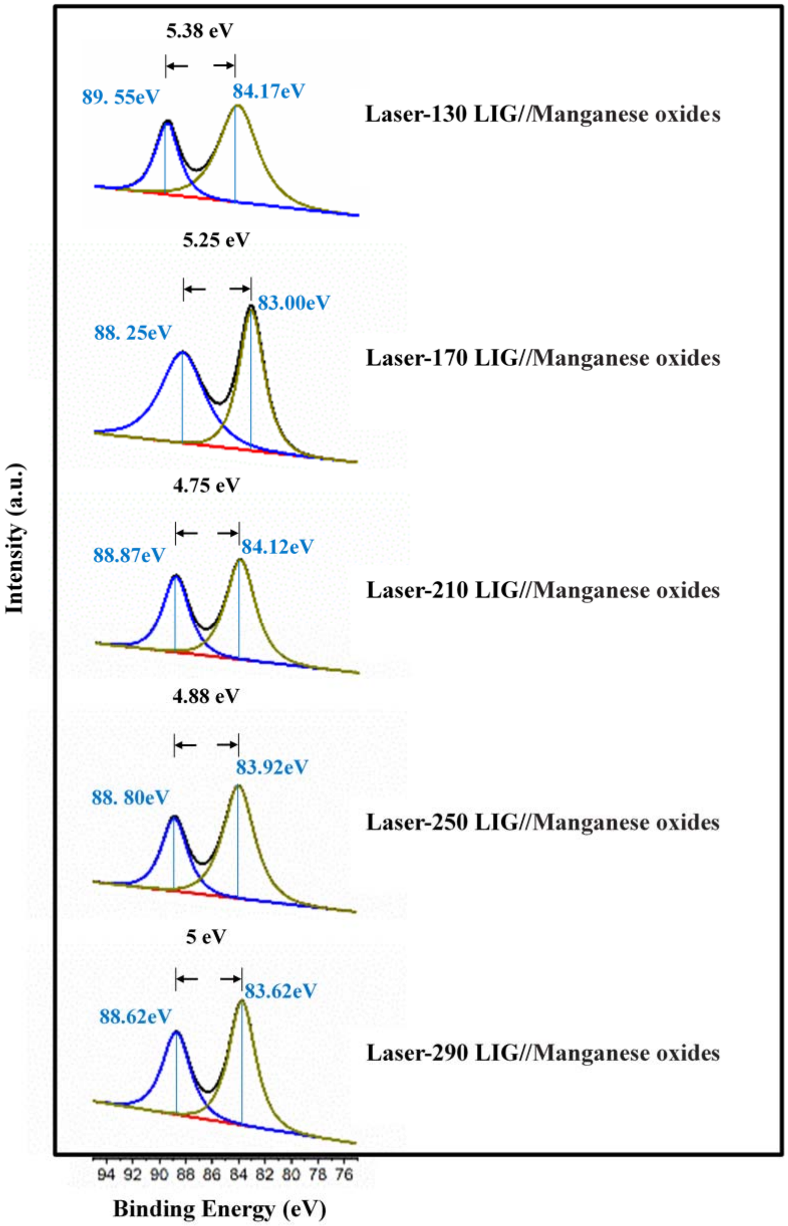

Supplementary Figure 14: XPS spectra of Mn 3s for Laser-130, 170, 210, 250 and 290 LIG/Manganese oxide. Mn 3s is more sensitive to the average oxidation state of manganese than Mn 2p is. The energy separation between the two peaks (ΔE) is closely related to the mean manganese oxidation state. The Mn 3s XPS spectra reveals that the ΔE values of a Laser-130LIG/Manganese oxide, Laser-170LIG/Manganese

oxide, Laser-210 LIG/Manganese oxide, Laser-250LIG/Manganese oxide, and Laser-290LIG/Manganese oxide are 5.38, 5.25, 4.75, 4.88, and 5.0 eV, respectively The position of the lower binding energy peak gradually shifts to higher energy as the oxidation number of the sample increases, which is consistent with previous results. The standard ΔE values for MnO_2, Mn_2O_3, and MnO are 4.7, 5.2, and 5.8eV, respectively. When the laser fluence was 210 mJ/cm^2, ΔE was 4.75, which is extremely close to the MnO_2 value. As the laser fluence increases or decreases, the value of ΔE increases, which indicates the presence of manganese oxides of lower-valence states. There are typically defects in the interlayers, such as vacancies and Mn^{4+} substitution by $Mn^{3+}$and Mn^{2+}. This result is consistent with those discussed earlier.

2.4 Reviewer's Comments

2.4. "Although the laser induced fabrication of metal oxides is not new, the authors should provide more details about the mechanism(s) related to the transition from Mn^{2+} to MnO_2."

Our Response

The detailed comments are valuable. We thank the reviewer for providing advice to improve the paper's quality.

Laser radiation is characterized by its highly coherent, responsive, and intense nature. Furthermore, laser radiation can be delivered in short pulses[R12]. In particular, the femtosecond laser has ultrahigh peak powers (>10^{13} w cm^{-2}) and ultrashort irradiation periods. Femtosecond laser radiation exhibits nonlinear nonequilibrium processing. Thus, materials subjected to intense laser pulses exhibit considerable structure and compositional transformations that are often not in thermodynamic equilibrium[R13-17]. These phase changes can be induced by either high-temperature thermal cycles or through direct photoreactions (or their combination). Laser irradiation can induce the development of diverse types of functional metal oxides from metal ion precursors[R18-22]. Femtosecond laser fabrication has a high degree of

certainty and repeatability. The intensity of the femtosecond pulse provides sufficient kinetic energy to cause lattice defects. However, as the number of femtosecond laser pulses increases, the thermal effect becomes apparent, resulting in photothermal fractures of carbon bond structures and an increase in lattice defects. Accumulated energy provokes the metal ion precursor to bond to oxygen, leading to the formation of metal oxide. However, the accumulation of laser pulses, which increases the effective annealing time, leads to further diffusion processes and subsequent improvement of the quality of metal oxide[R12].

First, we fully mixed GO with the precursor of manganese ion (Mn^{2+}). Then, we used femtosecond laser irradiation to complete GO reduction and synthesized manganese dioxide in one step. Thus, this process promoted the transition from Mn^{2+} to MnO_2. The mechanisms of photoreduction of GO in our study were divided into two main categories: photochemical reduction and photothermal reduction. In both categories, Mn^{2+} and GO mutually promote the synthesis reaction of the LIG/MnO_2 composite.

We proposed a photosynthetic method for synthesizing LIG–MnO_2. Figure R2.13 illustrate the physics and chemical mechanisms of GO reduction and MnO_2.

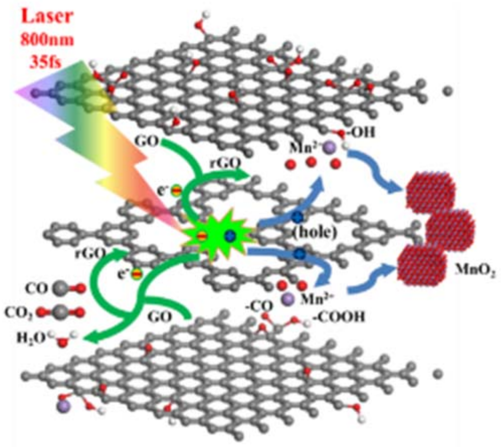

Figure R2.13 Schematic of the resultant morphology change and phase transformation by a femtosecond laser under the proposed photosynthetic routes

The thermal deoxygenation of GO was initiated using a small amount of heat. The temperature and energy increase corresponded to the energy released during the reduction of GO and propagated and sustained GO reduction[R26]. The energy released

from the deoxygenation of the GO functioned as an effective in situ power source that could drive the oxidation reaction of Mn^{2+} to MnO_2. The amount of heat released during the deoxygenation reaction of GO is several times the amount of heat required to drive the endothermic oxidation of metal ions[R27,28].

The equations for the reaction are expressed as follows:

$$GO+h\Upsilon \rightarrow rGO\ (h^{+}+e^{-}) \quad (E2\text{-}1)$$

$$Mn^{2+} \rightarrow Mn^{4+}+2e^{-} \quad (E2\text{-}2)$$

$$(GO)\text{-}OH+(GO)\text{-}COOH+Mn^{2+}+e^{-} \rightarrow rGO+MnO_2+CO_2\uparrow+H_2O\uparrow \quad (E2\text{-}3)$$

$$(GO)\text{-}O\text{-}(GO)+(GO)\text{-}COOH+Mn^{2+}+e^{-} \rightarrow rGO+MnO_2+CO\uparrow \quad (E2\text{-}4)$$

As the reaction continues, the photothermal reduction gradually emerges. The cumulative effect of the pulsed laser is bound to produce an amount of heat. It results in the pyrolysis reaction of GO, in which oxygen-containing functional groups such as hydroxyl (-OH), carboxyl (-COOH), and oxygen Bridges (C-O-C). They are break up into CO, CO_2 and H_2O, which can be removed[R29,30]. When the GO is cracked, the energy and accumulated heat in the reaction provide the original energy for oxidation to the Mn^{2+}, thus completing the formation of manganese oxide

$$GO+Mn^{2+} \xrightarrow{\Delta} rGO+MnO_2+CO_2\uparrow+CO\uparrow+H_2O\uparrow \quad (E2\text{-}5)$$

The high power of the shaped femtosecond laser we used was sufficient to irradiate GO at a local scale many times. This process also resulted in the photothermal reduction of GO and a porous graphene structure.

We proposed a novel photosynthetic mechanism for synthesizing LIG–MnO_2. In this mechanism, Mn^{2+} to MnO_2 transitions facilitates the reduction of GO to LIG. GO provides not only the energy required for Mn^{2+} oxidation but also a strong attachment point for MnO_2. Thus, high-quality MnO_2 nanoparticles were fabricated and doped with LIG to form a composite material with a firm structure.

Modifications

According to the comments of the reviewer, we have revised the corresponding part in our manuscript.

The original part form Page 3, Paragraph 2, Line 7 to Line 20 is as flowing:

"The SFLS strategy can simultaneously realize high-precision and ultra-efficient processing of large-scale multiple L-S-MSCs. Benefiting from the straightforward technology, the efficiency of processing L-S-MSCs has been unprecedentedly improved, and thousands of flexible L-S-MSCs could be precisely fabricated in 1 minute. Due to the special uniform light field effect brought by the spatially shaped femtosecond (ss-fs) laser, we have created the structure different from Gaussian light processing which is conducive to the formation of three-dimensional structures and avoid the incomplete processing caused by the uneven light intensity regionalization. Therefore, we transiently synthesize LIG with the uniformly distributed MnO_2 nanoparticles in situ, and simultaneously pattern the novel fluffy and porous three-dimensional composite structures with ultrahigh specific surfacearea and toughness. Mn2+ can facilitate the reduction process of graphene oxide (GO) due to the higher oxidation potential[22,23] and use the anchor sites provided by GO to be oxidized to MnO_2 nanoparticle in situ[24]. In this way, the fabricated LIG/MnO_2 L-S-MSCs are collector-free, arbitrary graphics, ultra-flexible, high-resolution, tens of micrometers in size with high specific capacitance and ultrahigh energy density."

These parts are revised as following:

"The SSFL strategy can perform high-precision and ultraefficient processing of large-scale multiple MSCs in one step. Femtosecond lasers are uniquely characterized by their ultrahigh peak power (>10^{13} w cm^{-2}) and ultrashort irradiation period[22,23]. The composites of LIG/MnO_2 are synthesized through photomodulation of the reaction mechanisms (photochemical and photothermal reduction/oxidation)[24]. Mn^{2+} can facilitate the graphene oxide (GO) reduction process because of its high oxidation potential[25,26], and it can use the anchor sites provided by GO to be oxidized to MnO_2 nanoparticle in situ[27]. At the same time, a fluffy porous three-dimensional structure with ultrahigh specific surface area and toughness can be obtained. The process does

not require oxidants, current collectors, demanding reaction conditions, addition of chemicals or metal foam. Furthermore, this SSFL technique is particularly attractive because it is suitable for numerous material systems benefiting from the universality of materials for femtosecond laser processing. The SSFL strategy has ultrahigh efficiency and consistently fabricates high-performance, high-resolution, and flexible MSCs, which is promising for application to advanced miniaturized electronics, such as microelectromechanical systems. This strategy also provides a pathway for high throughput in the industry and designable large-scale flexible energy storage devices."

The original part in Page 10, Figure 3 (figure 3i):

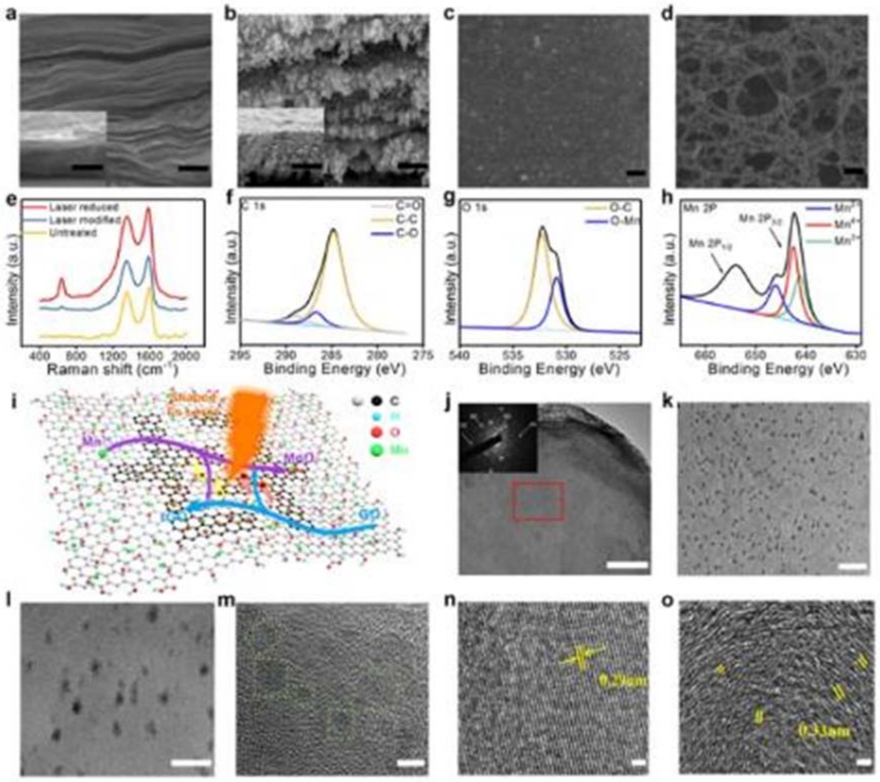

"**Fig. 3 |LIG/MnO$_2$ electrodes material processed by SFLS. a and b, SEM image of cross section view of the hybrid film before and after ss-fs laser ablation(scale bar, 100 nm；inset scale bar, 2 μm). c and d, SEM image of surface of the hybrid film before and after ss-fs laser ablation (scale bar, 500 nm). e, Raman spectra of GO hybrid film untreated, modified by ss-fs laser and reduced by ss-fs laser. f-h, C 1s, O 1s and Mn 2p XPS core level spectra of LIG/MnO$_2$ film. i, Schematic illustration showing the photosynthetic routes based on redox**

shuttle for LIG/MnO_2. j, TEM image taken at the edge of the nanocomposite (scale bar, 400 nm). k, Large area of uniform MnO_2 nanoparticles scattered on LIG (scale bar, 100 nm). l, Partially enlarged MnO_2 nanoparticles scattered on LIG (scale bar, 50nm). m, High-resolution TEM (HRTEM) image of MnO_2 nanoparticles scattered on LIG (scale bar, 10nm). n and o, HRTEM image of selected area indicated lattice space (scale bar, 2nm).."

The original part form Page 11, Paragraph 1, Line 1 to Line 10 is as flowing:

"Fig. 3 proposed an enlightening photosynthetic route for synthesizing LIG/MnO_2 at the atomic level. From the schematic diagram, we can clearly observe that in the area irradiated by ss-fs laser, the carbon-oxygen bond is significantly reduced, and a large amount of MnO_2 is generated. In the areas without laser irradiation, more carbon-oxygen bonds and Mn^{2+} exist on GO film. Therefore, the role of ss-fs lasers in synthesizing materials and building three-dimensional structures is very obvious. The ss-fs laser provides a large amount of photon energy, which promotes the autocatalysis of GO, which in turn produces electrons for the reduction of GO. Mn^{2+} will be absorbed on the negatively charged part of the hydrophilic oxygen functional groups of GO. During the reduction of graphene oxide, Mn^{2+} undergo oxidation to become MnO_2 using the energy and oxygen functional groups provided by the GO deoxidation process."

These parts are revised as following:

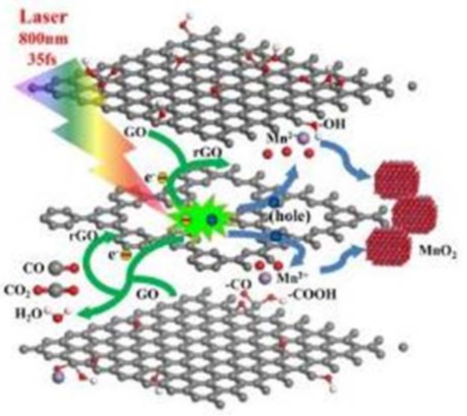

Figure 3a Schematic of the resultant morphology change and phase transformation by SSFL under the proposed photosynthetic routes

Fig. 3a illustrates our proposed photosynthetic route for synthesizing LIG/MnO_2.

SSFL provides numerous excited photons that form free-moving electrons and holes, which reduce GO to rGO. Under their action, GO was reduced to rGO. Mn^{2+} is absorbed on the negatively charged part of the hydrophilic oxygen functional groups of GO because of the electrostatic effect. GO-wrapped Mn^{2+} then facilitates the reduction of GO caused by the high oxidation potential and then uses the anchor sites provided by GO to be oxidized into manganese oxide nanoparticles in situ. The energy released from the deoxygenation of GO serves as an effective and in situ power source driving the oxidation of Mn^{2+} into MnO_2. The amount of heat released during the deoxygenation of GO is several times that required to drive the endothermic oxidation of metal ions[38,39]. The equations for the reaction is as follows,

$$\text{(GO)-OH+(GO)-O-(GO)+(GO)-COOH+Mn}^{2+}\text{+e}^- \xrightarrow{h\nu} \text{rGO+MnO}_2\text{+CO}_2\uparrow\text{+CO}\uparrow \quad (3)$$

As the reaction continues, the photothermal reduction gradually emerges, and the cumulative effect of the pulsed laser produces heat, which results in the pyrolysis of GO, whereby oxygen-containing functional groups such as hydroxyl (-OH), carboxyl (-COOH), and oxygen Bridges (C-O-C) are break up into CO, CO_2 and H_2O, which can be removed[40]. When the GO is cracked, the energy and accumulated heat in the reaction provide the original energy for oxidation to the manganese ions, thus completing the formation of manganese oxide.

$$\text{GO+Mn}^{2+} \xrightarrow{\Delta} \text{rGO+MnO}_2\text{+CO}_2\uparrow\text{+CO}\uparrow\text{+H}_2\text{O}\uparrow \quad (4)$$

Therefore, the SSFL reduction in the experiment was caused by the combined effect of photochemical and photothermal reactions. Our findings indicated that gas was released when the SSFL reduced GO composite film, which is crucial in the formation of 3D porous structure composites. The 3D porous patterned structure fabricated in one step using the SSFL employed graphene as a solid skeleton, and the uniform effect of the light field enabled manganese dioxide nanoparticles to be evenly distributed on the graphene skeleton. The pores functioned as shortcuts for rapid ion diffusion between graphene layers, which increased the speed of ion transport across the compressed film. The 3D porous scaffold was very stable, which would mitigate

mechanical stress within the electrode and thus ensure the stability of long-term cycling of electrochemical energy storage systems[41,42]. Therefore, the LIG/MnO_2 composite had improved capacitance."

The original part in Page 9, Paragraph 1:

"**Analytical characterization of the LIG/MnO_2.** Fig. 3a and b depict the cross-sectional view of the hybrid film before and after the ss-fs laser patterning. The original graphene oxide layers were stacked tightly together. Interestingly, after ss-fs laser ablation, the lamellar graphene fluffed up and many MnO_2 nanoparticles were attached to LIG. The SEM revealed the surface of the graphene film also became porous and fluffier after ss-fs laser ablation (Fig. 3c and d). The fluffy and wrinkled structure resulted in larger specific surface area, fast ion transport, and excellent electrochemical performance."

These parts are revised as following:

"**Mechanism and analytical characterization of LIG/MnO_2 3D composite synthesis using the SSFL.** The mechanisms of laser reduction presented in this study fall into two main categories: photochemical and photothermal reduction/oxidation. In the initial stage of SSFL interaction with materials, the photochemical reduction/oxidation induces nonthermal ultrafast electron excitation, nonlinear absorption, and subsequent oxygen group removal because of the ultrashort pulse width and ultrahigh intensity of the SSFL. The instantaneous intensity of the SSFL provides sufficient kinetic energy to enable oxygen atoms to leave the graphene sheet without damaging it[37]."

The added part is inserted following Page 9, Paragraph 1, Line 1 is as following:

"During the SSFL reduction process, the effect of photothermal reduction/oxidation became increasingly apparent as the laser fluence was increased. By altering the laser fluence, LIG/Manganese oxides were successfully synthesized through photomodulation of the reaction mechanisms (photochemical and photothermal reduction/oxidation). We varied the laser fluence (170-290 mJ cm^{-2}) of the SSFL to investigate differences in conductivity and electrochemical characterization and identify the optimal reduction/oxidation effect. The resistance

and conductivity of the material reached their minimum and maximum, respectively, when the laser fluence was 210 mJ cm^{-2} (Supplementary Fig. 7 and 8), which implies that the LIG/Manganese oxides composite has large potential capacitance[43]. As expected, the measured area-specific capacitance of the LIG/MnO_2 MSC was highest under the laser fluence of 210 mJ cm^{-2} (Supplementary Fig. 9). We investigated whether the high-performance electrode materials were successfully synthesized under the interaction of photochemical and photothermal reduction/oxidation at this laser fluence.

The X-ray diffraction (XRD) patterns of the as-prepared GO and LIG/MnO_2 nanocomposite synthesized under the laser fluence of 210 mJ cm^{-2} were analyzed (Fig. 3b). The most intensive peak of GO at $2\theta = 11.2°$ corresponds to the (001) reflection. The diffraction peaks of LIG/Manganese oxides appeared at 2θ = 12.73°, 18.14°, 28.82°, 37.46°, 41.95°, 49.86°, 56.32°, 60.27°, 65.11°, 69.76° and 72.73°, which correspond to the expected diffraction peaks of (110), (200), (310), (211), (301), (411), (600), (521), (002), (541) and (312) crystal planes of α-MnO_2 standard data, respectively, following the Joint Committee on Powder Diffraction Standards card PDF file no. 44-0141. We then collected XRD patterns of different composites (LIG/MnO_2) synthesized under different laser fluence (Supplementary Fig. 10). The different XRD patterns exhibited similar peaks, which could be all well indexed to plane of the α-MnO_2 structure. However, when the laser fluence was considerably lower or higher than 210 mJ cm^{-2}, one or two other weak peaks appeared, and the peak intensities in the XRD patterns were lower.

The Raman spectra were extremely similar when the laser fluence was between 170 and 290 mJ cm^{-2} (Supplementary Fig. 11). We compared the Raman spectra in three cases-untreated, fs-laser modified, and fs-laser reduced-on the basis of the laser fluence (Fig. 3c). Clear G bands were characteristics of sp^2 hybridized C–C bonds at 1580 cm^{-1}. D bands of residual oxygen functional groups and other defects[44] in the graphite layer were detected at 1350 cm^{-1}, and the high intensity of G peak demonstrated a high C–C bond ratio, confirming the formation of new graphite domains. The peak value at 641 cm^{-1} was caused by Mn-O vibrations perpendicular

to the direction of the MnO_6 octahedral double chains of MnO_2, indicating the presence of MnO_2 in the mixed electrode material[45]."

The added part is inserted following Page 9, Paragraph 2, Line 6 is as following:

"X-ray photoelectron spectroscopy (XPS) was used to assess the difference before (Supplementary Fig.12) versus after SSFL ablation under a laser fluence of 210 mJ cm^{-2} (Fig. 3d-f). The C 1s spectrum consisted of three peaks: C–C/C=C (284.8 eV), C–O (286.7 eV), and C=O (288.8 eV). After SSFL ablation, the C–O and C=O bonds of LIG/MnO_2 decreased, indicating that certain oxygen-containing groups on the GO hybrid film were removed during the reduction. The O 1s and Mn 2p spectrum further confirmed the presence of MnO_2. Fig. 3g illustrates the peaks centered at 530.9 and 532.3 eV, which correspond to the O–Mn and O–C bonds, respectively[46]. The peaks centered at 653.8 eV could be assigned to the Mn $2p_{1/2}$, and Mn $2p_{3/2}$ peaks were observed at three peaks: 641.2, 642.5, and 646, corresponding to +3, +4 and +2 Mn cations, respectively[47] (Fig. 3f). We also obtained high resolution Mn 2p spectra of LIG/MnO_2 under various laser fluence (Supplementary Fig 13). Supplementary Table presents the characteristic peaks and corresponding percentages of Mn^{2+}, Mn^{3+} and Mn^{4+}. The percentage of the Mn^{4+} and Mn^{3+} are the highest (79.4%) and lowest (4%) when the laser fluence was 210 mJ cm^{-2}. The percentage of manganese in different valence states could also be adjusted by altering the laser fluence. Mn 3s is more sensitive to the average oxidation state of manganese than Mn 2p was. The energy separation between the two peaks (ΔE) was closely related to the mean manganese oxidation state[48,49]. The Mn 3s XPS spectra (Supplementary Fig. 14) were used to assess the ΔE of the LIG/MnO_2 synthesized under various laser fluence. When the laser fluence is 210 mJ cm^{-2}, the ΔE is 4.75, which is very close to the value of MnO_2."

2.5 Reviewer's Comments

2.5. "The conductivity of 3.2 S m^{-1} of the laser induced graphene is moderate. However, for the MSC, the measured time constant of 10.6 μs, is extremely short time.

The author should provide more explanation about how such a fast time constant is possible with such relatively poor conductivity."

Our Response

We truly appreciate the reviewer's suggestion regarding conductivity and time constants. The reviewer's valuable advice is crucial for improving the quality of the paper.

The theoretical conductivity of monolayer graphene materials is high, which is a crucial concern in electrochemical devices. However, GO material has poor electrical conductivity. Therefore, GO films are reduced to LIG films to improve electrical conductivity[R31-34]. However, in graphene films, because layers are stacked on top of each other, it is impossible to accurately measure the conductivity of the surface.

In our study, when we used the shaped femtosecond laser to reduce GO, laser irradiation on GO produced deoxygenated GO (or LIG) with carbon structures tunable to defect concentration by the amount of laser pulses impingement; higher fluence results in an severely damaged carbon morphology with low electrical conductivity. The controlled low fluence of the femtosecond laser was sufficient to remove oxygen species and drastically increase the conductivity of the sample by several orders of magnitude with negligible damage to the C–C network. Therefore, the optimal laser fluence parameters were selected by measuring the conductivity of different laser fluence values. Notably, 3.2 S m^{-1} is the bulk conductivity and not the surface conductivity of laser-induced graphene. The thickness of the converted region can be as small as 30 nm (estimated by simulations) on an overall thickness of 3000 nm. Thus, our measurements were an underestimation of actual conductivity values[R35].

A precision probe station (MPS-100S) with a microscopic system was used for conductivity measurements. The tungsten probes (tip diameter ~5 μm) were used to ensure accurate contact with the electrode material. Measurements were performed by varying the applied voltage between −4.0 and +4.0 V. The studied patterns were 100 μm × 100 μm, and the probes were placed 80 μm apart. Bulk conductivity was evaluated using the following equation:

$$\sigma = \frac{I}{RA} \tag{E2-4}$$

where R is the resistance for the voltage current plots (Supplementary material), I is the distance between the probe tips (80 μm), and A is the cross-sectional area of the pattern (100 μm × 3 μm). By measuring the bulk conductivity as a reference, we obtained the optimal selection of laser parameters by conducting experiments.

The reviewer mentioned "the measured time constant of 10.6 μs is an extremely short time." However, the time constant (τ0) of 10.6 μs could be measured from the curves of the phase angle versus frequency (Figure 4i in the manuscript). Several methods of achieving fast time constant MSCs were proposed in our study. In addition to the underestimated conductivity mentioned earlier, the main concerns regarding time constants are as follows:

Ultrafast time constant supercapacitors are useful for many practical applications, such as the filtering application[R36]. Few materials have satisfied this rigorous requirement in previous supercapacitor studies because both the electron adsorption in electric double layer and the electrochemical reaction in the pseudocapacitor are considerably slower than the electron storage in traditional capacitors[R37].

1) In our study, we fabricated a small (approximately 50 × 50 μm^2) MSC. Such a MSC has a very fast charging and discharging process. Some studies have revealed that such MSCs exhibit ultrahigh power and a submillisecond time constant. This is because of the very small amount of stored energy in MSCs, which can be quickly released[R38,39]. The effective electrode material area of miniature supercapacitors is very small. Therefore, the charge transfer and energy storage can be accomplished very quickly, which boosts the power delivery rate of the device. Therefore, such devices can be used widely in the electronic industry[R40,41].

2) Researchers have revealed that MSCs can be drastically improved by reducing the physical separation between the anode and cathode[R42,43]. The interspace between the electrodes of the MSC is a key factor affecting the time constant. According to the formula proposed by Olthuis et al[R44]

$$\tau = (R_{cc} + \kappa \times \rho_e)C \tag{E2-5}$$

where R_{cc} is the resistance of current collectors, ρ_e is the resistivity of the electrolyte, and κ is the analytical expression of the cell constant. Pech et al[R45] studied in detail the effect of interspace on the time constant and concluded that under the same size condition, the smaller the interspace between the MSC electrodes is, the shorter the time constant (Figure R2.14).

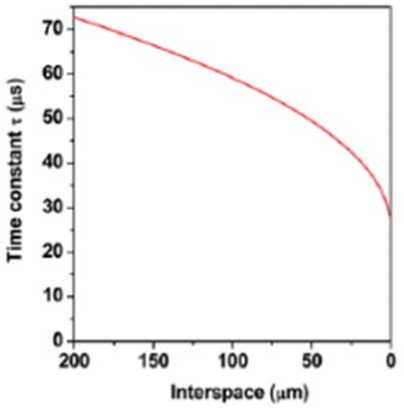

Figure R2.14 The evolution of the time constant with interspace[R43]

In our study, the interspace between the MSC electrodes was just a few microns (2–5 μm). MSCs with smaller interdigitated spacing have faster frequency response capabilities. Under the same micro size condition, the smaller the interspace the MSCs are, the shorter time constant they have. A smaller interspace reduces the transport path of ions from one electrode to the counter electrode, resulting in a reduction of the internal resistance and complete utilization of active materials.

3) The electrode materials with layered crystalline structures are promising materials for supercapacitors because of their ability to intercalate ions in a wide range of sites[R46-48]; this causes efficient transfer of ions, resulting in a very short time constant. Porous materials, especially 3D porous materials, can provide greater surface area and more pathways for ion transport[R49,50].

In our study, deoxidization and reduction of GO produced a gas, which subsequently formed a 3D porous structure. The porous laser-induced graphene/MnO_2 with a large specific surface area and multiple paths facilitated ion transmission in the electrodes. These pores functioned as shortcuts for rapid ion diffusion between graphene layers and increased the speed of ion transport across the compressed film.

As depicted in Figure R2.15, the porous structure provided a fast path for ion transfer, which enabled effective ion contact with the electrode material. This ensured rapid charging and discharging. Therefore, our MSCs exhibited an ultrashort time response (10.6 μs).

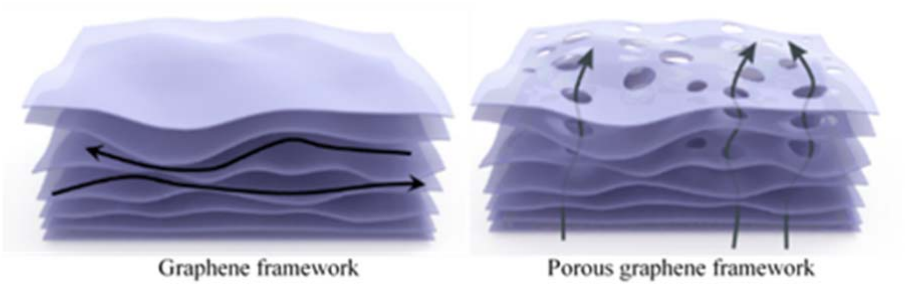

Figure R2.15 Illustration of a compressed graphene framework and porous graphene framework with arrows depicting the ion transport pathways

Modifications

According to the comments of the reviewer, we have revised the corresponding part in our manuscript.

The original part in Page 13, Paragraph 1, Line 1 to 5, the detail is as following:

"To discover the optimal reduction effect, we changed laser fluence to explore the difference in conductivity and electrochemical characterization. Supplementary Fig. 8 depicts the smallest resistance of material at a laser fluence of 210 mJ cm^{-2} under different potentials. The conductivity of the material first increased and then decreased, increased to a maximum of 3.2 S m^{-1} at 210 mJ cm^{-2} (Supplementary Fig. 9). Other studies[47] have suggested that measurements report the bulk conductivity and not the surface conductivity, due to our hybrid film being very thin, the calculated conductivity compared with traditional techniques was probably underestimated. To further explore the effects of laser fluence on materials, we performed Raman spectroscopy and the electrochemical performance of LIG/MnO_2 L-S-MSCs processed in different laser fluence (Supplementary Fig. 10). Coincidentally, the laser fluence of the best conductivity, the most obvious Raman characteristic peak and the highest area specific capacitance of the L-S-MSCs (Supplementary Fig. 11) were all

at 210 mJ cm^{-2}. The reason might be that in microelectronic devices, the L-S-MSCs with excellent electrical conductivity can achieve higher capacitance characteristics because of the rapid transfer of charge."

These parts are revised as following:

"During the SSFL reduction process, the effect of photothermal reduction/oxidation became increasingly apparent as the laser fluence was increased. By altering the laser fluence, LIG/Manganese oxides were successfully synthesized through photomodulation of the reaction mechanisms (photochemical and photothermal reduction/oxidation). We varied the laser fluence (170-290 mJ cm^{-2}) of the SSFL to investigate differences in conductivity and electrochemical characterization and identify the optimal reduction/oxidation effect. The resistance and conductivity of the material reached their minimum and maximum, respectively, when the laser fluence was 210 mJ cm^{-2} (Supplementary Fig. 7 and 8), which implies that the LIG/Manganese oxides composite has large potential capacitance[43]. As expected, the measured area-specific capacitance of the LIG/MnO2 MSC was highest under the laser fluence of 210 mJ cm^{-2} (Supplementary Fig. 9). We investigated whether the high-performance electrode materials were successfully synthesized under the interaction of photochemical and photothermal reduction/oxidation at this laser fluence.

The X-ray diffraction (XRD) patterns of the as-prepared GO and LIG/MnO_2 nanocomposite synthesized under the laser fluence of 210 mJ cm^{-2} were analysed (Fig. 3b). The most intensive peak of GO at $2\theta = 11.2°$ corresponds to the (001) reflection. The diffraction peaks of LIG/Manganese oxides appeared at 2θ = 12.73°, 18.14°, 28.82°, 37.46°, 41.95°, 49.86°, 56.32°, 60.27°, 65.11°, 69.76° and 72.73°, which correspond to the expected diffraction peaks of (110), (200), (310), (211), (301), (411), (600), (521), (002), (541) and (312) crystal planes of α-MnO_2 standard data, respectively, following the Joint Committee on Powder Diffraction Standards card PDF file no. 44-0141. We then collected XRD patterns of different composites (LIG/MnO_2) synthesized under different laser fluence (Supplementary Fig. 10). The

different XRD patterns exhibited similar peaks, which could be all well indexed to plane of the α-MnO_2 structure. However, when the laser fluence was considerably lower or higher than 210 mJ cm^{-2}, one or two other weak peaks appeared, and the peak intensities in the XRD patterns were lower.

The Raman spectra were extremely similar when the laser fluence was between 170 and 290 mJ cm^{-2} (Supplementary Fig. 11). We compared the Raman spectra in three cases-untreated, fs-laser modified, and fs-laser reduced-on the basis of the laser fluence (Fig. 3c). Clear G bands were characteristics of sp^2 hybridized C–C bonds at 1580 cm^{-1}. D bands of residual oxygen functional groups and other defects[44] in the graphite layer were detected at 1350 cm^{-1}, and the high intensity of G peak demonstrated a high C–C bond ratio, confirming the formation of new graphite domains. The peak value at 641 cm^{-1} was caused by Mn-O vibrations perpendicular to the direction of the MnO_6 octahedral double chains of MnO_2, indicating the presence of MnO_2 in the mixed electrode material[45]."

According to the reviewer's advice, we have added the detailed explanation about such a fast time constant.

The added part is inserted following Page 15, Paragraph 1, Line 4 is as following:

"The ultra-fast time constant may be attributed to several factors. First, the MSC fabricated by SSFL is tens of microns in size, and thus charge transfer and energy storage could be accomplished rapidly. Second, the smaller interspace reduced the length of the transport path of ions from one electrode to the counter electrode, resulting in reduced internal resistance and complete utilization of active materials. Third, the porous LIG/MnO_2 had a large specific surface area and multiple paths, which facilitated ion transmission in the electrodes. The pores functioned as shortcuts for rapid ion diffusion between graphene layers, increasing the speed of ion transport across the compressed film, providing faster paths, providing more path choices for ion transfer, and enabling ions to come into contact with the electrode material quickly and fully. Therefore, the high-resolution MSCs fabricated using SSFL could charge and discharge more rapidly and exhibited an ultrasmall time response (10.6

μm)."

The corresponding content added in Supporting Information is as following:

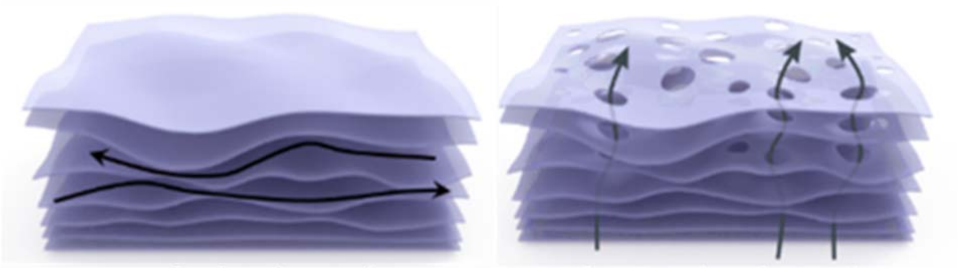

Supplementary Figure 5: Illustration of a compressed graphene framework and porous graphene framework with arrows depicting the ion transport pathways. The porous structure provided a faster path and more path choices for the ion transfer. This enabled the ions to contact the electrode material quickly and fully, thus charging and discharging more quickly. This ability to optimize charge transport is shown in Nyquist plots and Bode plots. Our MSCs exhibited an ultra-small time response (0.01 ms) and low equivalent series resistance (0.85 mΩ/cm^2).

2.6 Reviewer's Comments

2.6. "More details about the dimensional parameters, such as the interdigital gap width and the electrode and device dimensions, should be provided in the device electrochemical performance discussion. These parameters can critically affect electrochemical performance."

Our Response

Thank for your detailed comments regarding the manuscript. They have proved valuable for improving our paper.

We studied the parameters of different shapes of MSCs and the laser fluence during processing. However, these parameters are not sufficient, and more parameters should be researched to understand the chemical performance of MSCs. As recommended by the reviewer, we performed experiments on the interdigital gap width of the electrode, device dimensions, and thickness of the MSC.

(1) We explored the effect of the interdigital gap width on electrochemical performance when designing the MSCs with narrow gaps of 500 nm and 2, 4, and 6 μm (Figure R2.16).

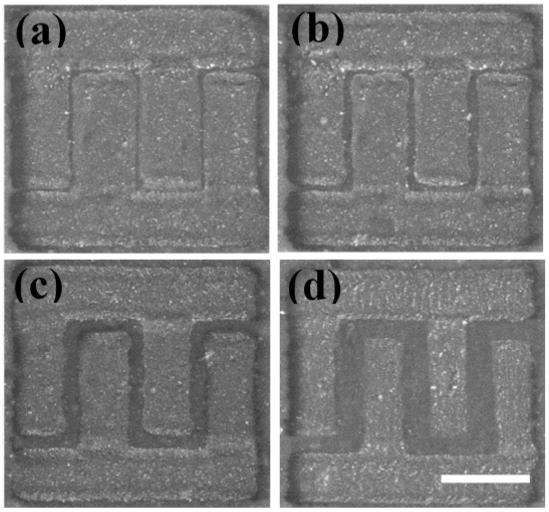

Figure R2.16 The SEM of interdigital MSCs with different narrow gaps (scale bar, 20 μm). The narrow gaps of the figures (a), (b), (c) and (d) are represented as 500 nm, 2μm, 4μm and 6μm respectively

As depicted in Figure R2.17, cyclic voltammograms (CVs) were recorded at variable scan rates to evaluate the effect of various microscale interdigital narrow gaps of MSCs. The CVs of the high-resolution MSCs maintained a rectangular shape at all scan rates. Notably, the MSCs with a narrow gap of 2 μm exhibited the best electrochemical performance.

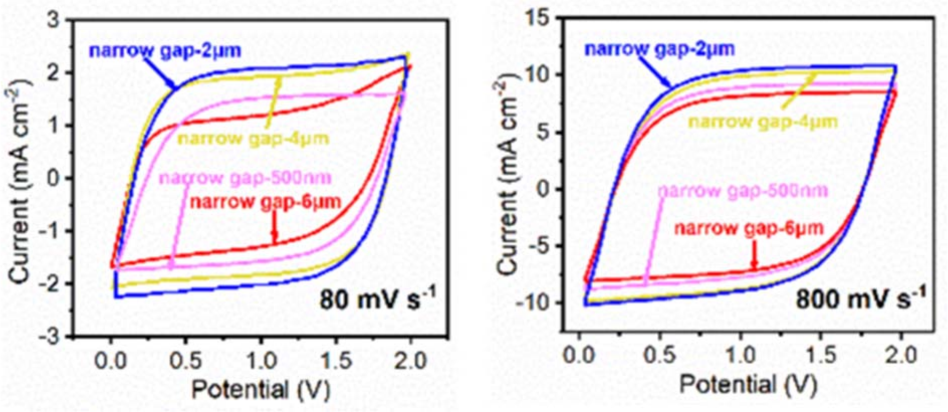

Figure R2.17 The comparison of electrochemical performances (CV characterizations at different scan rates) of the MSCs with different narrow gaps.

The narrower the gaps of electrodes are, the faster ion transfer and charge and discharge are, which considerably contributes to electrochemical performance. In the actual measurement, a gap of a few hundred nanometers can cause collision losses of electrode materials, which affects charge transfer and electrochemical performance. The electrochemical performance of MSCs with different narrow gap was plotted against various scan rates (Figure R2.18).

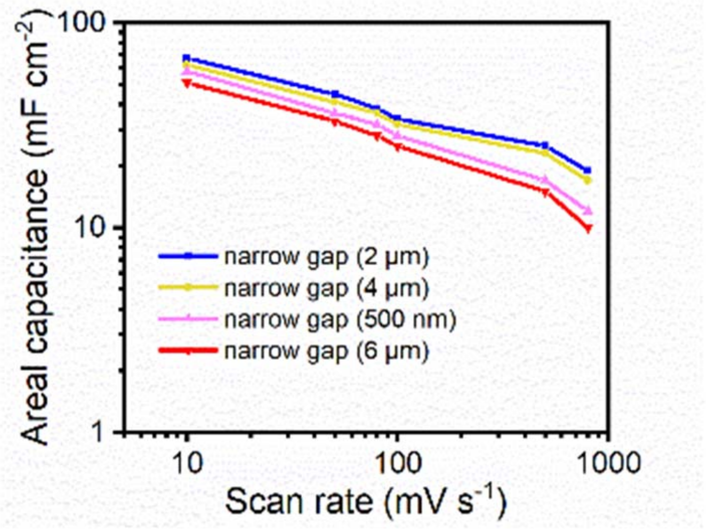

Figure R2.18 The areal capacitance of the MSCs with different narrow gaps as a function of scan rates

The results of our experiments revealed that MSCs with different narrow gaps differences from the areal capacitance in the case of a lower scan rate. When the narrow gap was 2 μm, the highest areal capacitance was 67 mF cm^{-2}. The areal capacitance was 62, 58, and 51 mF cm^{-2} at the corresponding gaps of 4 μm, 500 nm, and 6 μm.

(2) Based on reviewer's suggestion, we also tested the electrochemical performance of MSCs with the same shape and different sizes to investigate the influence of device dimensions on electrochemical performance. Our technique allowed the ultrafast fabrication of complete MSCs, ranging in size from 15 × 15 μm^2 to 100 × 100 μm^2, in one step. To analyze the effect of device dimensions on electrochemical performance, we selected MSCs of three sizes (15 × 15, 50 × 50, and 100 × 100 μm^2) for the test. For accurate measurement and for measurement of the

size of a dozen micron miniature supercapacitor requirements, we customized a probe with a contact diameter of 3 microns for use with a 100× confocal microscopy system.

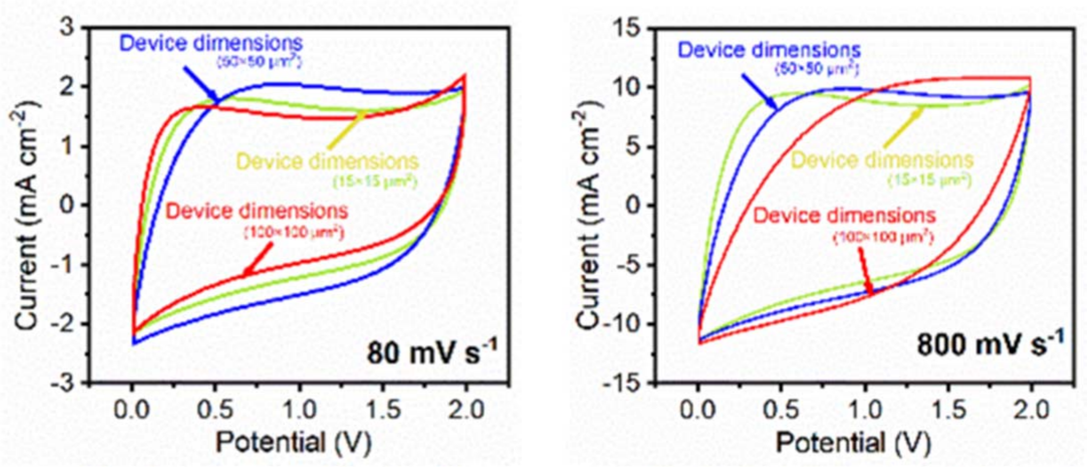

Figure R2.19 The comparison of electrochemical performances (CV characterizations at diffenrent scan rates) of the MSCs with different device dimensions.

We evaluated the electrochemical behavior of MSC at different scan rates. Figure R2.19 depicts the CV curves at low and high scan rates of the MSCs with different device dimensions. Notably, the areal capacitances of different device dimensions were close at high or low scan rates. An areal capacitance of 38 mF cm^{-2} was achieved at 80 mV s^{-1} when the device dimension was 50 × 50 μm^2, which was slightly higher than that when device dimensions were 15 × 15 and 100 × 100 μm^2 (37 and 35, respectively). When the scan rate was increased to 800 mV s^{-1}, the areal capacitance for all the dimensions was almost the same (18 mF cm^{-2}).

Figure R2.20 illustrates the effect of device dimensions on electrochemical performance. When the scan rate was 10 mV s^{-1}, the MSC with dimensions of 50 × 50, 15 × 15, and 100 × 100 μm^2 exhibited an areal capacitance of 68, 64, and 60 mF cm^{-2}, respectively. Notably, when the scan rate increased, the differences in electrochemical performance due to dimensions of MSCs disappeared.

The results revealed that the dimension of MSCs affected electrochemical performance at low scan rates. However, at high scan rates, dimensions did not affect electrochemical performance. However, the MSC of dimensions 50 × 50 μm^2 exhibited excellent electrochemical performance uniformity irrespective at high or low scan rates.

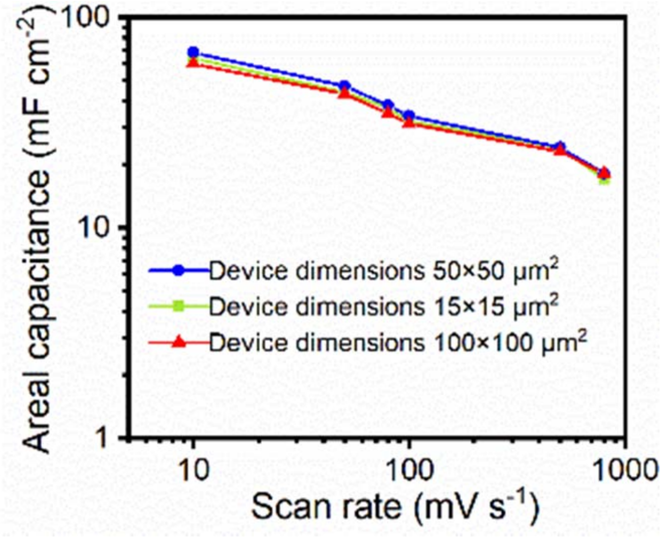

Figure R2.20 Areal capacitances of MSCs with different device dimensions as functions of the scan rate.

(3) In addition to the aforementioned two parameters, we studied the effect of the thickness of MSCs on electrochemical performance. We prepared graphene composite films of different thicknesses to process MSCs. The SEM image of the crosssection view of GO hybrid films of different thicknesses is provided in Figure R2.21.

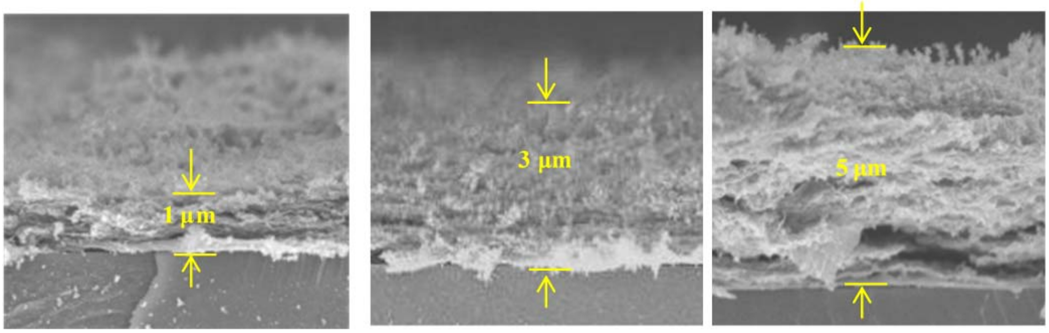

Figure R2.21 SEM image of cross section view of the hybrid film with different thicknesses

For a micro energy storage device, the optimum use of the limited room is critical to boost the areal capacitance and energy density. The 3D expandability of MSCs based on planar configuration is crucial. The increase in the thickness improves the areal capacitance and energy density, which is consistent with our goals. We also have to consider the volumetric capacitance and energy density.

Figure R2.22 (a) and (b) displays the CV characterizations of MSCs with different thicknesses at 80 and 800 mV s^{-1}. As expected, the thicker the MSC is, the

larger the areal capacitance is. The areal capacitances of the MSCs with thicknesses of 1, 3, and 5 μm were 13, 38, and 64 mF cm^{-2} at 80 mV s^{-1}. At high scan rates, the difference in the areal capacitance caused by thickness gradually decreases. The MSC with a thickness of 5 μm has the largest areal capacitance.

Figure R2.22 (c) and (d) depicts the areal and volumetric capacitance of MSCs with different thicknesses as functions of the scan rate. The higher the thickness of the MSC is, the higher the capacitance performance is. The influence of thickness on volumetric capacitance is more intuitive. Therefore, the volumetric capacitance can better explain the true influence of thickness on performance than capacitance. We determined that the volumetric capacitance of MSCs of different thicknesses were very close. At a low scan rate, the volumetric capacitance of MSCs of 3 μm is slightly higher than those of the other two. At a high scan rate, the thickness of the 1-μm supercapacitor exhibits superior performance.

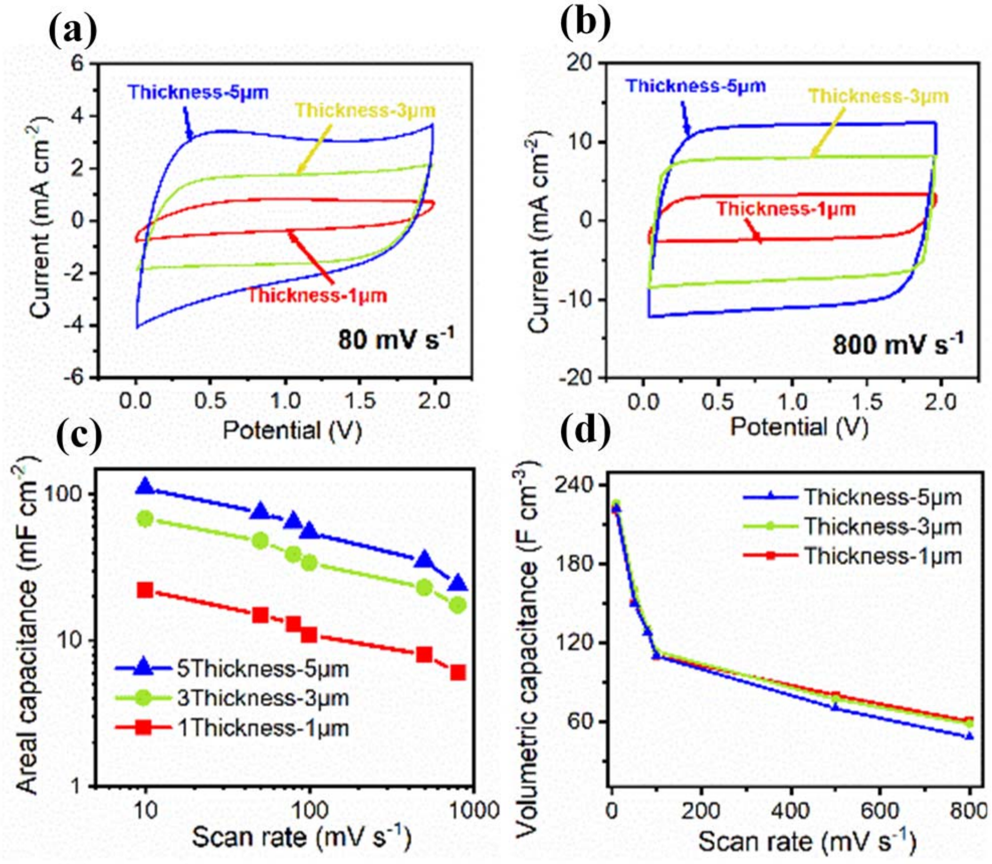

Figure R2.22 CV characterizations at low (a) and high (b) scan rates of the MSCs with different thicknesses. (c) and (d) Areal and volumetric capacitance of MSCs with different thicknesses as the functions of the scan rate

Modifications

According to the comments of the reviewer, we have added the impact of parameters on electrochemical performance.

The added part is inserted following Page 16, Paragraph 3, Line 2 is as following:

"On the basis of our findings, we selected the optimal parameter configuration of the interdigital MSCs for further electrochemical tests. We performed a series of parameter research and optimizations for the interdigital MSCs. We investigated the interdigital gap width, device dimensions, and thickness of the MSCs (Supplementary 21-23) to explore the effects of these factors on electrochemical performance."

The corresponding content added in Supporting Information is as following:

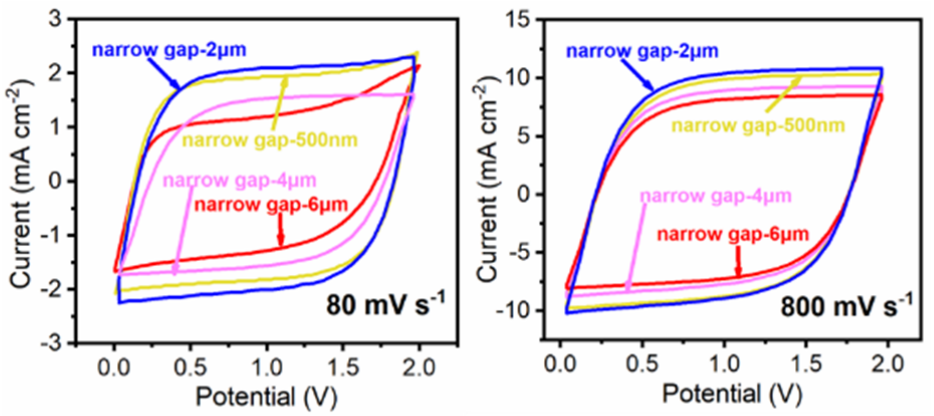

Supplementary Figure 21-1: Comparison of the electrochemical performance (CV characterizations at 80 and 800 mV/s) of MSCs with different narrow gaps. CV curves were recorded at variable scan rates to evaluate the effect of different microscale interdigital narrow gaps in the MSCs. As shown, the CVs of the high-resolution MSCs maintained a rectangular shape at different scan rates. When the interdigital narrow gaps were 500 nm, 2 μm, 4 μm, and 6 μm, different electrochemical performances are obtained. Of these, the MSCs with a narrow gap of 2 μm exhibited the optimal electrochemical performance, slightly higher than that

with a gap of 500 nm and higher than those with gaps of 4 and 6 μm.

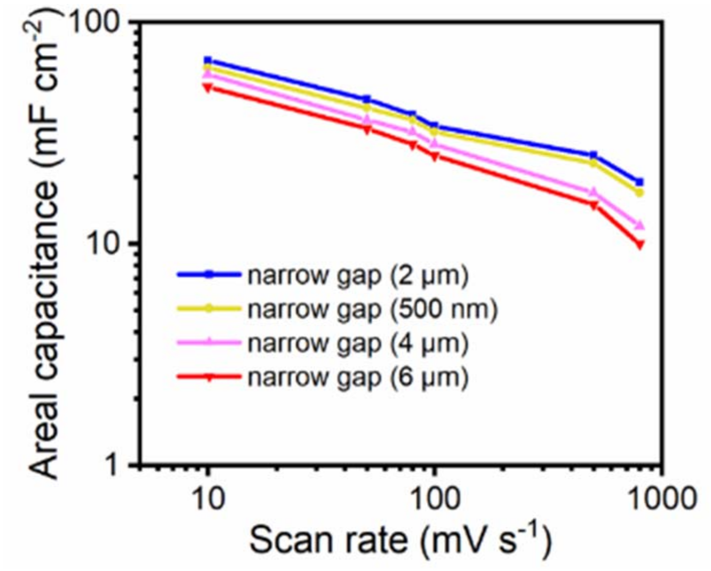

Supplementary Figure 21-2: Areal capacitance of MSCs with different narrow gaps as a function of scan rates. Our measurements and calculations showed that MSCs with different narrow gaps exhibited differences from areal capacitance under a lower scan rate. The highest areal capacitance of 67 mF/cm^2 was obtained when the narrow gap was 2 μm. Areal capacitances of 62, 58, and 51 mF/cm^2 were obtained for narrow gaps of 500 nm, 4 μm, and 6 μm, respectively.

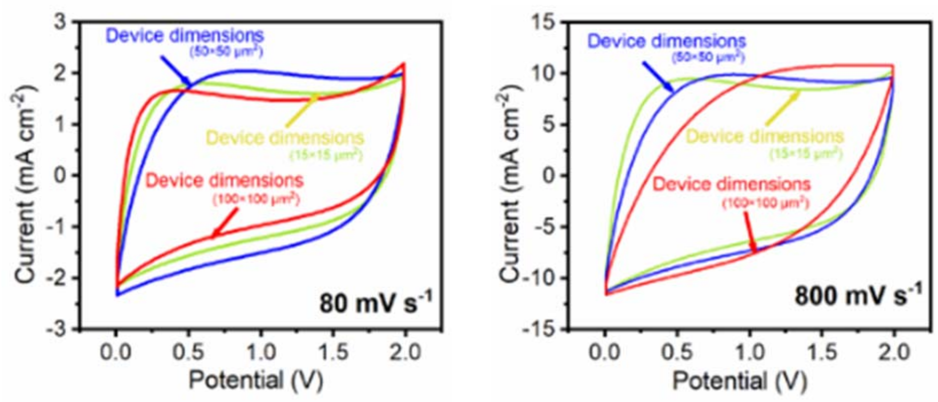

Supplementary Figure 22-1：Comparison of electrochemical performance (CV characterizations at 80 and 800 mV/s) of MSCs with different device dimensions. To understand and analyze the effect of device dimensions on electrochemical performance, we tested three sizes (15 × 15 μm^2, 50 × 50 μm^2, 100 × 100 μm^2) of MSCs. To increase the accuracy of the measurements, we simultaneously measured

the size of a dozen micron miniature supercapacitor requirements. We customized a probe with a contact diameter of 3 μm for use with a 100× confocal microscopy system. CV curves are shown at low and high scan rates for MSCs with different device dimensions. Contrary to expectations, the areal capacitances of different device dimensions were extremely similar at high or low scan rates. An areal capacitance of 38 mF/cm^2 was achieved at 80 mV/s when the device dimension was 50 × 50 μm^2, which was slightly higher than the areal capacitances of 37 and 35 mF/cm^2 achieved when the device dimensions were 15 × 15 μm^2 and 100 × 100 μm^2, respectively. When the scan rate was increased to 800 mV/s, the areal capacitance was almost the same (18 mF/cm^2).

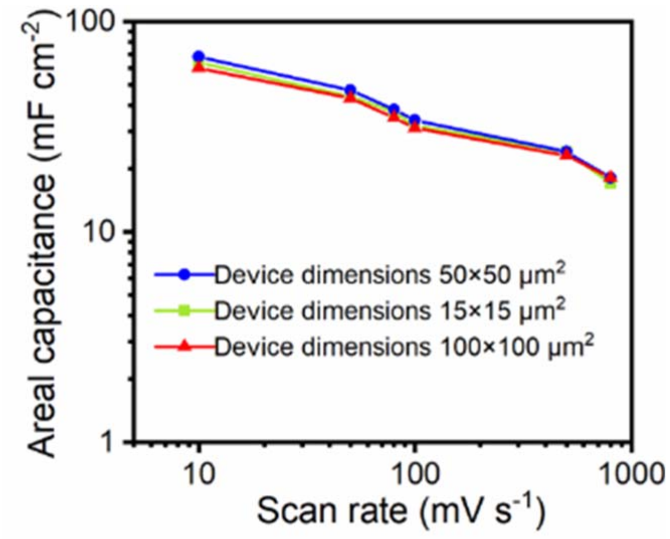

Supplementary Figure 22-2: Areal capacitance of MSCs with different device dimensions as functions of the scan rate. This figure shows the effect of device dimensions on electrochemical performance. When the scan rate is low, the dimension has little influence on the performance of the MSC. Thus, when the scan rate is 10 mV/s, the MSC with dimensions of 50×50 μm^2 has an areal capacitance of 68 mF/cm^2.Those with dimensions of 15 × 15 μm^2 and 100 × 100 μm^2 have areal capacitances of 64 mF/cm^2 and 60 mF/cm^2, respectively. Notably, as the scan rate increases, these differences in electrochemical performance disappear. The results showed that the dimensions of the MSC had an effect on the electrochemical performance at low scan rates, but this was almost negligible at high scan rates.

However, the MSC with dimensions of 50 × 50 μm^2 exhibited uniformly good electrochemical performance at both high and low scan rates.

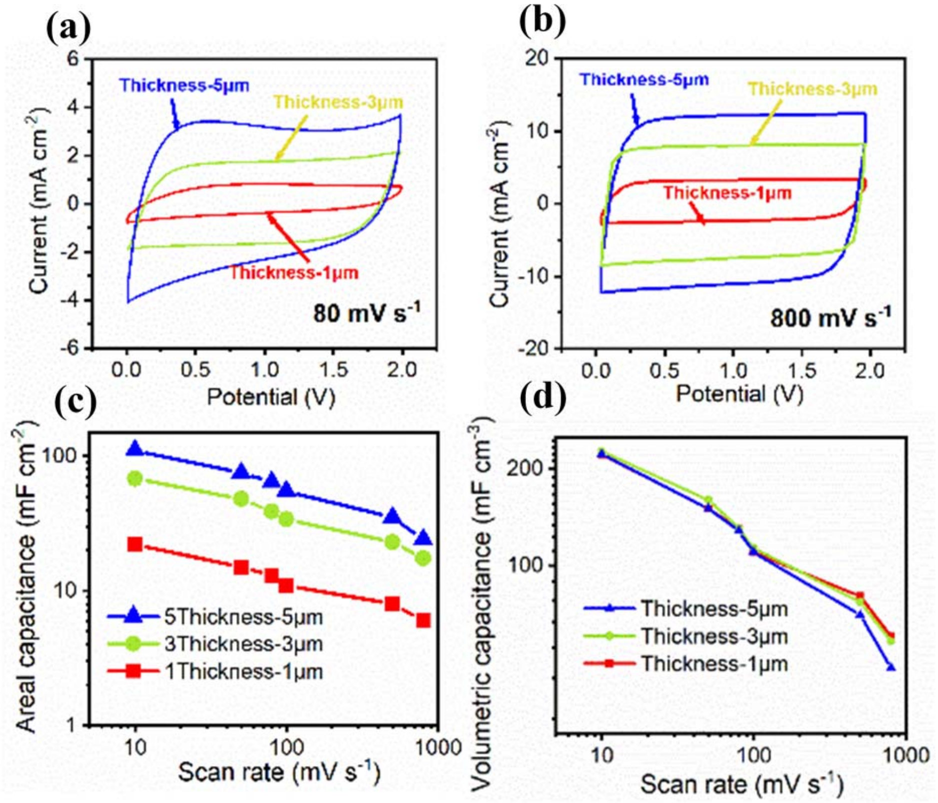

Supplementary Figure 23: Comparison of CV characterizations at (a) low and (b) high scan rates of MSCs with different thicknesses, as well as (c) areal and (d) volumetric capacitances of MSCs with different thicknesses as functions of the scan rate. Figure R14 (a) and (b) depicts the CV characterizations of MSCs with different thicknesses at 80 mV/s and 800 mV/s. As expected, the thicker the MSC, the larger the areal capacitance. The areal capacitances of MSCs with thicknesses of 1, 3, and 5 μm were 13, 38, and 64 mF/cm^2, respectively, at 80 mV/s. As the scan rate increases, the difference in areal capacitance caused by thickness gradually decreases. The MSC with a thickness of 5 μm exhibited the highest areal capacitance. Figure 14 (c) and (d) depicts the areal and volumetric capacitances of MSCs with different thicknesses as functions of the scan rate. With respect to areal capacitance, the higher the thickness of the MSC, the higher the capacitance. However, for volumetric

capacitance, the influence of thickness is more intuitive. Therefore, volumetric capacitance can better explain the true influence of thickness on performance. The volumetric capacitances of MSCs of different thicknesses are extremely similar. Specifically, at a low scan rate, the volumetric capacitance of a MSC with a thickness of 3 μm is slightly higher than that of the other MSCs. At a high scan rate, the supercapacitor with a thickness of 1 μm has the advantage.

2.7 Reviewer's Comments

2.7. "Either the "2 nm" scale bar in Figure 3 n and o or the indicated lattice spacings (0.29 nm and 0.33 nm) in these two TEM images are incorrect."

Our Response

We sincerely thank the reviewer for their patient review and valuable suggestions. We are very sorry for our oversight. We submitted the original TEM image to the system and used professional measurement software to resize the lattice spacing. Furthermore, we also evaluated other TEM and SEM graphs in the paper. Thank you for your meticulous reading and kind reminder.

We agree that the scale bars in Figure 3 were incorrect in the original manuscript. We used the aforementioned program to measure the correct lattice spacing, but erred in reading the scale bars. Therefore, we replaced the TEM image in the source data, remeasured the lattice distance and relabeled the scale.

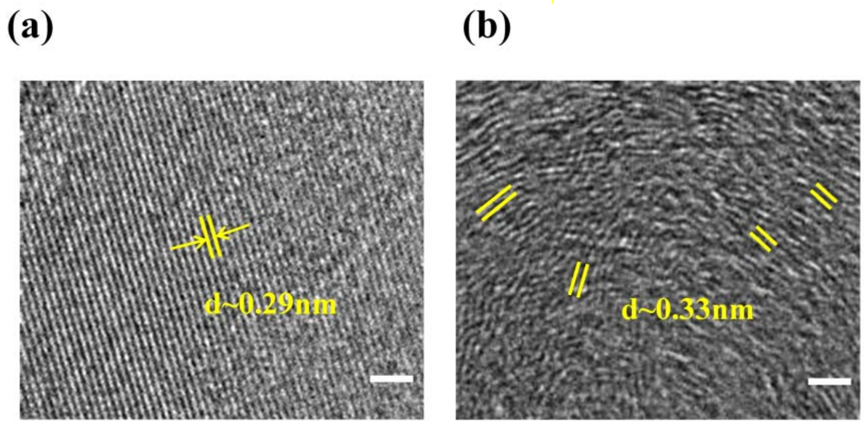

Figure 2.23 (a) and (b) HRTEM image of the indicated lattice space (scale bar, 2 nm).

Figure 2.23 (a) exhibits the HRTEM image of MnO_2 nanoparticles. The distance between the lattice fringes was approximately 0.29 nm, which was consistent with that of the {001} lattice planes of the α-MnO_2 crystal. The ripple-like wrinkled structures in graphene improves the electrochemical performance of devices, and the lattice space of 0.33 nm in Figure 2.23 (b) corresponds to the distance between two neighboring (002) planes in graphitic materials.

Modifications

We have revised the corresponding part in our manuscript as per the reviewer's suggestions

Figure 3 in manuscript was replaced.

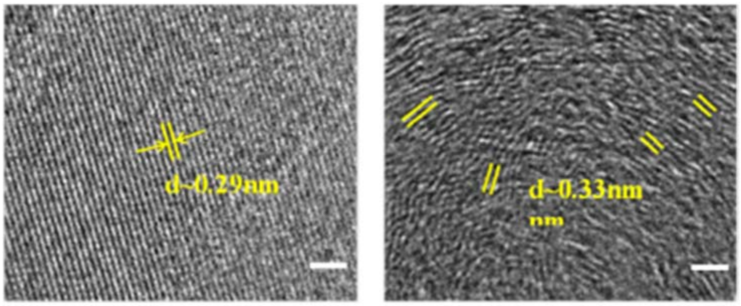

2.8 Reviewer's Comments

2.8. "The English in this manuscript, especially the materials characterization discussion and electrochemical performance evaluation could be further improved by a professional English polishing service team. There are still some typos that need further revision before submission to another journal, such as "surfacearea" on page 2."

Our Response

We deeply admire the reviewer's professional perspective on the paper. Your

suggestion helped us to considerably improve the quality of the article and avoid mistakes.

All of our coauthors rewrote the manuscript to improve its fluency and readability. We then sent the article to a professional English editing service team to improve the language and grammar of the article. We have emphasized material characterization and electrochemical performance evaluation.

Thank you again for your patient review and valuable comments.

[1] El-Kady, M. F., & Kaner, R. B. Scalable fabrication of high-power graphene MSCs for flexible and on-chip energy storage. Nature communications, 4, 1475 (2013).
[2] Zhang, C., Kremer, M. P., Seral‐Ascaso, A., Park, S. H., McEvoy, N., Anasori, B., ... & Nicolosi, V. Stamping of flexible, coplanar micro‐supercapacitors using MXene inks. Advanced Functional Materials, 28(9), 1705506 (2018).
[3] Liu, Y., Weng, B., Xu, Q., Hou, Y., Zhao, C., Beirne, S., ... & Chen, J. Facile fabrication of flexible MSC with high energy density. Advanced Materials Technologies, 1(9), 1600166 (2016).
[4] Liu, Z., Wu, Z. S., Yang, S., Dong, R., Feng, X., & Müllen, K. Ultraflexible in‐plane micro‐supercapacitors by direct printing of solution‐processable electrochemically exfoliated graphene. Advanced Materials, 28(11), 2217-2222 (2016).
[5] Wang, W., Liu, W., Zeng, Y., Han, Y., Yu, M., Lu, X., & Tong, Y. A novel exfoliation strategy to significantly boost the energy storage capability of commercial carbon cloth. Advanced materials, 27(23), 3572-3578 (2015).
[6] Jeon, H., Jeong, J. M., Hong, S. B., Yang, M., Park, J., Hwang, S. Y., & Choi, B. G. Facile and fast microwave-assisted fabrication of activated and porous carbon cloth composites with graphene and MnO2 for flexible asymmetric supercapacitors. Electrochimica Acta, 280, 9-16 (2018).
[7] Gao, W., Singh, N., Song, L., Liu, Z., Reddy, A. L. M., Ci, L., ... & Ajayan, P. M. Direct laser writing of MSCs on hydrated graphite oxide films. Nature nanotechnology, 6(8), 496-500 (2011).
[8] Gerchberg, R. W. A practical algorithm for the determination of phase from image and diffraction plane pictures. Optik, 35, 237-246 (1972).
[9] Di Leonardo, R., Ianni, F., & Ruocco, G. Computer generation of optimal holograms for optical trap arrays. Optics Express, 15(4), 1913-1922 (2007).
[10] Dinh, T. M., Armstrong, K., Guay, D., & Pech, D. High-resolution on-chip supercapacitors with ultra-high scan rate ability. Journal of Materials Chemistry A, 2(20), 7170-7174 (2014).
[11] Ferris, A., Reig, B., Eddarir, A., Pierson, J. F., Garbarino, S., Guay, D., & Pech, D. Atypical properties of FIB-patterned RuOx nanosupercapacitors. ACS Energy Letters, 2(8), 1734-1739 (2017).
[12] del Pino, A. P., György, E., Alshaikh, I., Pantoja-Suárez, F., Andújar, J. L., Pascual, E., ... & Bertran-Serra, E. Laser-driven coating of vertically aligned carbon nanotubes with manganese oxide from metal organic precursors for energy storage. Nanotechnology, 28(39), 395405 (2017).
[13] Baeuerle, D. Laser Processing and Chemistry Springer Verlag. Heidelberg Berlin, 13 (2000).
[14] Maeng, J., Heo, S., Jo, G., Choe, M., Kim, S., Hwang, H., & Lee, T. The effect of excimer laser annealing on ZnO nanowires and their field effect transistors. Nanotechnology, 20(9), 095203 (2009).
[15] del Pino, A. P., György, E., Marcus, I. C., Roqueta, J., & Alonso, M. I. Effects of pulsed laser radiation on epitaxial self-assembled Ge quantum dots grown on Si substrates. Nanotechnology, 22(29), 295304 (2011).
[16] Aminuzzaman, M., Watanabe, A., & Miyashita, T. Direct writing of conductive silver micropatterns on flexible polyimide film by laser-induced pyrolysis of silver

nanoparticle-dispersed film. Journal of Nanoparticle Research, 12(3), 931-938 (2010).

[17] Queraltó, A., Perez del Pino, A., de la Mata, M., Arbiol, J., Obradors, X., & Puig, T. Ultrafast crystallization of Ce0. 9Zr0. 1O2–y epitaxial films on flexible technical substrates by pulsed laser irradiation of chemical solution derived precursor layers. Crystal Growth & Design, 15(4), 1957-1967 (2015).

[18] Ghaith, E. S., Hayakawa, T., Kasuga, T., & Nogami, M. Apatite formation on rutile type TiO 2 films formed by laser irradiation. Journal of materials science, 41(8), 2521-2524 (2006).

[19] Baldus, O., & Waser, R. Laser crystallization studies of barium strontium titanate thin films. Journal of the European Ceramic Society, 24(10-11), 3013-3020 (2004).

[20] Bharadwaja, S. S. N., Dechakupt, T., Trolier‐McKinstry, S., & Beratan, H. Excimer laser crystallized (Pb, La)(Zr, Ti) O3 thin films. Journal of the American Ceramic Society, 91(5), 1580-1585 (2008).

[21] Queraltó, A., Del Pino, A. P., De La Mata, M., Tristany, M., Obradors, X., Puig, T., & Trolier-McKinstry, S. Ultraviolet pulsed laser crystallization of Ba0. 8Sr0. 2TiO3 films on LaNiO3-coated silicon substrates. Ceramics International, 42(3), 4039-4047 (2016).

[22] Tao, X. Y., Fsaifes, I., Koncar, V., Dufour, C., Lepers, C., Hay, L., ... & Bouazaoui, M. CO2 laser-induced crystallization of sol–gel-derived indium tin oxide films. Applied physics A, 96(3), 741-749 (2009).

[23] Xu, M., Kong, L., Zhou, W., & Li, H. Hydrothermal synthesis and pseudocapacitance properties of α-MnO2 hollow spheres and hollow urchins. The Journal of Physical Chemistry C, 111(51), 19141-19147 (2007).

[24] Devaraj, S., & Munichandraiah, N. Effect of crystallographic structure of MnO2 on its electrochemical capacitance properties. The Journal of Physical Chemistry C, 112(11), 4406-4417 (2008).

[25] Toupin, M., Brousse, T., & Bélanger, D. Influence of microstucture on the charge storage properties of chemically synthesized manganese dioxide. Chemistry of Materials, 14(9), 3946-3952 (2002).

[26] Kim, F., Luo, J., Cruz‐Silva, R., Cote, L. J., Sohn, K., & Huang, J. Self‐propagating domino‐like reactions in oxidized graphite. Advanced Functional Materials, 20(17), 2867-2873 (2010).

[27] Hwang, J. Y., El-Kady, M. F., Li, M., Lin, C. W., Kowal, M., Han, X., & Kaner, R. B. Boosting the capacitance and voltage of aqueous supercapacitors via redox charge contribution from both electrode and electrolyte. Nano Today, 15, 15-25 (2017).

[28] Hwang, J. Y., El-Kady, M. F., Wang, Y., Wang, L., Shao, Y., Marsh, K., ... & Kaner, R. B. Direct preparation and processing of graphene/RuO2 nanocomposite electrodes for high-performance capacitive energy storage. Nano Energy, 18, 57-70 (2015).

[29] Larciprete, R., Fabris, S., Sun, T., Lacovig, P., Baraldi, A., & Lizzit, S. Dual path mechanism in the thermal reduction of graphene oxide. Journal of the American Chemical Society, 133(43), 17315-17321 (2011).

[30] Zhou, S., & Bongiorno, A. Origin of the chemical and kinetic stability of graphene oxide. Scientific reports, 3, 2484 (2013).

[31] Yin, Z., Wu, S., Zhou, X., Huang, X., Zhang, Q., Boey, F., & Zhang, H. Electrochemical deposition of ZnO nanorods on transparent reduced graphene oxide electrodes for hybrid solar cells. Small, 6(2), 307-312 (2010).

[32] Wu, J., Agrawal, M., Becerril, H. A., Bao, Z., Liu, Z., Chen, Y., & Peumans, P. Organic light-emitting diodes on solution-processed graphene transparent electrodes. ACS nano, 4(1), 43-48 (2010).

[33] Li, B., Cao, X., Ong, H. G., Cheah, J. W., Zhou, X., Yin, Z., ... & Zhang, H. All‐Carbon Electronic Devices Fabricated by Directly Grown Single‐Walled Carbon Nanotubes on Reduced Graphene Oxide Electrodes. Advanced Materials, 22(28), 3058-3061 (2010).

[34] HeQ, S. H. G. Centimeter-long and large-scale micropatterns of reduced grapheme oxide films: Fabrication and sensing applications. ACS Nano, 4(6), 3201 (2010).

[35] Lobo, D. E., Banerjee, P. C., Easton, C. D., & Majumder, M. Miniaturized supercapacitors: focused ion beam reduced graphene oxide supercapacitors with enhanced performance metrics. Advanced Energy Materials, 5(19), 1500665 (2015).

[36] Miller, J. R., Outlaw, R. A., & Holloway, B. C. Graphene double-layer capacitor with ac line-filtering performance. Science, 329(5999), 1637-1639 (2010).

[37] Zhao, G., Zhang, N., & Sun, K. Porous MoO3 films with ultra-short relaxation time used for supercapacitors. Materials research bulletin, 48(3), 1328-1332 (2013).

[38] Liu, W., Lu, C., Wang, X., Tay, R. Y., & Tay, B. K. High-performance MSCs based on two-dimensional graphene/manganese dioxide/silver nanowire ternary hybrid film. ACS nano, 9(2), 1528-1542 (2015).

[39] Du, X., Guo, P., Song, H., & Chen, X. Graphene nanosheets as electrode material for electric double-layer capacitors. Electrochimica Acta, 55(16), 4812-4819 (2010).

[40] Wu, Z. S., Liu, Z., Parvez, K., Feng, X., & Müllen, K. Ultrathin printable graphene supercapacitors with AC line‐filtering performance. Advanced Materials, 27(24), 3669-3675 (2015).

[41] Zhang, M., Zhou, Q., Chen, J., Yu, X., Huang, L., Li, Y., ... & Shi, G. An ultrahigh-rate electrochemical capacitor based on solution-processed highly conductive PEDOT: PSS films for AC line-filtering. Energy & Environmental Science, 9(6), 2005-2010 (2016).

[42] Pech, D., Brunet, M., Durou, H., Huang, P., Mochalin, V., Gogotsi, Y., ... & Simon, P. Ultrahigh-power micrometre-sized supercapacitors based on onion-like carbon. Nature nanotechnology, 5(9), 651-654 (2010).

[43] Pech, D., Brunet, M., Dinh, T. M., Armstrong, K., Gaudet, J., & Guay, D. Influence of the configuration in planar interdigitated electrochemical micro-capacitors. Journal of power sources, 230, 230-235 (2013).

[44] Olthuis, W., Streekstra, W., & Bergveld, P. Theoretical and experimental determination of cell constants of planar-interdigitated electrolyte conductivity sensors. Sensors and Actuators B: Chemical, 24(1-3), 252-256 (1995).

[45] Liu, W., Lu, C., Li, H., Tay, R. Y., Sun, L., Wang, X., ... & Yan, J. Paper-based all-solid-state flexible MSCs with ultra-high rate and rapid frequency response capabilities. Journal of Materials Chemistry A, 4(10), 3754-3764 (2016).

[46] Brezesinski, T., Wang, J., Tolbert, S. H., & Dunn, B. Ordered mesoporous α-MoO 3 with iso-oriented nanocrystalline walls for thin-film pseudocapacitors. Nature materials, 9(2), 146-151 (2010).

[47] Tarascon, J. M., & Armand, M. Determination of the chemical diffusion coefficient of lithium in LiFePO4. Nature, 414, 359-367 (2001).

[48] Whittingham, M. S. Lithium batteries and cathode materials. Chemical reviews, 104(10),

4271-4302 (2004).
[49] Zhang, X., Zhang, H., Li, C., Wang, K., Sun, X., & Ma, Y. Recent advances in porous graphene materials for supercapacitor applications. Rsc Advances, 4(86), 45862-45884 (2014).
[50] Sun, H., Zhu, J., Baumann, D., Peng, L., Xu, Y., Shakir, I., ... & Duan, X. Hierarchical 3D electrodes for electrochemical energy storage. Nature Reviews Materials, 4(1), 45-60 (2019).

3. REVIEWER NO.3

3.0 Reviewer's Comments

This paper deals with laser-stamping mcrosupercapacitor technology and its manufacturing. The authors have used the femtosecond laser source for patterning the electrodes. Although this a new finding in the MSC field by femtosecond laser, however, several issues are still there as a matured technology. A few comments are given as appended below.

Our Response

We truly appreciate the reviewer for the efforts for reviewing our paper. We are happy to have received the following excellent suggestions for improving the paper quality. We have modified the corresponding text following the reviewer's comments.

3.1 Reviewer's Comments

3.1. "I have a crucial comment on the title of this paper. What does ultrafast signify? Is it a very fast method or the authors used ultrafast laser sources? Clarify."

Our Response

We are very grateful for the reviewer's detailed comments, which were crucial for improving our paper.

We apologize for the misunderstanding caused by the unclear expression. We think the comment on the title is meaningful; hence, the title has been revised. We want to emphasize the very fast processing method (the femtosecond laser we used is indeed an ultrafast laser) in the title. In the title of the original manuscript, "Ultrafast laser-stamping for MSC manufacturing," the term "ultrafast" is really ambiguous. In combination with the comment that "Manufacturing technology is not the proper phrase for this manuscript," all coauthors have made rigorous and accurate changes to the title.

Modifications

According to the reviewer's comments, we have revised the corresponding part in our manuscript.

The original title is as follows:

"Ultrafast laser-stamping for MSCs manufacturing"

The title is revised as following:

"Laser photonic-reduction stamping for graphene-based MSCs ultrafast fabrication"

3.2 Reviewer's Comments

3.2. "If it is the very fast production of the device, then one has to justify with literature and explain with the statement '1 cm2 within 10 minutes', which is not a fast procedure."

Our Response

We thank the reviewer for the detailed and thorough attention to the paper and the advice for improving the paper quality. However, our description in the article was unclear and could cause misunderstanding. In the revised manuscript, we have mentioned that the processing time of 10 min was only to form a specific concept of our technology, ">30,000 laser-stamping MSCs of 1 cm^2 were produced within 10 min," to reflect the capacity of ultrafast processing and micro integration.

The recent technological trend of using electronic devices has increased the requirement of micro power sources and small-scale energy storage devices[R1-3]. Therefore, we must minimize the size of single supercapacitors to integrate multiple supercapacitors into the smallest possible area. The size of an average miniature supercapacitor is mostly in the centimeter range, and the minimum distance between adjacent electrodes in an interdigitated configuration ranges from 500 to 5 μm[R4-18]. In our study, we realized the ultrafast fabrication of supercapacitors with a minimum size (15×15 μm^2) and high resolution (500 nm). We integrated >30,000 MSCs of this size into a considerably small area (1 cm^2).

To demonstrate our technology more intuitively, Figures R3.1 and 2 illustrate the difference between the laser direct writing and our shaped-laser processing of MSCs.

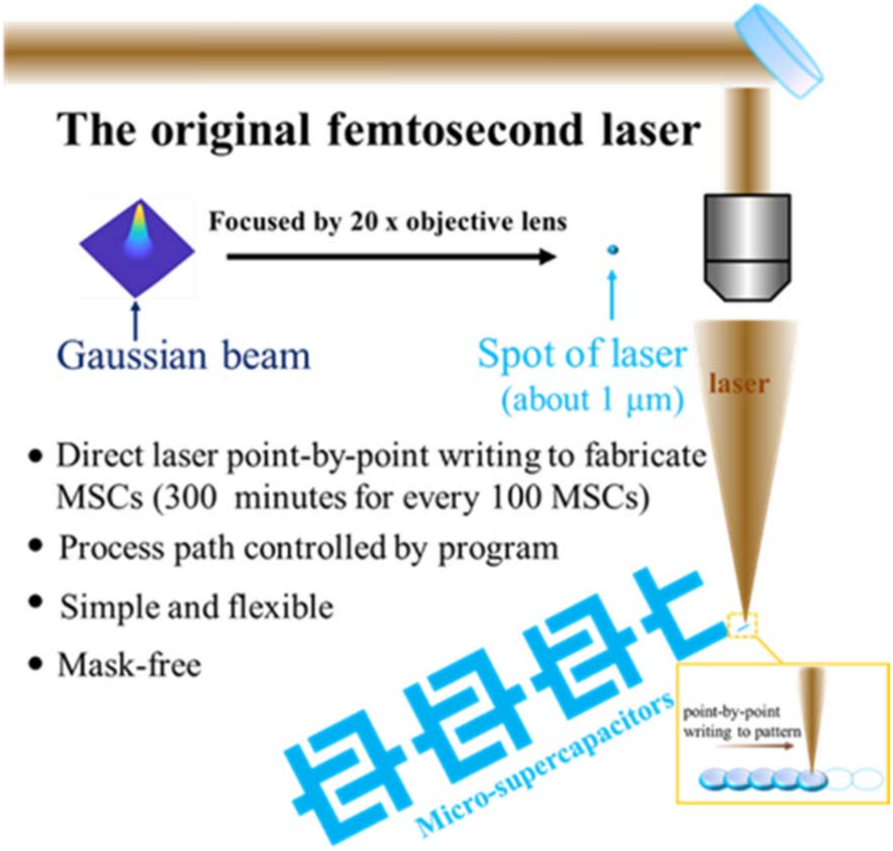

Figure R3.1 Schematic of the fabrication of micro-supercapcacitors using direct laser writing

Traditional laser direct writing improves the efficiency of the fabrication of MSCs and other micro devices and presents the advantages of a mask-free process, flexibility, and high resolution (Figure R3.1). The laser point-by-point writing of the focal spot is realized by controlling the movement of the translation stage. Therefore, in actual processing, we must consider the stability of the translation stage and repeated positioning accuracy to ensure processing consistency, and achieving multipatterned rapid processing is difficult.

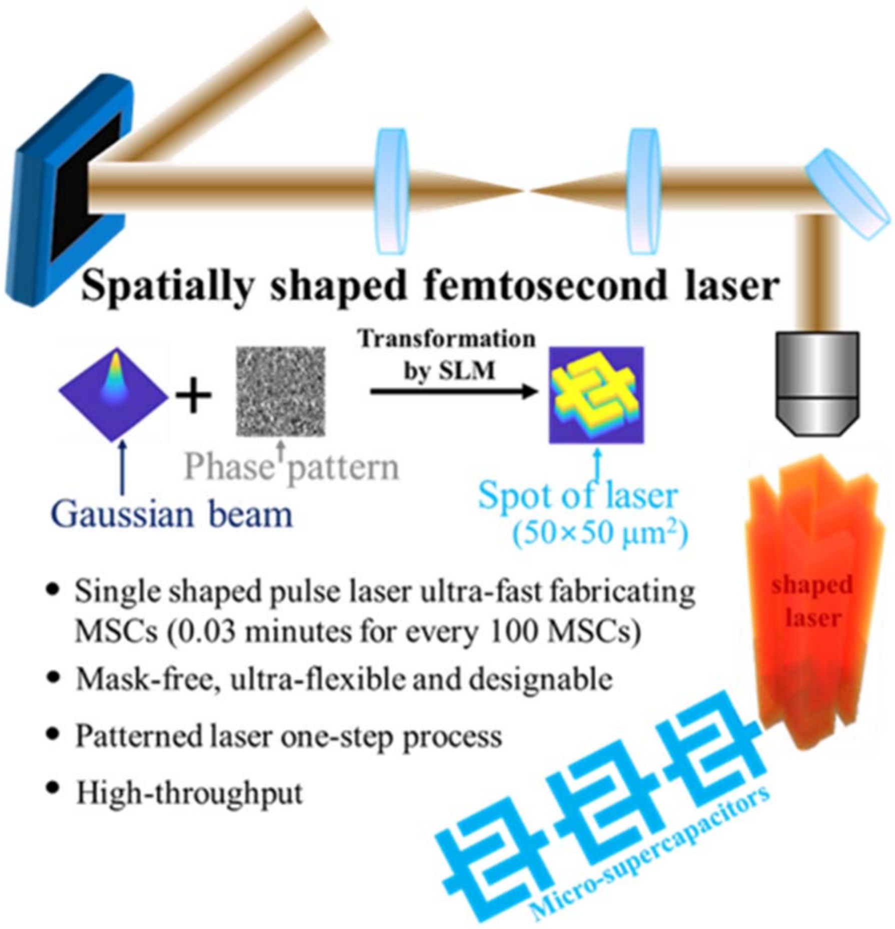

Figure R3.2 Schematic of the fabrication of micro-supercapcacitors using shaped laser

Figure R3.2 shows that the shaped femtosecond laser can be formed in one step, and the shape can be changed by changing the phase pattern. The spot of the shaped laser is a designable pattern spot that can directly and instantaneously achieve the fabrication of patterned MSCs. In addition to the advantages of laser direct writing, this technology considerably improves processing efficiency and can enable the consistency of large-area processing. In theory, this process is completed in an instant.

Thus, "considerably rapid device production" implies that we can fabricate tens of thousands of MSCs within 10 min. We used the Ti:sapphire laser regenerative amplifier system, and it can generate 1,000 single pulses per second. In theory, each single pulse can be achieved through MSC processing; consequently, a single MSC can be fabricated in only 0.001 s. During actual processing, we processed >50 MSCs per second. As the below table shows, our technology is ten or hundred times more

efficient than the reported processes of MSCs[R18-23].

**Table R3.1: Comparison of various methods in fabrication efficiency, size and narrow gap of fabricated MSCs**		
**Methods**	**Size of supercapacitors (narrow gap)**	**Fabrication efficiency /30min**
Light scribe DVD[18]	0.5×1 cm^2 (150 μm)	100
3D-Printed stamping[19]	1×2 cm^2 (400 μm)	24
Coating and laser etching[20]	1×1 cm^2 (500 μm)	180
Inkjet-printing[21]	1×2 cm^2 (1000 μm)	100
Electrochemical activation[22]	1×1 cm^2	10
Microwave radiation[23]	1×2 cm^2	180
SFLS strategy (Our work)	50×50 μm^2 (0.5 μm)	90,000

We apologize for the misguiding statement of "1 cm^2 within 10 minutes." In fact, completing processing of >30,000 supercapacitors in 10 min is considerably rare, and for a size of a dozen microns of supercapacitors, 1 cm^2 is a relatively large area. In this case, we are not emphasizing the relationship between 10 min and 1 cm^2. The statement emphasizes that numerous MSCs can be prepared in a considerably short time and small area, which is highly crucial for the practical applications of energy storage in microdevices. To show our super-high processing efficiency to readers in a more specific and digital way, we used "10 min" as a measure of time required to process >30,000 supercapacitors. Therefore, "10 min" is just an expression used for a specific description, and "1 cm^2" is used to represent the substantially small area required for accommodating numerous MSCs, thereby emphasizing the advantages of our miniaturized preparation.

This confusing expression has been revised in the article.

Modifications

According to the reviewer's comments, we have revised the corresponding part in our manuscript to better describe the ultrahigh efficiency of this technology without causing misunderstanding.

The original abstract form Page 1, Paragraph 1, Line 3 to Line 6 is as flowing:

"Here, a versatile spatially shaped femtosecond laser stamp strategy is proposed to ultrafastly manufacture the designable flexible laser-stamping MSCs from the graphene oxide based film. More than 30,000 laser-stamping MSCs are produced in 1 cm^2 within 10 minutes."

This part is revised as following:

"Here, a flexible, designable MSC can be fabricated by a single pulse laser photonic-reduction stamping. A thousand spatially shaped laser pulses can be generated in one second, and over 30,000 MSCs are produced within 10 minutes."

The original part form Page 6, Paragraph 1, Line 1to Line 9 is as flowing:

"The ss-fs laser can achieve ultra-fast fabrication of various-shapes L-S-MSCs (Fig. 1b). Notably, such high fabrication efficiency for flexible L-S-MSCs is rare, and ss-fs laser also demonstrates high machining accuracy and near faultless processing consistency, which enable rapid and large-scale applications. Under present conditions, we could process 3,000 L-S-MSCs in 1 minute. This rate was tens or hundreds of times more efficient than previously reported processes of MSCs[28-33]. As the Table shows, our SFLS strategy is compared with the methods that have been reported before in manufacturing efficiency, size and narrow gap. The technology we proposed has unprecedented manufacturing efficiency and could fabricate 90,000 L-S-MSCs in thirty minutes. Our processing video is included in the Supplementary Information."

This part is revised as following:

"The SSFL method achieved ultrafast fabrication of variously shaped MSCs (Fig. 1b) and revolutionizes the traditional processing method of direct laser writing (Supplementary Fig. 1). Traditional laser point-by-point writing of the focal spot is realized by controlling the movement of the translation stage, which considerably limits the method' s application in the ultrafast fabrication of MSCs. In our work, the

spot of each laser pulse can be a designable pattern spot shaped by a spatial light modulator that directly and instantaneously completes a patterned MSC. The SSFL strategy not only retains the advantages of being mask-free, flexibility and high-resolution, it also achieves the ultrafast fabrication of MSCs.

We used the Ti: sapphire laser regenerative amplifier system, which can generate 1,000 single pulses per second. In theory, a single MSC could be fabricated in only 1 millisecond. The actual processing speed observed was more than 3,000/min, which is tens or hundreds of times more efficient than previously reported processes for fabricating MSCs[31-36]. The SSFL also has the advantages of high machining accuracy and near faultless processing consistency, enabling rapid and large-scale application. As illustrated in the Table, our SSFL strategy was compared with the methods previously reported regarding fabrication efficiency, size, and narrow gap. The proposed technique has unprecedented fabrication efficiency and fabricated high-resolution and micron-scale supercapacitors (a processing video is included in the Supplementary Information)."

The corresponding content in Supporting Information is as following:

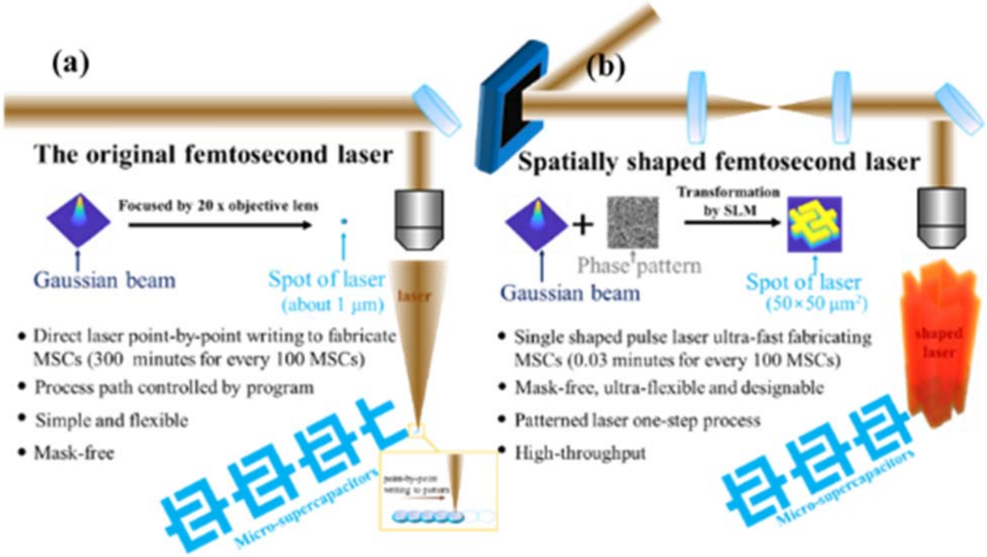

Supplementary Figure 1: Schematics of the fabrication of MSCs using (a) direct laser writing and (b) a spatially shaped laser. Traditional laser point-by-point writing of the focal spot is realized by controlling the movement of the translation

stage. Therefore, in actual processing, we consider the stability of the translation stage and repeated positioning accuracy to ensure the consistency of processing; multipatterned rapid processing is difficult to achieve. The incident beams (Gaussian beams) were transformed into arbitrary geometric target beams in SLM by programming phase patterns; the shaped beams were then transmitted on the hybrid GO films. The shaped femtosecond laser can be formed in one step and alters the shape by changing the phase pattern. The spot of the shaped laser is a designable pattern that can directly and instantaneously complete patterned MSCs. In conjunction with the advantages of laser direct writing, this technology greatly improves processing efficiency and achieves consistency for large-area processing. In theory, this process is instantaneous.

The added part is inserted following Page 8, Paragraph 1, Line 1 is as following:

"Notably, the proposed technique can fabricate numerous MSCs within an extremely short time and small area, which is particularly valuable for the practical application of energy storage in microdevices and can be widely extended to other material systems, or other graphene-based composites."

3.3 Reviewer's Comments

3.3. "Several optimization parameters are missing. e.g. spot size and speed of the laser or the film quality and how it is changing during that optimization."

Our Response

We thank the reviewer for their careful review of our paper. We are grateful to receive such excellent advice to improve our paper quality.

We studied the parameters of different shapes of microcapacitors and different laser fluences during processing. However, these parameters were not enough to further discuss the influence of many parameters on the chemical performance of MSCs. According to the reviewer's suggestions, we performed an experiment of parameters of the spot size and the narrow gap between the spot and film quality of MSCs.

(1) The spot size is a vital parameter in the preparation of our MSC. We

employed spatial light field shaping to transform the original Gaussian light beam into a three-dimension-shaped laser. This laser was patterned in one step in the form of a single pulse without direct laser writing. Laser direct writing requires a program-controlled continuous laser scan of focused spots to complete one pattern[R24-26], which makes MSC preparation a time-consuming procedure. In our method, the laser is directly changed into a pattern laser that can be used for processing, and the light spot focused on a single point is changed to a shaped light field with a controllable shape and size. We used the Ti:sapphire laser regenerative amplifier system that can generate 1,000 single pulses per second. Therefore, the laser speed for fabrication was fixed.

Each single pulse can achieve MSC processing; consequently, a single MSC can be fabricated in only 0.001 s. During actual processing, we processed >50 MSCs per second. The processing efficiency obtained using this method is considerably high. Reshaping the femtosecond laser can provide "ultrafast patterning of laser-induced graphene/MnO_2 electrodes." The laser spots of our shaped laser are shown in the following figure.

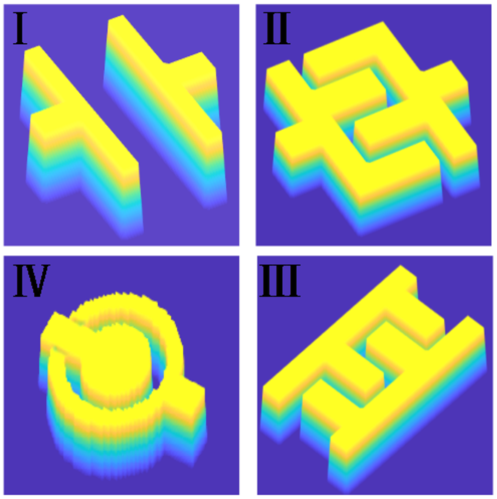

Figure R3.3 The optical field distribution diagram optimized by the algorithm

Furthermore, our technique can adjust the spot size to realize the processing of MSCs of different sizes, ranging from 15 × 15 to 100 × 100 μm^2 (Figure R3.4).

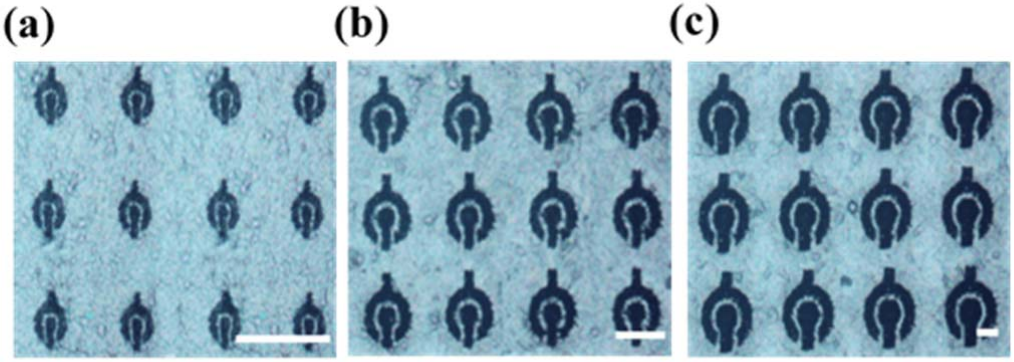

Figure R3.4 The micrographs of MSCs with varying sizes (scale bar, 50 μm)

To understand and analyze the effects of spot sizes on electrochemical performance, we selected three sizes (15 × 15, 50 × 50, and 100 × 100 μm^2) of MSCs in this study. To accurately determine the size and requirements of a dozen micron-sized miniature supercapacitors, we customized a probe with a contact diameter of 3 microns for use with a 100× confocal microscopy system.

We evaluated the electrochemical behavior of MSC at different scan rates. Figure R3.5 depicts the CV curves at low and high scan rates of the MSCs with different device dimensions. Notably, the areal capacitances of different device dimensions were close at high or low scan rates. An areal capacitance of 38 mF cm^{-2} was achieved at 80 mV s^{-1} when the device dimension was 50 × 50 μm^2, which was slightly higher than that when device dimensions were 15 × 15 and 100 × 100 μm^2 (37 and 35, respectively). When the scan rate was increased to 800 mV s^{-1}, the areal capacitance for all the dimensions was almost the same (18 mF cm^{-2}).

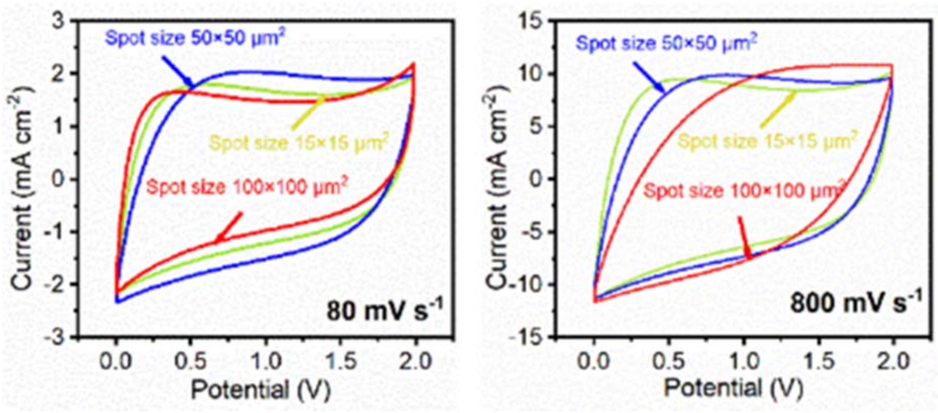

Figure R3.5 The comparison of electrochemical performances (CV characterizations at different scan rates) of the MSCs fabricated by different size of spots.

Figure R3.6 illustrates the effect of device dimensions on electrochemical performance. When the scan rate was 10 mV s^{-1}, the MSC with dimensions of 50 × 50, 15 × 15, and 100 × 100 μm^2 exhibited an areal capacitance of 68, 64, and 60 mF cm^{-2}, respectively. Notably, when the scan rate increased, the differences in electrochemical performance due to dimensions of MSCs disappeared.

The results revealed that the dimension of MSCs affected electrochemical performance at low scan rates. However, at high scan rates, dimensions did not affect electrochemical performance. However, the MSC of dimensions 50 × 50 μm^2 exhibited excellent electrochemical performance uniformity irrespective at high or low scan rates.

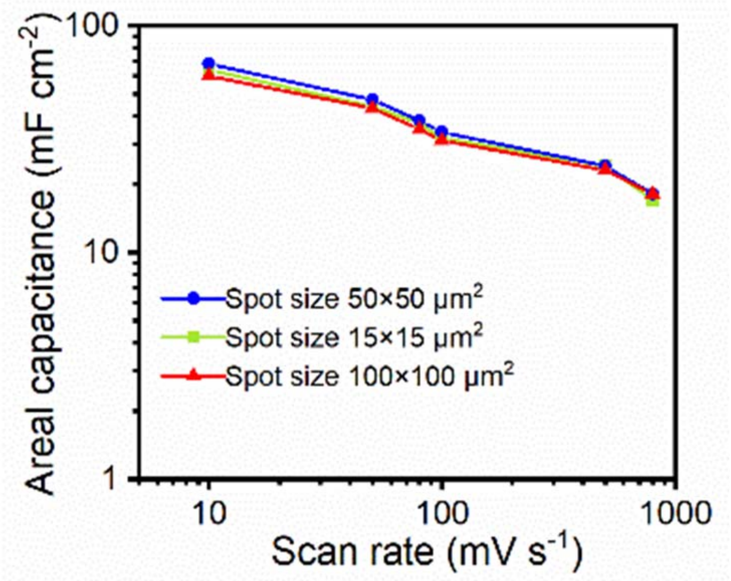

Figure R3.6 Areal capacitances of MSCs fabricated by different size of spots as functions of the scan rate.

(2) We explored the effect of the interdigital gap width on electrochemical performance when designing the MSCs with narrow gaps of 500 nm and 2, 4, and 6 μm (Figure R3.7).

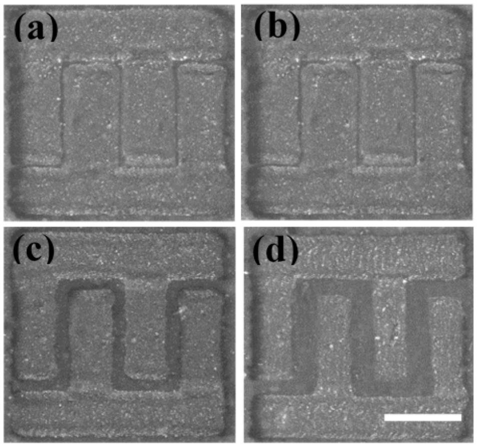

Figure R3.7 The SEM of interdigital MSCs with different narrow gaps (scale bar, 20 μm). The narrow gaps of the figures (a), (b), (c) and (d) are represented as 500 nm, 2μm, 4μm and 6μm respectively

As depicted in Figure R3.8, cyclic voltammograms (CVs) were recorded at variable scan rates to evaluate the effect of various microscale interdigital narrow gaps of MSCs. The CVs of the high-resolution MSCs maintained a rectangular shape at all scan rates. Notably, the MSCs with a narrow gap of 2 μm exhibited the best electrochemical performance.

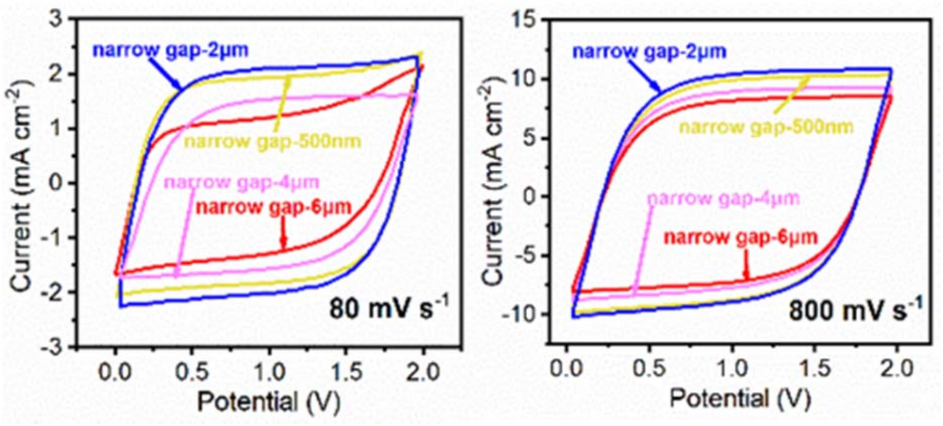

Figure R3.8 The comparison of electrochemical performances (CV characterizations at different scan rates) of the MSCs with different narrow gaps.

The narrower the gaps of electrodes are, the faster ion transfer and charge and discharge are, which considerably contributes to electrochemical performance. In the actual measurement, a gap of a few hundred nanometers can cause collision losses of electrode materials, which affects charge transfer and electrochemical performance. The electrochemical performance of MSCs with different narrow gap was plotted against various scan rates (Figure R3.9).

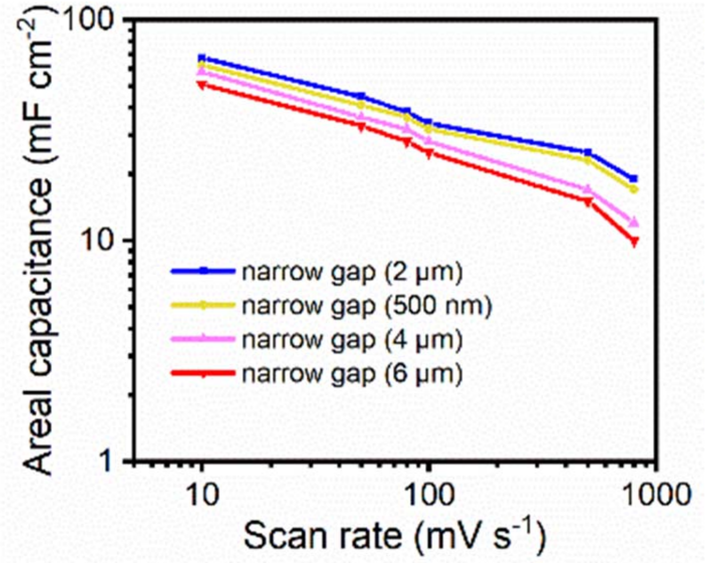

Figure R3.9 The areal capacitance of the MSCs with different narrow gaps as a function of scan rates

The results of our experiments revealed that MSCs with different narrow gaps differences from the areal capacitance in the case of a lower scan rate. When the narrow gap was 2 μm, the highest areal capacitance was 67 mF cm^{-2}. The areal capacitance was 62, 58, and 51 mF cm^{-2} at the corresponding gaps of 4 μm, 500 nm, and 6 μm.

(3) In addition to the aforementioned two parameters, we studied the effect of the thickness of MSCs on electrochemical performance. We prepared graphene composite films of different thicknesses to process MSCs. The SEM image of the crosssection view of GO hybrid films of different thicknesses is provided in figure R3.10.

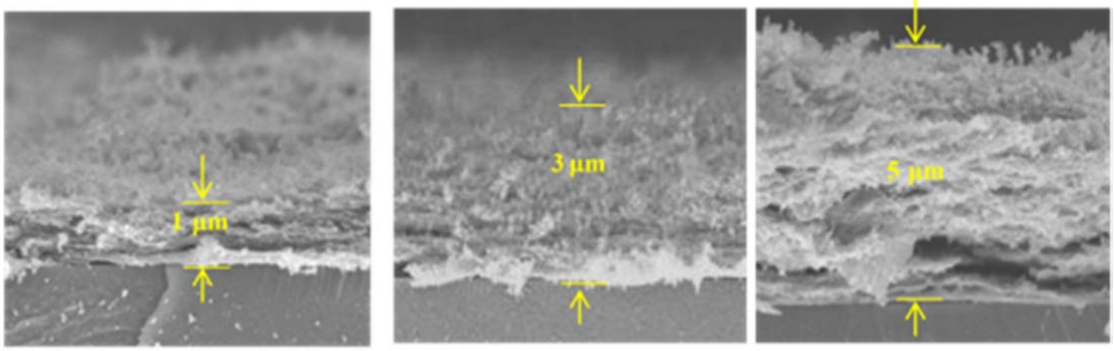

Figure R3.10 SEM image of cross section view of the hybrid film with different thicknesses

For a micro energy storage device, the optimum use of the limited room is critical to boost the areal capacitance and energy density. The 3D expandability of MSCs based on planar configuration is crucial. The increase in the thickness improves the areal capacitance and energy density, which is consistent with our goals. We also have to consider the volumetric capacitance and energy density.

Figure R3.11 (a) and (b) displays the CV characterizations of MSCs with different thicknesses at 80 and 800 mV s^{-1}. As expected, the thicker the MSC is, the larger the areal capacitance is. The areal capacitances of the MSCs with thicknesses of 1, 3, and 5 μm were 13, 38, and 64 mF cm^{-2} at 80 mV s^{-1}. At high scan rates, the difference in the areal capacitance caused by thickness gradually decreases. The MSC with a thickness of 5 μm has the largest areal capacitance.

Figure R3.11 (c) and (d) depicts the areal and volumetric capacitance of MSCs with different thicknesses as functions of the scan rate. The higher the thickness of the MSC is, the higher the capacitance performance is. The influence of thickness on

volumetric capacitance is more intuitive. Therefore, the volumetric capacitance can better explain the true influence of thickness on performance than capacitance. We determined that the volumetric capacitance of MSCs of different thicknesses were very close. At a low scan rate, the volumetric capacitance of MSCs of 3 μm is slightly higher than those of the other two. At a high scan rate, the thickness of the 1-μm supercapacitor exhibits superior performance.

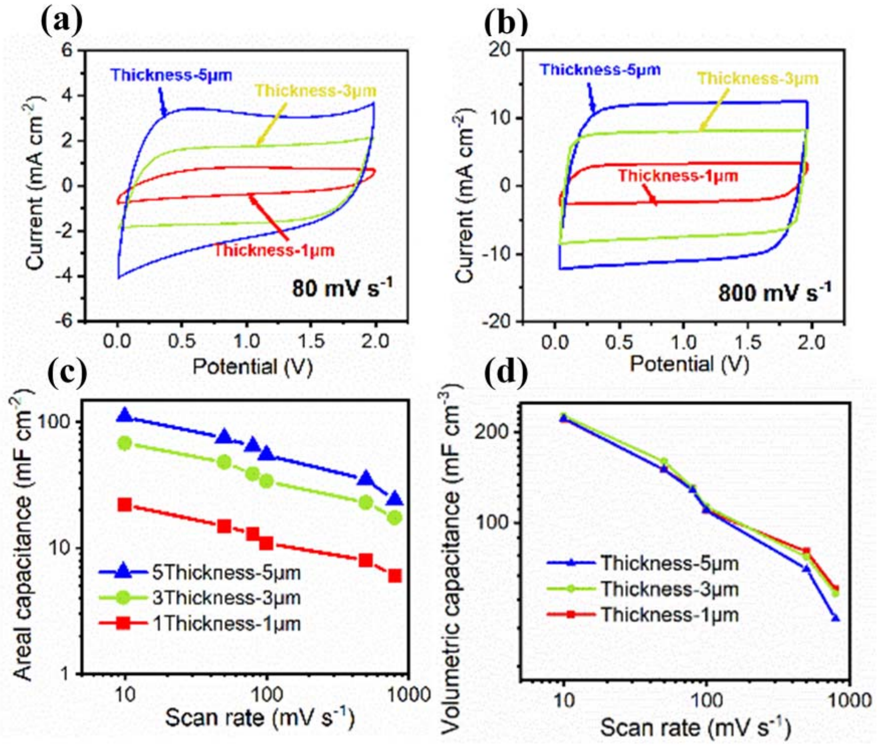

Figure R3.11CV characterizations at low (a) and high (b) scan rates of the MSCs with different thicknesses. (c) and (d) Areal and volumetric capacitance of MSCs with different thicknesses as the functions of the scan rate

(4) We used the Ti:sapphire laser regenerative amplifier system that can generate 1,000 single pulses per second. In theory, each single pulse can achieve MSC processing; consequently, a single MSC can be fabricated in only 0.001 s. During actual processing, we processed >50 MSCs per second.

Traditional laser direct writing improves the efficiency of the fabrication of MSCs and other microdevices and presents the advantages of a mask-free process,

flexibility, and high resolution. The laser point-by-point writing of the focal spot is realized by controlling the movement of the translation stage. Therefore, in actual processing, we must consider the stability of the translation stage and repeated positioning accuracy to ensure processing consistency, and achieving multipatterned rapid processing is difficult.

The shaped femtosecond laser can be formed in one step, and shapes can be changed by changing phase patterns. The spot of the shaped laser is a designable pattern spot that can directly and instantaneously achieve the fabrication of patterned MSCs. In addition to the advantages of laser direct writing, this technology considerably improves processing efficiency and can enable the consistency of large-area processing. In theory, this process is completed in an instant.

Therefore, in our processing, changing the speed of laser scanning to influence the processing effect is not required. The whole process is completed in 0.001 s. Thus, the “speed of the laser” in our experiment is equivalent to the speed of laser patterning, which is considerably rapid and stable.

Modifications

According to the comments of the reviewer, we have added the impact of parameters on electrochemical performance.

The added part is inserted following Page 16, Paragraph 3, Line 2 is as following:

“On the basis of our findings, we selected the optimal parameter configuration of the interdigital MSCs for further electrochemical tests. We performed a series of parameter research and optimizations for the interdigital MSCs. We investigated the interdigital gap width, device dimensions, and thickness of the MSCs (Supplementary 21-23) to explore the effects of these factors on electrochemical performance.”

The corresponding content added in Supporting Information is as following:

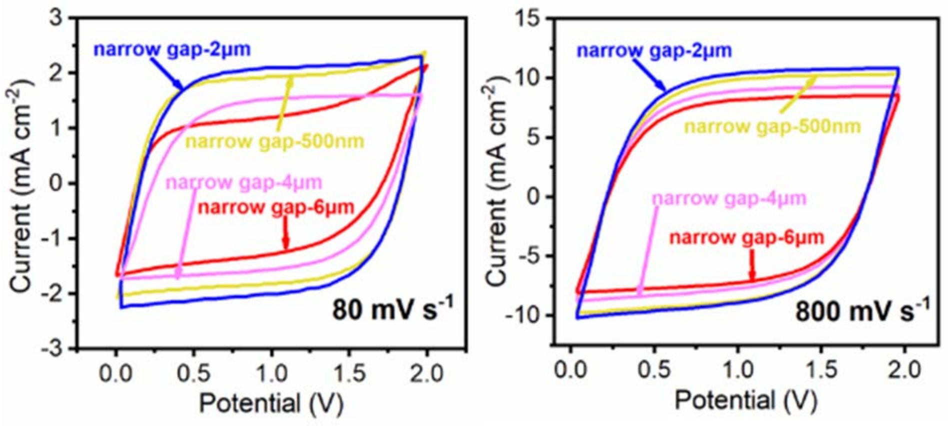

Supplementary Figure 21-1: Comparison of the electrochemical performance (CV characterizations at 80 and 800 mV/s) of MSCs with different narrow gaps. CV curves were recorded at variable scan rates to evaluate the effect of different microscale interdigital narrow gaps in the MSCs. As shown, the CVs of the high-resolution MSCs maintained a rectangular shape at different scan rates. When the interdigital narrow gaps were 500 nm, 2 μm, 4 μm, and 6 μm, different electrochemical performances are obtained. Of these, the MSCs with a narrow gap of 2 μm exhibited the optimal electrochemical performance, slightly higher than that with a gap of 500 nm and higher than those with gaps of 4 and 6 μm.

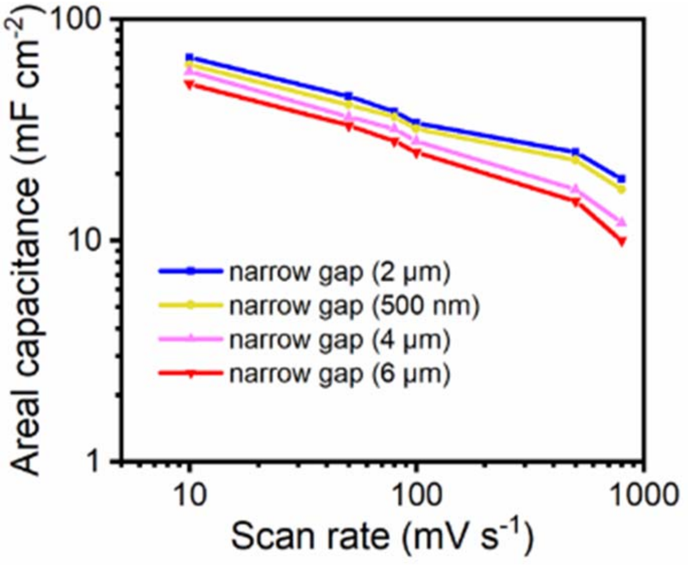

Supplementary Figure 21-2: Areal capacitance of MSCs with different narrow gaps as a function of scan rates. Our measurements and calculations showed that MSCs with different narrow gaps exhibited differences from areal capacitance under a

lower scan rate. The highest areal capacitance of 67 mF/cm^2 was obtained when the narrow gap was 2 μm. Areal capacitances of 62, 58, and 51 mF/cm^2 were obtained for narrow gaps of 500 nm, 4 μm, and 6 μm, respectively.

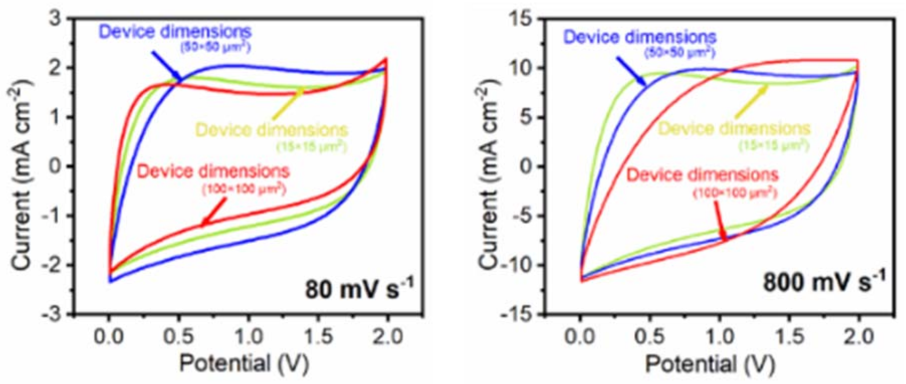

Supplementary Figure 22-1: Comparison of electrochemical performance (CV characterizations at 80 and 800 mV/s) of MSCs with different device dimensions. To understand and analyze the effect of device dimensions on electrochemical performance, we tested three sizes (15 × 15 μm^2, 50 × 50 μm^2, 100 × 100 μm^2) of MSCs. To increase the accuracy of the measurements, we simultaneously measured the size of a dozen micron miniature supercapacitor requirements. We customized a probe with a contact diameter of 3 μm for use with a 100× confocal microscopy system. CV curves are shown at low and high scan rates for MSCs with different device dimensions. Contrary to expectations, the areal capacitances of different device dimensions were extremely similar at high or low scan rates. An areal capacitance of 38 mF/cm^2 was achieved at 80 mV/s when the device dimension was 50 × 50 μm^2, which was slightly higher than the areal capacitances of 37 and 35 mF/cm^2 achieved when the device dimensions were 15 × 15 μm^2 and 100 × 100 μm^2, respectively. When the scan rate was increased to 800 mV/s, the areal capacitance was almost the same (18 mF/cm^2).

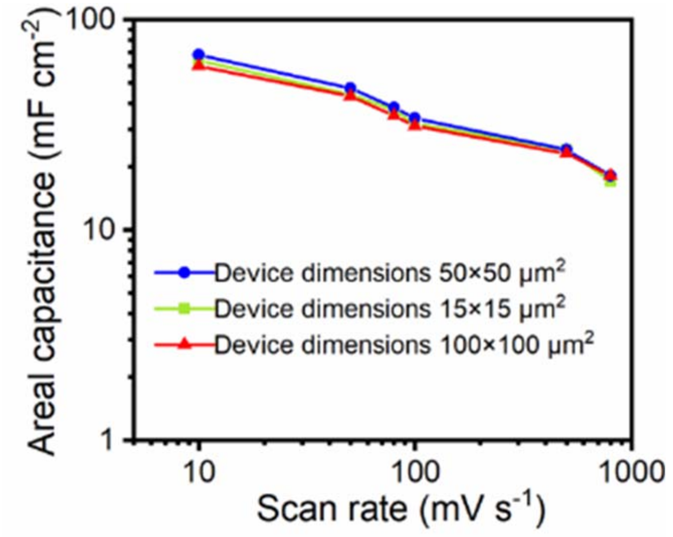

Supplementary Figure 22-2: Areal capacitance of MSCs with different device dimensions as functions of the scan rate. This figure shows the effect of device dimensions on electrochemical performance. When the scan rate is low, the dimension has little influence on the performance of the MSC. Thus, when the scan rate is 10 mV/s, the MSC with dimensions of 50×50 μm^2 has an areal capacitance of 68 mF/cm^2.Those with dimensions of 15 × 15 μm^2 and 100 × 100 μm^2 have areal capacitances of 64 mF/cm^2 and 60 mF/cm^2, respectively. Notably, as the scan rate increases, these differences in electrochemical performance disappear. The results showed that the dimensions of the MSC had an effect on the electrochemical performance at low scan rates, but this was almost negligible at high scan rates. However, the MSC with dimensions of 50 × 50 μm^2 exhibited uniformly good electrochemical performance at both high and low scan rates.

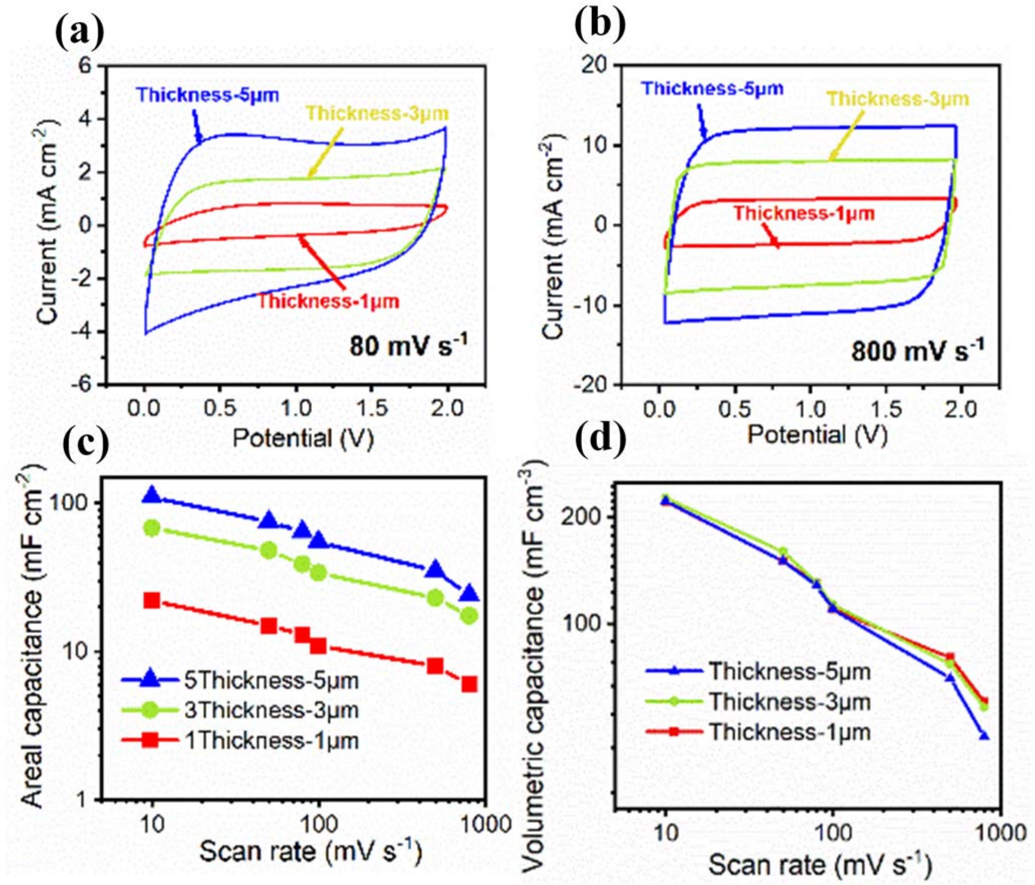

Supplementary Figure 23：Comparison of CV characterizations at (a) low and (b) high scan rates of MSCs with different thicknesses, as well as (c) areal and (d) volumetric capacitances of MSCs with different thicknesses as functions of the scan rate. Figure R14 (a) and (b) depicts the CV characterizations of MSCs with different thicknesses at 80 mV/s and 800 mV/s. As expected, the thicker the MSC, the larger the areal capacitance. The areal capacitances of MSCs with thicknesses of 1, 3, and 5 μm were 13, 38, and 64 mF/cm^2, respectively, at 80 mV/s. As the scan rate increases, the difference in areal capacitance caused by thickness gradually decreases. The MSC with a thickness of 5 μm exhibited the highest areal capacitance. Figure 14 (c) and (d) depicts the areal and volumetric capacitances of MSCs with different thicknesses as functions of the scan rate. With respect to areal capacitance, the higher the thickness of the MSC, the higher the capacitance. However, for volumetric capacitance, the influence of thickness is more intuitive. Therefore, volumetric

capacitance can better explain the true influence of thickness on performance. The volumetric capacitances of MSCs of different thicknesses are extremely similar. Specifically, at a low scan rate, the volumetric capacitance of a MSC with a thickness of 3 μm is slightly higher than that of the other MSCs. At a high scan rate, the supercapacitor with a thickness of 1 μm has the advantage.

3.4 Reviewer's Comments

3.4. "Manufacturing technology is not the proper phrase for this manuscript. To do this several large scale optimization required at different batch."

Our Response

We thank the reviewer for carefully checking the paper in detail and for the suggestion to improve the paper quality. Especially in terms of the professional vocabulary grasp, which highly beneficial us, but also allows us have a more rigorous attitude toward scientific words.

Because of the reviewer's reminder, we realized that "manufacturing technology" is really not a proper phrase for this manuscript. According to the Oxford dictionary, "manufacture" means the making of articles on a large scale using machinery. This is contrary to the central idea of our manuscript.

As the reviewer indicated, our study is actually narrowing down the device geometry and area rather than focusing on bulk-scale production. We prefer to emphasize our innovative proposal of a microfabrication technique that enables ultrafast fabrication of microelectronic devices. We apologize for the misuse of this word. We have revised the word "manufacturing technology" in the revised manuscript. Microfabrication may be an appropriate word choice and may highlight the innovation of our study. Simultaneously, this word provides readers a clear understanding of this novel ultra-efficient technology proposed for the micro–nano field.

Modifications

According to the reviewer's comments, we have revised the corresponding part in our manuscript.

The original title is as follows:

"Ultrafast laser-stamping for MSCs manufacturing"

The title is revised as following:

"Laser photonic-reduction stamping for graphene-based MSCs ultrafast fabrication"

The original part in Page2, Paragraph 2, Line 5, the detail is as following:

"the manufacturing accuracy"

This part is revised as following:

"the fabrication accuracy"

The original part in Page2, Paragraph 2, Line 10, the detail is as following:

"the manufacturing efficiency"

This part is revised as following:

"the fabrication efficiency"

The original part in Page4, Paragraph 2, Line 1, the detail is as following:

"manufactured technology for L-SLMSCs via SFLS strategy"

This part is revised as following:

"Micro fabrication of the MSCs via SSFL"

The original part in the Table in Page 6 is as following:

"manufacturing"

This part is revised as following:

"fabrication"

3.5 Reviewer's Comments

3.5. "In my opinion, this work is actually narrowing down the device geometry and area rather focusing the bulk scale production. In this respect, I am curious to know about the gravimetric capacitance and volumetric capacitance, which an Industry may look into it.

In Fig. 4, authors have demonstrated shape versatility of MSC devices. I doubt all the devices that have the same geometrical are or not. Although they have normalized it with the area, however different areas will contribute to different mass loading of the devices. Therefore it is recommended to keep a similar area of the

devices and compare the gravimetric capacitance as well during the optimization of shape versatility."

Our Response

The detailed comments are very helpful and greatly valued. We thank the reviewer for the suggestion for improving the paper quality.

The reviewer's suggestions regarding the industrial application of our MSCs are very meaningful, and we have incorporated the industrial applications of our technology in the manuscript. Therefore, providing a highly accurate evaluation of the gravimetric and volumetric capacitance in this manuscript is necessary. According to the reviewer's comments, we carefully studied the gravimetric and volumetric capacitance of MSCs through experiments. Simultaneously, we maintained a similar area of devices and compared the gravimetric capacitance during shape versatility optimization.

(1) Volumetric capacitance of the MSCs fabricated using a shaped femtosecond laser. We selected three typical MSC shapes—concentric circle, parallel strip, and interdigital—and tested their volumetric capacitance. Figure R3.12 reveals the cyclic voltammetry (CV) curves of different patterns acquired at different scan rates. Irrespective of high and low scan rates, the three types of MSCs with different shapes maintained rectangular CV curves under a voltage window of 0.5 V, proving that the capacitance of the MSCs processed through our strategy is considerably excellent. By contrast, concentric circle-shaped and interdigital MSCs exhibited more regular rectangular curves than parallel strip-shaped MSCs did.

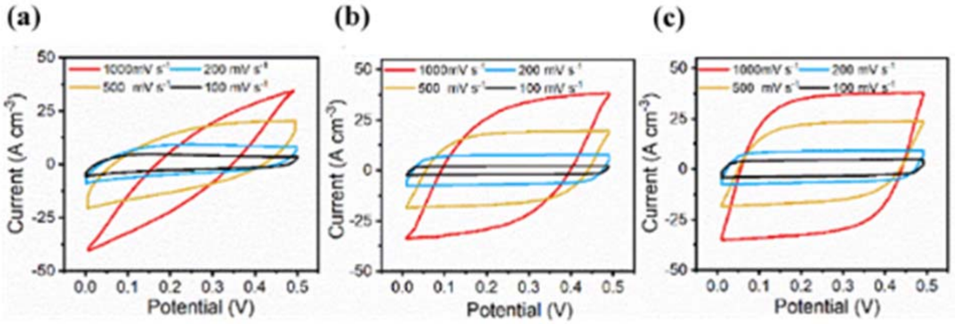

Figure R3.12 Electrochemical characterization of MSCs with different geometries. CV curves of the parallel strip (a), concentric circle (b), and interdigital (c) MSCs at the scan rate from

100 to 1000 mV s^{-1} in 0.5 M Na_2SO_4.

Figure R3.13 presents the volumetric capacitance of versatile-shaped MSCs at diverse scan rates: interdigital MSCs always exhibit a high volumetric capacitance. The volumetric capacitance of the interdigital MSCs is 131 F cm^{-3}, which is higher than that of the parallel strip (110 F cm^{-3}) and concentric circle (116 F cm^{-3}) MSCs.

We concluded that our MSCs were probably micron-scaled, and efficient ion and charge transfer between electrode materials and electrolyte solutions were crucial. In interdigital MSCs, the electrode material area and volume highly were efficiently employed, and the contact area between electrode material and electrolyte was increased; because of the short diffusion distance between ions, electrolyte ions are placed in a narrow gap between electrodes, which makes its transportation easy and results in higher performance[R27].

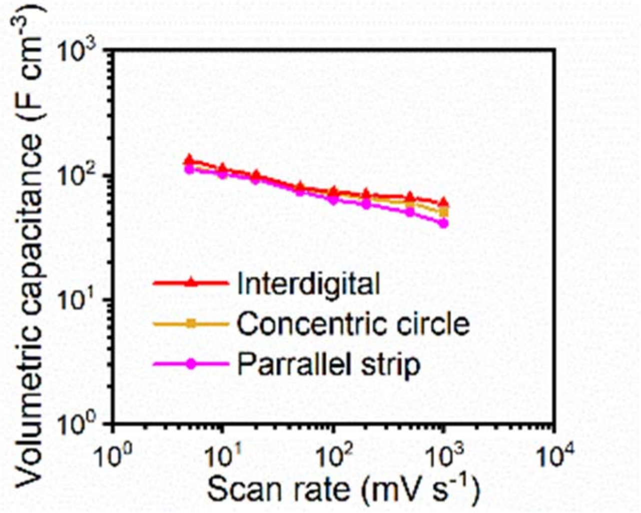

Figure R3.13 The volumetric capacitance of three different geometries of MSCs at diverse scan rates.

Furthermore, to demonstrate the excellent volumetric capacitance of interdigital MSCs, we tested CV curves under high voltage windows. We found that they could maintain the rectangular shape under a voltage window of 2 V, and they provided ultrahigh volumetric capacitances (Figure R11).

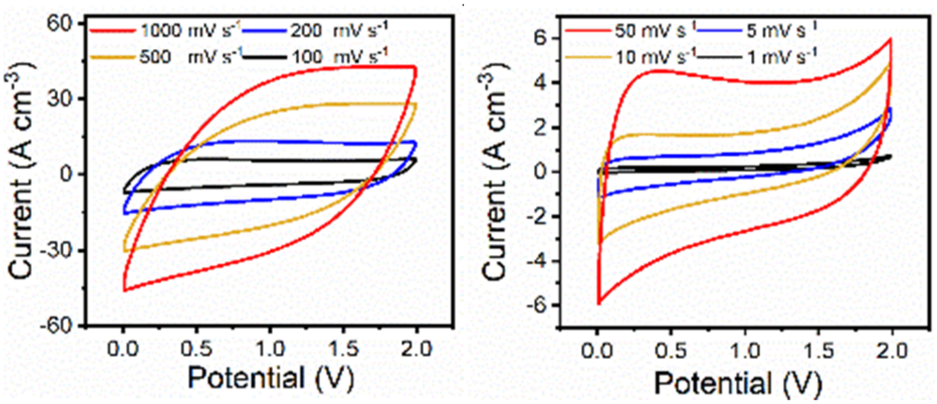

Figure R3.14 CV curves of interdigital MSCs at scan rates ranging from 1 to 1000 m V s^{-1} under 2 V voltage windows

The volumetric capacitance of MSCs varies with scan rates, and its highest volumetric capacitance is 426 F cm^{-3} (Figure R3.14). This performance is excellent. Our technology for ultrafast preparation of MSCs with ultrahigh volumetric capacitance provides broad opportunities for the industrial application of miniature supercapacitors.

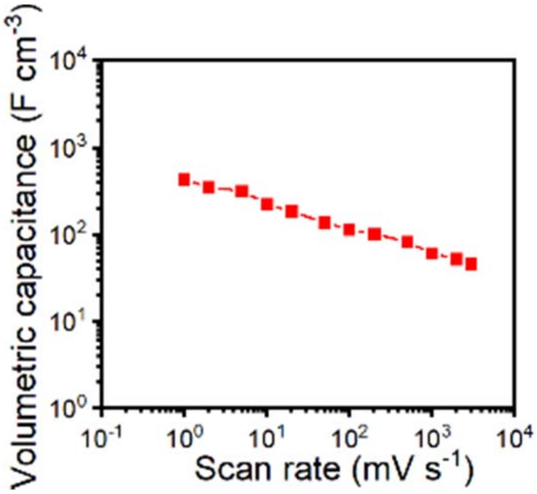

Figure R3.15 The volumetric capacitance under different scan rates

(2) (2) The gravimetric capacitance of the MSCs fabricated using the shaped femtosecond laser is vital for its industrial application. The specific capacitances (C, F g^{-1}) of MSCs are calculated from the following equation[R28]

$$C = \frac{\int_{V1}^{V2} I(V)dV}{\vartheta(V_2 - V_1)m} \quad \text{(E3-1)}$$

where *I(V)* is the average cathodic current (*A*) of the CV loop, ϑ is the scan rate

(mV s^{-1}), and *m* is the active mass (*g*) of both electrodes' material. The term in the numerator represents the area of the *I*−*V* curve, and V_1 and V_2 are the switching potentials (*V*) in the CV curve.

According to the reviewer's comments, "different areas will contribute to different mass loading of devices; therefore, we must maintain a similar area of devices and compare the gravimetric capacitance during shape versatility optimization." As aforementioned, we normalized the area to study the effect of shapes on the electrochemical capacitance. Therefore, we have presented the schematics of the different shapes of MSCs (Figure R3.16). All the MSCs of different shapes are of the 50 × 50 μm^2 size. However, although their area distribution is in the range of 50 × 50 μm^2, the mass loading of their effective electrode materials is different.

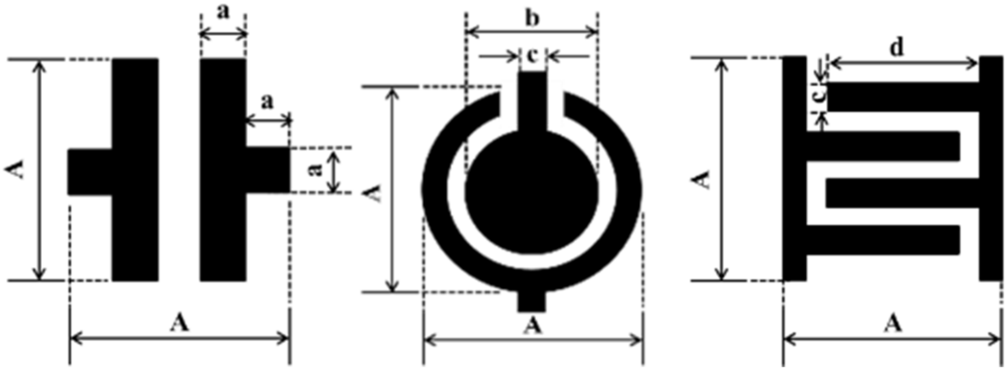

Figure R3.16 The detailed dimensions of three shapes of MSCs

According to the detailed dimensions of the three patterns, we can calculate the different mass loadings of single MSCs. The values of A, a, b, c, and d are 50 μm, 10 μm, 30 μm, 6 μm, and 35 μm, respectively (Figure R13). The mass ratio (R) of effective electrode materials for a single-parallel strip, concentric circle, and interdigital MSCs is 0.48, 0.56, and 0.53, respectively, according to the following equation:

$$R = \frac{m_e}{m_t} = \frac{\rho s_e h}{\rho S_t h} \quad \text{(E3-2)}$$

where m_e is the effective electrode mass, m_t is the total mass in the range area,

S_e is area of the effective electrode material, S_t is the area of the normalized area, ρ is the density of the film of the electrode, h is the thickness of the film. We prepared tens of thousands of MSCs by using quantitative electrode material. According to the mass ratio of different shapes, we can accurately calculate the mass loading of a single MSC of each shape and obtain the gravimetric capacitance.

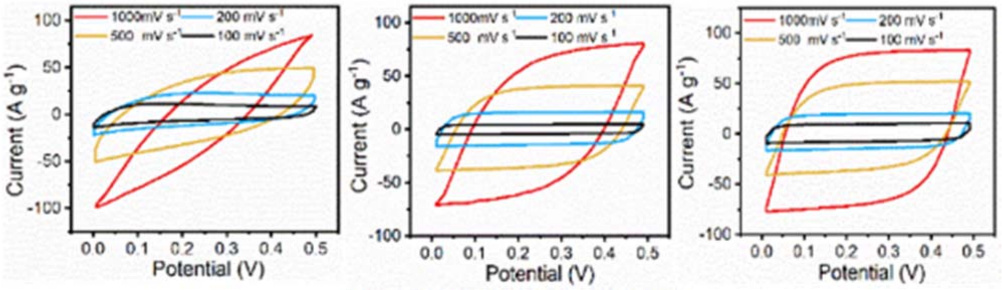

Figure R3.17 The CV curves of MSCs with different geometries. (a) parallel strip, (b) concentric circle, (c) interdigital MSCs at different scan rates.

CV curves indicate that we can calculate the gravimetric capacitance according to Figure R3.17; MSCs with different shapes have different gravimetric capacitance. Interdigital MSCs have the highest gravimetric capacitance of up to 290 F g^{-1} under a voltage window of 0.5 V (Figure R3.18). Moreover, parallel strip MSCs exhibit an excellent gravimetric capacitance (272 F g^{-1}), and its gravimetric capacitance is even higher than that of concentric circle MSCs at the same scan rates.

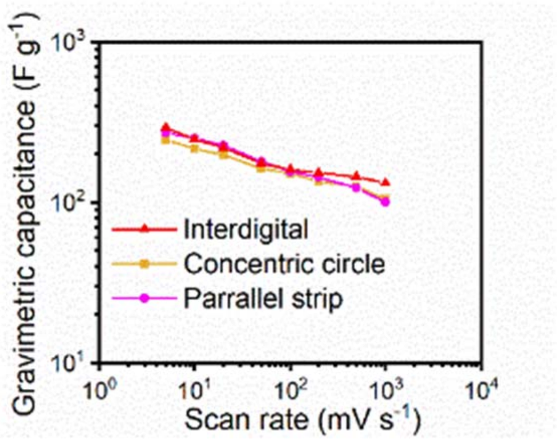

Figure R3.18 The gravimetric capacitance of three different geometries of MSCs at diverse scan rates.

To further explore the gravimetric capacitance of the prepared miniature

supercapacitors, we conducted the electrochemical test of interdigital MSCs at a voltage window of 2 V and calculated the gravimetric capacitance. Figure R3.19 presents CV curves obtained under high voltage windows at different scan rates.

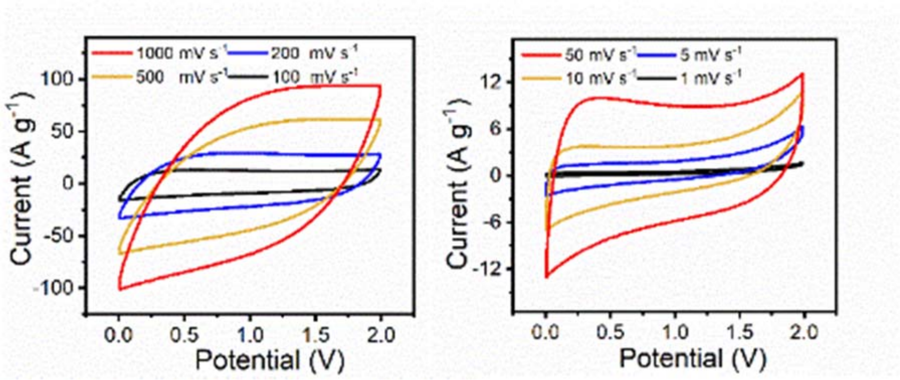

Figure R3.19 CV curves of interdigital MSCs at scan rates ranging from 1 to 1000 m V s^{-1}

The gravimetric capacitance of MSCs varies with scan rates, and the highest gravimetric capacitance is 941 F g^{-1} (Figure R3.20). Thus, interdigital MSCs have excellent gravimetric capacitance during shape versatility optimization. This result is consistent with the previous analysis in which the areal and volumetric capacitance was compared. However, the difference is that the parallel strip MSC with a simpler structure and lower mass loading exhibits a higher gravimetric capacitance than the concentric circle MSC does.

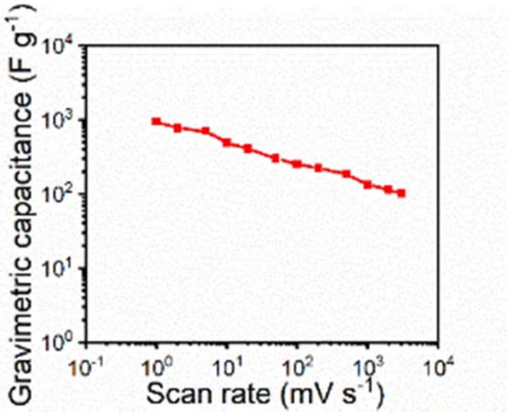

Figure R3.20 The gravimetric capacitance under different scan rates

Modifications

According to the reviewer's comments, we have revised the corresponding text in our manuscript.

The original part in Page 14, Paragraph 1, Line, 7 the detail is as following:

"Fig. 4g depicted the capacitance of versatile-shaped L-S-MSCs at diverse scan rates: interdigital L-S-MSCs always have the higher areal capacitance."

This part is revised as following:

"Fig. 4g and Supplementary Fig.17 depicted the areal and volumetric capacitance of versatile-shaped MSCs at diverse scan rates: the interdigital MSCs always have the higher capacitance performance."

The added part is inserted in Page 14, Paragraph 1, Line, 8 the detail is as following:

"Considering that gravimetric capacitance is a critical factor for industrial applications, we calculated the mass loading (Supplementary Fig. 18) and obtained the corresponding gravimetric capacitance (Supplementary Fig. 19) of MSCs with different shapes but the same size at several scan rates. The interdigital MSC exhibited the optimal gravimetric capacitance; however, the parallel strip MSC exhibited excellent gravimetric capacitance, higher than the gravimetric capacitance of the concentric circle MSC. This finding indicates that the shape design of MSCs affected performance. In interdigital MSCs, electrode material areas are used more efficiently and the contact area between the electrode material and electrolytes is greater. Furthermore, interdigital MSCs are interlaced with electrode materials that can shorten the ion diffusion pathway by narrowing the width of the fingers in the MSC and increasing the length of the interface between the active-material electrode and the electrolyte. Therefore, appropriately designing the shapes of an MSC is conducive to fast ion transfer rate, rapid charge and discharge, improved double layer storage, and enhanced rate capability[53]."

The corresponding content added in Supporting Information is as following:

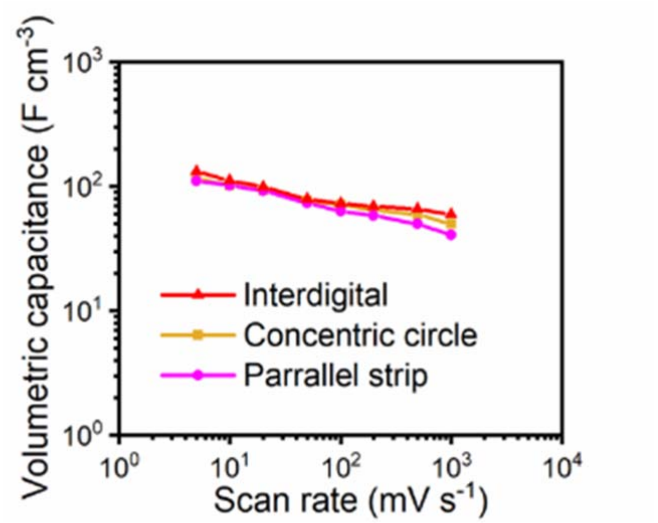

Supplementary Figure 17: Volumetric capacitance of three different geometries of MSCs at diverse scan rates. Figure R10 depicts the volumetric capacitance of versatile-shaped MSCs at diverse scan rates. At 131 F/cm^3, interdigital MSCs have the highest volumetric capacitance compared with the parallel strip (110 F/cm^3) and concentric circle (116 F/cm^3) MSCs. We concluded that our MSCs are probably micron-scale and that efficient ion and charge transfer between electrode materials and electrolyte solutions is more important. Interdigital MSCs utilize the electrode material area and volume more efficiently and increase the contact area between the electrode material and electrolyte; due to the short diffusion distance of ions, electrolyte ions are placed in the narrow gap between electrodes. This makes them easy to transport and results in higher performance.

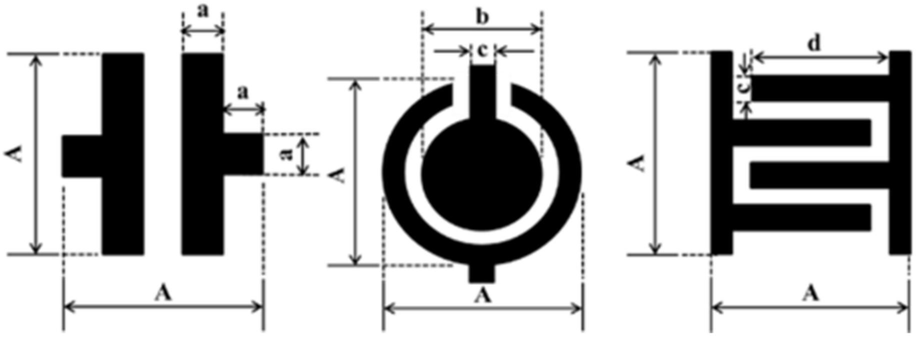

Supplementary Figure 18: Detailed dimensions of three shapes of MSCs. The different shaped MSCs are all 50 × 50 μm^2 in size. However, although their area

distribution is in the range 50 × 50 μm^2, the mass loading of their effective electrode materials is clearly different. Based on the detailed dimensions of the three patterns, we calculated the different mass loadings of a single MSC. The values of A, a, b, c and d were 50, 10, 30, 6, and 35 μm, respectively. The mass ratio (R) values of effective electrode materials for a single parallel strip, concentric circle, and interdigital MSCs were 0.48, 0.56 and 0.53, respectively. These were determined using the following equation:

$$R = \frac{m_e}{m_t} = \frac{\rho s_e h}{\rho S_t h}$$

where m_e is the effective electrode mass, m_t is the total mass in the range area, s_e is area of the effective electrode material, s_t is the area of the normalized area, ρ is the density of the film of the electrode, h is the thickness of the film. In our work, we prepared tens of thousands of MSCs using a quantitative electrode material. According to the mass ratio of different shapes, we can accurately calculate the mass loading of a single MSC of each shape and obtain the gravimetric capacitance.

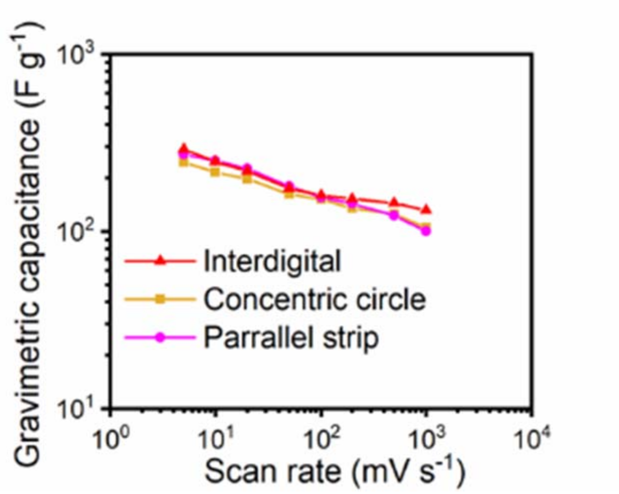

Supplementary Figure 19: Gravimetric capacitance of three different geometries of MSCs at diverse scan rates. As shown, the interdigital MSC has the highest gravimetric capacitance of up to 290 F/g at a voltage of 0.5 V. Confounding our expectations, the parallel strip MSCs exhibited excellent gravimetric capacitance (272

F/g), which was even higher than that of the concentric circle MSCs at the same scan rates.

3.6 Reviewer's Comments

3.6. "When MSC manufacturing is focusing on the manuscript, one should check the stability of the devices with respect to cycling performances."

Our Response

We truly appreciate the reviewer for their efforts for reviewing our paper. The stability of devices is of importance in both the process and measurement of electrochemical performance. Therefore, we ensured the experimental stability and data reliability from three aspects.

(1)Stability during MSC processing

As discussed earlier, we used the spatially shaped femtosecond laser to fabricate thousands of MSCs in a substantially short duration. For the process, an image loaded using a computer is used to shape the Gaussian beam emitted from the Ti:sapphire laser regenerative amplifier system, and the resulting shaped femtosecond laser directly processes GO hybrid films. Before each experiment, we calibrated the laser and evaluated its parameters to ensure that its performance was ideal. Figure R3.21 presents the interface to verify the laser state. We can only conduct experiments when all settings work normally.

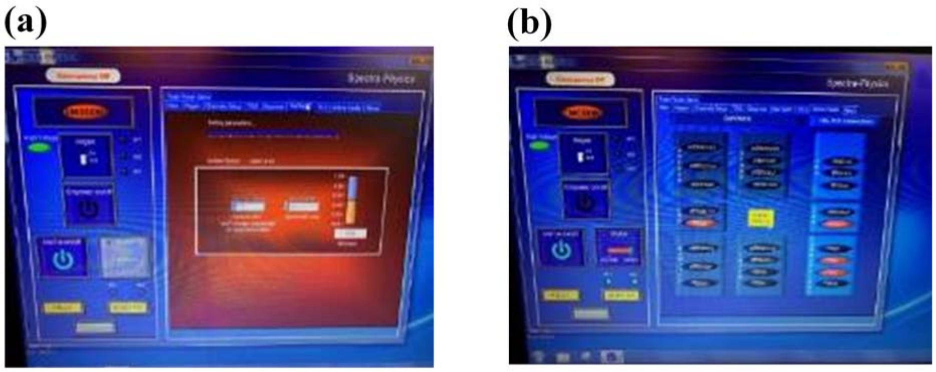

Figure R3.21 Stability of laser parameters, (a) the measurement of laser output power stability, (b) the normal operation and stability detection of each component in the laser system

Subsequently, we tested the power of the laser outlet to ensure processing stability. After the laser beam was emitted from the laser source, we collected the laser in an optical path and tested its distribution uniformity. Figure R 3.22 presents the tests by measuring the optical port power and detecting the laser quality. We obtained a uniform and high-intensity light field by optimizing the distribution of the original laser with poor quality and collected and detected the distribution of light field in real time by using a beam quality meter to ensure the stability of the light field in the experiment [Figure R3.22 (a), (b), (c), and (d)].

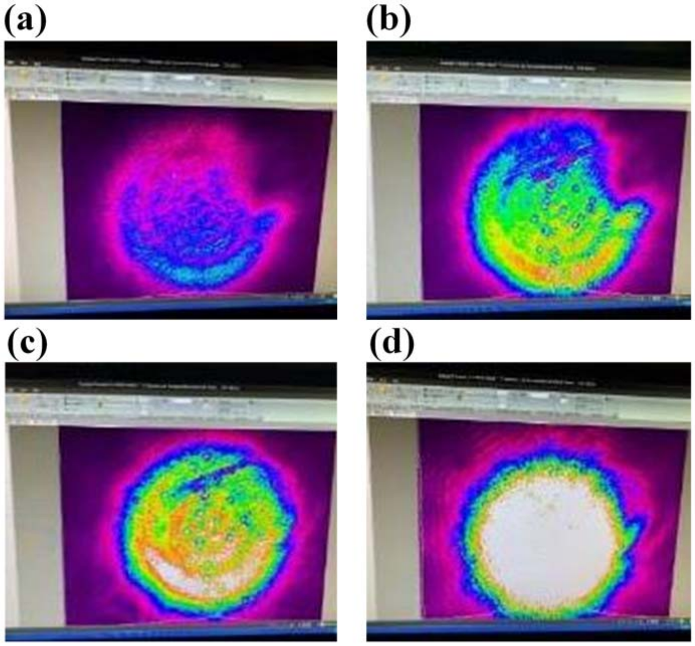

Figure R3.22 Stability and beam quality detection of laser, (a) the initial beam with an uneven distribution and low energy, (b) the distribution of the light field after increasing the intensity of the beam, (c) the distribution of light field after increasing intensity and adjusting the uniformity of the beam, (d) further optimizing the distribution of the beam

In addition, we purified air to eliminate particulate matter from the experimental area where the laser was situated and conducted daily inspection, and humidity and temperature were controlled. Figure R3.23 illustrates the environment of the experimental area and the daily temperature and humidity monitoring.

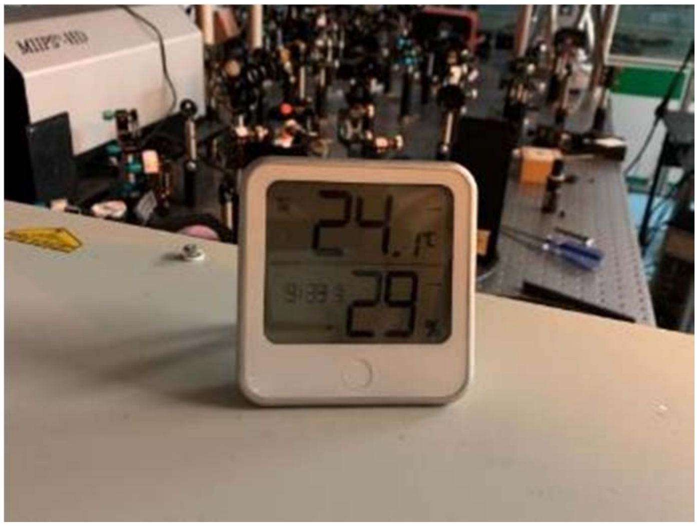

Figure R3.23 Real-time monitoring of temperature and humidity

In this manner, we can guarantee the repeatability of the entire process and the stability of the processing equipment.

(2) Stability of MSCs

For the practical application of miniature capacitors, the cycle life is crucial. Cycling stability with high performance remains a challenge mainly because of the easy fracture of thick electrodes during repeated charging and discharging[R29].

In the manuscript, we have described that the prepared composite film exhibits large toughness and high flexibility (Figure 2). The structure of the 3D graphene skeleton was constructed using the femtosecond laser in one step; simultaneously, the attached manganese dioxide particles were synthesized. This 3D porous structure is highly stable and is not easily destroyed during electrode charging and discharging[R30]. Because of minimum size of the MSC, our prepared MSCs can attain a size of 10 microns, and their narrow gap can reach 500 nm; thus, transfer between ions was rapid and the transfer path was short, which reduced electrode instability in the electrolyte solution.

To demonstrate the cycle life of our MSCs, the same MSCs were sequentially subjected to 6,000 GCD cycles under different voltage windows. Figure R3.24 presents the capacitance retention of MSCs under different voltage windows. Inset: five GCD curves of interdigital MSCs before and after cycling for 6,000 times.

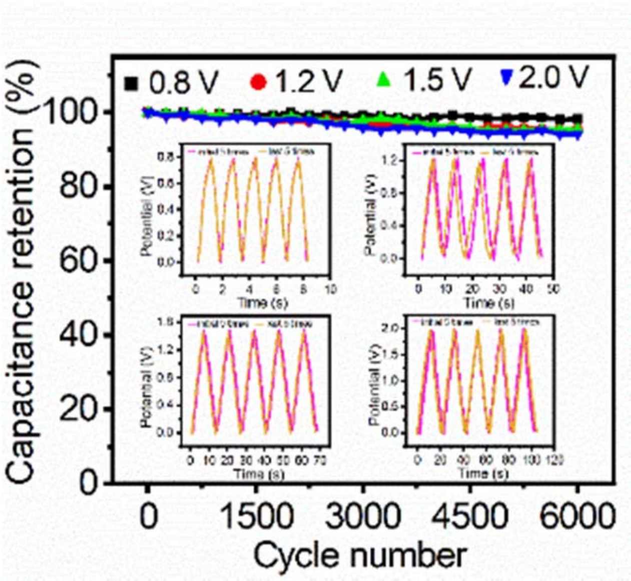

Figure R3.24Cycle life of the MSCs under different voltage windows.

After 6,000 cycles, the GCD curve remained almost identical to the GCD curve in the first cycle. When the voltage window was low, the capacitance retention was close to 100%, and when the voltage window was 2 V, the capacitance retention rate was above 93%. This proves that our MSCs exhibit excellent cycling stability.

To verify MSC stability, we conducted electrochemical tests over 30 days on the miniature supercapacitor fabricated. After 30 days, the miniature supercapacitor exhibited almost the same electrochemical performance as the freshly prepared MSC did. Under the same scanning rate, the CV curves of the two supercapacitors almost coincided, which indicated that the supercapacitor prepared using the shaped femtosecond laser exhibits highly stable capacitance characteristics (Figure R3.25).

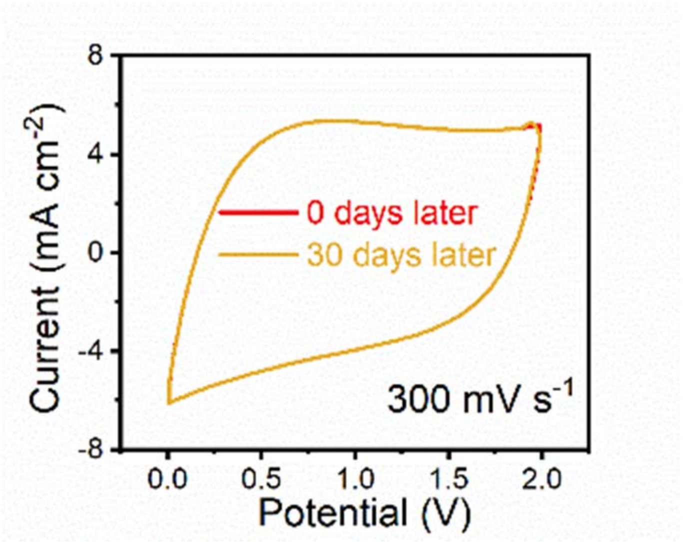

Figure R3.25 CV curves of the fabricated MSCs after 0 and 30 days

Furthermore, we tested the CV curves of the same single interdigital MSC at various bending states (Figure R3.26). All CV curves were almost coincident. Even the CV curve of the MSC was measured after 180° bending, and it was almost the same as the CV curve after 0° bending.

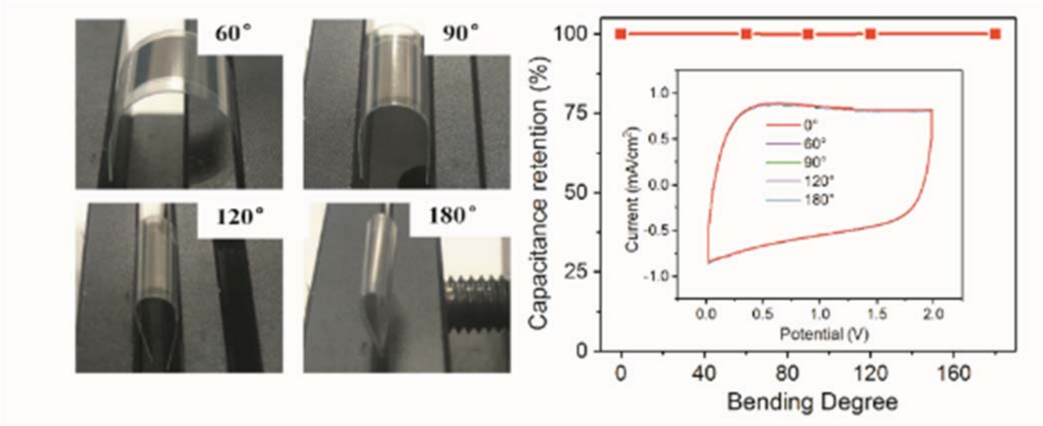

Figure R3.26 Displaying the mechanical stability of MSC

The outstanding stability can extend MSC use in numerous fields, including integrated circuits, wearable microelectronics, and medical devices.

(3) Stability of devices during electrochemical testing

Electrochemical testing was performed at a CHI760E electrochemical workstation connected through a precision probe station (MPS-100S) with a microscopic system using tungsten probes (tip diameter = 5 μm) as current collectors.

To ensure a stable electrochemistry environment, the open-circuit potential (Eocp) was measured for 1 h until fluctuations were <10 mV in 10 min before every electrochemistry measurement. The electrochemical performance of MSCs was measured in a two-electrode system. In addition, our electrochemical workstation performed self-detection and troubleshooting after start-up and measured the rated resistance before and after electrochemical testing to ensure that no errors occurred. Figure R3.27 illustrates the self-measurement of the electrochemical workstation and the I/V curve obtained when measuring the fixed resistance to ensure the stability of the process of testing supercapacitor performance.

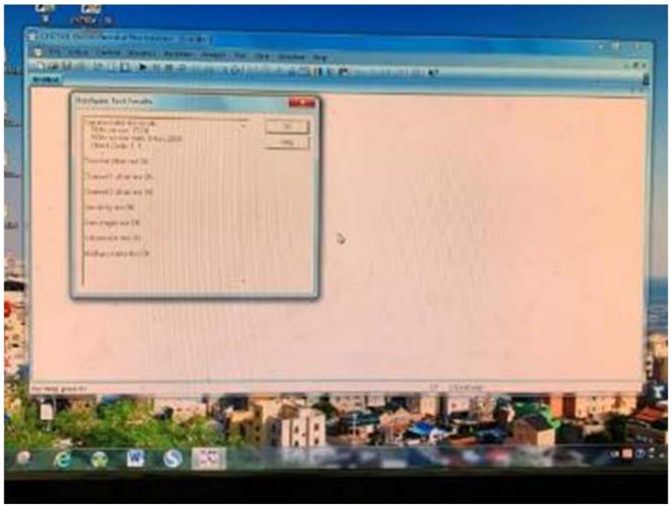

Figure R3.27 Self-check the stability of electrochemical workstation before each measurement

Modifications

According to the reviewer's comments, we have revised the corresponding text in our manuscript.

The added part is inserted in Page 19, Paragraph 3, Line, 1 the detail is as following:

"The electrochemical tests were carried on the MSC fabricated over 30 days and the capacitance performance is almost the same as the newly prepared MSC, which demonstrates the super stability of our MSCs (Supplementary Fig, 27)."

The corresponding content added in Supporting Information is as following:

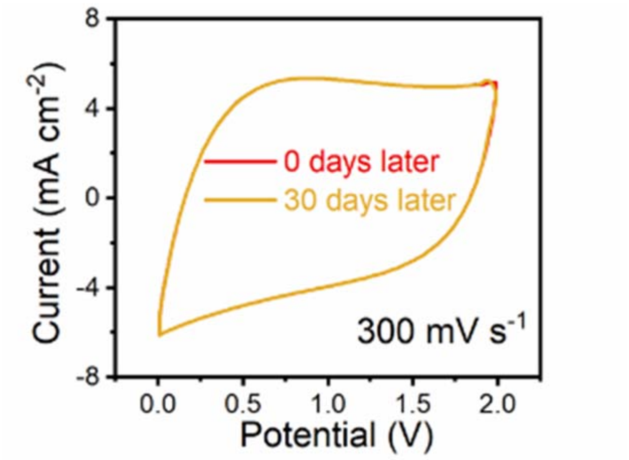

Supplementary Figure 27: CV curves of the fabricated MSCs after 0 and 30 days. To verify the stability of our MSC, we conducted electrochemical tests on the miniature supercapacitor fabricated after 30 days. We found that it exhibited almost the same electrochemical performance as the initial MSC. As shown, under the same scanning rate the CV curves of the two supercapacitors almost coincided. This also indicated that the supercapacitor prepared using a shaped femtosecond laser has highly stable capacitance characteristics.

3.7 Reviewer's Comments

3.7. "I can easily found that several other works with better performances for laser irradiated graphene in the literature, however, authors didn't consider them for comparison with their data."

Our Response

We deeply appreciate the reviewer's efforts taken for reviewing our manuscript. Thank you very much for the suggestion to compare our data with the data of studies showing the better performance of laser-irradiated graphene-based supercapacitors.

By following the reviewer's comments, all co-authors have investigated and reviewed the literature. A list of laser-irradiated graphene-based supercapacitors has been presented, and their performance highlights have been summarized in the revised manuscript. Their data are summarized in Table R3.2. Capacitance performance, energy density, and power density of graphene-based supercapacitors fabricated through laser irradiation (from previous studies) have been compared.

Table R3.2: Comparison of various graphene-based supercapacitors by laser irradiation in capacitance, energy density and power density.

**Electrode materials**	**Capacitance**	**Energy density**	**Power density**	**Ref.**
Laser-processed graphene	3.9 mF cm^{-2}<br>694 mF cm^{-3}	0.98 mWh cm^{-3}	4 W cm^{-3}	31
Laser-induced bi-metal sulfide/graphene	58.3 mF cm^{-2}	7.0 µWh cm^{-2}	1 mW cm^{-2}	32
Laser-induced nitrogen doping of graphene	49 mF cm^{-2}	2.7 µWh cm^{-2}	3 mW cm^{-2}	33
Laser-induced graphene/ PDMS	25 µF cm^{-2}	0.0023 µWh cm^{-2}	2 µW cm^{-2}	34
Laser-induced boron-doped graphene	16.5 mF cm^{-2}	0.5 mWh cm^{-3}	3.5 W cm^{-3}	35
Laser-induced graphene-polyurethane-poly	23 mF cm^{-2}	3.2 µWh cm^{-2}<br>0.53 mWh cm^{-3}	570 µW cm^{-2}<br>95 mW cm^{-3}	36
Laser irradiated Few-Layer Graphene Polyhedra	34.7 mF cm^{-2}	1 mWh cm^{-3}	3 W cm^{-3}	37
Laser-irradiated graphene/ ZnO	9 F cm^{-3}	1.2 mWh cm^{-3}	70 mW cm^{-3}	38
Laser etching□induced graphene-based	8.5 mF cm^{-2}<br>213 mF cm^{-3}	-	-	39
Laser-printed graphene	49 mF cm^{-2}	6.73 mWh cm^{-2}	2.5 mW cm^{-2}	40
Laser-scribed graphene	2.35 mF cm^{-3}	1 mWh cm^{-3}	**200 W cm^{-3}**	18
Laser- reduced graphene oxide/Au	4.92 mF cm^{-2}<br>35 F cm^{-3}	-	-	41
Laser-scribed graphene–MnO_2	**400 mF cm^{-2}**<br>**250 F cm^{-3}**	42 mWh cm^{-3}	10 W cm^{-3}	42

Laser-induced graphene	24 F cm^{-3}	4 mWh cm^{-3}	2 W cm^{-3}	43
Laser-induced graphene /MnO_2/PANI	<u>**934 mF cm^{-2}**</u><br><u>**93.4 F cm^{-3}**</u>	2.4 mWh cm^{-3}	2.89 W cm^{-3}	44
Laser-irradiated graphene	31.9 mF cm^{-2}	1.5 mWh cm^{-3}	0.15 W cm^{-3}	45
Laser □induced graphene/ MnO_2/Fe_2O_3	160 F cm^{-3}	16.3 mWh cm^{-3}	3.54 W cm^{-3}	46
Laser-induced porous graphene	4.2 mF cm^{-2}	-	9 mW cm^{-3}	25
Laser-induced graphene/ MoS_2	16 mF cm^{-2}	4.8 μWh cm^{-2}	0.15 mW cm^{-2}	47
Laser-scribed graphene–Fe_3O_4	<u>**176 mF cm^{-2}**</u><br><u>**19.2 F cm^{-3}**</u>	17.4 mWh cm^{-3}	63 W cm^{-3}	48
Laser-scribed graphene–RuO_2	70 mF cm^{-2}<br>8.6 F cm^{-3}	5 mWh cm^{-3}	4 W cm^{-3}	49
Our work<br>(spatially shaped laser-induced graphene/MnO_2)	128 mF cm^{-2}<br>426.6 F cm^{-3}	70.1 μWh cm^{-2}<br>230 mWh cm^{-3}	40 mW cm^{-2}<br>136 W cm^{-3}	

Through the aforementioned comparison, we found that the supercapacitors in some studies exhibited better performance than our supercapacitors. Most of these performance results are almost reflected in real capacitance and power density, but our energy density remains higher. For a more complete representation, we reconstructed the Ragone plot comparing the energy and power density of different laser-irradiated graphene-based supercapacitors, including some supercapacitors that exhibit more satisfactory performance than our supercapacitor in terms of one parameter (Figure R 3.28).

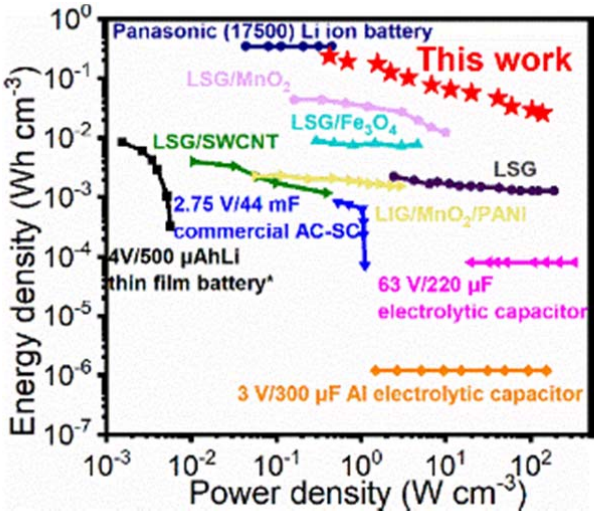

Figure R3.28 Volume-specific Ragone plot comparing the energy and power density of LIG-based supercapacitors and a number of commercially available energy storage devices.

Furthermore, Figure R3.29 presents the area-specific Ragone plot that maps the performance of various laser-irradiated graphene-based supercapacitors. The prepared miniature supercapacitor presents obvious advantages in volume-specific energy density because of its small size, high voltage window, and small thickness. In the same manner, other supercapacitors with large thickness have exceeded in the area-specific Ragone plot. Moreover, this finding reflects that our miniaturized supercapacitor exhibits more advantages and potential in the volume level.

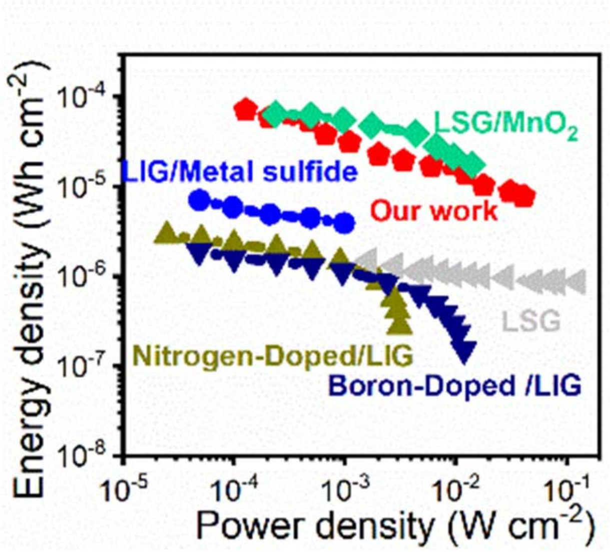

Figure R3.29 The area-specific Ragone plot of various supercapacitors

Modifications

According to the comments of the reviewer, we have revised the corresponding part in our manuscript.

The original part of Figure 5 i in Page 17 is as flowing:

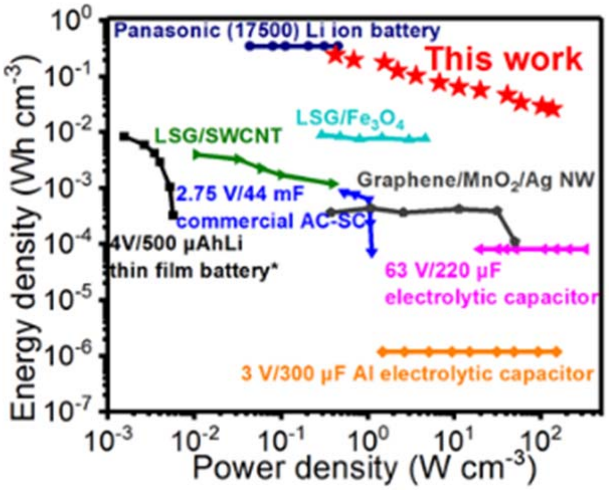

This part is revised as following:

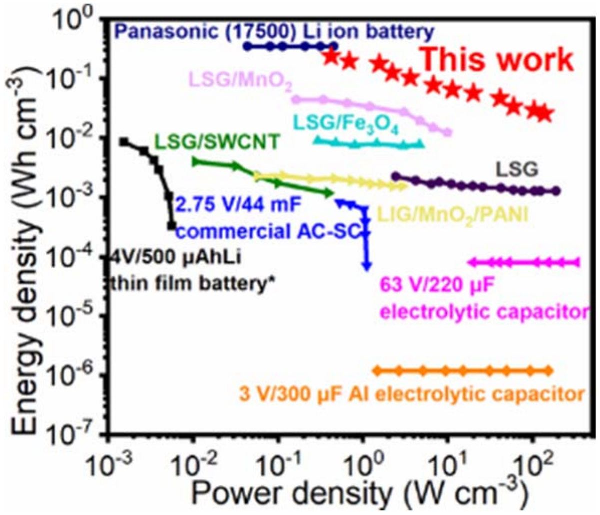

The original part in Page 19, Paragraph 2, Line,5 to 7 the detail is as following:

"This energy density value was also much higher than previous works (graphene/MnO_2/Ag nanowire supercapacitors[57], LIG/carbon nanotubes supercapacitors[58] and LSG/Fe_3O_4 supercapacitors[59])."

This part is revised as following:

"The remarkable energy density of 0.23 Wh cm^{-3} was very similar to the energy

density of Panasonic (17500) Li-ion batteries, several orders of magnitude higher than other capacitors or batteries, and much higher than MSCs presented in previous studies (laser-scribed graphene[31], laser-scribed graphene–MnO_2[62], laser-induced graphene/MnO_2/PANI[63], rGO/carbon nanotubes supercapacitors[64] and LSG/Fe_3O_4 supercapacitors[65])"

[1] Wang, X., & Shi, G. Flexible graphene devices related to energy conversion and storage. Energy & Environmental Science, 8(3), 790-823 (2015).

[2] Hu, C., Song, L., Zhang, Z., Chen, N., Feng, Z., & Qu, L. Tailored graphene systems for unconventional applications in energy conversion and storage devices. Energy & Environmental Science, 8(1), 31-54 (2015).

[3] Li, L., Wu, Z., Yuan, S., & Zhang, X. B. Advances and challenges for flexible energy storage and conversion devices and systems. Energy & Environmental Science, 7(7), 2101-2122 (2014).

[4] Kyeremateng, N. A., Brousse, T., & Pech, D. MSCs as miniaturized energy-storage components for on-chip electronics. Nature nanotechnology, 12(1), 7-15 (2017).

[5] Pech, D., Brunet, M., Durou, H., Huang, P., Mochalin, V., Gogotsi, Y., ... & Simon, P. Ultrahigh-power micrometre-sized supercapacitors based on onion-like carbon. Nature nanotechnology, 5(9), 651-654 (2010).

[6] Wang, K., Zou, W., Quan, B., Yu, A., Wu, H., Jiang, P., & Wei, Z. An all‐solid‐state flexible micro‐supercapacitor on a chip. Advanced Energy Materials, 1(6), 1068-1072 (2011).

[7] Gao, W., Singh, N., Song, L., Liu, Z., Reddy, A. L. M., Ci, L., ... & Ajayan, P. M. Direct laser writing of MSCs on hydrated graphite oxide films. Nature nanotechnology, 6(8), 496-500 (2011).

[8] Cao, L., Yang, S., Gao, W., Liu, Z., Gong, Y., Ma, L., ... & Vajtai, R. Direct laser‐patterned micro‐supercapacitors from paintable MoS2 films. Small, 9(17), 2905-2910 (2013).

[9] Lin, J., Zhang, C., Yan, Z., Zhu, Y., Peng, Z., Hauge, R. H., ... & Tour, J. M. 3-dimensional graphene carbon nanotube carpet-based MSCs with high electrochemical performance. Nano letters, 13(1), 72-78 (2013).

[10] Liu, W., Yan, X., Chen, J., Feng, Y., & Xue, Q. Novel and high-performance asymmetric MSCs based on graphene quantum dots and polyaniline nanofibers. Nanoscale, 5(13), 6053-6062 (2013).

[11] Makino, S., Yamauchi, Y., & Sugimoto, W. Synthesis of electro-deposited ordered mesoporous RuOx using lyotropic liquid crystal and application toward MSCs. Journal of power sources, 227, 153-160 (2013).

[12] Jones, E. T. T., Chyan, O. M., & Wrighton, M. S. Preparation and characterization of molecule-based transistors with a 50-nanometer source-drain separation with use of shadow deposition techniques. Toward faster, more sensitive molecule-based devices. Journal of the American Chemical Society, 109(18), 5526-5528 (1987).

[13] Hsia, B., Kim, M. S., Vincent, M., Carraro, C., & Maboudian, R. Photoresist-derived porous carbon for on-chip MSCs. Carbon, 57, 395-400 (2013).

[14] Beidaghi, M., & Wang, C. Micro‐supercapacitors based on interdigital electrodes of reduced graphene oxide and carbon nanotube composites with ultrahigh power handling performance. Advanced Functional Materials, 22(21), 4501-4510 (2012).

[15] Shen, C., Wang, X., Li, S., Zhang, W., & Kang, F. A high-energy-density micro supercapacitor of asymmetric MnO2–carbon configuration by using micro-fabrication technologies. Journal of power sources, 234, 302-309 (2013).

[16] Chen, C. H., Tsai, D. S., Chung, W. H., Lee, K. Y., Chen, Y. M., & Huang, Y. S. Electrochemical capacitors of miniature size with patterned carbon nanotubes and cobalt

hydroxide. Journal of Power Sources, 205, 510-515 (2012).

[17] Pech, D., Brunet, M., Taberna, P. L., Simon, P., Fabre, N., Mesnilgrente, F., ... & Durou, H. Elaboration of a microstructured inkjet-printed carbon electrochemical capacitor. Journal of Power Sources, 195(4), 1266-1269 (2010).

[18] El-Kady, M. F., & Kaner, R. B. Scalable fabrication of high-power graphene MSCs for flexible and on-chip energy storage. Nature communications, 4, 1475 (2013).

[19] Zhang, C., Kremer, M. P., Seral‐Ascaso, A., Park, S. H., McEvoy, N., Anasori, B., ... & Nicolosi, V. Stamping of flexible, coplanar micro‐supercapacitors using MXene inks. Advanced Functional Materials, 28(9), 1705506 (2018).

[20] Liu, Y., Weng, B., Xu, Q., Hou, Y., Zhao, C., Beirne, S., ... & Chen, J. Facile fabrication of flexible MSC with high energy density. Advanced Materials Technologies, 1(9), 1600166 (2016).

[21] Liu, Z., Wu, Z. S., Yang, S., Dong, R., Feng, X., & Muellen, K. Ultraflexible in-plane MSCs by direct printing of solution-processable electrochemically exfoliated graphene. Advanced Materials, 28(11), 2217-2222 (2016).

[22] Wang, W, Liu, W., Zeng, Y. Han, Y, Yu, M., & Lu, X., et al. A novel exfoliation strategy to significantly boost the energy storage capability of commercial carbon cloth. Advanced Materials, 27(23), 3572-3578 (2015).

[23] Jeon, H., Jeong, J. M., Hong, S. B., Yang, M., Park, J., Hwang, S. Y., & Choi, B. G. Facile and fast microwave-assisted fabrication of activated and porous carbon cloth composites with graphene and MnO2 for flexible asymmetric supercapacitors. Electrochimica Acta, 280, 9-16 (2018).

[24] Shao, Y., El-Kady, M. F., Wang, L. J., Zhang, Q., Li, Y., Wang, H., ... & Kaner, R. B. Graphene-based materials for flexible supercapacitors. Chemical Society Reviews, 44(11), 3639-3665 (2015).

[25] Lin, J., Peng, Z., Liu, Y., Ruiz-Zepeda, F., & Tour, J. M. Laser-induced porous graphene films from commercial polymers. Nature Communications, 5, 5714 (2014)

[26] Peng, Z., Lin, J., Ye, R., Samuel, E. L., & Tour, J. M. Flexible and stackable laser-induced graphene supercapacitors. ACS applied materials & interfaces, 7(5), 3414-3419 (2015).

[27] Wu, Z. S., Parvez, K., Feng, X., & Müllen, K. Photolithographic fabrication of high-performance all-solid-state graphene-based planar MSCs with different interdigital fingers. Journal of Materials Chemistry A, 2(22), 8288-8293 (2014).

[28] Fu, Y., Cai, X., Wu, H., Lv, Z., Hou, S., Peng, M., ... & Zou, D. Fiber supercapacitors utilizing pen ink for flexible/wearable energy storage. Advanced materials, 24(42), 5713-5718 (2012).

[29] Huang, T., Chu, X., Cai, S., Yang, Q., Chen, H., Liu, Y., ... & Gao, C. Tri-high designed graphene electrodes for long cycle-life supercapacitors with high mass loading. Energy Storage Materials, 17, 349-357 (2019).

[30] Chen, S., Wu, B., Qian, H., Wu, Z., Liu, P., Li, F., ... & Liu, B. An asymmetric supercapacitor using sandwich-like NiS/NiTe/Ni positive electrode exhibits a super-long cycle life exceeding 200 000 cycles. Journal of Power Sources, 438, 227000 (2019).

[31] Xie, B., Wang, Y., Lai, W., Lin, W., Lin, Z., Zhang, Z., ... & Kang, F. Laser-processed graphene based MSCs for ultrathin, rollable, compact and designable energy storage components. Nano Energy, 26, 276-285 (2016).

[32] Zhou, C., Hong, M., Yang, Y., Yang, C., Hu, N., Zhang, L., ... & Zhang, Y. Laser-induced bi-metal sulfide/graphene nanoribbon hybrid frameworks for high-performance all-in-one fiber supercapacitors. Journal of Power Sources, 438, 227044 (2019).

[33] Kim, K. Y., Choi, H., Van Tran, C., & In, J. B. Simultaneous densification and nitrogen doping of laser-induced graphene by duplicated pyrolysis for supercapacitor applications. Journal of Power Sources, 441, 227199 (2019).

[34] Parmeggiani, M., Zaccagnini, P., Stassi, S., Fontana, M., Bianco, S., Nicosia, C., ... & Lamberti, A. PDMS/Polyimide Composite as an Elastomeric Substrate for Multifunctional Laser-Induced Graphene Electrodes. ACS applied materials & interfaces, 11(36), 33221-33230 (2019).

[35] Peng, Z., Ye, R., Mann, J. A., Zakhidov, D., Li, Y., Smalley, P. R., ... & Tour, J. M. Flexible boron-doped laser-induced graphene MSCs. ACS nano, 9(6), 5868-5875 (2015).

[36] Tehrani, F., Beltrán - Gastélum, M., Sheth, K., Karajic, A., Yin, L., Kumar, R., ... & Mueller, M. Laser - Induced Graphene Composites for Printed, Stretchable, and Wearable Electronics. Advanced Materials Technologies, 4(8), 1900162 (2019).

[37] Liu, C., Liang, H., Wu, D., Lu, X., & Wang, Q. Direct Semiconductor Laser Writing of Few - Layer Graphene Polyhedra Networks for Flexible Solid - State Supercapacitor. Advanced Electronic Materials, 4(7), 1800092 (2018).

[38] Amiri, M. H., Namdar, N., Mashayekhi, A., Ghasemi, F., Sanaee, Z., & Mohajerzadeh, S. Flexible micro supercapacitors based on laser-scribed graphene/ZnO nanocomposite. Journal of Nanoparticle Research, 18(8), 237 (2016).

[39] Li, T., Cao, Y., Xue, W., Sun, B., & Zhu, D. Self-assembly of graphene-based planar MSC with selective laser etching-induced superhydrophobic/superhydrophilic pattern. SN Applied Sciences, 2(2), 1-9 (2020).

[40] Thekkekara, L. V., & Gu, M. Large-scale waterproof and stretchable textile-integrated laser-printed graphene energy storages. Scientific reports, 9(1), 1-7 (2019).

[41] Li, R. Z., Peng, R., Kihm, K. D., Bai, S., Bridges, D., Tumuluri, U., ... & Hu, A. High-rate in-plane MSCs scribed onto photo paper using in situ femtolaser-reduced graphene oxide/Au nanoparticle microelectrodes. Energy & Environmental Science, 9(4), 1458-1467 (2016).

[42] El-Kady, M. F., Ihns, M., Li, M., Hwang, J. Y., Mousavi, M. F., Chaney, L., ... & Kaner, R. B. Engineering three-dimensional hybrid supercapacitors and MSCs for high-performance integrated energy storage. Proceedings of the National Academy of Sciences, 112(14), 4233-4238 (2015).

[43] Cai, J., Lv, C., & Watanabe, A. Laser direct writing of high-performance flexible all-solid-state carbon MSCs for an on-chip self-powered photodetection system. Nano Energy, 30, 790-800 (2016).

[44] Li, L., Zhang, J., Peng, Z., Li, Y., Gao, C., Ji, Y., ... & Fei, H. High - performance pseudocapacitive MSCs from laser - induced graphene. Advanced Materials, 28(5), 838-845 (2016).

[45] Cai, J., Lv, C., & Watanabe, A. Cost-effective fabrication of high-performance flexible all-solid-state carbon MSCs by blue-violet laser direct writing and further surface treatment. Journal of Materials Chemistry A, 4(5), 1671-1679 (2016).

[46] Lee, J., Seok, J. Y., Son, S., Yang, M., & Kang, B. High-energy, flexible MSCs by one-step laser fabrication of a self-generated nanoporous metal/oxide electrode. Journal of Materials

Chemistry A, 5(47), 24585-24593 (2017).

[47] Clerici, F., Fontana, M., Bianco, S., Serrapede, M., Perrucci, F., Ferrero, S., ... & Lamberti, A. In situ MoS2 decoration of laser-induced graphene as flexible supercapacitor electrodes. ACS applied materials & interfaces, 8(16), 10459-10465 (2016).

[48] Hwang, J. Y., El-Kady, M. F., Li, M., Lin, C. W., Kowal, M., Han, X., & Kaner, R. B. Boosting the capacitance and voltage of aqueous supercapacitors via redox charge contribution from both electrode and electrolyte. Nano Today, 15, 15-25 (2017).

[49] Hwang, J. Y., El-Kady, M. F., Wang, Y., Wang, L., Shao, Y., Marsh, K., ... & Kaner, R. B. Direct preparation and processing of graphene/RuO2 nanocomposite electrodes for high-performance capacitive energy storage. Nano Energy, 18, 57-70 (2015).

Reviewer #1 (Remarks to the Author):

In my original review report I challenged the authors about the novelty in two sides from energy material chemistry and laser manufacturing. Although the authors have carefully tried to address my criticisms and questions I do not think that authors have successfully addressed these challenges. Let me explain my judgement from these sides:

1. Energy material chemistry: the authors have chase GO/manganese acetate as precursors for supercapacitor. Since neither reduce GO or MnO2 nanoparticles are novel for supercapacitors, the authors have to display that this system can display superior capacitance performance. The authors reported the areal capacitor is 128 mF/cm2 at 50mV/s. Indeed a simple electroplating of MnO2 nanoparticle onto the laser writing carbon capacitors can easily display 300 mF/cm2 (for example, J. Laser Appl. 29 (2017) 022203).

The authors claimed that GO/manganese acetate can induce the simultaneous reduction of GO and oxidization of Mn2+ to Mn4+. Although more evidence is needed to confirm such a reacting scheme the authors have to display the scientific merits of such a reaction if it is true, i.e., does this improve the reduction of GO and/or improve the porosity, enhance the surface area, and further these result in higher conductivity or capacitance? For their XPS results, the reduction of graphene oxide can be quantitatively deduced from the C/O ratio. From Table 1s, this C/O ratio is only 5.4, much lower than extensively reported values more than 10. This indicates there is no improvement of GO reduction in such a composite. Their Raman spectra is not presented in quantitative. But, there is no evidence to form nanoporosity in graphene. They did not provide the characterization of the global porosity if this is of benefits to improve the capacitance.

They do show there is minor Mn2O3 formed at a high laser fluence. But what is the significance of Mn2O3? Why should we care the minor Mn2O3?

Therefore, there is limited novelty from the viewpoint of energy materials.

2. Laser manufacturing. Using a special phase modulator is well known for a stereolithography, which facilitate the manufacturing in 2D without scanning. The current work is similar by using a linear photo-thermal effect, which induces the GO reduction and Mn oxidization. Although the authors explain in detail how a holographic imaging form through a simulation and phase control of phase modulator, they failed to unveil progress beyond a stereolithorgraphy. Fig. 1 is still seriously misled. How many capacitors can be formed their holography? If only one, then a X-Y san is still needed to complete a manufacturing in a large area (for an example, 1 cm x 1cm). they do mention the largest area can be fabricated is 100 um x 100 um. Even at this case, they still shift the spot in 100x100 times to complete an area of 1 cm x 1 cm. How many capacitors in 100 um x 100 um? Fig. 1 is also misled for the fabrication at the z-direction. Although they show that the holographic beam can pattern tens micro thickness, they did not study the resolution in the z-direction. Will thy need a z-san for a thick film? It is impossible to form a beam in a cm thickness as shown in Fig. 1. In another word, a z-scan is needed for a 3D stereographic manufacturing unless such a manufacturing is 2D.

The individual capacitor array without proper connection is useless, as shown in many of their figures. Note that they only measure one microcapacior with a microprobe. This is scientific misleading to display so-called million capacitors. Will they want to use one by one? They do display two connected in serial or parallel. It is unclear how they make the connection.

In summary, they do display capacitor array in a large area, but their manufacturing procedures do not support these results.

I do not want to deny some merits of this work but it is hard to believe the current version has satisfied the publication standards of Nature Comm.

Reviewer #2 (Remarks to the Author):

The authors have made a major revision and indeed improved the quality of this work. However, there are still some major concerns need to be addressed before publication of this work. The two critical questions as are:

1. The authors claim that more than 30,000 MSCs can be integrated in 1 cm2. This is quite amazing, if in fact 30,000 MSCs could be integrated together. However, from SEM images demonstrated in Fig. 2c, the gaps between the two interdigited electrodes are not distinct. This may lead to short circuiting of the final fabricated MSCs. Furthermore, it’s difficult to precisely connect the individual MSCs to realize the integration with such a limited distance between the MSCs. The uniform and clear gaps between the individual MSCs should be demonstrated along with the details of the integration methods of the tens of thousands of individual MSCs.
2. Based on the CV curves demonstrated in Fig. 5b, the LIG/MnO2 is no longer stable when the applied voltage is increased to 2.0 V even at a relative high scan rate of 80 mV s-1. This involves obvious electrode polarization and electrolyte decomposition. The asymmetric curve shape and low Coulombic efficiency again demonstrates that the fabricated MSCs cannot achieve such a high stable electrochemical voltage window. In addition, the authors claim that the MSC device can achieve a similar energy density with Panasonic commercialized Li-ion batteries. This could be a very big step if a supercapacitor can achieve the energy density up to the level of a commercial Li-ion battery. All other electrochemical performance including the individual and integrated MSCs should be carefully revisited before its publication to demonstrate such a spectacular claim.

Reviewer #3 (Remarks to the Author):

In the revised manuscript, authors have substantially changed the manuscript and clarified most of the concerns raised by all three reviewers. This manuscript can be accepted, however, a few concerns are still there, which needs to address.

1. The shape diversity study was done in the manuscript according to Fig. 18, SI, however, according to the ai=uthors they have different geometrical areas or the different mass loading after patterning. One can carry out the same mass loading or the area. To do that please follow one recent publication on a similar topic (Energy Environ. Sci., 2019,12, 2507-2517).

2. The cycling performance is shown in figure 5 C is only for 6000 cycle, which is very less and can not be comparable with other microsupercapacitor with very high cycle life (Energy Environ. Sci., 2019,12, 2507-2517, J. Mater. Chem. A, 2020,8, 6740-6756). I will suggest checking the device performance at least up to 10000 cycle.

3. although they have compared the cycle life with some literature, but they again failed to consider two important recent papers on the similar direction as mentioned above.
I will suggest to include this paper in the table and the table can be placed in the supporting information of the article.

4. Although the authors have addressed the comments raised by the reviewers, but the answer to the reviewer's file is too long. Several figures used multiple times, which can be avoided. The to-the-point answers will be appreciated.

Point-by-Point Responses to Reviewers' Comments

Manuscript ID: NCOMMS-20-02591B

Title: Laser photonic-reduction stamping for graphene-based micro-supercapacitors ultrafast fabrication

We are indebted to you and the reviewers for the time and efforts in reviewing our paper. The detailed comments from the referees are very impressive and greatly valued.

1. REVIEWER NO. 1

1.1 Reviewer's Comments

1.1.1 Energy material chemistry: the authors have chase GO/manganese acetate as precursors for supercapacitor. Since neither reduce GO or MnO_2 *nanoparticles are novel for supercapacitors, the authors have to display that this system can display superior capacitance performance. The authors reported the areal capacitor is 128 mF/cm2 at 50mV/s. Indeed a simple electroplating of MnO2 nanoparticle onto the laser writing carbon capacitors can easily display 300 mF/cm2 (for example, J. Laser Appl. 29 (2017) 022203).*

1.1.2 The authors claimed that GO/manganese acetate can induce the simultaneous reduction of GO and oxidization of Mn2+ to Mn4+. Although more evidence is needed to confirm such a reacting scheme the authors have to display the scientific merits of such a reaction if it is true, i.e., does this improve the reduction of GO and/or improve the porosity, enhance the surface area, and further these result in higher conductivity or capacitance? For their XPS results, the reduction of graphene oxide can be quantitatively deduced from the C/O ratio. From Table 1s, this C/O ratio is only 5.4, much lower than extensively reported values more than 10. This indicates there is no improvement of GO reduction in such a composite. Their Raman spectra is not presented in quantitative. But, there is no evidence to form nanoporosity in graphene. They did not provide the characterization of the global porosity if this is of benefits to improve the capacitance.

1.1.3 They do show there is minor Mn_2O_3 *formed at a high laser fluence. But what is the significance of* Mn_2O_3*? Why should we care the minor Mn2O3? Therefore, there is limited novelty from the viewpoint of energy materials.*

1.1.1 Our Response

Thank you very much for your invaluable feedback and for your effort in reviewing our manuscript. In our work, the areal capacitance of our MSCs with a thickness of 3 μm was 128 mF cm^{-2}. The reviewer mentioned that the areal capacitance of 300 mF/cm^2 was obtained under the condition of a relatively large film thickness (60 μm)[R1]. Therefore, it is hard to compare the areal capacitance and ignore the film thickness. Later, we also made a slightly thicker MSC, which realized the areal capacitance of 332 mF/cm^2. On the other hand, our volumetric (426.7 F cm^{-3}) and gravimetric capacitance (941 F g^{-1}) are far higher than that work. The thickness of MSCs in our work is a few microns and a greater thickness is associated with higher capacitance performance. To demonstrate the superior areal capacitance of our MSC, we fabricated 10-μm–thick MSCs and electrochemically tested them at a voltage window of 2 V. Figure R1.1 depicts the CV curves at low and high scan rates of the MSCs with the thickness of 10 μm. The maximum value was 332.8 mF cm^{-2}. Increasing the thickness of the MSCs increases the areal capacitance. However, achieving high energy density using practical materials and synthetic techniques is a major challenge for MSCs. Our MSCs with the thickness of a few microns possess a high volumetric (426.7 F cm^{-3}) capacitance and ultrahigh energy density (0.23 Wh cm^{-3}).

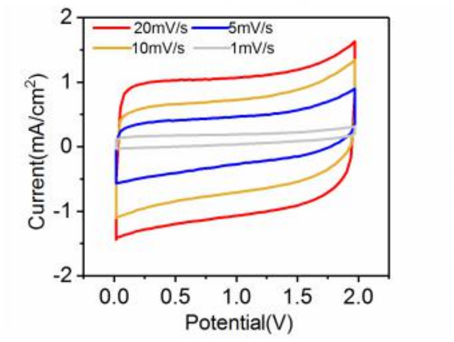

Figure R1.1 The CVs at different scan rates of MSCs with the thicknesses of 10 μm.

1.1.2 Our Response

We are very grateful for the reviewer's detailed comments, which were crucial to improving our paper. We have carefully read the suggestions and clarified the use of the C:O ratio. Table 1s is presented in Supplementary Figure 15 and displays the TEM mapping and EDS of LIG/MnO_2. This presents the proportion of three elements (C, O, and Mn). Notably, the oxygen content comprises the oxygen in the MnO_2 and unreduced graphene.

TableR1.1 TEM EDS of LIG/MnO_2.

	atomic proportions
C	**81.17%**
O	14.67%=8.32%(associated with MnO_2)+**6.35%(associated with graphene)**
Mn	4.16%

The reduction of graphene oxide can be quantitatively deduced from the C:O ratio of graphene oxide. As presented in Table R1.1, the C:O ratio was 12.78. The value "5.4" refers to the C:O ratio of the entire composite material, not the C:O ratio of graphene. A more detailed characterization of XPS is required to calculate the C:O ratio and explore the effects of GO reduction by manganese ions. Figure R1.12 displays the XPS survey spectra for the original GO, laser-induced GO, and laser-induced

GO doped with different proportions of manganese acetate ($GO/Mn_{25\%}$, $GO/Mn_{50\%}$, $GO/Mn_{75\%}$). Figure R1.2-a and -b reveal that both GO and laser-induced GO display carbon and oxygen signals. After laser reduction, the O1s peak intensity of laser-induced GO is significantly reduced compared with the peak intensity of GO, indicating a loss of oxygen. Figures R1.2-c, -d, and -e display the results of the laser-induced GO composite doped with different percentages of Mn^{2+}.

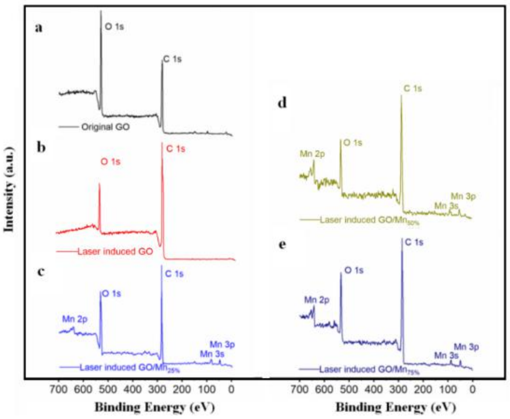

TableR1.2 Atomic concentration table of different samples

Sample	C 1s	O 1s		Mn 2p
		O (associated with graphene)	O (associated with MnO_2)	
Original GO	67.38%	32.62%	/	/
Laser induced GO	87.28%	12.72%	/	/
Laser induced $GO/Mn_{25\%}$	80.20%	7.89%	7.94%	3.97%
Laser induced $GO/Mn_{50\%}$	80.33%	6.08%	9.06%	4.53%
Laser induced $GO/Mn_{75\%}$	78.69%	5.98%	10.22	5.11%

Figure R1.2 XPS survey spectra for the original GO, laser induced GO, laser induced $GO/Mn_{25\%}$, laser induced $GO/Mn_{50\%}$, laser induced $GO/Mn_{75\%}$.Table R1.2 The exact atomic concentration of carbon, oxygen, and manganese.

We used professional equipment (PHI Quantera X-ray photoelectron spectrometer in State key laboratory of tribology Tsinghua University) to obtain the exact atomic concentration of carbon, oxygen, and manganese to accurately calculate the C:O ratio. The results are presented in Table R1.2.

In the reduction of GO, the XPS survey of GO and laser-induced rGO/MnO_2 demonstrate that the C:O ratio increases from 2.06 (GO) to 6.86 (laser-induced GO), 10.16 (laser-induced $GO/Mn_{25\%}$), 13.21 (laser-induced $GO/Mn_{50\%}$), and 13.15 (laser-induced $GO/Mn_{75\%}$). Therefore, the presence of manganese ions contributes to the increase in the C:O ratio in graphene. This finding indicates that composites that include manganese ions achieve a definite improvement in GO reduction. The 6-fold increase represents a marked decrease in the presence of oxygen-containing functional groups at the surface after processing GO/Mn^{2+} into laser-induced rGO/MnO_2. The extent is comparable to the decrease obtained using chemical and thermal reduction methods[R2,3] and studies that have explored UV nanosecond processing of GO under ambient conditions[R4].

To demonstrate nanoporosity in the LIG, we tested the N_2 adsorption-desorption isotherms of the GO mixed film before and after laser irradiation. Figure R1.3-a shows type II N_2 adsorption isotherms displayed by LIG/MnO_2. Remarkably, the Brunauer–Emmet–Teller (BET) specific surface area of LIG/MnO_2 (217.56 $m^2\ g^{-1}$) is approximately eight-times higher than the specific surface area of the GO/Mn^{2+} (27.23 $m^2\ g^{-1}$), which indicates that the porous structure of graphene and MnO_2 nanoparticles increase the specific surface

area of the electrode material after SSFL irradiation, thereby improving the capacitance. The numerous mesopores with sizes of <5 nm (Fig. R1.3 b) accords with the reports of studies[R5,6]. Most pores had sizes of 3.35, 3.70, or 4.05 nm.

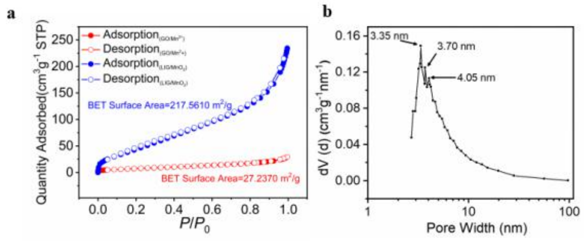

Figure R1.3 (a) Nitrogen adsorption/desorption isotherms of the hybrid GO and LIG/MnO_2 and (b) pore size distribution of the LIG/MnO_2

1.1.3 Our Response

Thank you very much for your careful review of our manuscript. In our last response, we explored the classification of manganese oxides because we observed a small amount of Mn^{3+} in the XPS data, which may interest other reviewers. Therefore, we supplemented relevant experiments and characterizations to explore the differences in the irradiated products of manganese ions and GO with different laser fluence.

Detailed analysis using XPS and XRD revealed that the shaped femtosecond laser can regulate the synthesis of materials and control photoinduced and photothermal-induced reduction and oxidation and manganese valence states. Altering the laser fluence can also yield heterogeneous junctions of different compositions. This is also one of the highlights of our work, which aimed to regulate the synthesis of products by using shaped lasers. We determined that more Mn_2O_3 was produced when the laser fluence was high. Furthermore, the optimal fluence (210 mJ/cm^{-2}) also exhibited a small amount of manganese oxide or structural defects other than MnO_2. Therefore, the femtosecond laser reduces GO and simultaneously facilitates the action of metal ions, which is a novel mechanism of action and provides a new approach to the synthesis of energy materials.

The coexistence of aliovalent cations (Mn^{2+}, Mn^{3+}, and Mn^{4+}) may facilitate the formation of more ionic defects (e.g., vacancies and misplaced ions) and electronic defects (electrons and holes), thereby altering the electronic, ionic, and catalytic properties of the manganese oxides. These defects may accelerate the kinetics of surface redox reactions. Furthermore, the mismatches induced by structural differences in different manganese oxide phases may produce additional defects (cavities, stacking faults, etc.), which facilitate the formation of porous nanoarchitectures that may enhance the transport of charged species and extend the reaction sites from the surface to the subsurface of electrodes[R7]. The in situ X-ray absorption near-edge spectroscopy (XANES) spectra (Fig. R1.4a) of the as-prepared MnO_2 electrodes acquired during the first discharge (0.8 to −0.8 V) revealed a continuous shift of the entire edge toward a lower energy, suggesting that the charge storage is mostly associated with the Mn^{3+}/Mn^{4+} redox reactions, as conventionally believed. However, the XANES spectra (Fig. R1.4b) for the mixed-valent MnO_x collected under identical conditions (except over a wider voltage window from 1.0 to −1.0 V) revealed completely different spectral features. Therefore, mixed-valence manganese oxide materials have a higher voltage

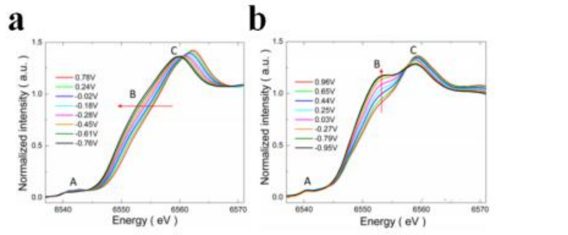

Figure R1.4 Investigation into the charge storage mechanism. (a) In situ XANES spectra of the as-prepared MnO_2 and (b) the mixedvalent manganese oxide electrode during discharge[R7]

window. Theoretical studies have predicted that electronic properties can be tailored through tuning of the defect structure during the synthesis of MnO_2. The results (Fig. R1.5) presented here reveal that a defect in a MnO_2 nanosheet effectively reduces the band-gap energy between occupied and unoccupied electronic states relative to a defect-free layered magnesium oxide nanosheet. The enhancement of electronic properties as a result of introducing defects suggests that photoinduced electronic transitions in MnO_2 nanosheets can be optimized through control over the defect formation rate during the synthesis of self-doped materials[R8,9].

As illustrated in Figure R1.6, CVs were captured at 80 mV/s to evaluate the effect of prepared pure MnO_2 and laser-induced MnO_2. The CV of the prepared pure MnO_2/rGO could maintain satisfactory performance at a voltage of 1.5 V but not at higher voltage windows. The CV curves of SSFL-induced MnO_2/rGO displayed a higher electrochemical performance than pure MnO_2/rGO under the same voltage window and satisfactory performance at a higher voltage window. In summary, we could regulate the synthesis of products photochemically by altering the laser fluence and optimized parameters to achieve the highest electrochemical performance. The coexistence of aliovalent cations and additional defects of MnO_2/rGO theoretically has higher electrical properties than the mixture of pure MnO_2/rGO, which was also demonstrated in our Supplementary Information.

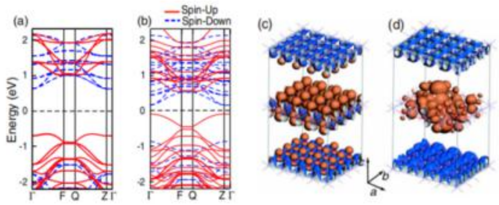

Figure R1.5 [H: white, Mn: purple (or dark gray), O:red (or lightest gray)] Band structures of (a) vacancy-free MnO_2 and (b) Defected MnO_2. Views of charge distributions of valence band maximum (VBM) hole states [orange (or light gray)] and conduction band minimum (CBM) electron states [blue (or gray)]and (d) its defected MnO_2 Investigation[R8].

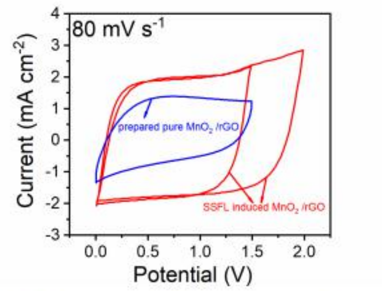

Figure R1.6 CV curves of prepared pure MnO_2/rGO and SSFL induced MnO_2/rGO MSCs obtained at 80 mV s^{-1}

Modifications

Following the reviewer's suggestions, we have made great efforts to further present the novelty of our work in the revised manuscript, where necessary, please see page 8 line 14-16, page 14 line 2-6 and page 20 line 7-10.

The corresponding contents (Fig. R1.3, Fig. R1.4 and Table R1.2) are added in Supplementary material as Supplementary Fig.17, Fig.6 and Table 3.

1.2 Reviewer's Comments

1.2.1 Laser manufacturing. Using a special phase modulator is well known for a stereolithography, which facilitate the manufacturing in 2D without scanning. The current work is similar by using a linear photo-thermal effect, which induces the GO reduction and Mn oxidization. Although the authors explain in detail how a holographic imaging form through a simulation and phase control of phase modulator, they failed to unveil progress beyond a stereolithorgraphy. Fig. 1 is still seriously misled. How many capacitors can be formed their holography? If only one, then a X-Y san is still needed to complete a manufacturing in a large area (for an example, 1 cm x 1cm). they do mention the largest area can be fabricated is 100 um x 100 um. Even at this case, they still shift the spot in 100x100 times to complete an area of 1 cm x 1 cm. How many capacitors in 100 um x 100 um? Fig. 1 is also misled for the fabrication at the z-direction. Although they show that the holographic beam can pattern tens micro thickness, they did not study the resolution in the z-direction. Will they need a z-san for a thick film? It is impossible to form a beam in a cm thickness as shown in Fig. 1. In another word, a z-scan is needed for a 3D stereographic manufacturing unless such a manufacturing is 2D.

1.2.2 The individual capacitor array without proper connection is useless, as shown in many of their figures. Note that they only measure one microcapacitor with a microprobe. This is scientific misleading to display so-called million capacitors. Will they want to use one by one? They do display two connected in serial or parallel. It is unclear how they make the connection. In summary, they do display capacitor array in a large area, but their manufacturing procedures do not support these results.

1.2.1 Our Response

Thank you very much for your detailed feedback regarding laser manufacturing.

Stereolithography (SLA) is an example of a line-by-line and layer-by-layer manufacturing process based on polymerization. With the development of technology, using a special phase modulator is well known for stereolithography, which facilitates the manufacturing in 2D without scanning. We did extensive literature research on the stereolithography by special phase modulator.

Stereolithography usually used DMD as a special phase modulator to fabricate 3D prototypes[R10,11], the method exposes the entire layer pattern on the surface of the liquid photopolymer. And the planar resolution of the system was found to be approximately 10 μm. The size of the three-dimensional structure processed by this method is generally between a few millimeters and a few centimeters, and the X, Y axis, especially the Z axis, needs to be moved to satisfy the three-dimensional processing. SLA is usually a rapid prototyping method for 3D polymer part fabrication. A polymer (usually highly cross-linked) is formed layer by layer

through photo-induced polymerization; the light source, which is either a highly focused beam or laser, is used to initiate polymerization by photodegrading an initiator to form radicals, cations, or carbene-like species, which complicates the manufacture of other materials using SLA, especially active electrode materials.

For our technology (SSFL), the processing mechanism of our technology is different from SLA additive manufacturing by UV-light or laser-induced polymerization. The selective generation of electrode materials was realized through laser reduction and oxidation, modification, additive and subtractive manufacturing. The SSFL technique is particularly attractive because it is suitable for numerous material systems, which breaks the limitations of SLA technology for processing materials. We used the SSFL pattern on Ti_3C_2 MXene, MoS_2, PEDOT, polymers, metals and metal organic frameworks in one step.

On the other hand, our SSFL can achieve ultra-fine structure processing, with extremely high resolution (＜500nm), and can obtain an extremely small structure with the size of 10 microns. Compared to SLA technology, we have improved the precision of micro-nano manufacturing and enabling more miniaturized manufacturing without requiring extra time for postcuring.

Furthermore, SLA uses a fixed-focus beam with an X-Y translation stage to solidify the layers, instead of galvanometric mirrors, and a rastering laser to selectively polymerize a liquid polymer resin on a layer-by-layer basis to fabricate 3D objects. The scanning microstereolithography machine processes each layer individually and requires a program to control the movement on the X, Y, and Z axis. The reviewer suggested that “a z-scan is needed for a 3D stereographic manufacturing unless the manufacturing is 2D.” In our work, SSFL focuses on a number of thin film material and we used the Ti: sapphire laser regenerative amplifier system, which can generate 1,000 single pulses per second. Each shaped laser pulse can fabricate a single MSC, resulting in the fabrication of a single MSC in only 0.001 s. The translation platform need only be moved rapidly along the X- or Y-axis to perform rapid large-area machining.

The laser repetition frequency must be adjusted according to the size of the MSCs to satisfy the machining requirements under the reported experimental equipment and conditions (an X–Y stage with a maximum speed of 2,000 μm/s), otherwise the obtained MSCs overlap. When the scanning speed of the X-axis is 2,000 μm/s, the translation platform can accurately move 2,000 μm per second. A total of 20, 40, and 100 MSCs can be realized on a single path of 2,000 μm in 1 s when the size of the laser spots is 100 × 100, 50 × 50, and 20 × 20 μm^2, respectively. The laser repetition frequency at this time is also adjusted to 20, 40, and 100 shaped laser subpulses per second. Therefore, the laser remains on at all times. We could achieve the highest machining efficiency by adjusting the repetition frequency of the laser pulse and moving the stage as quickly as possible. Therefore, we could control the stage at a speed of 2,000 μm/s and spacing

of 100 μm to scan an area of 1 × 1 cm^2 along the X–Y direction to complete an area of 1 × 1 cm^2, as the reviewer mentioned. Designing other complex procedures or shifting the spot to 100 × 100 times is unnecessary. Our SSFL method is highly operable and efficient. The actual processing speed observed exceeded 3,000 MSCs/min, which is tens or hundreds of times more efficient than previously reported processes for fabricating MSCs. Approximately 1.8 million MSCs can be fabricated in 30 min if the translation stage maintains an accuracy of <1 μm and the laser repetition frequency is adjusted to 1,000. According to the comments of the reviewer, we have added the corresponding part in Supporting Information 1.5.

The aim of this study was to develop an method for fabricating MSCs, especially ultrathin MSCs. Ultrathin MSCs can store energy in a very small volume and have suitable dependence and flexibility. In our previous response, we displayed a cross-section view of the processing films of different thicknesses and provided a detailed characterization (in the last response 1.1.3), which can prove the processing capacity of the SSFL in the z-axis direction, and a porous 3D composite material synthesized by SSFL.

MSCs that are tens of microns thick are relatively common among prepared ultrathin MSCs, and this is also the thickness range that can be fully processed by a laser. These MSCs do not require processing in the Z-axis direction and can be fabricated directly using a laser[R5,12-14]. Figure R1.7 summarizes four studies on the direct processing of graphene-based MSCs by using lasers. These studies reported the processing of the Z-axis without scanning in the Z-axis direction and obtained 3D porous structures of graphene. The thickness of the MSCs prepared was several microns or tens of microns thick.

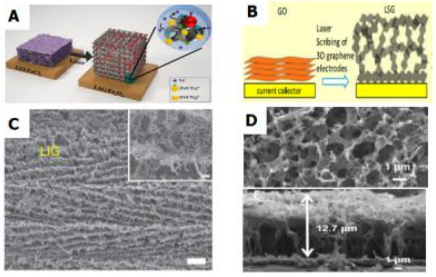

Figure R1.7 a, Schematic illustration showing the fabrication process for 3D-LSG/Fe_3O_4 nanocomposite electrodes by laser[R12]. b, Schematic diagram presents the fabrication procedure for the LSG electrodes[R13]. c and d, SEM images of the 3D laser induced graphene film[R14].

1.2.2 Our Response

Thank you very much for your time and effort in reviewing our manuscript. We designed individual light fields to prepare separate arrays of MSCs of different shapes to highlight their integrity and facilitate readers' shape perception at a glance. However, we designed corresponding light fields to prepare self-assembling and serial-parallel MSCs arrays because these independent MSCs must be combined in practical processing. We could directly obtain the array of multiple MSCs in series by controlling the spacing between the MSCs, and the multiple MSCs connected well with each other. As presented in Fig. R1.8-a, multiple different shapes of MSCs can be obtained in series. Video 2 in the Supplementary material

also illustrates the process of directly and rapidly preparing multiple MSCs in a series.

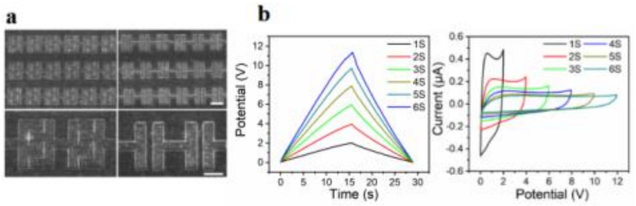

Figure R1.8 a, SEM of MSCs in series with different shapes (scale bar of the figure above and below is 100 and 50 μm) b, CV curves at 300 mV s^{-1} and GCD profiles at 8 mA cm^{-2} obtained from series (1S to 6S)

As projected, the interdigital MSCs connected in series, from a single device to six devices, which revealed the triangular charge-discharge curves and nearly rectangular shapes of EDLC behaviors. The working voltage increased from 2 to 11.39 V (Fig. R1.8-b). To obtain an array of parallel MSCs, we connected MSCs end to end so that we only needed to improve the shape of the light field to satisfy the processing requirements. We modified the design of the pattern to produce the light field illustrated in Figure R1.9-a. We could rapidly fabricate multiple MSCs in parallel by adjusting the spacing between individual laser spots. The MSCs fabricated using this method have suitable consistency and can be assembled arbitrarily. As illustrated in Figure R 1.9-b, the parallel MSCs arrays are obtained after laser spots processing the thin film material in order.

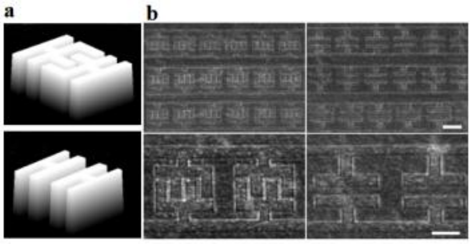

Figure R1.9 a, The optical field distribution diagram optimized by the algorithm, b, SEM of MSCs in parallel with different shapes (scale bar of the figure above and below is 100 and 50 μm)

We could also obtain the array of serial and parallel MSCs (Fig.R1.10-a). Only a simple operation was required to process serial and parallel MSC arrays, and the processing area could be adjusted according to the requirements of microdevices of different sizes. The corresponding contents are added in Supporting Information (Supplementary Figure 3).

Figure R1.10-b presents typical MSC modules with 25 interdigital devices (Fig. R1.10-a), arranged as five parallel rows of five serially connected devices (5S × 5P). The current and capacitance of the five rows in parallel increased nearly five-fold compared with a single row (5S × 1P), attaining a working voltage of 9.69 V.

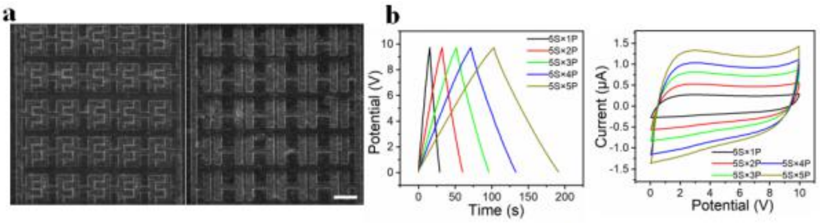

Figure R1.10 a, The SEM image of 5×5 MSCs arrays with different shapes (scale bar,100 μm). b, The CV curves and GCD obtained from mS×nP (m = 5 and n = 1 to 5) configured module where m = number of cells in series and n = number of cells in parallel.

2. REVIEWER NO.2

2.1 Reviewer's Comments

2.1. The authors claim that more than 30,000 MSCs can be integrated in 1 cm2. This is quite amazing, if in fact 30,000 MSCs could be integrated together. However, from SEM images demonstrated in Fig. 2c, the

gaps between the two interdigited electrodes are not distinct. This may lead to short circuiting of the final fabricated MSCs. Furthermore, it ’ s difficult to precisely connect the individual MSCs to realize the integration with such a limited distance between the MSCs. The uniform and clear gaps between the individual MSCs should be demonstrated along with the details of the integration methods of the tens of thousands of individual MSCs.

Our Response

Thank you very much for your detailed and invaluable feedback and for your time and effort in reviewing our manuscript. Your constructive feedback has helped us revise our manuscript, which has enabled us to improve the quality of our manuscript.

In our paper, we ignored the demonstration of series and parallel of MSCs. As reviewers have pointed out, it is difficult to connect individual MSCs in such size. But the SSFL strategy can solve this problem in our work. In order to minimize the length of the reply, the editor suggested that if there are any figures, points repeated then you may wish to place them at one instance and point the referees.

We have made detailed answers and analysis of experimental data in **1.2.2 Our Response,** which showed the series and parallel connections of multiple MSCs clearly and performed the detailed electrochemical tests. We would be very grateful if you could refer to the above reply.

2.2 Reviewer’s Comments

2.2. Based on the CV curves demonstrated in Fig. 5b, the LIG/MnO2 is no longer stable when the applied voltage is increased to 2.0 V even at a relative high scan rate of 80 mV s-1. This involves obvious electrode polarization and electrolyte decomposition. The asymmetric curve shape and low Coulombic efficiency again demonstrates that the fabricated MSCs cannot achieve such a high stable electrochemical voltage window. In addition, the authors claim that the MSC device can achieve a similar energy density with Panasonic commercialized Li-ion batteries. This could be a very big step if a supercapacitor can achieve the energy density up to the level of a commercial Li-ion battery. All other electrochemical performance including the individual and integrated MSCs should be carefully revisited before its publication to demonstrate such a spectacular claim.

Our Response

We deeply appreciate the reviewer for the suggestions for improving the quality of our paper. We are grateful to receive such excellent advice to improve our paper quality.

Electrode polarization and electrolyte decomposition occur easily in MSCs at low scan rates and high

voltages. We aim to avoid this situation through the use of a favorable material design and neutral electrolyte to obtain higher Coulomb efficiency. The CV and GCD curves indicated that the symmetric MSCs possess a high working voltage of 2.0 V, which is considerably higher than the working voltage of the average MSCs. However, as mentioned by the reviewer, an asymmetrical curve was observed in our MSCs under a high voltage window (Fig. 5b). In our initial experiments, to explore the extension of the voltage window, we ignored the curve changes under the high voltage window.

We reviewed the literature to understand and analyze this behavior and improved the experimental conditions to optimize our experiment. When the MSCs were tested at much higher voltages, considerably above the upper limit for water decomposition (H_2O) into oxygen (O_2) and hydrogen (H_2) gas, additional faradaic current contributions were observed. Although this phenomenon is considerably limited by neutral electrolytes and material systems which we mentioned in the manuscript, the faradaic current increased steadily with the cell voltage (from 1.5 to 2.0 V), suggesting that the reaction is under kinetic control[R15]. For a fixed cell voltage of ≥1.5 V, the current response was the sum of two contributions: a pseudocapacitive current (i_c) originating from MnO_2 and a faradaic current (i_f) originating from water electrolysis. For V ≥ 1.5 V, at low and medium scan rates, the faradaic current was more apparent on the capacitive current. However, having a microsecond time constant τ of 10.6 μs, the MSCs can maintain capacitive behaviors at ultrahigh voltages[R16,17]. This phenomenon explains the atypical extension of the MSCs voltage at 2.0 V. In the present study, the ultrafast time constant can be attributed to several factors. First, the MSC fabricated by SSFL is tens of microns in size. Therefore, charge transfer and energy storage can be performed rapidly. Second, the smaller interspace reduced the length of the transport path of ions from one electrode to the counter electrode, resulting in reduced internal resistance and the complete use of active materials. During the process of revising this manuscript, we continually optimized the experimental results, including the numerous parameters in the last response. We optimized the regularity of the shape of the MSCs, selected the optimal narrow gap, and created a more detailed extension of the high voltage window because the regularity of the MSC pattern and narrow gap affect the performance of the MSCs under high voltages. Figure R2.1 displays the performance of the voltage window extension of our optimized MSCs. We originally tested the scan rate and then challenged the CV curve at lower and higher scan rates to assess the performance of the optimized MSCs over a wider scan rate range. The corresponding contents have been modified in the manuscript.

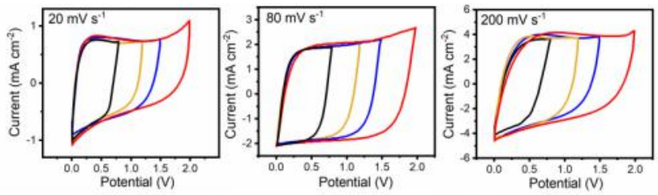

Figure R2.1 CV curves of LIG/MnO_2 MSCs in 0.5 M Na_2SO_4 with different operating voltages from 0.8 to 2 V at different scanning rates.

We determined that the CV curves of optimized MSCs at high voltages were more symmetrical than

before. However, they still exhibited an upwards trend at the end, which indicated electrode polarization and electrolyte decomposition. Therefore, we performed a detailed test and calculation of Coulomb efficiency. Figure R2.2 displays the GCD curves of the optimized interdigital MSCs at various current densities under a voltage window of 2.0 V. The MSCs had triangular charge-discharge curves. The Coulombic efficiency was defined as the total charge removed divided by the total charge added to compensate for the charge removed, as expressed in equation (1)[R2.18].

$$\eta_{\text{Coulombic}} = \frac{\text{Total charge released}}{\text{Total charge stored}} = \frac{\text{Discharging time}}{\text{Charging time}} \quad (1)$$

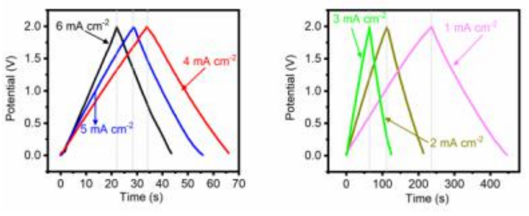

Figure R2.2 The GCD profiles of the MSCs at current density from 1 to 6 mA cm^{-2} under 2 V voltage window.

We calculated the Coulombic efficiency of our MSCs at different current densities under high voltage windows (2 V) using equation (1). In our initial tests, we determined that the charging and discharging times of the GCD curves were similar at different current densities. In the initial cycles, the Coulombic efficiency reached 98%. Moreover, the Coulombic efficiency was higher at a high current density as the number of cycles increased. To investigate the stability of the LIG/MnO_2, the number of cycles was increased to 12,000 to observe the capacitance retention rate and Coulomb efficiency. The performance was more favorable than the original results.

Figure R2.3-a displays the capacitance retention and Coulombic efficiency of the optimized MSCs. The GCD curves after 12,000 cycles are displayed in Figure R2.3-b. The GCD curves indicate high Coulomb efficiency and capacitance retention. Over 95% of the capacitance of each voltage window was retained after 12,000 cycles, and the Coulomb efficiency was close to 100%. These findings demonstrate that our MSCs have favorable electrochemical performance and can achieve a highly stable electrochemical voltage window. The corresponding contents have been modified in the manuscript.

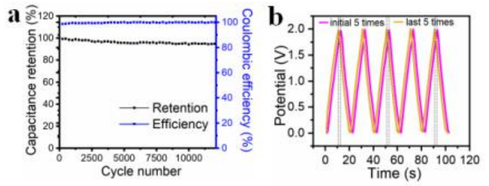

Figure R2.3 (a) Cycle life and Coulombic efficiency of the MSCs, (b) five GCD curves of interdigital MSCs before and after cycling 12,000 times under the voltage windows of 2V.

In our manuscript, we claim that the energy density of our MSCs is similar to that of Panasonic (17500) Li-ion batteries. We did not consider this claim carefully and fully. We measured the electrochemical cycle curves five times at different scan rates and measured the areal capacitance and volumetric capacitance of each scan. We calculated the standard error of the five values using the function. Figure R2.4 displays the statistics of areal capacitance and volumetric capacitance at different scan rates. The error bars represent the standard deviation of the capacitance for each condition.

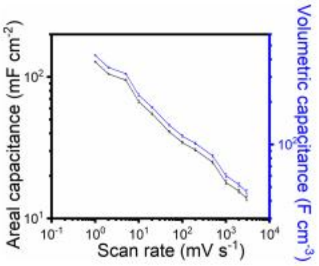

Figure R2.4 Areal capacitance and volumetric capacitance under different scan rates with the error bar of the capacitance.

The development of efficient energy storage capabilities by using advanced materials is a key area of interest. MSCs are promising miniaturized energy storage devices that have attracted considerable research interest. However, their widespread use is limited by inefficient microfabrication technologies and their low energy density. The relatively low energy density hinders scientific research and practical applications[R19,20]. Furthermore, a lack of largescale technologies for the fabrication of electrodes with outstanding mechanical strength limits the development of flexible and deformable MSCs[R21,22]. Therefore, studies have focused on developing advanced structural materials and synthesis technologies for flexible MSCs with high energy densities. Fabrication technologies for producing flexible electrodes with a small volume, flexibility, and deformability are crucial for enabling their practical application[R23].

We propose an ultrafast, one-step, high-resolution, largescale SSFL method for patterning LIG/MnO_2 flexible MSCs. The SSFL technique differs from previously reported methods because it can be used to directly complete the processing of electrical devices in batches without the use of any other methods or laser direct writing. Furthermore, our technology is particularly suitable for ultrathin, difficult-to-process MSCs, which have even lower volume requirements because of their small thickness. Our MSCs are only a few microns thick and can even reach a submicron thickness level. Therefore, the volume of the entire MSC can be considerably reduced, resulting in a higher volumetric capacitance and volumetric energy density. The capacitance of LIG/MnO_2 MSCs was calculated using the following equation:

$$C = \frac{1}{\vartheta \times V}\int_{Vi}^{Vf} I(V)dV \tag{2}$$

where I is the current applied, ϑ is the scan rate and V corresponds to the voltage range (V_f and Vi represent final voltage and the initial voltage respectively). The volumetric energy density of the LIG/MnO_2 MSCs was obtained from the equation:

$$E_{cell} = C_{cell}\Delta E^2 /(2\times 3{,}600) \tag{3}$$

where the E, C and ΔE represent the energy density, capacitance of LIG/MnO_2 MSC and operating voltage, respectively. An increase in the voltage window is of considerable value for increasing MSC energy density. Our MSCs extended the voltage window and achieved excellent electrochemical performance at high voltage windows. Therefore, we considered each parameter that affects energy density and designed the material system and MSC structure to obtain optimal results.

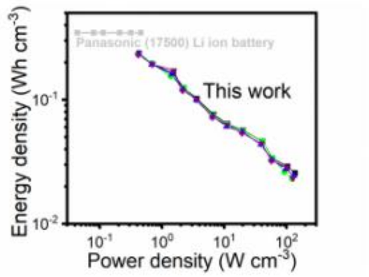

Figure R2.5 Energy and power densities of the LIG/MnO_2 MSCs compared with Panasonic (17500) Li ion battery.

Figure R 2.5 displays five sets of data to illustrate the energy density and power density of the MSCs and avoid data contingency. The five Ragone plots compare the energy density and power density of our MSCs with those of Panasonic (17500) Li-ion batteries. We determined that the highest energy density of our MSCs was close to the energy density of the Panasonic Li-ion batteries. The overall energy density of our MSCs was lower than that of Li-ion batteries. However, Panasonic Li-ion batteries achieve corresponding energy density in practical applications, and the overall performance of our MSC was not comparable. Therefore, our statement was not sufficiently rigorous. The reviewer's comment reminded us that we should maintain a rigorous attitude in scientific research and prompted us to review the comprehensive data and perform new electrochemical tests, which will be valuable for future work.

Modifications

Following the reviewer's suggestions, we have made great efforts to further present the novelty of our work in the revised manuscript, where necessary, please see page 19 line 1-2, figure 5 b and c.

REVIEWER NO.3

3.1 Reviewer's Comments

3.1. The shape diversity study was done in the manuscript according to Fig. 18, SI, however, according to the authors they have different geometrical areas or the different mass loading after patterning. One can carry out the same mass loading or the area. To do that please follow one recent publication on a similar topic (Energy Environ. Sci., 2019,12, 2507-2517).

Our Response

We truly appreciate the reviewer for the efforts for reviewing our paper. We are happy to have received the following suggestions for improving the paper quality. We have modified the corresponding text following the reviewer's comments.

We designed three electrodes with different shapes and mass loading to more comprehensively illustrate the influence of shape on capacitance. Furthermore, we redesigned MSCs of different shapes to have the same mass loading for testing their electrochemical performance, in accordance with another method (Energy Environ. Sci., 2019,12, 2507-2517).Therefore, versatile structural MSCs devices with

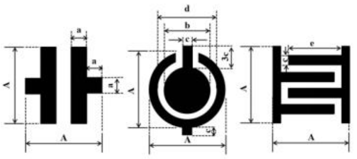

Figure R3.1 Schematics of various shapes of our MSCs (parallel strip, concentric circle, and interdigital) with the same mass loading and area. The values of A, a, b, c, d and e are 50 μm, 10 μm, 32 μm, 5 μm, 45 μm and 30 μm, respectively.

interdigital, parallel strips and concentric circles were fabricated with specific size parameters. As illustrated in Figure R 3.1, the same mass loading or area can be verified using simple mathematical calculations with maintenance of a constant geometrical area of the different structures[R24].

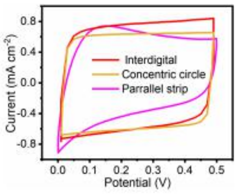

Figure R3.2 CV curves of versatile-shaped MSCs obtained at a scan rate of 50 mV s^{-1} in 0.5 M Na_2SO_4.

The corresponding contents have been added in the Supplementary Information. The CV curves (Fig. R3.2) of all three MSC shapes were rectangular at high or low scan rates, demonstrating the high-quality capacitive performance, shape compatibility, and suitability of the MSCs processed using the SSFL method. Furthermore, the specific capacitance and the shape of the CV graph of the interdigital MSC were of high quality.

Modifications

According to the reviewer's comments, we have revised the corresponding part in our manuscript, where necessary, please see page 14 line 15-20 and Supplementary Information figure 19.

3.2 Reviewer's Comments

3.2. The cycling performance is shown in figure 5 C is only for 6000 cycle, which is very less and can not be comparable with other microsupercapacitor with very high cycle life (Energy Environ. Sci., 2019,12, 2507-2517, J. Mater. Chem. A, 2020,8, 6740-6756). I will suggest checking the device performance at least up to 10000 cycle.

Our Response

We are very grateful to the reviewer for the suggestions and for drawing our attention to the two papers reporting that MSCs can achieve a long cycle life.

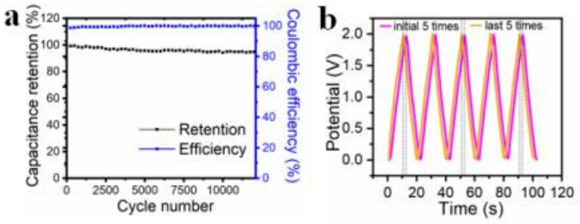

Figure R3.3 (a) Cycle life and Coulombic efficiency of the MSCs, (b) five GCD curves of interdigital MSCs before and after cycling 12,000 times under the voltage windows of 2V.

In accordance with the reviewer's comments, we verified the device performance up to 12,000 cycles. Furthermore, to demonstrate the stable electrochemical performance of our MSCs, we calculated the corresponding Coulomb efficiency. Figure R3.3-a displays the capacitance retention and Coulombic efficiency of our MSCs, and Figure R3.3-b displays the GCD curves after 12,000 cycles, which demonstrate the high Coulomb efficiency and capacitance retention

of the MSCs. Over 95% of the capacitance of each voltage window was retained after 12,000 cycles, and the Coulomb efficiency was nearly 100%. These findings demonstrate that our MSCs have satisfactory electrochemical performance and can achieve a highly stable electrochemical voltage window.

Modifications

Following the reviewer's suggestions, we have made great efforts to further present the novelty of our work in the revised manuscript, where necessary, please see figure 5 c and page 19 line 1-2.

3.3 Reviewer's Comments

3.3. Although they have compared the cycle life with some literature, but they again failed to consider two important recent papers on the similar direction as mentioned above.

I will suggest to include this paper in the table and the table can be placed in the supporting information of the article.

Our Response

Thank you for your suggestions. Cycle life is crucial in practical applications of MSCs. Therefore, we have added a comparison of studies related to cycle life and capacitance retention rate on the basis of information provided in the original table of Supplementary Information.

Supplementary Table 1: Comparison of various manufacturing methods in manufacturing efficiency, size, narrow gap and cycle life of fabricated micro-supercapacitors

Manufacturing methods	Size of supercapacitors (narrow gap)	Manufacturing efficiency /30min	Cycle life (capacitance retention)
Light scribe DVD[R13]	0.5×1 cm^2 (150 μm)	100	10,000 (96%)
3D-Printed stamping[R25]	1×2 cm^2 (400 μm)	24	10,000 (93.7)
Coating and laser etching[R26]	1×1 cm^2 (500 μm)	180	10,000 (94.3%)
Inkjet-printing[R27]	1×2 cm^2 (1000 μm)	100	1,000 (98.5%)
Microwave radiation[R28]	1×2 cm^2	180	10,000 (95%)
Electrochemical synthesis /laser irradiation[R24]	5×7 mm^2(200 μm)	--	100,000 (100%)
Electrochemical polymerization[R29]	2×2 cm^2 (150 μm)	--	10,000 (92%)
SSFL strategy (Our work)	50×50 μm^2 (0.5 μm)	90,000	12,000 (95%)

As displayed in the table, the cycle life of most MSCs is approximately 10,000 times, and the capacitance retention can be maintained above 90%. Our MSCs demonstrated excellent cycle life (12,000) and capacitance retention (95%) under a voltage window of 2 V. Furthermore, we determined that our MSCs achieved an excellent cycle life and capacitance retention under other voltage windows.

We also noticed a method for increasing the cycle life of MSCs to 100,000 cycles with a high capacitance retention (100%)[R24]. The high cycle life can be attributed to the interconnected sheet-like structure of LIG and the minimal defects in its backbone, which facilitate electronic transport during GCD

cycling. The unique porous structure and absence of a metal current collector in the LIG-based MSCs improved the cycling performance.

3.4 Reviewer’s Comments

3.4. Although the authors have addressed the comments raised by the reviewers, but the answer to the reviewer's file is too long. Several figures used multiple times, which can be avoided. The to-the-point answers will be appreciated.

Our Response

Thank you very much for the reminder. We deeply appreciate the reviewer for the suggestions for improving the quality of our paper. We have attempted to simplify the language and reduce redundancy in the figures and main text.

References

[1] Wang, S. et al. (2017). Journal of Laser Applications, 29(2), 022203.
[2] Guo, L. et al. (2012). Carbon, 50(4), 1667-1673.
[3] Zhang, Y. et al. (2010). Nano today, 5(1), 15-20.
[4] Yung, K. C. et al. (2013). Journal of Applied Physics, 113(24), 244903.
[5] Lin, J. et al. (2014). Nature communications, 5(1), 1-8.
[6] Wang, F. et al. (2017). Journal of Laser Micro Nanoengineering, 12(2), 165.
[7] Song, M. et al.(2012). Nano letters, 12(7), 3483-3490.
[8] Kwon, K. D. et al. (2008). Physical review letters, 100(14), 146601.
[9] Wang, Y. et al. (2017). Energy & Environmental Science, 10(4), 941-949.
[10] Miri, A. K.et al. (2018). Advanced Materials, 30(27), 1800242.
[11] Jiang, C.et al.(2014). International journal of precision engineering and manufacturing, 15(11), 2413-2419.
[12] Male, A. T. et al. (2003). Journal of Materials Processing Technology, 135(2-3), 340-346.
[13] El-Kady, M. F., & Kaner, R. B. (2013). S. Nature communications, 4, 1475.
[14] Zhang, W. et al. (2019). Small Methods, 3(5), 1900005.
[15] Bard, A. J. et al. (2001). Electrochemical Methods, 2(482), 580-632.
[16] Kyeremateng, N. A., Brousse, T., & Pech, D. (2017). Nature nanotechnology, 12(1), 7-15.
[17] Ferris, A.et al. (2017). ACS Energy Letters, 2(8), 1734-1739.
[18] Yang, H. et al. (2017). Nanotechnology, 28(44), 445401.
[19] Wang, X., & Shi, G. (2015).Energy & Environmental Science, 8(3), 790-823.
[20] Lv, Q. et al. (2016). Nano letters, 16(1), 40-47.
[21] Lu, C. et al. (2017). Advanced Functional Materials, 27(8), 1606219.
[22] Gao, Y. et al. (2017). CrystEngComm, 19(3), 404-418.
[23] Wu, X. et al. (2018). Nature communications, 9(1), 1-11.
[24] Kamboj, N .et al. (2019). Energy & Environmental Science, 12(8), 2507-2517.
[25] Zhang, C. et al. (2018). Advanced Functional Materials, 28(9), 1705506.
[26] Liu, Y. et al. (2016). Advanced Materials Technologies, 1(9), 1600166.
[27] Liu, Z. et al. (2016). Advanced Materials, 28(11), 2217-2222.
[28] Jeon, H. et al. (2018). Electrochimica Acta, 280, 9-16.
[29] Purkait, T. et al. (2020). Journal of Materials Chemistry A, 8(14), 6740-6756.

Reviewer #1 (Remarks to the Author):

The authors have well addressed my significant concerning on both material science and laser manufacturing. Their arguments on the C/O ratios and the benefits of Mn multi-valent cations are persuaded. Their novelty justification of laser direct writing on the improved resolution compared to stereolithography is reasonable by adding the new data of proper configuration of device arrays potentially connected in parallel and/or in series. Based on these evaluations I would recommend this work for publication.

Reviewer #2 (Remarks to the Author):

Although the authors have provided a number of supplemental experiments to reply to the referees' comments, the responses are still not convincing. First, the resolution of the laser photonic reduction stamping method is relatively low even for the supplemented SEM images provided in the response letter. I find it difficult to believe the authors claimed achievement that more than 30,000 MSCs can be integrated in 1 cm2. Even for the SEM image of the selected laser scribed interdigital pattern, it is very hard to distinguish the isolated gap between the two interdigital electrodes to avoid short circuiting the full device. Second, the authors should present their research results based on the objective facts without overstating their achievements. Third, the authors should get to the point and reply in a straight forward manner to each of the referees' comments rather than give a long winded essay. I don't believe every reviewer has the patience to go through 50 or even 200 pages of a response letter every time. Overall, I cannot recommend this manuscript for publication in Nature Communications.

Reviewer #3 (Remarks to the Author):

The authors have satifactorily addressed all the comments raised by the reviewer 3. Therefore the manuscript can be accepted for publications.

Point-by-Point Responses to Reviewers' Comments

Manuscript ID: NCOMMS-20-02591B

Title: Laser photonic-reduction stamping for graphene-based micro-supercapacitors ultrafast fabrication

We greatly appreciate the excellent, detailed feedback provided by you and the reviewers. We have carefully revised the manuscript and the Supplementary Information section accordingly. All revisions have been highlighted in the text, and we have added the ORCIDs of the lead authors. We have also jointly uploaded the best quality images in the PPT document, as well as the other required documents.

1. REVIEWER NO. 1

Reviewer's Comments

The authors have well addressed my significant concerning on both material science and laser manufacturing. Their arguments on the C/O ratios and the benefits of Mn multi-valent cations are persuaded. Their novelty justification of laser direct writing on the improved resolution compared to stereolithography is reasonable by adding the new data of proper configuration of device arrays potentially connected in parallel and/or in series. Based on these evaluations I would recommend this work for publication.

Our Response

We greatly appreciate the reviewer's efforts in reviewing our paper. Your suggestions and the detailed comments are insightful and critical for improving the manuscript.

2. REVIEWER NO. 2

Reviewer's Comments

Although the authors have provided a number of supplemental experiments to reply to the referees' comments, the responses are still not convincing. First, the resolution of the laser photonic reduction stamping method is relatively low even for the supplemented SEM images provided in the response letter. I

find it difficult to believe the authors claimed achievement that more than 30,000 MSCs can be integrated in 1 cm^2. Even for the SEM image of the selected laser scribed interdigital pattern, it is very hard to distinguish the isolated gap between the two interdigital electrodes to avoid short circuiting the full device. Second, the authors should present their research results based on the objective facts without overstating their achievements. Third, the authors should get to the point and reply in a straight forward manner to each of the referees' comments rather than give a long winded essay. I don't believe every reviewer has the patience to go through 50 or even 200 pages of a response letter every time

Our Response

We thank the reviewer for the efforts for reviewing our paper. In our work, the size of the micro-supercapacitors (MSCs) that we prepared was in the tens of microns. Furthermore, SSFL technology can be used for the rapid and integrated preparation of MSCs. The process used in our work has been detailed in **1.2.1** of the last response. As depicted in Figure 1, a 1 cm^2 area is large enough to accommodate our MSCs, according to the formula: $\frac{(10000\mu m * 10000\mu m)}{(50\mu m * 50\mu m)} = 40000 > 30000$. Therefore, more than 30,000 MSCs can be packed into 1 cm^2 (Figure 2e in our manuscript).

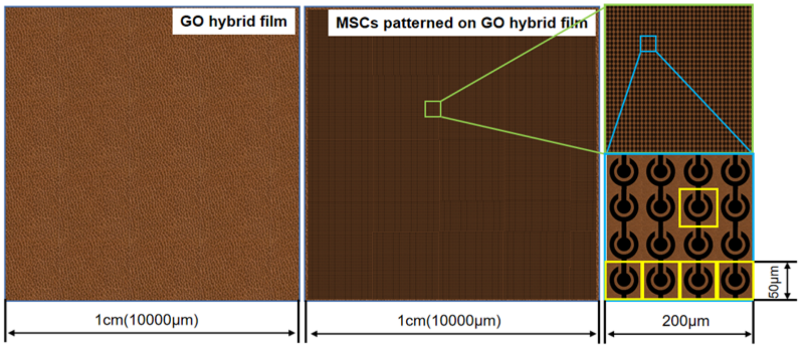

Figure 1 Schematic diagram of the processing area and MSCs arrangement.

As shown in Figure 2, we randomly selected several MSCs for characterization in the array of large-area fabricated MSCs. In Figure 2, each number corresponds to a given region in the photographs. We then randomly selected the MSCs and observed their morphology through SEM, which indicated that the MSCs all have a regular shape and that no short circuit occurred in the devices.

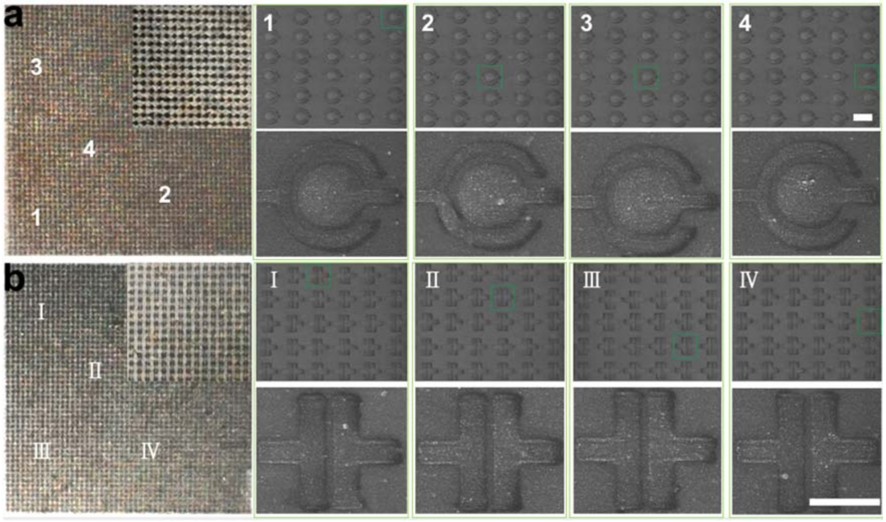

Figure 2 The SEM image of large-scale and locally enlarged MSCs with different shapes

(scale bar, a:100 μm, b:50 μm).

Our processing resolution was realized through the patterned design of the light field shape using the SSFL strategy. To improve the machining effect, we optimized the GS algorithm to achieve high-quality light fields for rapid fabrication of high-resolution MSCs. In **2.2** of the first response, we explained that a spatially shaped femtosecond laser can be used to accomplish high-resolution machining beyond the diffraction limit, and we also presented a complete SEM image of the device. The narrow gap of the device has no short circuits. Furthermore, SSFL has the considerable advantage of allowing for the preparation of many large-scale MSCs. We also performed several cyclic electrochemical tests on the devices to ensure the stability of the fabrication of MSCs.

In our last response, we revised the statement in the manuscript according to the reviewer comments to ensure that our description of our work is grounded in evidence. We sincerely apologize for the initially disproportionate response. We were aware of this problem and reduced this part to a dozen pages in our last response. We have also responded to the reviewer's comment as directly as possible in this response. In our last response, we revised the statement in the manuscript according to the reviewer comments to ensure that our description of our work is grounded in evidence. We sincerely apologize for the initially disproportionate response. We were aware of this problem and reduced this part to a dozen pages in our last

response. We have also responded to the reviewer’s comment as directly as possible in this response.

3. REVIEWER NO.3

Reviewer’s Comments

The authors have satisfactorily addressed all the comments raised by the reviewer 3. Therefore, the manuscript can be accepted for publications.

Our Response

Thank you very much for your detailed and invaluable feedback and for your time and effort in reviewing our manuscript. Your constructive feedback has helped us revise our manuscript, which has enabled us to improve the quality of our manuscript.
